# On the Structure of Intermediates in Enyne Gold(I)‐Catalyzed Cyclizations: Formation of *trans*‐Fused Bicyclo[5.1.0]octanes as a Case Study

**DOI:** 10.1002/chem.202004237

**Published:** 2020-11-19

**Authors:** Imma Escofet, Helena Armengol‐Relats, Hanna Bruss, Maria Besora, Antonio M. Echavarren

**Affiliations:** ^1^ Institute of Chemical Research of Catalonia (ICIQ) Barcelona Institute of Science and Technology (BIST) Av. Països Catalans 16 43007 Tarragona Spain; ^2^ Departament de Química Orgànica i Analítica Universitat Rovira i Virgili (URV) C/ Marcel⋅lí Domingo s/n 43007 Tarragona Spain; ^3^ Departament de Química Física i Inorgànica Universitat Rovira i Virgili (URV) C/ Marcel⋅lí Domingo s/n 43007 Tarragona Spain

**Keywords:** cycloisomerization, cyclopropanation, DFT calculations, dienynes, gold(I) catalysis

## Abstract

The nature of cyclopropyl gold(I) carbene‐type intermediates has been reexamined as part of a mechanistic study on the formation of *cis*‐ or *trans*‐fused bicyclo[5.1.0]octanes in a gold(I)‐catalyzed cascade reaction. Benchmark of DFT methods together with QTAIM theory and NBO analysis confirms the formation of distinct intermediates with carbenic or carbocationic structures in the cycloisomerizations of enynes.

## Graphical abstract

**Going for gold**: The nature of cyclopropyl gold(I) carbene‐type intermediates has been explored as part of a mechanistic study on the gold(I)‐catalyzed formation of *trans*‐fused bicyclo[5.1.0]octanes. Complete computational analysis, that includes QTAIM theory and NBO analysis, confirms the formation of different species with carbenic or cationic nature as intermediates in the cycloisomerizations of enynes.

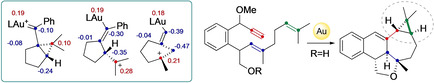

## Introduction

Research in homogenous gold(I) catalysis has provided unique tools for the construction of molecular complexity.^[1]^ Thus, fundamental knowledge gathered in the study of 1,*n*‐enyne cycloisomerizations^[2]^ has led to many applications in total synthesis of complex natural products.^[3]^ Although gold(I) carbenes have been proposed as key intermediates of many gold(I) catalyzed transformations, there is still some uncertainty regarding the structure of these species (carbenic or cationic character of C−Au bond),^[4]^ especially considering their high reactivity, which makes their isolation very challenging.^[5]^ In this context, our group recently reported the spectroscopic characterization of mesityl gold(I) carbenes in solution by NMR at low temperature,^[6]^ which correspond to actual species present under catalytic conditions.

**Scheme 1:**
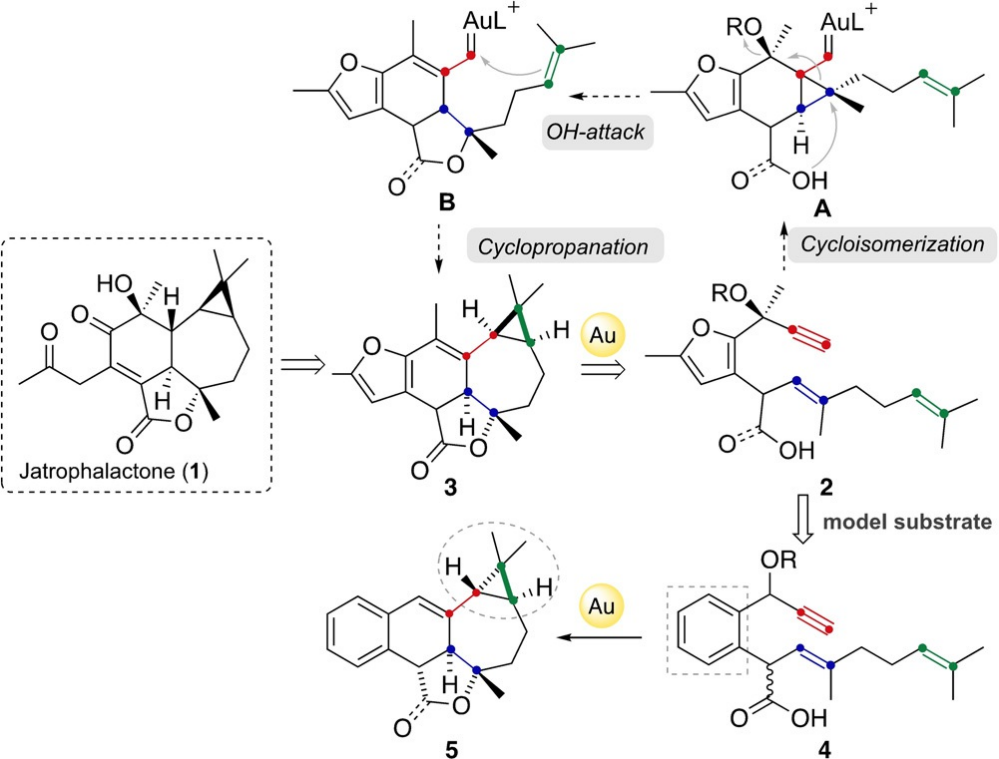
Proposed retrosynthesis of jatrophalactone (**1**) and model substrate **4** for the gold(I)‐catalyzed cycloisomerization key step.

As part of our program on the total synthesis of jatrophalactone (**1**)^[7]^ (Scheme 1), a cytotoxic diterpene isolated from the roots of *Jatropha curcas*, we observed the unexcepted formation of *trans*‐fused bicyclo[5.1.0]octanes (**5**) by a gold(I)‐catalyzed cyclization cascade. To date, no total synthesis of **1** has been reported. We envisioned that after the coordination of gold to the alkyne of dienyne **2**, a 6‐*exo*‐dig cyclization would form cyclopropyl gold carbene **A**, which, after intramolecular nucleophilic attack of the OH moiety to the carbene, followed by OR elimination, would lead to the formation of intermediate **B**. Product **3** would finally be obtained by intramolecular cyclopropanation of the second alkene. In order to explore the feasibility of this transformation, simpler model substrate **4** was designed having a phenyl ring instead of the furan. However, to our surprise, product **5** was obtained in this reaction bearing a rare *trans*‐fused bicyclo[5.1.0]octane.

**Figure 1:**
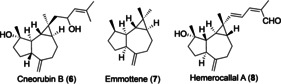
Natural products bearing a *trans*‐fused bicyclo[5.1.0]octane.

Although, the *trans*‐bicyclo[5.1.0]‐octane motif is present in some natural products,^[8]^ such as cneorubin B (**6**), emmottene (**7**), and hemerocallal A (**8**) (Figure 1), the formation of this ring system is rather unusual because it formally corresponds to the cyclopropanation of (*E*)‐cycloheptene, which is unstable at room temperature.^[9]^ This type of *trans*‐fused bicyclo[5.1.0]octanes had only been obtained before as minor byproducts in cyclization cascade reactions catalyzed by gold(I).^[10]^

**Scheme 2:**
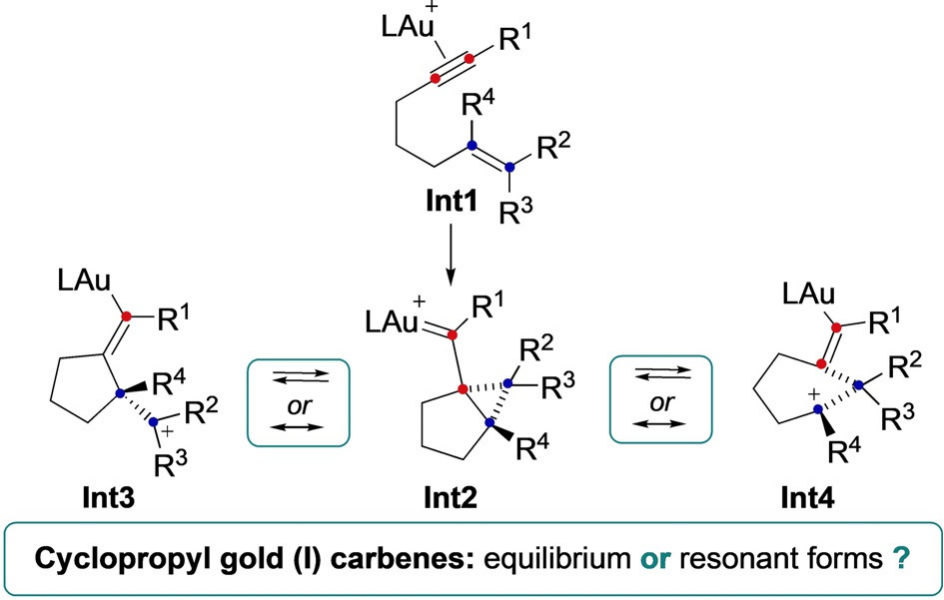
Different structures of cyclopropyl gold(I) carbene‐type intermediates **Int2**, **Int3** and **Int4**.

Herein, we present an experimental and computational study on the selective formation of *cis*‐ or *trans*‐fused bicyclo[5.1.0]octanes by a gold(I)‐catalyzed cascade. This investigation also led us to reconsider the puzzling structure of the cyclopropyl gold(I) carbene‐type intermediates.^[11]^ In principle, upon coordination of gold(I) to the alkyne of the 1,6‐enyne in **Int1**, three different possible structures could be generated, **Int2**‐**4**. Whether or not structures **Int2**‐**4** are resonance forms or distinct stationary points in the reaction coordinate is still an open question and the different interpretations coexist in the current literature^[4]^ (Scheme 2). Thus, we performed DFT calculations (including benchmark of functionals, QTAIM theory, and NBO analysis) in order to further describe the structure of intermediates **Int2**–**4** and how they are interconnected.

## Results and Discussion

### Formation of *trans*‐fused bicyclo[5.1.0]octanes

**Scheme 3:**
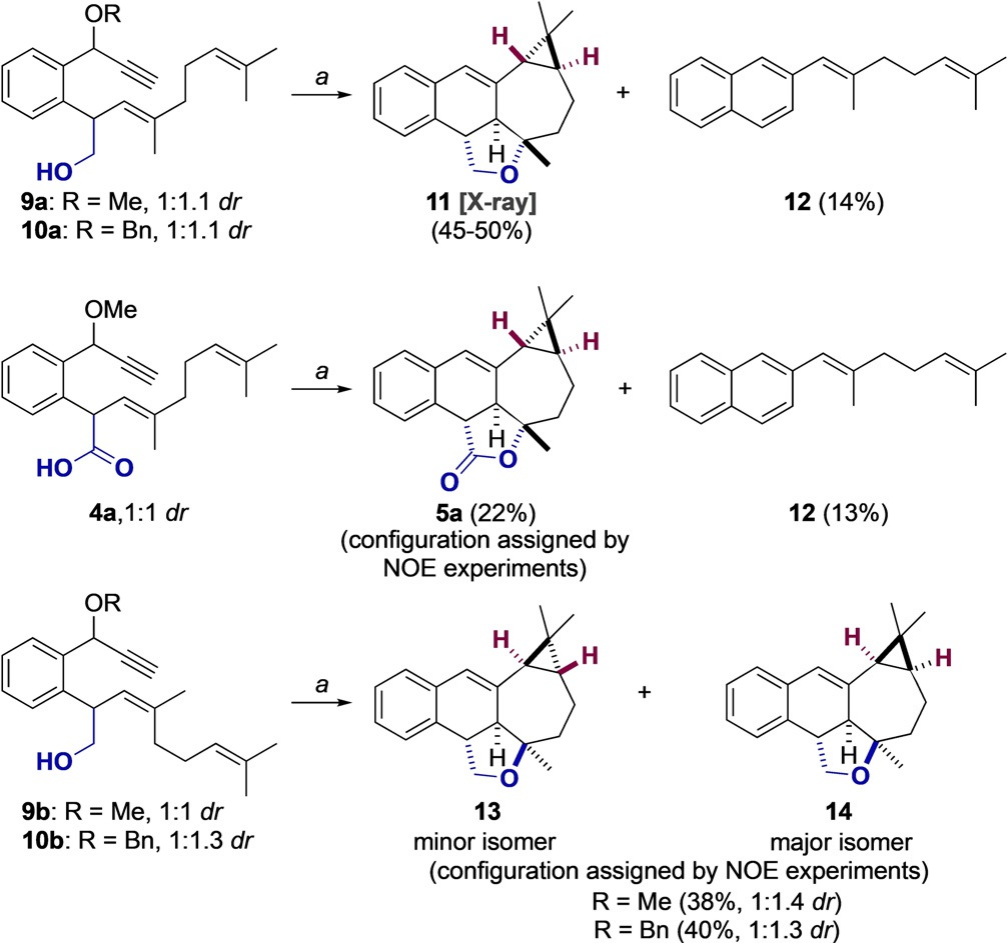
Gold(I) catalysis. a) Standard reaction conditions: [(JohnPhos)Au(MeCN)]SbF_6_ (2 mol %), CH_2_Cl_2_ (0.1 m), 23 °C, 10 min.

**Figure 2:**
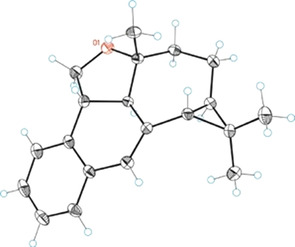
ORTEP structure of pentacyclic compound **11**.

We examined the cyclization of dienynes **4 a**, **9** and **10**, bearing OH or CO_2_H as intramolecular nucleophiles, as models for the key cascade cyclization for the synthesis of jatrophalactone (see Scheme 1). Dienynes **4 a**, **9** and **10** reacted almost instantaneously with [(JohnPhos)Au(MeCN)]SbF_6_ as the catalyst at room temperature in CH_2_Cl_2_ (Scheme 3). Surprisingly, (*E*)‐configured enynes **9 a** and **10 a** (ca. 1:1 diastereomeric mixture at the benzylic positions), afforded **11** as a single diastereomer in 45–50 % yield, along with 2‐substituted naphthalene **12** (14 % yield). The presence of rare *trans*‐fused bicyclo[5.1.0]octane, along with a *trans*‐fused tetrahydronaphtho[1,2‐*c*]furan unit, in compound **11** was confirmed by X‐ray diffraction (Figure 2). Similarly, carboxylic acid **4 a** yielded *trans*‐fused cyclopropane **5 a** as a single diastereomer, albeit in lower yield. Again, naphthalene **12** was isolated in this reaction as a minor product. On the other hand, (*Z*)‐configured dienynes **9 b** and **10 b** gave an inseparable mixture of isomers **13** and **14** in moderate yield, together with traces of naphthalene (*Z*)‐**12** (Scheme 3). Remarkably, when the two diastereomers of **9 b** and **10 b** were separated by chromatography and exposed to gold(I)‐catalysis, the same mixture of **13** and **14** was obtained, although (*Z*)‐**12** was only formed from one of them. The relative configuration of pentacyclic products **13** and **14**, which are clearly distinct from **11**, was assigned by NOE NMR experiments.^[12]^

**Scheme 4:**
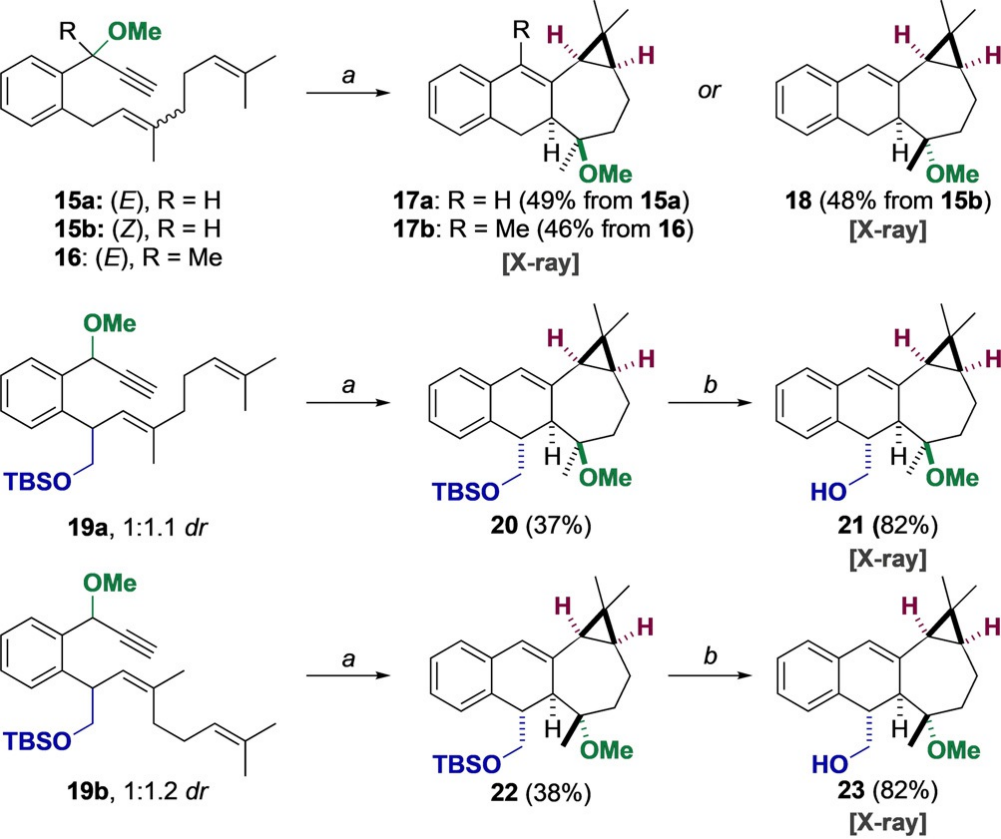
Gold(I) catalysis of modified dienynes. a) [(JohnPhos)Au(MeCN)]SbF_6_ (2 mol %), CH_2_Cl_2_, 23 °C, 10 min; b) THF/ 10 % HCl, 23 °C, 2 h.

To understand the effect of substituents and alkene configuration in the stereoselectivity of these transformations, other dienynes were prepared and submitted to the gold(I)‐catalyzed cascade cyclization (Scheme 4). Therefore, simpler substrates **15 a**,**b** and **16** missing the benzylic hydroxymethyl group gave products **17 a**,**b** and **18** as single diastereomers in ca. 50 % yield. All of these products feature a *cis*‐fused bicyclo[5.1.0]octane system as confirmed by X‐ray diffraction analysis. The cascade reactions proved to be stereospecific with respect to the configuration of the alkene, as observed in many other gold(I)‐catalyzed transformations. Additionally, products **20** and **22** were obtained as single diastereomers by reaction of dienynes **19 a**,**b**. Deprotection of TBS group of **20** and **22** gave rise to crystalline primary alcohols **21** and **23**, whose relative configuration was confirmed by X‐ray diffraction.

**Scheme 5:**
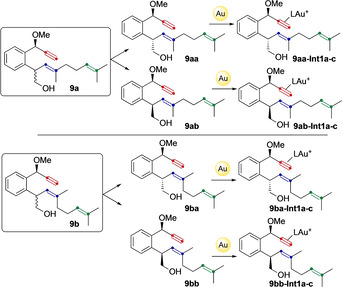
Gold(I)‐catalyzed formation of **Int1 a**–**c** of (*E*)‐configured **9 a** and (*Z*)‐configured **9 b**.

In order to rationalize the formation of *trans*‐fused bicyclo[5.1.0]octanes and the different selectivity observed for (*E*)‐ and (*Z*)‐dienynes in the new gold(I)‐catalyzed cyclization cascade reactions we performed DFT calculations. Calculations were performed with B3LYP^[13]^‐D3^[14]^/6‐31G(d)^[15]^ (C, H, O, P) and SDD^[16]^ (Au) in CH_2_Cl_2_ (PCM)^[17]^ and considering the model catalyst (PMe_3_)Au^+^.^[12]^ We computed the system for (*E*)‐ and (*Z*)‐configured dienynes **9 a**,**b** (Scheme 5). Four possible diastereomers **9 a** (**9 aa** and **9 ab**) and **9 b** (**9 ba** and **9 bb**) were studied. Thus, upon the activation of the alkyne moiety, three conformers of **Int1** were located in all cases, differing on the orientation of the alkene (**9 aa‐Int1 a**–**c**, **9 ab‐Int1 a**–**c**, **9 ba‐Int1 a**–**c**, and **9 bb‐Int1 a**–**c**). In the following mechanistic discussion, **Int1**–**3** refer to structures similar to those depicted in Scheme 2.

**Scheme 6:**
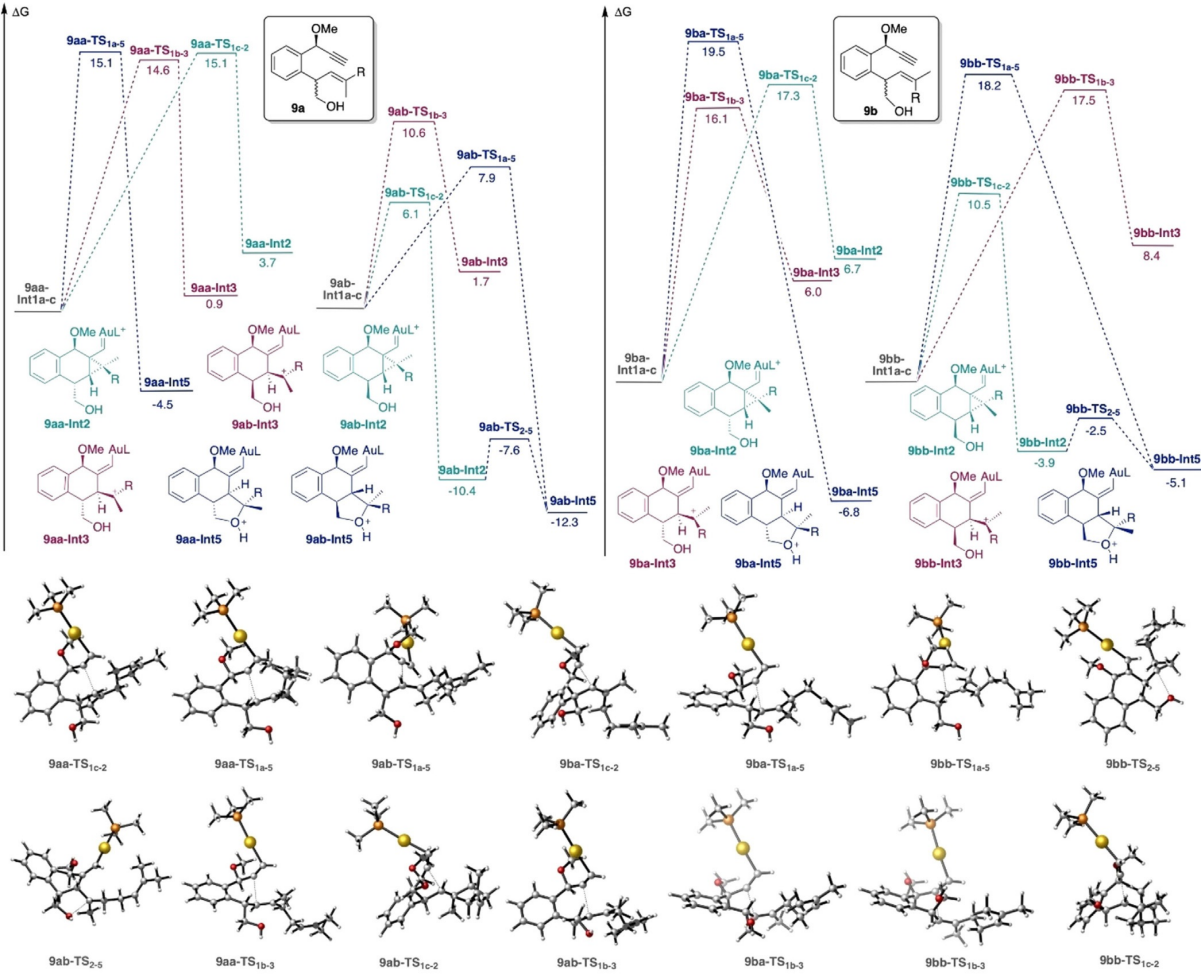
Computed formation of intermediates type of **Int2**, **Int3 and Int5** for enynes **9 a** and **9 b**. L=PMe_3_. Free energies in kcal mol^−1^.

Firstly, we investigated the mixture of diastereomers **9 aa** and **9 ab** (Scheme 6). The six possible conformers of **Int1** for enyne **9 a** (**9 aa‐Int1 a**–**c**, **9 ab‐Int1 a**–**c**) were found to have similar energies (within 0.9 kcal mol^−1^), although different reactivity. For **9 aa**, the three conformers take different pathways generating different products. Transition states were located and energy barriers were found to be also similar. Interestingly, **9 aa‐Int1 a** leads via **9 aa‐TS_1 a‐5_** directly, without the intermediacy of any cyclopropyl gold(I)‐type structure, to the formation of **9 aa‐Int5**. This is a different type of intermediate (**Int5**) not depicted in Scheme 2 and corresponds to a structure similar to **Int3**, but with a fused tetrahydrofuran unit. Intermediates **9 aa‐Int1 b** and **9 aa‐Int1 c** lead to **9 aa‐Int3** and **9 aa‐Int2** respectively. It was also interesting to find that, in this system, open carbocation **9 aa‐Int3** is more stable than closed cyclopropyl gold(I) carbene **9 aa‐Int2** by 2.8 kcal mol^−1^, but 5.4 kcal mol^−1^ less than **9 aa‐Int5**. On the other hand, for **9 ab**, formation of cyclopropyl gold(I)carbene **9 ab‐Int2** is kinetically and thermodynamically more favored than **9 ab‐Int3** by 4.5 and 12.7 kcal mol^−1^, respectively. Intermediate **9 ab‐Int2** gives rise to **9 ab‐Int5** via **9 ab‐TS_2_**_–**5**_, which has a very low energy barrier (2.8 kcal mol^−1^). Furthermore, direct formation of **9 ab‐Int5** was also observed from **9 ab‐Int1 a** through **9 ab‐TS_1 a‐5_**. Therefore, we conclude that for **9 a**, both isomers **9 aa** and **9 ab** give rise to **Int5** type of products (**9 aa‐Int5** and **9 ab‐Int5**) although the reaction is more selective for isomer **9 ab** giving only rise to **9 ab‐Int5**.

**Scheme 7:**
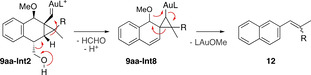
Proposed mechanism for the formation of naphthalene side product **12**.

For those intermediates having the alcohol moiety in *syn*‐position with respect to the cyclopropyl (**9 aa‐Int2**) and to the carbocationic center (**9 ab‐Int3**), closure of the tetrahydrofuran ring was not observed. These intermediates could instead lead to the formation of naphthalene derivative side products **12** by loss of a molecule of formaldehyde in concomitance with a single cleavage rearrangement, connecting these intermediates (**9 aa‐Int2** and **9 ab‐Int3**) with **9 aa‐Int8** (Scheme 7). This mechanistic possibility was not computationally explored, but it is supported by the products observed experimentally.

Similarly, computed pathways for diastereomer **9 ba** have very similar energy barriers leading to **9 ba‐Int5**. However, for **9 bb** the predominant pathway by at least 7 kcal mol^−1^ is the formation of cyclopropyl gold(I) carbene **9 bb‐Int2** via **9 bb‐TS_1 c‐2_**_,_ which would immediately give rise to **9 bb‐Int5** through **9 bb‐TS_2_**_–**5**_. Again, as it happened for **9 aa‐Int2**, the formation of tetrahydrofuran product type **Int5** was not observed from **9 ba‐Int2**. We assumed that this intermediate would also lead to the formation of corresponding (*Z*)‐configured side product **12** (Scheme 7). Hence, **9 bb** would selectively lead to the formation of **9 bb‐Int5**, in good agreement with the experimental results, since no side‐product **12** was obtained for one of the diastereoisomers (presumably **9 bb**, according to the presented calculations).

**Scheme 8:**
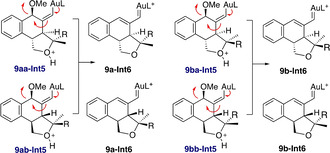
Formation of intermediates **9 a‐Int6** and **9 b‐Int6** for substrates **9 a** and **9 b**.

For both substrates **9 a**,**b** the two pairs **9 aa‐Int5** and **9 ab‐Int5**, and **9 ba‐Int5** and **9 bb‐Int5** would lead to the formation of same intermediates **9 a‐Int6** and **9 b‐Int6** by elimination of the methoxy group (Scheme 8). This explains why, when the reaction was attempted separately (**9 ba** and **9 bb**), both diastereomers delivered the same products, arising from common intermediate **9 b‐Int6**.

**Scheme 9:**
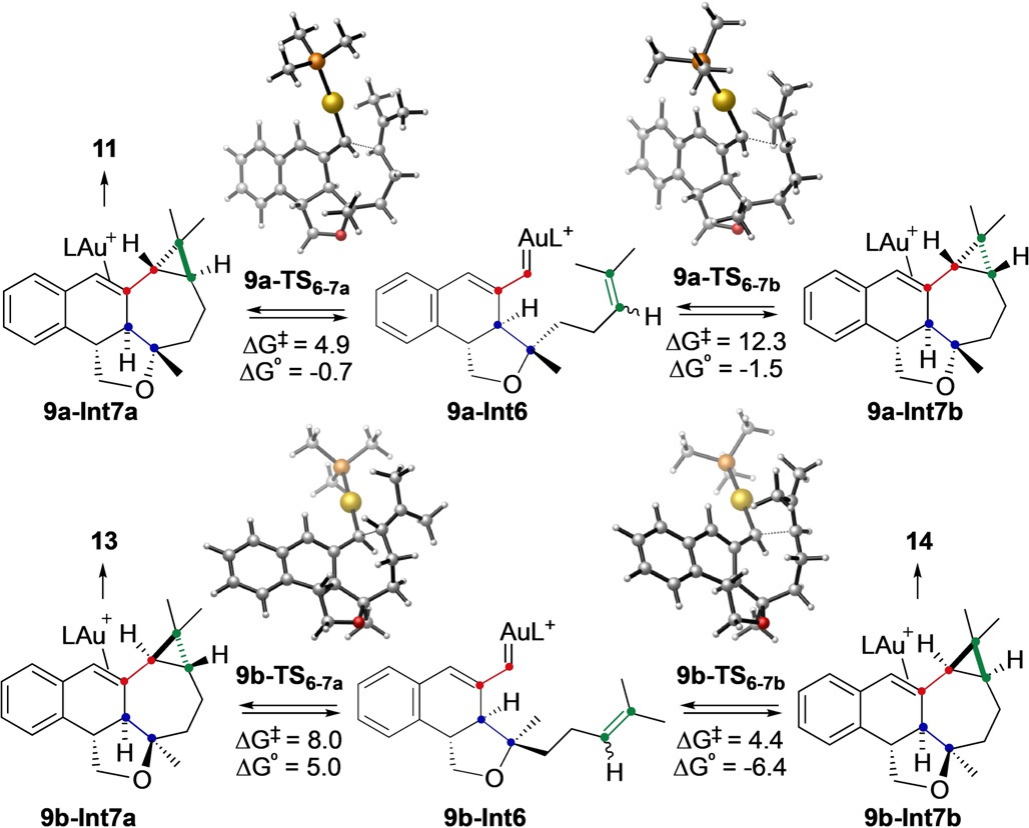
Different mechanistic pathways for cyclopropanation reactions of intermediates **9 a‐Int6** and **9 b‐Int6**. L=PMe_3_. Free energies in kcal mol^−1^.

To understand the selective formation of *trans*‐fused rings **11** and **13** from **9 a**‐**b**, the last cyclopropanation step was computed from intermediates **9 a‐Int6** and **9 b‐Int6** (Scheme 9). For substrate **9 a** with an (*E*)‐configured alkene, intermediate **9 a‐Int6** can react further to form **9 a‐Int7 a** via **9 a‐TS_6_**_–**7 a**_ or **9 a‐Int7 b** through **9 a‐TS_6_**_–**7 b**_, respectively. Remarkably, formation of **9 a‐Int7 a,** which leads to *trans*‐fused cyclopropane **11**, is 7.4 kcal mol^−1^ more favorable than formation of **9 a‐Int7 b**, in agreement with our experimental results. However, for (*Z*)‐alkene **9 b**, the energy difference for the two pathways is lower (3.6 kcal mol^−1^) and a mixture of *cis/trans*‐cyclopropane products could be formed, favoring *cis*‐**14** (from **9 b‐Int7 b**), as observed experimentally.

### Structure of cyclopropyl gold(I) carbene intermediates

In the previous mechanistic study (Scheme 6) we found that the initial cyclization can lead to the formation of cyclopropyl gold carbenes (**Int2**) and open carbocations (**Int3**), such as the pairs **9 aa‐Int2** and **9 aa‐Int3**, with very distinct energies and geometries. Therefore, to rigorously settle this issue computationally and to determine the dependence of the structure of the most stable intermediates on the different substituents, we studied the first step in the cyclization of several 1,6‐enynes.

**Scheme 10:**
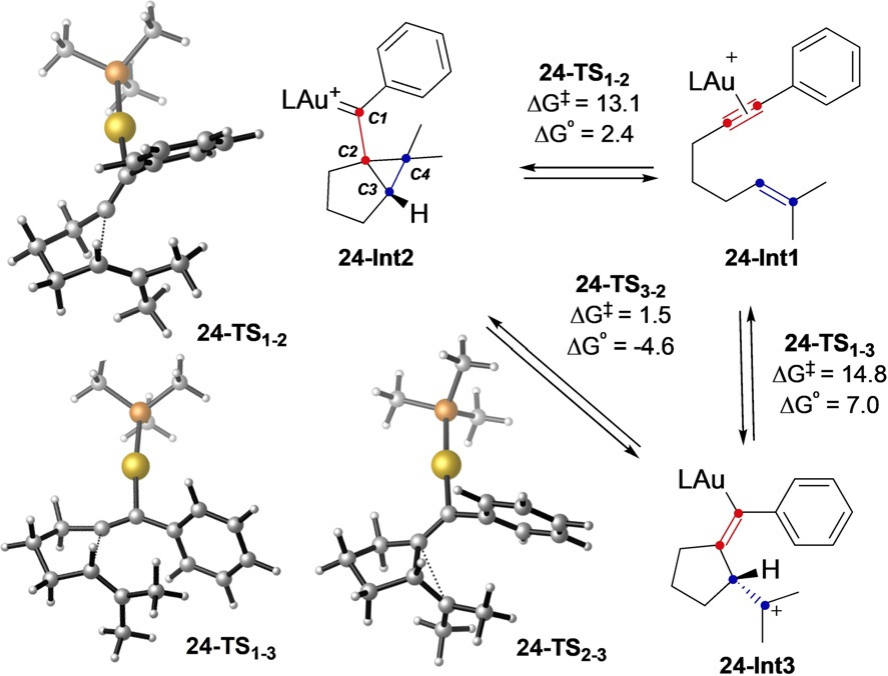
Formation of cyclopropyl gold(I) carbene **24‐Int2** and open carbocation **24‐Int3** from **24‐Int1**, L=PMe_3_. Free energies in kcal mol^−1^.

First, we studied the behavior of 1‐phenyl‐7‐methyloct‐6‐en‐1‐yne (**24**) in the gold(I)‐catalyzed cyclization (Scheme 10). Initially, we performed a benchmark of DFT functionals using DLPNO‐CCSD(T)[^18^, ^19^] as calibration method in order to discard any artifact associated to the chosen DFT method.^[12]^

We found that the most appropriate functionals for this system are M06^[20]^‐D3^[14]^ and B3LYP^[13]^‐D3.^[14]^ Hence, in this section, M06‐D3/6‐31G(d)^[15]^ (C, H, P), SDD^[16]^ (Au) level in CH_2_Cl_2_ (PCM)^[17]^ level of theory was used to investigate the effect of substituents on the formation of different carbene‐type intermediates. Both cyclopropyl gold(I) carbene **24‐Int2** and open carbocation **24‐Int3** could be accessed from **24‐Int1**, although the former intermediate was favored by 1.7 kcal mol^−1^. Interestingly, **24‐Int2** and **24‐Int3** are in equilibrium through **24‐TS_2_**_–**3**_. These intermediates show very different angles and bond lengths.^[12]^ Thus, the C2‐C3‐C4 angle is 69.6° for **24‐Int2** and 115.9° for **24‐Int3**. The C2−C4 distance in **24‐Int2** (1.734 Å) is significantly shorter than that for open carbocation **24‐Int3** (2.584 Å), whereas the C1−C2 distance shows the opposite trend (1.417 Å for **24‐Int2** vs. 1.351 Å fo**r 24‐Int3**).

**Scheme 11:**
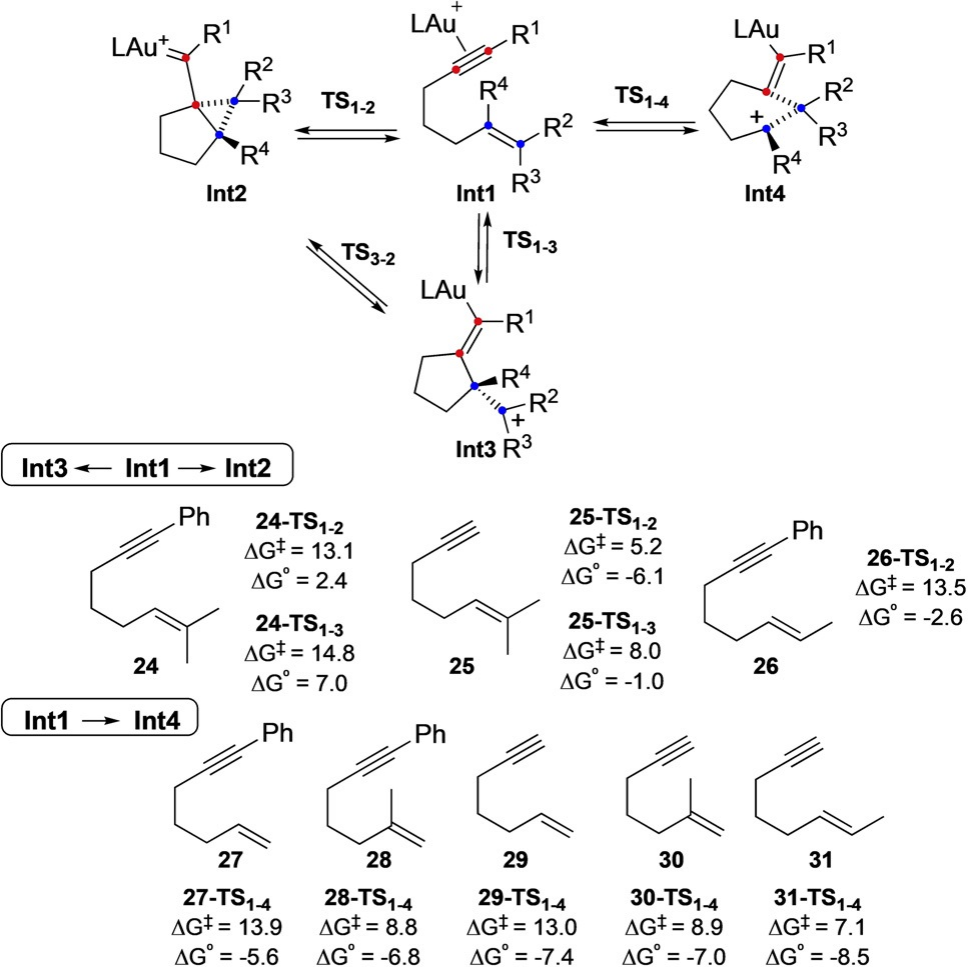
Possible pathways for the formation of carbene **Int2**, open carbocation **Int3** and semi‐opened **Int4**. L=PMe_3_. Free energies in kcal mol^−1^.

We decided to investigate the effect of substituents on the initial formation of carbenic (**Int2**) or carbocationic (**Int3** and/or **Int4**) intermediates with different enynes **24**–**31** (Scheme 11). In general, the three different intermediates **Int2**–**4** were located having different angles and distances: cyclopropyl gold(I) carbene (**Int2**), open carbocation (**Int3**) and semi‐opened system (**Int4**) with a positive charge delocalized at C3 from enyne **24** that has a disubstituted alkene moiety. For 1,6‐enynes **24**–**26** with at least one methyl substituent at the terminal carbon of the alkene or a phenyl group at the alkyne, both **Int2** and **Int3** intermediates are formed, although in all cases the former is kinetically and thermodynamically favored.^[21]^ On the other hand, for enynes **27**–**31** bearing terminal alkyne or alkene, formation of **Int4** was observed. Similar results were observed when computing systems that contain aryl tethered 1,6‐enynes or alkyl tethered 1,7‐enynes.^[12]^

**Figure 3:**
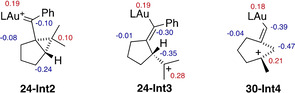
NPA charges by NBO analysis. Positive charges in red and negative charges in blue.

The carbenic or carbocationic character of intermediates **Int2**, **Int3**, and **Int4** was confirmed by a natural bond orbital (NBO) population analysis on representative structures (Figure 3).[^12^, ^22^] The Au NPA charge was 0.193 for **24‐Int2**, 0.189 for **24‐Int3** and 0.185 for **30‐Int4**, being slightly higher for cyclopropyl gold(I) carbene species of type **Int2**, as expected. Moreover, NPA charges of the carbons connected to the gold atom are also slightly different for each system. For **24‐Int3** and **30‐Int4**, positive charges are clearly delocalized and, for that reason charges on the carbon‐carbon double bond are more negative than for **24‐Int2**.

**Scheme 12:**
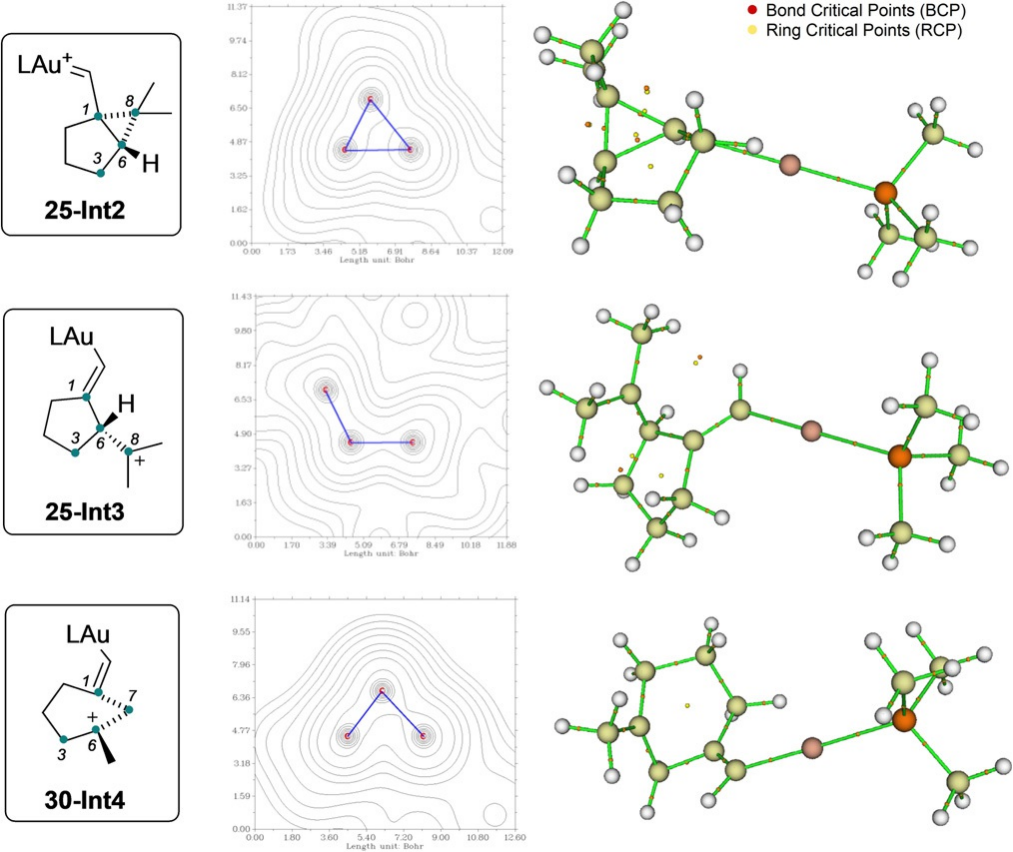
Molecular structure of **25‐Int2**, **25‐Int3** and **30‐Int4**. Laplacian map with electron density contour of atoms 1–6–8 and 1–6–7, bonds depicted in blue. QTAIM Topological Analysis graph with (BCPs) in red and (RCPs) in yellow.

To further prove the structures of **25‐Int2**, **25‐Int3**, **30‐Int4**, we carried out Quantum Theory Atoms in Molecules (QTAIM)[^12^, ^23^] (Scheme 12). The bond critical points (BCPs) and the ring critical points (RCPs) were located and analyzed using Laplacian maps. Two RCP were located for intermediate **25‐Int2**, whereas no RCP was observed between C8−C1 in the case of **25‐Int3**, and the Laplacian clearly indicates the absence of this bond. Finally, only one ring critical point was observed in **30‐Int4** as a consequence of its semi‐opened ring system. Hence, QTAIM theory confirms that the molecular representation of these intermediates is accurate.

A mechanistic study on indium‐catalyzed cycloisomerizations of 1,6‐enynes found that the key intermediates for substrates of type **29** are also of type **Int4**.^[24]^ For the sake of completeness, we also studied the possible involvement of intermediates related to **Int2** and **Int3** starting from enynes **24** and **30** using InCl_3_. We chose M06‐D3/6‐31G(d) (C, H) and LANL2DZ (In, Cl) level of theory in CH_2_Cl_2_ (PCM). We observed the formation of both intermediates **24‐In‐Int2** and **24‐In‐Int3** for enyne **24**, whereas in the case of **30**, only **30‐In‐Int4** was found (Table 1). Interestingly, **24‐In‐Int3** is the most favored intermediate species when using indium catalyst, whereas in the case of gold(I), cyclopropyl gold carbene **24‐Int2** is the preferred intermediate. Slight structural differences were observed when comparing these intermediates (**24‐In‐Int2** and **24‐In‐Int3**) with the ones found previously with gold(I) (**24‐Int2**, **24‐Int3**). In the case of cyclopropyl carbene **24‐In‐Int2**, longer distances were observed between C2−C4, indicating a less strained cyclopropane ring than for the gold(I) intermediate (Table 1, entry 2). In addition, the C2‐C3‐C4 angle was larger (72°) than for the gold(I)‐carbene **24‐Int2** (66.5°) (Table 1, entries 1–2). Intermediate **24‐In‐Int3** was found to be very similar to **24‐Int3** (Table 1, entries 3–4). On the other hand, for **30‐Int4** we observed the formation of a more opened system (longer distances between C2–C3), which presents the smallest angle observed in this study (Table 1, entries 5–6).

**Table 1:** Optimized geometries and comparative list of calculated bond distances and angles of intermediates.^[a]^

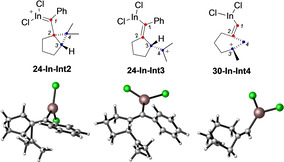					
entry	species	*d*(C2‐C3)	*d*(C2‐C4)	∡(C2‐C3‐C4)	Int2‐Int3^[b]^
1	**24‐Int2**	1.536	1.652	66.5	0.0
2	**24‐In‐Int2**	1.542	1.768	72.0	0.0
3	**24‐Int3**	1.555	2.549	115.4	7.6
4	**24‐In‐Int3**	1.538	2.548	115.9	−4.4
5	**30‐Int4**	1.758	1.561	57.4	–
6	**30‐In‐Int4**	2.213	1.566	44.9	–

[a] DFT calculations performed using InCl_3_ as catalyst or L=PMe_3_ for Au. Free energies in kcal mol^−1^. Distances expressed in Å and angles in degrees. [b] Differences between free energies of (**Int2**, **Int3**) and (**In‐Int2**, **In‐Int3**).

## Conclusions

In summary, we have uncovered a new gold(I)‐catalyzed cyclization cascade of substituted dienynes that can lead to selective formation of unexpected *trans*‐fused cyclopropanes within a *trans*‐bicyclo[5.1.0]octane framework, depending on the substrate geometry. DFT calculations and control experiments show that this specific selectivity is directed by the rigidity of the system. Likewise, computed pathways provide a rational for the role played by the fused tetrahydrofuran ring in the final cyclopropanation step. These new results expand the scope of these type of gold(I)‐catalyzed cyclizations for the formation of highly complex carbocyclic skeletons, in this case bearing *trans*‐fused cyclopropanes.

Our computational study included a reevaluation of the nature of the key intermediates in cycloisomerizations of enynes, providing evidence on the existence of three different types of cationic intermediates depending on the substitution of the initial substrate. The QTAIM theory confirms that the molecular representation of the different types of intermediates is accurate. Moreover, the metal carbenic or cationic character of these intermediates was confirmed by NBO analysis.

## Experimental

Full details of all synthesis, characterization and DFT calculations can be found in the Supporting Information.

Deposition Numbers 1906609 (**11**), 1906610 (**17 a**), 1906613 (**17 b**), 1906611 (**18**), 1906612 (**21**), and 1906608 (**23**) contain the supplementary crystallographic data for this paper. These data are provided free of charge by the joint Cambridge Crystallographic Data Centre and Fachinformationszentrum Karlsruhe Access Structures service www.ccdc.cam.ac.uk/structures.

## Conflict of interest

The authors declare no conflict of interest.

## Supporting information

As a service to our authors and readers, this journal provides supporting information supplied by the authors. Such materials are peer reviewed and may be re‐organized for online delivery, but are not copy‐edited or typeset. Technical support issues arising from supporting information (other than missing files) should be addressed to the authors.

Supplementary
