# Supplementary material for: On the Structure of Intermediates in Enyne Gold(I)‐Catalyzed Cyclizations: Formation of trans‐Fused Bicyclo[5.1.0]octanes as a Case Study
Source: Chemistry. 2020 Nov 19;26(67):15738–45. doi: 10.1002/chem.202004237 (PMC7756441; doi:10.1002/chem.202004237)
Supplement: Supplementary file 1 — Supplementary [file CHEM-26-15738-s001.pdf]

Chemistry–A European Journal

Supporting Information

On the Structure of Intermediates in Enyne Gold(I)-Catalyzed Cyclizations: Formation of *trans*-Fused Bicyclo[5.1.0]octanes as a Case Study

Imma Escofet,[a, b] Helena Armengol-Relats,[a, b] Hanna Bruss,[a, b] Maria Besora,[c] and Antonio M. Echavarren*[a, b]

Table of Contents

1. Experimental part ... 3

1.1 General experimental methods ... 3

1.2 Synthesis of the cyclization precursors ... 4

1.2.1 Synthesis of cyclization substrates 15-17 ... 4

1.2.2 Synthesis of simpler substrates 15-16 ... 16

1.3 General procedure for gold(I)-catalyzed cyclization: ... 22

1.3.1 Characterization of cyclization products 5a, 11-14, 17-18, 20 and 22 ... 22

1.3.2 General procedure for TBS-deprotection of cyclization products 20 and 22 ... 26

1.4 Control experiments ... 28

1.4.1 Parallel reactions with diastereomerically pure 10b ... 28

1.4.2 Synthesis and reactivity of 7,12-dien-1-enyes ... 30

2. Theoretical DFT Computations ... 34

2.1 Computational methods ... 34

2.2 Results ... 35

2.3 Coordinates and Energies of Relevant Intermediates and Transition States (B3LYP, and M06, L=PMe_3) ... 41

3. References ... 85

4. NMRS ... 86

5. X-ray data ... 144

1. Experimental part

1.1 General experimental methods

Unless otherwise specified solvents were collected from a PureSolv™ solvent purification system (SPS, Innovative Technologies, Inc., MA) and reactions were run under argon atmosphere. Commercially available reagents were used without further purification. Organic solutions were concentrated under reduced pressure on a Büchi rotary evaporator. Thin layer chromatographies were carried out on TLC aluminium sheets coated with 0.2 mm of silica gel with fluorescent indicator F254 (Merck). UV light (254 nm) was used as visualizing agent and an acidic solution of vanillin in ethanol was used as stain. GCMS analyses were performed on an Agilent Technologies apparatus equipped with gas chromatograph GC 7890B, column HP-5MS and mass spectrometer MSD 5977A (electron ionization ion source and single quadrupole analyzer).

Column chromatography purifications were performed on flash grade silica gel (Panreac AppliChem silica gel 60, 40-63 μm) using pure grade solvents. Preparative TLC were performed on glass TLC plates (20 x 20 cm) loaded with 2000 μm of silica gel (Silica Gel GF, Analtech).

The two diastereomers of **10b** were separated by collection of the fractions from chiral HPLC: column: Chiralpak IC 250x4.6mm, 5μm, method: Hex / DCM / IPA 84:15:1, flow: 1mL/min, sample: 1 mg/mL in mobile phase.

NMR spectroscopy experiments were performed at 25 ºC in a Bruker Avance 300, Bruker Avance 400 Ultrashield or Bruker Avance 500 Ultrashield apparatuses. The signals are given as δ / ppm (multiplicity, coupling constant (Hertz), number of protons) downfield from tetramethylsilane, with calibration against the residual protio-solvent used (δ_H = 7.26 ppm and δ_C = 77.16 ppm for $CDCl_3$, δ_H = 5.32 ppm and δ_C = 53.84 ppm for CD_2Cl_2). ^{13}C NMR always refers to ^{13}C NMR decoupled of ^{1}H. Mass spectra (ESI, APCI) were recorded on a Waters LCT Premier HPLC/MS-TOF or a Waters Alliance HPLC coupled MicroTOF Focus (Bruker Daltonics) apparatuses.

Crystal structure determinations were carried out using a Bruker-Nonius diffractomer equipped with an APEX II 4K CCD area detector, a FR591 rotating anode with Mo K_α radiation, Montel mirrors as monochromator and a Kryoflex low temperature device (T = –173 ºC). Full-sphere data collection was used with w and j scans. *Programs used*: Data collection APEX-2, data reduction Bruker Saint V/.60A and absorption correction SADABS. Structure Solution and Refinement: crystal structure solutions were achieved using direct methods as implemented in SHELXTL and visualized using the program XP. Missing atoms were subsequently located from difference Fourier synthesis and added to the atom list. Least-squares refinement on F2 using all measured intensities was carried out using the program SHELXTL. All non-hydrogen atoms were refined including anisotropic displacement parameters. Crystallographic data (excluding structure factors) for the structures in this paper have been deposited with the Cambridge Crystallographic Data Centre as supplementary publication nos. CCDC 1906608-1906613. Copies of the data can be obtained, free of charge, on application to CCDC, 12 Union Road, Cambridge CB2 1EZ, UK, (fax: +44-(0)1223-336033 or e-mail: deposit@ccdc.cam.ac.uk).

1.2 Synthesis of the cyclization precursors

1.2.1 Synthesis of cyclization substrates 15-17

(*E*)-((1-bromo-3,7-dimethylocta-1,6-dien-3-yl)oxy)triethylsilane (S1)

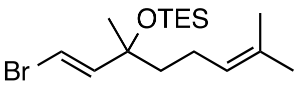

To a suspension of $Cp_2Zr(H)Cl$ (4.15 g, 16.1 mmol, 1.2 equiv) in CH_2Cl_2 (100 mL) was added a solution of alkyne[1] (3.57 g, 13.4 mmol, 1 equiv) in CH_2Cl_2 (15 mL). The resulting mixture was stirred at 23 ºC for 1 h protected from light. Afterwards NBS (2.86 g, 16.1 mmol, 1.2 equiv) was added in one portion and stirring was continued for 1 h. The reaction was quenched by addition of $H_2O/NaHCO_3/Na_2SO_3$ (1:1:1) and the mixture was stirred for 30 min before the layers were separated. The aqueous phase was extracted with CH_2Cl_2 (2 x) and the combined organic layers were washed with brine, dried ($MgSO_4$) and concentrated under reduced pressure. The residue was taken up in heptane, passed through a plug of basic alumina oxide and evaporated in vacuo. Purification by flash column chromatography on silica with heptane provided **S1** (3.61 g, 10.4 mmol, 78%).
^{1}H NMR (400 MHz, $CDCl_3$) δ = 6.21 (d, *J* = 15.7 Hz, 1H), 6.18 (d, *J* = 15.8 Hz, 1H), 5.06 (tp, *J* = 7.2, 1.4 Hz, 1H), 2.08 – 1.90 (m, 2H), 1.68 (d, *J* = 1.2 Hz, 3H), 1.59 (s, 3H), 1.55 – 1.43 (m, 2H), 1.31 (s, 3H), 0.95 (t, *J* = 7.9 Hz, 9H), 0.59 (q, *J* = 8.0 Hz, 6H) ppm.
^{13}C NMR (126 MHz, $CDCl_3$) δ = 144.7, 131.7, 124.4, 104.9, 76.6, 43.8, 27.8, 25.8, 22.9, 17.8, 7.2 (3C), 6.8 (3C) ppm.
HRMS (APCI) *m/z* calc. for $C_{10}H_{16}BrO^+$ $[M\text{-}SiEt_3]^+$: 231.0379, found: 231.0378.

(*E*)-2-(3,7-dimethyl-3-((triethylsilyl)oxy)octa-1,6-dien-1-yl)benzonitrile (S2)

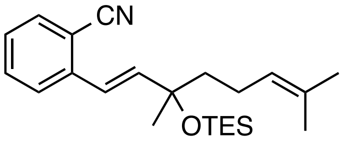

A stirred solution of **S1** (2.86 g, 8.22 mmol, 1 equiv), 2-cyanophenylboronic acid (1.45 g, 9.87 mmol, 1.2 equiv), $Pd(PPh_3)_4$ (950 mg, 0.82 mmol, 0.1 equiv) and Cs_2CO_3 (4.56 mg, 12.3 mmol, 1.5 equiv) in THF (85 mL) was heated to 80 ºC for 2 h. After cooling to 23 ºC and filtration through Celite with Et_2O water was added. The layers were separated, and the aqueous phase was extracted with Et_2O (2 x). The combined organic layers were washed with brine, dried ($MgSO_4$) and concentrated under reduced pressure. Purification by column chromatography on silica with cyclohexane/EtOAc 50:1 as eluent gave **S2** (1.67 g, 4.52 mmol, 55%).
^{1}H NMR (500 MHz, CD_2Cl_2) δ = 7.64 – 7.60 (m, 2H), 7.56 – 7.52 (m, 1H), 7.31 (td, *J* = 7.6, 1.2 Hz, 1H), 6.91 (d, *J* = 15.8 Hz, 1H), 6.44 (d, *J* = 15.8 Hz, 1H), 5.11 (tp, *J* = 7.2, 1.4 Hz, 1H), 2.15 – 1.97 (m, 2H), 1.66 (d, *J* = 1.4 Hz, 3H), 1.65 – 1.59 (m, 2H), 1.58 (s, 3H), 1.45 (s, 3H), 1.00 (t, *J* = 7.9 Hz, 9H), 0.66 (q, *J* = 8.1 Hz, 6H) ppm.
^{13}C NMR (126 MHz, CD_2Cl_2) δ = 143.3, 141.3, 133.5, 133.2, 131.8, 127.8, 126.1, 125.1, 123.6, 118.4, 111.7, 76.1, 44.5, 28.4, 26.0, 23.5, 17.9, 7.5 (3C), 7.3 (3C) ppm.
HRMS (ESI) *m/z* calc. for $C_{23}H_{35}NOSiNa^+$ $[M+Na]^+$: 392.2380, found: 392.2374.

(*E*)-2-(3-hydroxy-3,7-dimethylocta-1,6-dien-1-yl)benzonitrile (S3)

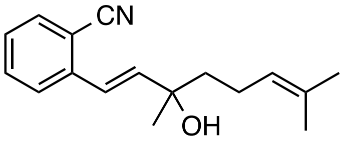

To a solution of **S2** (2.21 g, 5.98 mmol, 1 equiv) in THF (75 mL) was added TBAF (1 M in THF, 7.2 mL, 7.17 mmol, 1.2 equiv) at 0 ºC. The mixture was slowly warmed to 23 ºC over 1 h. After addition of aqueous saturated NH_4Cl solution, the two layers were separated and the aqueous phase was extracted with EtOAc. The combined organic layers were washed with brine, dried ($MgSO_4$) and concentrated under reduced pressure. Purification by flash chromatography on silica with cyclohexane/EtOAc (9:1) yielded **S3** (1.45 g, 5.67 mmol, 95%).
^{1}H NMR (400 MHz, $CDCl_3$) δ = 7.64 – 7.58 (m, 2H), 7.56 – 7.50 (m, 1H), 7.30 (td, *J* = 7.5, 1.3 Hz, 1H), 6.94 (d, *J* = 16.0 Hz, 1H), 6.49 (d, *J* = 16.0 Hz, 1H), 5.14 (ddp, *J* = 7.2, 5.7, 1.4 Hz, 1H), 2.19 – 2.01 (m, 2H), 1.83 (s, 1H), 1.74 – 1.69 (m, 2H), 1.68 (d, *J* = 1.3 Hz, 3H), 1.61 (s, 3H), 1.41 (s, 3H) ppm.
^{13}C NMR (126 MHz, $CDCl_3$) δ = 142.4, 140.6, 133.2, 132.8, 132.5, 127.5, 126.1, 124.2, 123.4, 118.1, 111.1, 73.8, 42.4, 28.4, 25.9, 23.1, 17.9 ppm.
HRMS (ESI) *m/z* calc. for $C_{17}H_{21}NONa^+$ $[M+Na]^+$: 278.1515, found: 278.1521.

(*E*)-2-(3,7-dimethyl-3-((trimethylstannyl)methoxy)octa-1,6-dien-1-yl)benzonitrile (S4)

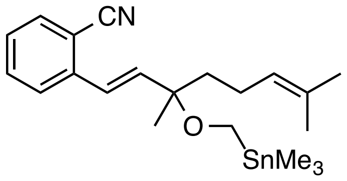

To a solution of potassium hydride (50% in parrafin, 524 mg, 6.54 mmol, 1.2 equiv) and 18-crown-6 (1.73 g, 6.54 mmol, 1.2 equiv) in THF (100 mL) was added a solution of **S3** (1.39 g, 5.45 mmol, 1 equiv) in THF (15 mL) followed by a solution of ICH_2SnMe_3 (8.30 g, 27.2 mmol, 5 quiv) in THF (15 mL). The reaction mixture was stirred at 23 ºC for 2.5 h and then quenched with saturated aqueous NH_4Cl-solution. The aqueous phase was extracted with EtOAc (2 x) and the combined organic layers were washed with brine, dried ($MgSO_4$) and concentrated under reduced pressure. Purification by flash chromatography on silica with cyclohexane/EtOAc (30:1) as eluent gave **S4** (2.13 g, 4.94 mmol, 91%).
^{1}H NMR (400 MHz, CD_2Cl_2) δ = 7.69 – 7.59 (m, 2H), 7.55 (tdd, *J* = 8.0, 1.5, 0.6 Hz, 1H), 7.32 (td, *J* = 7.6, 1.3 Hz, 1H), 6.82 (d, *J* = 16.2 Hz, 1H), 6.36 (d, *J* = 16.2 Hz, 1H), 5.13 (tp, *J* = 7.2, 1.4 Hz, 1H), 3.51 (d, *J* = 4.7 Hz, 2H), 2.05 – 1.96 (m, 2H), 1.73 – 1.67 (m, 1H), 1.67 (d, *J* = 1.3 Hz, 3H), 1.65 – 1.61 (m, 1H), 1.60 (s, 3H), 1.34 (s, 3H), 0.14 (s, 9H) ppm.
^{13}C NMR (101 MHz, CD_2Cl_2) δ = 141.8, 141.3, 133.5, 133.2, 131.9, 127.9, 126.2, 125.6, 125.1, 118.4, 111.6, 78.9, 52.9, 39.9, 26.0, 23.0, 22.5, 17.9, -10.4 (3C) ppm.
HRMS (ESI) *m/z* calc. for $C_{21}H_{31}NO^{120}SnNa$ $[M+Na]^+$: 456.1323, found: 456.1320.

2-(1-hydroxy-4,8-dimethylnona-3,7-dien-2-yl)benzonitrile (S5)

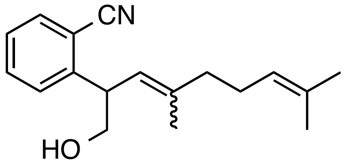

To a solution of **S4** (1.05 g, 2.43 mmol, 1 equiv) and 18-crown-6 (963 mg, 3.64 mmol, 1.5 equiv) in THF (40 mL) was added *n*BuLi (2.5 M, 1.2 mL, 2.92 mmol, 1.2 equiv) at -90 ºC. After stirring for 1 h NH_4Cl-solution was added and the layers were separated. The aqueous phase was with EtOAc (2 x) and the combined organic layers were washed with brine, dried ($MgSO_4$) and concentrated under reduced pressure. Purification by flash chromatography on silica with cyclohexane/EtOAc (7:1) as eluent gave **S5** (338 mg, 1.25 mmol, 52%) as a 1.5:1 mixture of isomers.

^{1}H NMR (500 MHz, $CDCl_3$) δ = 7.66 – 7.60 (m, 1H *E*-isomer, 1H *Z*-isomer), 7.55 (td, *J* = 7.7, 1.5 Hz, 1H *E*-isomer, 1H *Z*-isomer), 7.41 (dd, *J* = 8.0, 1.1 Hz, 1H *Z*-isomer), 7.40 (dd, *J* = 8.0, 1.1 Hz, 1H *E*-isomer), 7.31 (td, *J* = 7.6, 1.2 Hz, 1H *E*-isomer), 7.30 (td, *J* = 7.6, 1.2 Hz, 1H *Z*-isomer), 5.41 (dd, *J* = 9.4, 1.6 Hz, 1H *Z*-isomer), 5.32 (dq, *J* = 9.1, 1.3 Hz, 1H *E*-isomer), 5.06 – 5.02 (m, 1H *E*-isomer, 1H *Z*-isomer), 4.26 – 4.16 (m, 1H *E*-isomer, 1H *Z*-isomer), 3.86 – 3.73 (m, 2H *E*-isomer, 2H *Z*-isomer), 2.19 – 1.98 (m, 4H *E*-isomer, 4H *Z*-isomer), 1.77 (d, *J* = 1.4 Hz, 3H, *Z*-isomer), 1.72 (d, *J* = 1.4 Hz, 3H, *E*-isomer), 1.66 (s, 3H *E*-isomer), 1.60 (s, 3H *Z*-isomer), 1.57 (s, 3H *E*-isomer), 1.55 (s, 3H *Z*-isomer) ppm.

^{13}C NMR (126 MHz, $CDCl_3$) δ = 146.9, 146.8, 141.0, 140.9, 133.3, 133.2, 133.1, 133.1, 132.3, 132.1, 128.3, 128.0, 127.0, 127.0, 124.0, 123.8, 123.1, 122.7, 118.3, 118.3, 112.9, 112.7, 67.0, 66.6, 45.5, 45.3, 39.9, 32.7, 26.5, 26.4, 25.8, 25.8, 23.7, 17.8, 17.8, 17.0 ppm.

HRMS (ESI) *m/z* calc. for $C_{18}H_{23}NONa$ $[M+Na]^+$: 292.1672, found: 292.1675.

2-(1-((*tert*-butyldimethylsilyl)oxy)-4,8-dimethylnona-3,7-dien-2-yl)benzonitrile (S6)

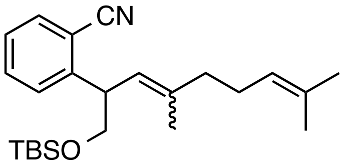

To a solution of **S5** (950 mg, 3.53 mmol, 1 equiv) in DMF (20 mL) was added imidazole (360 mg, 5.29 mmol, 1.5 equiv) and TBSCl (691 mg, 4.58 mmol, 1.3 equiv). The reaction mixture was stirred at 23 ºC overnight and then quenched with saturated NH_4Cl solution. The aqueous phase was with EtOAc (2 x) and the combined organic layers were washed with brine, dried ($MgSO_4$) and concentrated under reduced pressure. Purification by flash chromatography on silica with cyclohexane/EtOAc (50:1) as eluent gave **S6** (1.06 mg, 2.76 mmol, 78%) as a 1:1.5 inseparable mixture of double bond isomers.

^{1}H NMR (500 MHz, $CDCl_3$) δ = 7.59 (dd, *J* = 7.7, 1.5 Hz, 1H *E*-isomer), 7.58 (dd, *J* = 7.6, 1.3 Hz, 1H *Z*-isomer), 7.50 (td, *J* = 7.7, 1.4 Hz, 1H *E*-isomer, 1H *Z*-isomer), 7.40 (dd, *J* = 8.0, 1.3 Hz, 1H *Z*-isomer), 7.39 (dd, *J* = 7.9, 1.3 Hz, 1H *E*-isomer), 7.29 – 7.23 (m, 1H *E*-isomer, 1H *Z*-isomer), 5.44 (d, *J* = 9.4 Hz, 1H *Z*-isomer), 5.39 (dq, *J* = 8.8, 1.3 Hz, 1H *E*-isomer), 5.08 – 5.00 (m, 1H *E*-isomer, 1H *Z*-isomer), 4.20 – 4.10 (m, 1H *E*-isomer, 1H *Z*-isomer), 3.82 – 3.72 (m, 2H *E*-isomer, 2H *Z*-isomer), 2.13 – 1.92 (m, 4H *E*-isomer, 4H *Z*-isomer), 1.74 (d, *J* = 1.4 Hz, 3H *Z*-

isomer), 1.66 (d, J = 1.3 Hz, 3H E-isomer), 1.65 (d, J = 1.4 Hz, 3H E-isomer), 1.60 (d, J = 1.4 Hz, 3H Z-isomer), 1.57 (s, 3H E-isomer), 1.54 (s, 3H Z-isomer), 0.79 (s, 9H E-isomer), 0.78 (s, 9H Z-isomer), -0.06 (s, 3H E-isomer), -0.08 (s, 3H Z-isomer), -0.09 (s, 3H E-isomer), -0.11 (s, 3H Z-isomer) ppm.
^{13}C NMR (101 MHz, $CDCl_3$) δ = 148.2, 148.1, 139.3, 139.2, 132.8, 132.7, 132.6, 132.5, 132.0, 131.7, 128.8, 128.7, 126.5 (2C), 124.2, 124.0, 123.4, 123.0, 118.6, 118.5, 113.0, 112.9, 67.4, 67.1, 45.4, 45.2, 39.8, 32.7, 26.6, 26.5, 25.9 (6C), 25.8, 25.8, 23.6, 18.3 (2C), 17.8, 17.7, 17.0, -5.4, -5.4, -5.5, -5.6 ppm.
HRMS (ESI) *m/z* calc. for $C_{24}H_{37}NNaOSi$ $[M+Na]^+$: 406.2537, found: 406.2539.

2-(1-((*tert*-butyldimethylsilyl)oxy)-4,8-dimethylnona-3,7-dien-2-yl)benzaldehyde (S7a-b)

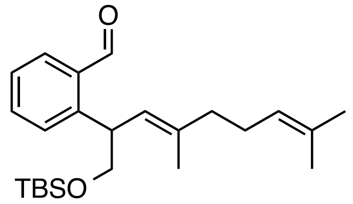

To a solution of **S6** (295 mg, 0.77 mmol, 1 equiv) in toluene (10 mL) was added DIBAL-H (1 M in toluene, 1.2 mL, 1.15 mmol, 1.5 equiv) at 0 ºC. Stirring at 0 ºC was continued for 2 h followed by the addition of 0.5 M HCl. The aqueous phase was extracted with EtOAc (3 x) and the combined organic layers were washed with saturated NH_4Cl-solution and brine, dried ($MgSO_4$) and concentrated under reduced pressure. Purification by flash chromatography on silica with cyclohexane/EtOAc (50:1) as eluent gave **S7** (249 mg, 0.64 mmol, 84%) as a mixture of double bond isomers. At this stage the two double bond isomers were separated by preparative TLC using pentane/Et_2O 50:1 as eluent.

S7a:
^{1}H NMR (400 MHz, $CDCl_3$) δ = 10.42 (s, 1H), 7.83 (dd, J = 7.7, 1.5 Hz, 1H), 7.51 (td, J = 7.5, 1.5 Hz, 1H), 7.42 (dd, J = 7.9, 1.3 Hz, 1H), 7.33 (td, J = 7.4, 1.3 Hz, 1H), 5.45 (dq, J = 8.5, 1.3 Hz, 1H), 5.12 – 5.02 (m, 1H), 4.70 (td, J = 7.9, 5.8 Hz, 1H), 3.80 (dd, J = 9.7, 5.8 Hz, 1H), 3.72 (dd, J = 9.7, 7.4 Hz, 1H), 2.13 – 1.99 (m, 4H), 1.67 (s, 3H), 1.57 (s, 6H), 0.77 (s, 9H), -0.12 (s, 3H), -0.13 (s, 3H) ppm.
^{13}C NMR (101 MHz, $CDCl_3$) δ = 192.6, 146.8, 138.3, 134.5, 133.7, 131.7, 130.2, 128.9, 126.4, 124.3, 124.2, 68.0, 40.7, 39.8, 26.6, 25.9 (3C), 25.8, 18.4, 17.8, 17.0, -5.5, -5.5 ppm.
HRMS (ESI) *m/z* calc. for $C_{24}H_{38}O_2SiNa$ $[M+Na]^+$: 409.2533, found: 409.2534.

S7b:

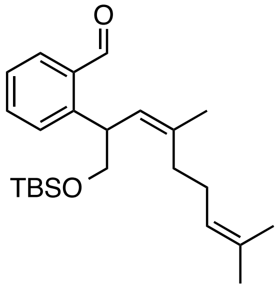

^{1}H NMR (400 MHz, $CDCl_3$) δ = 10.42 (s, 1H), 7.83 (dd, *J* = 7.9, 1.4 Hz, 1H), 7.51 (td, *J* = 7.6, 1.6 Hz, 1H), 7.43 (dd, *J* = 7.9, 1.3 Hz, 1H), 7.35 (td, *J* = 7.3, 1.1 Hz, 1H), 5.50 (dd, *J* = 8.9, 1.5 Hz, 1H), 5.01 – 4.91 (m, 1H), 4.75 (td, *J* = 8.2, 5.5 Hz, 1H), 3.79 (dd, *J* = 9.6, 5.5 Hz, 1H), 3.69 (dd, *J* = 9.6, 7.6 Hz, 1H), 2.07 – 1.89 (m, 4H), 1.75 (d, *J* = 0.9 Hz, 3H), 1.58 (s, 3H), 1.50 (s, 3H), 0.76 (s, 9H), -0.15 (s, 3H), -0.16 (s, 3H) ppm.
^{13}C NMR (101 MHz, $CDCl_3$) δ = 192.4, 147.1, 138.5, 134.4, 133.7, 131.9, 130.1, 129.0, 126.4, 124.6, 124.0, 68.4, 40.2, 32.8, 26.3, 25.9 (3C), 25.7, 23.6, 18.4, 17.7, -5.5, -5.5 ppm.
HRMS (ESI) *m/z* calc. for $C_{24}H_{38}O_2SiNa$ $[M+Na]^+$: 409.2533, found: 409.2534.

(*E*)-1-(2-(1-((*tert*-butyldimethylsilyl)oxy)-4,8-dimethylnona-3,7-dien-2-yl)phenyl)prop-2-yn-1-ol (S8a)

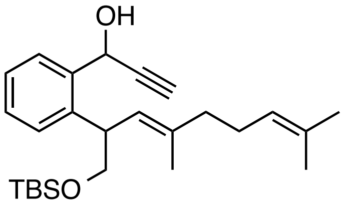

To a solution of **S7a** (224 mg, 0.58 mmol, 1 equiv) in THF (10 mL) was added ethynylmagnesium bromide (0.5 M in THF, 1.27 mL, 0.64 mmol, 1.1 equiv) at -20 ºC. The reaction mixture was slowly warmed to 23 ºC and stirring was continued for 5 h. After addition of saturated NH_4Cl-solution, the layers were separated and the aqueous phase was extracted with EtOAc (2 x). The combined organic layers were washed with brine, dried ($MgSO_4$) and solvents were removed under reduced pressure. Purification by flash chromatography on silica using cyclohexane/EtOAc 30:1 as eluent yielded **S8a** (151 mg, 0.37 mmol, 63%) as a 1:1.1 mixture of diastereomers.
^{1}H NMR (400 MHz, $CDCl_3$) δ = 7.88 (dd, *J* = 7.7, 1.4 Hz, 1H isomer A), 7.41 (dd, *J* = 7.2, 1.6 Hz, 1H isomer B), 7.37 – 7.29 (m, 2H isomer A, 2H isomer B), 7.29 – 7.16 (m, 1H isomer A, 1H isomer B), 5.90 (s, 1H isomer A), 5.55 (dd, *J* = 6.8, 2.4 Hz, 1H isomer B), 5.35 (tdd, *J* = 8.4, 2.7, 1.4 Hz, 1H isomer A, 1H isomer B), 5.10 – 5.02 (m, 1H isomer A, 1H isomer B), 4.68 (ddd, *J* = 10.3, 8.5, 5.3 Hz, 1H isomer B), 4.52 (d, *J* = 6.8 Hz, 1H isomer B), 4.32 (d, *J* = 1.4 Hz, 1H isomer A), 4.09 (ddd, *J* = 10.1, 8.5, 5.0 Hz, 1H isomer A), 3.85 (dd, *J* = 9.0, 4.9 Hz, 1H isomer A), 3.83 (dd, *J* = 9.1, 5.2 Hz, 1H isomer B), 3.61 (dd, *J* = 10.4, 9.3 Hz, 1H isomer A), 3.56 (dd, *J* = 10.3, 9.4 Hz, 1H isomer B), 2.69 (d, *J* = 2.2 Hz, 1H isomer A), 2.58 (d, *J* = 2.3 Hz, 1H isomer B), 2.11 – 1.99 (m, 4H isomer A, 4H isomer B), 1.75 (d, *J* = 1.5 Hz, 3H isomer B), 1.67 (s, 3H isomer A, 3H isomer B), 1.65 (d, *J* = 1.3 Hz, 3H isomer A), 1.59 (s, 3H isomer B), 1.57 (s, 3H isomer A), 0.72 (s, 9H isomer B), 0.71 (s, 9H isomer A), -0.10 (s, 3H isomer A), -0.11 (s, 3H isomer B), -0.14 (s, 3H isomer A), -0.17 (s, 3H isomer B) ppm.
^{13}C NMR (101 MHz, $CDCl_3$) δ = 142.2, 141.9, 139.3, 139.0, 138.7, 138.2, 131.8, 131.7, 129.2, 129.1, 129.0, 128.1, 127.3, 127.1, 126.6, 126.5, 124.2, 124.0, 123.5, 123.4, 85.1, 83.2, 75.2, 74.4, 69.5, 69.3, 64.8, 61.8, 41.8, 41.1, 40.1, 39.9, 26.7, 26.6, 25.9 (3C), 25.8 (3C), 25.8, 25.8, 18.6, 18.4, 17.9, 17.8, 17.1, 17.1, -5.5, -5.6, -5.7, -5.7 ppm.
HRMS (ESI) *m/z* calc. for $C_{26}H_{40}O_2SiNa$ $[M+Na]^+$: 435.2690, found: 435.2697.

(*Z*)-1-(2-(1-((*tert*-butyldimethylsilyl)oxy)-4,8-dimethylnona-3,7-dien-2-yl)phenyl)prop-2-yn-1-ol (S8b)

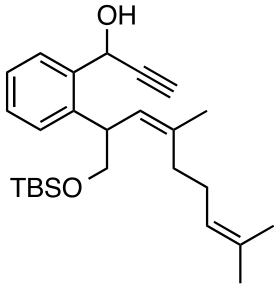

To a solution of **S7b** (110 mg, 0.28 mmol, 1 equiv) in THF (5 mL) was added ethynylmagnesium bromide (0.5 M in THF, 0.63 mL, 0.31 mmol, 1.1 equiv) at -20 ºC. The reaction mixture was slowly warmed to 23 ºC and stirring was continued for 5 h. After addition of saturated NH_4Cl-solution, the layers were separated and the aqueous phase was extracted with EtOAc (2 x). The combined organic layers were washed with brine, dried ($MgSO_4$) and solvents were removed under reduced pressure. Purification by flash chromatography on silica using cyclohexane/EtOAc 30:1 as eluent yielded **S8b** (58 mg, 0.14 mmol, 50%) as a 1:1.3 mixture of diastereomers.

^{1}H NMR (400 MHz, $CDCl_3$) δ = 7.89 (dd, *J* = 7.7, 1.4 Hz, 1H isomer A), 7.41 (dd, *J* = 7.8, 1.4 Hz, 1H isomer B), 7.39 – 7.29 (m, 2H isomer A, 2H isomer B), 7.24 (ddd, *J* = 7.8, 6.7, 1.8 Hz, 1H isomer A), 7.18 (td, *J* = 7.5, 1.4 Hz, 1H isomer B), 5.87 (s, 1H isomer A), 5.57 (dd, *J* = 6.8, 2.4 Hz, 1H isomer B), 5.44 – 5.34 (m, 1H isomer A, 1H isomer B), 5.11 – 5.05 (m, 1H isomer B), 5.05 – 4.99 (m, 1H isomer A), 4.67 (td, *J* = 9.7, 5.2 Hz, 1H isomer B), 4.42 (d, *J* = 6.8 Hz, 1H isomer B), 4.37 (d, *J* = 1.1 Hz, 1H isomer A), 4.11 (td, *J* = 9.8, 4.9 Hz, 1H isomer A), 3.85 (dd, *J* = 9.0, 4.9 Hz, 1H isomer A), 3.82 (dd, *J* = 8.8, 5.0 Hz, 1H isomer B), 3.55 (dd, *J* = 10.2, 9.4 Hz, 1H isomer A), 3.55 (dd, J = 10.2, 9.4 Hz, 1H isomer B), 2.68 (d, *J* = 2.2 Hz, 1H isomer A), 2.58 (d, *J* = 2.4 Hz, 1H isomer B), 2.41 – 2.32 (m, 1H isomer B), 2.13 – 1.95 (m, 4H isomer A, 3H isomer B), 1.74 (d, *J* = 1.4 Hz, 3H isomer B), 1.73 (d, *J* = 1.4 Hz, 3H isomer A), 1.66 (s, 3H isomer B), 1.63 (s, 3H isomer A), 1.59 (s, 3H isomer B), 1.57 (s, 3H isomer A), 0.71 (s, 9H isomer B), 0.69 (s, 9H isomer A), -0.11 (s, 3H isomer A), -0.12 (s, 3H isomer B), -0.16 (s, 3H isomer A), -0.19 (s, 3H isomer B) ppm.

^{13}C NMR (101 MHz, $CDCl_3$) δ = 142.3, 142.1, 139.2, 139.1, 138.9, 138.2, 132.4, 131.8, 129.2, 129.1, 129.0, 128.0, 127.4, 127.0, 126.6, 126.5, 124.4, 124.1, 124.0, 123.6, 85.0, 83.1, 75.2, 74.5, 69.8, 69.7, 64.7, 61.7, 41.5, 40.7, 33.0, 32.5, 26.6, 26.3, 25.9 (6C), 25.8, 25.8, 23.6 (2C), 18.6, 18.4, 17.9, 17.8, -5.5, -5.6, -5.7, -5.7 ppm.

HRMS (ESI) *m/z* calc. for $C_{26}H_{40}O_2SiNa$ $[M+Na]^+$: 435.2690, found: 435.2699.

(*E*)-*tert*-butyl((2-(2-(1-methoxyprop-2-yn-1-yl)phenyl)-4,8-dimethylnona-3,7-dien-1-yl)oxy)dimethylsilane (19a)

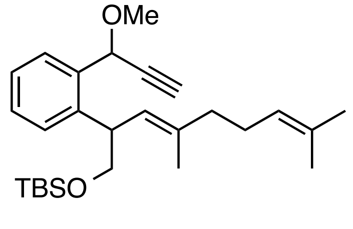

To a solution of **S8a** (35 mg, 0.085 mmol, 1 equiv) in THF (4 mL) was subsequently added sodium hydride (60% dispersion in mineral oil, 4.1 mg, 0.10 mmol, 1.15 equiv) and Me_2SO_4 (9.6 µl, 0.10 mmol, 1.15 equiv) at 0 ºC. The mixture was stirred for 5 h while slowly warming to 23

ºC and quenched by addition of water. After extraction of the aqueous phase with EtOAc (3 x) the combined organic layers were washed brine, dried ($MgSO_4$) and concentrated under reduced pressure. Purification of the crude product by flash chromatography on silica with cyclohexane/EtOAc (50:1) gave **19a** (33 mg, 77.3 µmol, 91%) as an inseparable mixture of diastereomers.

^{1}H NMR (400 MHz, $CDCl_3$) δ = 7.74 – 7.66 (m, 1H isomer B), 7.68 – 7.63 (m, 1H isomer A), 7.31 – 7.27 (m, 2H isomer A, 2H isomer B), 7.24 – 7.18 (m, 1H isomer A, 1H isomer B), 5.60 (d, J = 2.2 Hz, 1H isomer B), 5.41 – 5.34 (m, 2H isomer A, 1H isomer B), 5.13 – 5.04 (m, 1H isomer A, 1H isomer B), 4.12 – 3.99 (m, 1H isomer A, 1H isomer B), 3.79 – 3.63 (m, 2H isomer A, 2H isomer B), 3.46 (s, 3H isomer A), 3.42 (s, 3H isomer B), 2.64 (d, J = 2.2 Hz, 1H isomer A), 2.62 (d, J = 2.2 Hz, 1H isomer B), 2.12 – 1.99 (m, 4H isomer A, 4H isomer B), 1.67 (s, 6H isomer A, 3H isomer B), 1.63 (d, J = 1.3 Hz, 3H isomer B), 1.58 (s, 3H isomer A, 3H isomer B), 0.83 (s, 9H isomer A), 0.80 (s, 9H isomer B), -0.04 (s, 3H isomer A), -0.05 (s, 3H isomer A), -0.10 (s, 3H isomer B), -0.12 (s, 3H isomer B) ppm.

^{13}C NMR (101 MHz, $CDCl_3$) δ = 142.5, 141.6, 137.8, 137.3, 135.9, 135.7, 131.6, 131.5, 128.8, 128.6, 128.0, 127.8, 127.5, 126.3, 126.1, 125.6, 124.7 (2C), 124.4 (2C), 81.8, 81.7, 76.1, 75.9, 70.2, 70.2, 68.7, 67.9, 56.0, 55.8, 42.0, 41.7, 40.1, 40.0, 26.8, 26.8, 26.0 (6C), 25.8 (2C), 18.5, 18.4, 17.8 (2C), 17.0, 16.7, -5.3, -5.3, -5.4, -5.4 ppm.

HRMS (ESI) *m/z* calc. for $C_{27}H_{42}O_2SiNa$ $[M+Na]^+$: 449.2846, found: 449.2855.

(*Z*)-*tert*-butyl((2-(2-(1-methoxyprop-2-yn-1-yl)phenyl)-4,8-dimethylnona-3,7-dien-1-yl)oxy)dimethylsilane (19b)

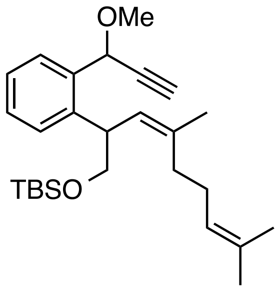

To a solution of **S8b** (58 mg, 0.14 mmol, 1 equiv) in THF (3 mL) was subsequently added sodium hydride (60% dispersion in mineral oil, 7 mg, 0.17 mmol, 1.2 equiv) and Me_2SO_4 (16 µl. 0.17 mmol, 1.2 equiv) at 0 ºC. The mixture was stirred for 5 h while slowly warming to 23 ºC and quenched by addition of water. After extraction of the aqueous phase with EtOAc (3 x) the combined organic layers were washed brine, dried ($MgSO_4$) and concentrated under reduced pressure. Purification of the crude product by flash chromatography on silica with cyclohexane/EtOAc (50:1) gave **19b** (54 mg, 0.17 mmol, 90%) as an inseparable mixture of diastereomers.

^{1}H NMR (400 MHz, $CDCl_3$) δ = 7.72 – 7.69 (m, 1H isomer B), 7.65 (dd, J = 7.7, 1.5 Hz, 1H isomer A), 7.32 – 7.27 (m, 2H isomer A, 2H isomer B), 7.23 – 7.17 (m, 1H isomer A, 1H isomer B), 5.62 (d, J = 2.2 Hz, 1H isomer B), 5.46 – 5.38 (m, 2H isomer A, 1H isomer B), 5.11 – 5.00 (m, 1H isomer A, 1H isomer B), 4.13 – 4.00 (m, 1H isomer A, 1H isomer B), 3.77 – 3.61 (m, 2H isomer A, 2H isomer B), 3.45 (s, 3H isomer A), 3.41 (s, 3H isomer B), 2.63 (d, J = 2.2 Hz, 1H isomer B), 2.61 (d, J = 2.2 Hz, 1H isomer A), 2.20 – 1.95 (m, 4H isomer A, 4H isomer B), 1.73 (s, 3H isomer A, 3H isomer B), 1.64 (s, 3H isomer A, 3H isomer B), 1.57 (s, 3H isomer A, 3H

isomer B), 0.83 (s, 9H isomer A), 0.79 (s, 9H isomer B), -0.06 (s, 3H isomer A), -0.07 (s, 3H isomer A), -0.12 (s, 3H isomer B), -0.14 (s, 3H isomer B) ppm.
^{13}C NMR (101 MHz, $CDCl_3$) δ = 142.7, 141.8, 138.0, 137.4, 135.8, 135.7, 131.7, 131.5, 128.7, 128.5, 128.2, 127.9, 127.8, 127.5, 126.3 (2C), 126.0, 125.2, 124.5, 124.4, 81.8, 81.7, 76.1, 76.0, 70.2, 70.1, 69.1, 68.3, 55.9, 55.7, 41.7, 41.3, 32.8, 32.5, 26.6, 26.6, 26.0 (6C), 25.8, 25.8, 23.7, 23.6, 18.5, 18.4, 17.8 (2C), -5.3, -5.3, -5.4, -5.5 ppm.
HRMS (ESI) *m/z* calc. for $C_{27}H_{42}O_2SiNa$ $[M+Na]^+$: 449.2846, found: 449.2851.

(*E*)-((2-(2-(1-(benzyloxy)prop-2-yn-1-yl)phenyl)-4,8-dimethylnona-3,7-dien-1-yl)oxy)(*tert*-butyl)dimethylsilane (S9a)

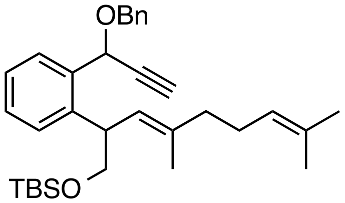

To a solution of **S8a** (15 mg, 36.3 µmol, 1 equiv) in THF (2 mL) was subsequently added sodium hydride (60% dispersion in mineral oil, 1.7 mg, 43.6 µmol, 1.2 equiv) and benzyl bromide (6.5 µl, 54.5 µmol, 1.5 equiv) at 0 ºC. The mixture was slowly warmed to 23 ºC and stirring was continued overnight. After adding saturated NH_4Cl-solution, the aqueous phase was extracted with EtOAc (3 x) the combined organic layers were washed brine, dried ($MgSO_4$) and concentrated under reduced pressure. Purification of the crude product by flash chromatography on silica with cyclohexane/EtOAc (100:1) gave **S9a** (11.5 mg, 22.9 µmol, 63%) as an inseparable mixture of diastereomers.
It was not possible to distinguish the two isomers in the ^{1}H-NMR spectra. Moreover, due to overlapping in the aromatic region some signals in the ^{13}C-NMR spectra are not resolved.
^{1}H NMR (400 MHz, $CDCl_3$) δ = 7.79 – 7.70 (m, 2H), 7.43 – 7.39 (m, 2H), 7.38 – 7.27 (m, 12H), 7.25 – 7.18 (m, 2H), 5.72 (d, *J* = 2.2 Hz, 1H), 5.52 (d, *J* = 2.2 Hz, 1H), 5.39 – 5.30 (m, 2H), 5.12 – 5.03 (m, 2H), 4.77 (d, *J* = 11.4 Hz, 1H), 4.72 – 4.62 (m, 3H), 4.08 – 3.95 (m, 2H), 3.75 (dd, *J* = 9.6, 5.9 Hz, 1H), 3.71 – 3.61 (m, 3H), 2.69 – 2.63 (m, 2H), 2.10 – 1.93 (m, 8H), 1.67 (s, 3H), 1.66 (s, 3H), 1.58 (s, 6H), 1.54 (d, *J* = 1.3 Hz, 3H), 1.47 (d, *J* = 1.3 Hz, 3H), 0.83 (s, 9H), 0.80 (s, 9H), -0.06 (s, 6H), -0.11 (s, 3H), -0.12 (s, 3H) ppm.
^{13}C NMR (101 MHz, $CDCl_3$) δ = 142.3, 141.6, 137.9, 137.8, 137.7, 137.2, 136.0, 135.9, 131.5 (2C), 128.7, 128.6, 128.5, 128.4, 128.4, 128.3, 128.0, 127.9, 127.9, 127.9, 127.8, 126.3, 126.2, 125.6, 124.8, 124.4, 124.4, 82.1, 82.0, 76.1, 76.1, 70.4, 70.1, 68.5, 68.0, 67.9, 67.7, 41.8, 41.7, 40.0, 40.0, 26.8, 26.8, 26.0 (6C), 25.8, 25.8, 18.4, 18.4, 17.8, 17.8, 17.0, 16.9, -5.3, -5.3, -5.4, -5.4.
HRMS (ESI) *m/z* calc. for $C_{33}H_{46}O_2SiNa$ $[M+Na]^+$: 525.3159, found: 525.3166.

(*Z*)-((2-(2-(1-(benzyloxy)prop-2-yn-1-yl)phenyl)-4,8-dimethylnona-3,7-dien-1-yl)oxy)(*tert*-butyl)dimethylsilane (S9b)

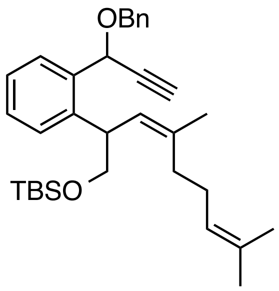

To a solution of **S8b** (40 mg, 96.9 µmol, 1 equiv) in THF (2 mL) was subsequently added sodium hydride (60% dispersion in mineral oil, 4.7 mg, 0.12 mmol, 1.2 equiv) and benzyl bromide (17 µl, 0.15 mmol, 1.5 equiv) at 0 ºC. The mixture was slowly warmed to 23 ºC and stirring was continued overnight. After adding saturated NH_4Cl-solution, the aqueous phase was extracted with EtOAc (3 x) the combined organic layers were washed brine, dried ($MgSO_4$) and concentrated under reduced pressure. Purification of the crude product by flash chromatography on silica with cyclohexane/EtOAc (100:1) gave **S9b** (36 mg, 71.6 µmol, 74%) as an inseparable mixture of diastereomers.

Due to overlapping in the aromatic region some signals in the ^{13}C-NMR spectra are not resolved.

^{1}H NMR (400 MHz, $CDCl_3$) δ = 7.78 – 7.70 (m, 1H isomer A, 1H isomer B), 7.44 – 7.39 (m, 1H isomer A, 1H isomer B), 7.37 – 7.25 (m, 6H isomer A, 6H isomer B), 7.25 – 7.18 (m, 1H isomer A, 1H isomer B), 5.73 (d, *J* = 2.2 Hz, 1H isomer B), 5.54 (d, *J* = 2.2 Hz, 1H isomer A), 5.44 – 5.35 (m, 1H isomer A, 1H isomer B), 5.02 – 4.96 (m, 1H isomer A), 4.96 – 4.86 (m, 1H isomer B), 4.77 (d, *J* = 11.4 Hz, 1H isomer A), 4.71 – 4.61 (m, 1H isomer A, 2H isomer B), 4.08 (q, *J* = 7.4 Hz, 1H isomer B), 3.99 (dt, *J* = 9.0, 6.4 Hz, 1H isomer A), 3.75 – 3.60 (m, 2H isomer A, 2H isomer B), 2.65 (d, *J* = 2.2 Hz, 1H isomer A), 2.65 (d, *J* = 2.3 Hz, 1H isomer B), 2.09 – 1.81 (m, 4H isomer A, 4H isomer B), 1.70 (d, *J* = 1.4 Hz, 3H isomer A), 1.69 (d, *J* = 1.4 Hz, 3H isomer B), 1.63 (s, 3H isomer A), 1.59 (s, 3H isomer B), 1.55 (s, 3H isomer A), 1.50 (s, 3H isomer B), 0.82 (s, 9H isomer A), 0.79 (s, 9H isomer B), -0.09 (s, 6H isomer A), -0.12 (s, 3H isomer B), -0.14 (s, 3H isomer B) ppm.

^{13}C NMR (101 MHz, $CDCl_3$) δ = 142.3, 141.7, 138.0, 137.9, 137.7, 137.3, 136.0, 135.7, 131.6, 131.5, 128.6, 128.5, 128.5, 128.4, 128.4, 128.2, 128.1, 128.0, 128.0, 127.9, 127.8, 127.7, 126.3 (2C), 126.2, 125.5, 124.5, 124.4, 82.2, 81.9, 76.2, 76.0, 70.4, 69.9, 68.7, 68.1, 68.0, 67.9, 41.5 (2C), 32.6, 32.5, 26.5, 26.4, 26.0 (6C), 25.83, 25.8, 23.6, 23.5, 18.5, 18.4, 17.8, 17.8, -5.3, -5.3, -5.4, -5.4 ppm.

HRMS (ESI) *m/z* calc. for $C_{33}H_{46}O_2SiNa$ $[M+Na]^+$: 525.3159, found: 525.3162.

(*E*)-2-(2-(1-methoxyprop-2-yn-1-yl)phenyl)-4,8-dimethylnona-3,7-dien-1-ol (9a)

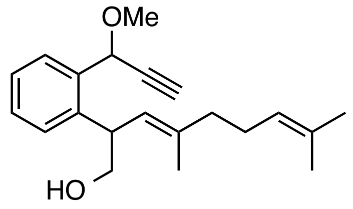

A solution of **19a** (59 mg, 0.14 mmol, 1 equiv) in THF (1.2 mL) was treated with 10% aqueous HCl (0.6 mL) and the resulting mixture was stirred for 2 h at rt. Then saturated $NaHCO_3$-solution was added and the aqueous phase was extracted with EtOAc (3 x). The combined organic layers were washed with brine, dried ($MgSO_4$) and concentrated under reduced pressure. Purification by flash chromatography on silica gel using cyclohexane/EtOAc 6:1 as eluent yielded **9a** (40 mg, 0.13 mmol, 91%).

It was not possible to distinguish the two isomers in the ^{1}H-NMR spectra. Not possible to assign all the signals.

^{1}H NMR (400 MHz, $CDCl_3$) δ = 7.74 – 7.66 (m, 1H), 7.60 (dt, *J* = 7.6, 1.0 Hz, 1H), 7.39 – 7.29 (m, 4H), 7.28 – 7.21 (m, 2H), 5.38 – 5.29 (m, 4H), 5.11 – 5.00 (m, 2H), 4.23 (dt, *J* = 9.0, 7.4 Hz, 1H), 4.12 (dt, *J* = 9.1, 7.3 Hz, 1H), 3.79 – 3.63 (m, 4H), 3.48 (s, 3H), 3.47 (s, 3H), 2.71 (d, *J* = 2.2 Hz, 1H), 2.66 (d, *J* = 2.2 Hz, 1H), 2.16 – 2.00 (m, 9H), 1.90 (bs, 1H), 1.71 (d, *J* = 1.3 Hz, 6H), 1.67 (s, 6H), 1.59 (s, 6H) ppm.

^{13}C NMR (101 MHz, $CDCl_3$) δ = 141.3, 140.9, 139.4, 139.06, 136.2, 135.5, 132.0, 132.0, 129.4, 129.2, 128.4, 128.4, 127.8, 127.6, 126.7, 126.6, 124.8, 124.4, 124.2, 124.1, 81.4, 81.0, 76.9, 76.4, 71.0, 70.8, 67.6, 67.4, 56.4, 56.0, 42.2, 42.0, 40.0, 39.9, 26.6, 26.5, 25.8 (2C), 17.8 (2C), 16.9, 16.8 ppm.

HRMS (ESI) *m/z* calc. for $C_{21}H_{28}O_2Na$ $[M+Na]^+$: 335.1982, found: 335.1981.

(*Z*)-2-(2-(1-methoxyprop-2-yn-1-yl)phenyl)-4,8-dimethylnona-3,7-dien-1-ol (9b)

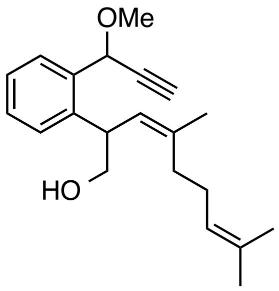

A solution of **19b** (36 mg, 84.4 µmol, 1 equiv) in THF (0.7 mL) was treated with 10% aqueous HCl (0.35 mL) and the resulting mixture was stirred for 1 h at rt. Then saturated $NaHCO_3$-solution was added and the aqueous phase was extracted with EtOAc (3 x). The combined organic layers were washed with brine, dried ($MgSO_4$) and concentrated under reduced pressure. Purification by flash chromatography on silica gel using cyclohexane/EtOAc 6:1 as eluent yielded **9b** (24 mg, 76.8 mmol, 91%). The two isomers could be separated by prep-TLC.

It was not possible to distinguish the relative configuration of the two isomers by ^{1}H-NMR spectra.

Isomer A: **^{1}H NMR** (500 MHz, $CDCl_3$) δ = 7.61 (dt, *J* = 7.6, 1.0 Hz, 1H), 7.38 – 7.31 (m, 2H), 7.28 – 7.20 (m, 1H), 5.41 (d, *J* = 9.2 Hz, 1H), 5.36 (d, *J* = 2.2 Hz, 1H), 5.11 – 5.03 (m, 1H), 4.22 (dt, *J* = 9.2, 7.2 Hz, 1H), 3.72 – 3.67 (m, 2H), 3.46 (s, 3H), 2.65 (d, *J* = 2.2 Hz, 1H), 2.30 – 2.21 (m, 1H), 2.13 – 1.99 (m, 3H), 1.80 (t, *J* = 6.2 Hz, 1H), 1.76 (d, *J* = 1.4 Hz, 3H), 1.66 (s, 3H), 1.58 (s, 3H) ppm.

^{13}C NMR (101 MHz, $CDCl_3$) δ = 141.0, 139.6, 136.1, 132.1, 129.2, 128.4, 127.8, 126.7, 124.9, 124.1, 81.5, 76.4, 70.8, 68.0, 56.3, 41.9, 32.5, 26.6, 25.8, 23.7, 17.8 ppm.

Isomer B: **^{1}H NMR** (500 MHz, $CDCl_3$) δ = 7.74 – 7.71 (m, 1H), 7.37 – 7.33 (m, 2H), 7.26 – 7.21 (m, 1H), 5.41 – 5.35 (m, 2H), 5.06 (ddp, *J* = 6.9, 5.5, 1.4 Hz, 1H), 4.08 (dt, *J* = 9.2, 7.3 Hz, 1H),

3.74 – 3.67 (m, 2H), 3.49 (s, 3H), 2.72 (d, *J* = 2.2 Hz, 1H), 2.22 – 2.14 (m, 1H), 2.14 – 1.98 (m, 4H), 1.75 (d, *J* = 1.4 Hz, 3H), 1.65 (s, 3H), 1.58 (s, 3H) ppm.
^{13}C NMR (101 MHz, $CDCl_3$) δ = 141.7, 139.1, 135.2, 132.27, 129.5, 128.4, 127.7, 126.5, 125.3, 123.9, 80.8, 77.1, 70.7, 67.9, 55.9, 41.7, 32.7, 26.5, 25.8, 23.6, 17.8 ppm.

HRMS (ESI) *m/z* calc. for $C_{21}H_{28}O_2Na$ $[M+Na]^+$: 335.1982, found: 335.1988.

***E*)-2-(2-(1-(benzyloxy)prop-2-yn-1-yl)phenyl)-4,8-dimethylnona-3,7-dien-1-ol (10a)**

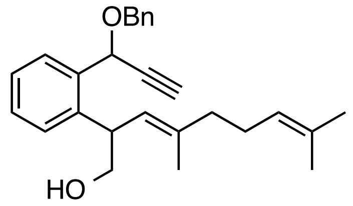

A solution of **S9a** (10 mg, 19.9 µmol, 1 equiv) in THF (0.2 mL) was treated with 10% aqueous HCl (0.1 mL) and the resulting mixture was stirred for 1.5 h at rt. Then saturated $NaHCO_3$-solution was added and the aqueous phase was extracted with EtOAc (3 x). The combined organic layers were washed with brine, dried ($MgSO_4$) and concentrated under reduced pressure. Purification by flash chromatography on silica gel using cyclohexane/EtOAc 6:1 as eluent yielded **10a** (5.5 mg, 14.2 mmol, 71%) as a mixture of diastereomers.
It was not possible to distinguish the two isomers in the ^{1}H-NMR spectra.
^{1}H NMR (400 MHz, $CDCl_3$) δ = 7.75 (dd, *J* = 7.7, 1.5 Hz, 1H), 7.64 – 7.60 (m, 1H), 7.46 – 7.42 (m, 2H), 7.39 – 7.23 (m, 14H), 5.45 (t, *J* = 2.4 Hz, 2H), 5.31 (dq, *J* = 9.0, 1.3 Hz, 1H), 5.27 (dq, *J* = 9.1, 1.3 Hz, 1H), 5.10 – 4.98 (m, 2H), 4.83 – 4.65 (m, 4H), 4.18 (dt, *J* = 8.8, 7.3 Hz, 1H), 3.95 (dt, *J* = 9.2, 7.4 Hz, 1H), 3.71 – 3.58 (m, 4H), 2.76 (d, *J* = 2.2 Hz, 1H), 2.67 (d, *J* = 2.2 Hz, 1H), 2.13 – 1.95 (m, 8H), 1.92 – 1.86 (m, 1H), 1.78 – 1.69 (m, 1H), 1.67 (d, *J* = 1.4 Hz, 3H), 1.66 (d, *J* = 1.4 Hz, 3H), 1.60 – 1.55 (m, 9H), 1.47 (d, *J* = 1.4 Hz, 3H) ppm.
^{13}C NMR (101 MHz, $CDCl_3$) δ = 141.2, 140.7, 139.5, 139.0, 137.4, 137.0, 136.4, 135.5, 132.0 (2C), 129.4, 129.1, 129.0 (2C), 128.7, 128.7 (2C), 128.6 (2C), 128.4, 128.4 (2C), 128.3, 128.0, 127.7, 127.6, 126.8, 126.6, 124.7, 124.4, 124.2, 124.1, 81.8, 81.3, 77.4, 76.3, 70.5, 70.5, 68.3, 67.9, 67.5, 67.3, 42.2, 41.7, 40.0, 39.9, 26.6, 26.5, 25.8, 25.8, 17.9, 17.8, 16.9, 16.7 ppm.
HRMS (ESI) *m/z* calc. for $C_{27}H_{32}O_2Na$ $[M+Na]^+$: 411.2295, found: 411.2302.

(*Z*)-2-(2-(1-(benzyloxy)prop-2-yn-1-yl)phenyl)-4,8-dimethylnona-3,7-dien-1-ol (10b)

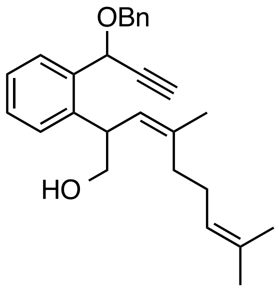

A solution of **S9b** (36 mg, 71.6 µmol, 1 equiv) in THF (0.7 mL) was treated with 10% aqueous HCl (0.35 mL) and the resulting mixture was stirred for 1.5 h at rt. Then saturated $NaHCO_3$-solution was added and the aqueous phase was extracted with EtOAc (3 x). The combined organic

layers were washed with brine, dried ($MgSO_4$) and concentrated under reduced pressure. Purification by flash chromatography on silica gel using cyclohexane/EtOAc 6:1 as eluent yielded **10b** (25 mg, 64.3 mmol, 90%) as a mixture of diastereomers. They could be separated by collection of chiral HPLC fractions.
It was not possible to distinguish the relative configuration of the two isomers by ^{1}H-NMR spectra.
Isomer 1: **^{1}H NMR** (400 MHz, $CDCl_3$) δ 7.65 – 7.58 (m, 1H), 7.40 – 7.22 (m, 8H), 5.46 (d, J = 2.3 Hz, 1H), 5.36 (dd, J = 9.0, 1.4 Hz, 1H), 4.99 (tt, J = 6.9, 1.4 Hz, 1H), 4.79 (d, J = 11.6 Hz, 1H), 4.65 (d, J = 11.6 Hz, 1H), 4.20 (dt, J = 9.1, 7.2 Hz, 1H), 3.73 – 3.60 (m, 2H), 2.67 (d, J = 2.3 Hz, 1H), 2.18 (ddd, J = 12.4, 8.6, 6.2 Hz, 1H), 2.05 – 1.83 (m, 3H), 1.71 (d, J = 1.4 Hz, 3H), 1.63 (d, J = 1.4 Hz, 3H), 1.55 (s, 3H).
^{13}C NMR (101 MHz, $CDCl_3$) δ 140.8, 139.5, 137.5, 136.5, 132.1, 129.1, 128.6, 128.4, 128.3, 128.0, 127.8, 126.8, 125.0, 124.2, 81.9, 76.3, 70.6, 68.5, 67.8, 42.0, 32.5, 26.5, 25.8, 23.6, 17.8.

Isomer 2: **^{1}H NMR** (400 MHz, $CDCl_3$) δ 7.78 (dd, J = 7.8, 1.4 Hz, 1H), 7.48 – 7.41 (m, 2H), 7.41 – 7.29 (m, 5H), 7.25 – 7.21 (m, 1H), 5.48 (d, J = 2.2 Hz, 1H), 5.33 (d, J = 9.3 Hz, 1H), 4.99 – 4.89 (m, 1H), 4.79 (d, J = 11.5 Hz, 1H), 4.71 (d, J = 11.4 Hz, 1H), 3.96 – 3.86 (m, 1H), 3.64 (d, J = 7.3 Hz, 2H), 2.77 (d, J = 2.3 Hz, 1H), 2.04 – 1.79 (m, 4H), 1.70 (d, J = 1.4 Hz, 3H), 1.62 (s, 3H), 1.55 (s, 3H).
^{13}C NMR (101 MHz, $CDCl_3$) δ 141.5, 139.0, 137.0, 135.3, 132.1, 129.5, 129.0, 128.7 (2C), 128.3, 127.7, 126.6, 125.3, 123.9, 81.1, 77.3, 70.6, 67.8, 67.7, 41.4, 32.4, 26.3, 25.8, 23.6, 17.9.

HRMS (ESI) *m/z* calc. for $C_{27}H_{32}O_2Na$ $[M+Na]^+$: 411.2295, found: 411.2298.

(*E*)-2-(2-(1-methoxyprop-2-yn-1-yl)phenyl)-4,8-dimethylnona-3,7-dienoic acid (4a)

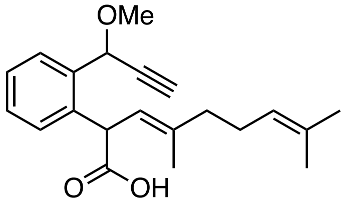

Step 1: To a suspension of **9a** (29 mg, 92.8 µmol, 1 equiv) and $NaHCO_3$ (78 mg) in CH_2Cl_2 (2 mL) was added Dess-Martin periodinane (59 mg, 0.14 mmol, 1.5 equiv). The mixture was stirred for 1.5 h at 23 ºC and then quenched by the addition of saturated $Na_2S_2O_3$ solution. The layers were separated and the aqueous phase was extracted with CH_2Cl_2 (2 x). The combined organic layers were washed with brine, dried ($MgSO_4$) and concentrated. The crude product was used in the next step without further purification.
Step 2: To a solution of the crude aldehyde in *t*BuOH (2 mL) and water (0.5 mL) was added 2-methyl-2-butene (0.20 mL), NaH_2PO_4 (45 mg, 0.37 mmol, 4 equiv) and $NaClO_2$ (80%, 42 mg, 0.37 mmol, 4 equiv). The resulting mixture was stirred at 23 ºC for 2 h. After adding saturated NH_4Cl-solution, the aqueous phase was extracted with Et_2O (3 x) the combined organic layers were washed brine, dried ($MgSO_4$) and concentrated under reduced pressure. Purification by flash chromatography on silica gel using cyclohexane/EtOAc 6:1with 0.1% AcOH as eluent yielded **4a** (21 mg, 64.3 µmol, 69%) as a 1:1 mixture of diastereomers.
It was not possible to distinguish the two isomers in the ^{1}H-NMR spectra.

^{1}H NMR (500 MHz, $CDCl_3$) δ = 7.69 (dd, *J* = 7.7, 1.5 Hz, 1H), 7.61 (dd, *J* = 7.7, 1.5 Hz, 1H), 7.47 (dd, *J* = 3.6, 1.4 Hz, 1H), 7.46 (dd, *J* = 3.6, 1.3 Hz, 1H), 7.39 – 7.30 (m, 2H), 7.30 – 7.25 (m, 2H), 5.71 (dd, *J* = 8.7, 1.4 Hz, 1H), 5.67 (dd, *J* = 8.8, 1.4 Hz, 1H), 5.45 (d, *J* = 2.2 Hz, 1H), 5.39 (d, *J* = 2.2 Hz, 1H), 5.10 – 5.02 (m, 3H), 4.86 (d, *J* = 8.8 Hz, 1H), 3.45 (s, 6H), 2.72 (d, *J* = 2.2 Hz, 1H), 2.68 (d, *J* = 2.2 Hz, 1H), 2.13 – 2.04 (m, 8H), 1.68 – 1.62 (m, 12H), 1.57 (s, 3H), 1.56 (s, 2H) ppm.
^{13}C NMR (126 MHz, $CDCl_3$) δ = 178.3, 177.5, 140.1, 139.7, 138.0, 137.8, 135.2, 134.9, 131.9, 131.9, 129.5, 129.4, 129.3, 129.1, 128.8, 128.3, 127.5, 127.4, 124.0, 123.9, 121.7, 121.2, 80.6, 80.3, 77.4, 77.1, 71.5, 70.9, 56.1, 55.9, 45.8, 45.8, 39.8, 39.7, 26.5, 26.5, 25.8, 25.8, 17.8, 17.8, 17.0, 16.8 ppm.
HRMS (ESI) *m/z* calc. for $C_{21}H_{26}O_3Na$ $[M+Na]^+$: 349.1774, found: 349.1773.

1.2.2 Synthesis of simpler substrates 15-16

(*E*)-2-(3,7-dimethylocta-2,6-dien-1-yl)benzaldehyde (S10a)

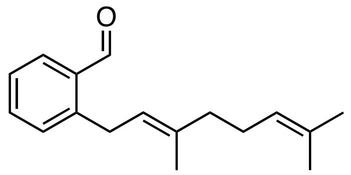

To a solution of commercially available 2-bromobenzaldehyde diethylacetal (500 mg, 1.93 mmol, 1 equiv) in THF (10 mL) was added *n*BuLi (2.5 M in hexanes, 0.92 mL, 2.12 mmol, 1.1 equiv) at -78 ºC. The reaction mixture was stirred at -78 ºC for 1 h. Then a solution of geranyl bromide (commercially available) (628 mg, 2.89 mmol, 1.5 equiv) in THF (2 mL) was added and the mixture was slowly warmed to 23 ºC by stirring overnight. After addition of 5% HCl (10 mL) stirring was continued for 1 h. Then layers were separated and the aqueous phase was extracted with EtOAc (2 x). The combined organic layers were washed with brine, dried ($MgSO_4$) and concentrated under reduced pressure. Purification of the crude material by flash chromatography on silica with cyclohexane/EtOAc 100:1 yielded **S10a** (301 mg, 1.24 mmol, 64%).
^{1}H NMR (400 MHz, $CDCl_3$) δ = 10.29 (s, 1H), 7.84 (dd, *J* = 7.7, 1.5 Hz, 1H), 7.50 (td, *J* = 7.5, 1.5 Hz, 1H), 7.41 – 7.29 (m, 2H), 5.28 (tp, *J* = 7.0, 1.3 Hz, 1H), 5.06 (ddq, *J* = 8.3, 5.7, 1.4 Hz, 1H), 3.78 (d, *J* = 7.0 Hz, 2H), 2.13 – 2.00 (m, 4H), 1.72 (s, 3H), 1.66 (d, *J* = 1.4 Hz, 3H), 1.58 (s, 3H) ppm.
^{13}C NMR (101 MHz, $CDCl_3$) δ = 192.6, 144.6, 136.9, 134.1, 134.0, 131.7, 131.2, 130.6, 126.6, 124.2, 122.7, 39.8, 31.2, 26.7, 25.8, 17.8, 16.4 ppm.
HRMS (APCI) *m/z* calc. for $C_{17}H_{23}O$ $[M+H]^+$: 243.1743, found: 243.1740.

(*Z*)-2-(3,7-dimethylocta-2,6-dien-1-yl)benzaldehyde (S10b)

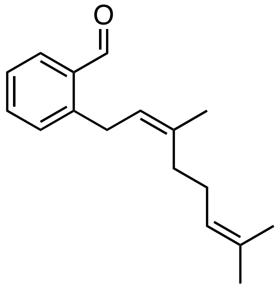

The title compound **S10b** was prepared by the same procedure as for the (*E*)-isomer, using commercially available neryl bromide instead of geranyl bromide, in 58% yield.

^{1}H NMR (300 MHz, $CDCl_3$) δ = 10.29 (s, 1H), 7.83 (dd, *J* = 7.6, 1.5 Hz, 1H), 7.50 (td, *J* = 7.5, 1.6 Hz, 1H), 7.41 – 7.28 (m, 2H), 5.29 (td, *J* = 7.1, 1.6 Hz, 1H), 5.20 – 5.07 (m, 1H), 3.83 – 3.74 (m, 2H), 2.23 – 2.01 (m, 4H), 1.74 (d, *J* = 1.3 Hz, 3H), 1.68 (s, 3H), 1.62 (s, 3H) ppm.

^{13}C NMR (126 MHz, $CDCl_3$) δ = 192.6, 144.7, 137.0, 134.0, 133.9, 132.0, 131.3, 130.6, 126.6, 124.1, 123.3, 32.3, 30.9, 26.5, 25.9, 23.5, 17.8 ppm.

HRMS (ESI) *m/z* calc. for $C_{17}H_{22}ONa$ $[M+Na]^+$: 265.1568, found: 265.1558.

(*E*)-1-(2-(3,7-dimethylocta-2,6-dien-1-yl)phenyl)prop-2-yn-1-ol (S11a)

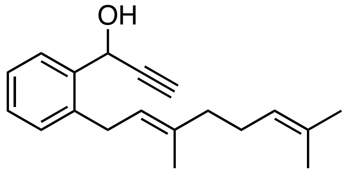

To a solution of **S10a** (1.10 g, 4.54 mmol, 1 equiv) in THF (40 mL) was added ethynylmagnesium bromide (0.5 M in THF, 10 mL, 4.99 mmol, 1.1 equiv) at 0 ºC. The reaction mixture was slowly warmed to 23 ºC and stirred overnight. After addition of saturated NH_4Cl-solution, the layers were separated and the aqueous phase was extracted with EtOAc (2 x). The combined organic layers were washed with brine, dried ($MgSO_4$) and solvents were removed under reduced pressure. Purification by flash chromatography on silica using cyclohexane/EtOAc 20:1 as eluent yielded **S11a** (849 mg, 3.16 mmol, 70%).

^{1}H NMR (400 MHz, $CDCl_3$) δ = 7.75 – 7.67 (m, 1H), 7.30 – 7.20 (m, 3H), 5.67 (dd, *J* = 5.7, 2.3 Hz, 1H), 5.28 (tq, *J* = 7.1, 1.3 Hz, 1H), 5.09 (ddp, *J* = 6.8, 4.3, 1.5 Hz, 1H), 3.61 – 3.44 (m, 2H), 2.65 (d, *J* = 2.3 Hz, 1H), 2.19 – 2.02 (m, 5H), 1.74 (d, *J* = 1.2 Hz, 3H), 1.68 (d, *J* = 1.4 Hz, 3H), 1.59 (s, 3H) ppm.

^{13}C NMR (126 MHz, $CDCl_3$) δ = 139.5, 137.9, 137.0, 131.8, 129.9, 128.9, 127.0, 126.6, 124.3, 122.8, 83.7, 74.8, 62.0, 39.8, 31.3, 26.7, 25.9, 17.9, 16.4 ppm.

HRMS (ESI) *m/z* calc. for $C_{19}H_{24}ONa$ $[M+Na]^+$: 291.1719, found: 291.1720.

(*Z*)-1-(2-(3,7-dimethylocta-2,6-dien-1-yl)phenyl)prop-2-yn-1-ol (S11b)

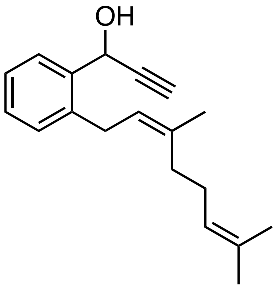

The title compound, **S11b**, was prepared from **S10b**, following the same procedure as for the (*E*)-isomer, in 84% yield.
^{1}H NMR (500 MHz, $CDCl_3$) δ = 7.73 – 7.68 (m, 1H), 7.30 – 7.20 (m, 3H), 5.67 (d, *J* = 2.3 Hz, 1H), 5.32 – 5.24 (m, 1H), 5.15 (ddt, *J* = 6.8, 5.4, 1.4 Hz, 1H), 3.59 – 3.45 (m, 2H), 2.65 (d, *J* = 2.3 Hz, 1H), 2.27 – 2.09 (m, 5H), 1.75 (d, *J* = 1.4 Hz, 3H), 1.70 (d, *J* = 1.4 Hz, 3H), 1.63 (s, 3H) ppm.
^{13}C NMR (126 MHz, $CDCl_3$) δ = 139.6, 137.8, 137.1, 132.0, 129.9, 128.9, 126.9, 126.6, 124.2, 123.5, 83.7, 74.8, 62.0, 32.2, 31.0, 26.6, 25.9, 23.5, 17.8 ppm.
HRMS (ESI) *m/z* calc. for $C_{19}H_{24}ONa$ $[M+Na]^+$: 291.1725, found: 291.1718.

(*E*)-1-(3,7-dimethylocta-2,6-dien-1-yl)-2-(1-methoxyprop-2-yn-1-yl)benzene (15a)

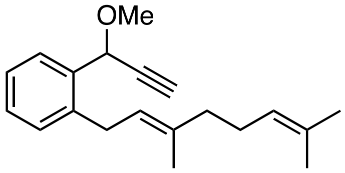

To a solution of **S11a** (250 mg, 0.93 mmol, 1 equiv) in THF (15 mL) was subsequently added sodium hydride (60% dispersion in mineral oil, 45 mg, 1.11 mmol, 1.2 equiv) and Me_2SO_4 (0.11 mL, 1.11 mmol, 1.2 equiv) at 0 ºC. The mixture was stirred for 10 min at 0 ºC and then for 3 h at 23 ºC before it was quenched by addition of water. After extraction of the aqueous phase with EtOAc (2 x) the combined organic layers were washed brine, dried ($MgSO_4$) and concentrated under reduced pressure. Purification of the crude product by flash chromatography on silica with cyclohexane/EtOAc (50:1) gave **15a** (250 mg, 0.89 mmol, 95%).
^{1}H NMR (500 MHz, $CDCl_3$) δ = 7.65 (dd, *J* = 7.4, 1.8 Hz, 1H), 7.29 – 7.19 (m, 3H), 5.28 (tq, *J* = 7.0, 1.3 Hz, 1H), 5.25 (d, *J* = 2.2 Hz, 1H), 5.11 (tq, *J* = 6.9, 1.5 Hz, 1H), 3.51 – 3.45 (m, 2H), 3.45 (s, 3H), 2.63 (d, *J* = 2.2 Hz, 1H), 2.16 – 2.03 (m, 4H), 1.73 (s, 3H), 1.69 (d, *J* = 1.4 Hz, 3H), 1.60 (s, 3H) ppm.
^{13}C NMR (101 MHz, $CDCl_3$) δ = 139.9, 136.6, 135.9, 131.7, 129.6, 128.8, 127.8, 126.3, 124.3, 122.7, 81.6, 75.7, 70.3, 56.3, 39.8, 31.1, 26.7, 25.9, 17.8, 16.4 ppm.
HRMS (ESI) *m/z* calc. for $C_{20}H_{26}ONa$ $[M+Na]^+$: 305.1876, found: 305.1864.

(*Z*)-1-(3,7-dimethylocta-2,6-dien-1-yl)-2-(1-methoxyprop-2-yn-1-yl)benzene (15b)

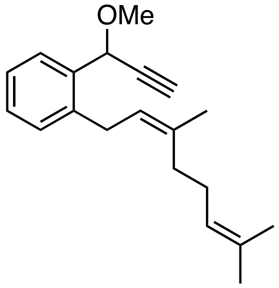

The title compound, **15b**, was prepared from **S11b**, following the same procedure as for the (*E*)-isomer, in 96% yield.

^{1}H NMR (400 MHz, $CDCl_3$) δ = 7.67 (dd, *J* = 7.2, 1.8 Hz, 1H), 7.33 – 7.21 (m, 3H), 5.34 – 5.26 (m, 2H), 5.21 – 5.15 (m, 1H), 3.52 – 3.48 (m, 2H), 3.47 (s, 3H), 2.66 (d, *J* = 2.2 Hz, 1H), 2.23 – 2.13 (m, 4H), 1.78 (s, 3H), 1.72 (s, 3H), 1.65 (s, 3H) ppm.

^{13}C NMR (101 MHz, $CDCl_3$) δ = 140.0, 136.7, 135.8, 131.9, 129.7, 128.8, 127.8, 126.3, 124.3, 123.4, 81.5, 75.7, 70.3, 56.2, 32.2, 30.8, 26.6, 25.9, 23.5, 17.8 ppm.

HRMS (ESI) *m/z* calc. for $C_{20}H_{26}ONa$ $[M+Na]^+$: 305.1881, found: 305.1877.

(*E*)-1-(2-(3,7-dimethylocta-2,6-dien-1-yl)phenyl)-3-(trimethylsilyl)prop-2-yn-1-ol (S12)

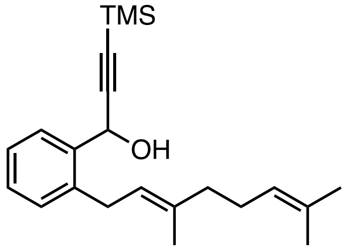

In a flame and dried 10 mL round-bottom flask ethynyltrimethylsilane (97 µl, 701 µmol, 1.7 equiv) was dissolved in dry THF (3 mL) and cooled to -78 °C under an atmosphere of argon. Then, nBuLi (2.5 M in hexanes, 0.27 mL, 619 µmol, 1.5 equiv) was slowly added and the mixture was stirred for 1 h. A solution of **S10a** (100 mg, 413 µmol, 1 equiv) in THF (1 mL) was added dropwise. After 2 h at -78 ºC the reaction was quenched with sat. NH_4Cl (3 mL). The layers were separated and the aqueous phase was extracted with EtOAc (2 x 3 mL). The combined organic phases were washed with brine, dried over $MgSO_4$ and the solvent was removed under reduced pressure. The crude material was purified by column chromatography on silica with cyclohexane/EtOAc 80:1 to afford **S12** (102 mg, 299 µmol, 73 % yield) as a yellow oil.

^{1}H NMR (500 MHz, $CDCl_3$) δ 7.71 (dd, *J* = 7.2, 1.9 Hz, 1H), 7.32 – 7.22 (m, 3H), 5.67 (d, *J* = 5.8 Hz, 1H), 5.31 (tq, *J* = 7.0, 1.3 Hz, 1H), 5.12 (tdd, *J* = 5.6, 2.8, 1.4 Hz, 1H), 3.56 (qd, *J* = 16.0, 7.1 Hz, 2H), 2.19 – 2.05 (m, 5H), 1.77 (q, *J* = 1.0 Hz, 3H), 1.71 (d, *J* = 1.3 Hz, 3H), 1.62 (d, *J* = 1.3 Hz, 3H), 0.22 (s, 9H).

^{13}C NMR (101 MHz, $CDCl_3$) δ 139.6, 138.0, 136.8, 131.6, 129.7, 128.6, 127.0, 126.4, 124.1, 122.8, 105.0, 91.4, 62.6, 39.7, 31.1, 26.6, 25.7, 17.7, 16.3, -0.2.

HRMS (ESI) *m/z* calc. for $C_{22}H_{32}NaOSi$ $[M+Na]^+$: 363.2115; found: 363.2112.

(*E*)-1-(2-(3,7-dimethylocta-2,6-dien-1-yl)phenyl)-3-(trimethylsilyl)prop-2-yn-1-one (S13)

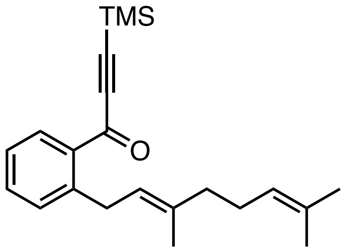

In a 50 mL round-bottom flask, **S12** (523 mg, 1.38 mmol, 1 equiv) was dissolved in CH_2Cl_2 (12 mL). Dess-Martin periodinane (720 mg, 1.70 mmol, 1.2 equiv) was added in portions at 0 °C. The reaction was stirred at 0 °C for 1 h when TLC indicated full conversion of the starting material. A saturated solution of sodium thiosulfate (10 mL) and sodium bicarbonate (10 mL) was added and the mixture was stirred for 1 h at 23º C. The aqueous layer was extracted with CH_2Cl_2 (3 x 15 mL) and the combined organic layers were dried over Na_2SO_4 and concentrated under reduced pressure. The crude product was purified by column chromatography on silica with cyclohexane/EtOAc 98:2 to yield **S13** (390 mg, 1.15 mmol, 83 % yield) as a pale-yellow oil.

^{1}H NMR (400 MHz, $CDCl_3$) δ 8.24 – 8.18 (m, 1H), 7.52 – 7.45 (m, 1H), 7.38 – 7.31 (m, 2H), 5.35 – 5.27 (m, 1H), 5.15 – 5.07 (m, 1H), 3.78 (d, *J* = 7.2 Hz, 2H), 2.18 – 2.01 (m, 4H), 1.73 – 1.71 (m, 3H), 1.69 (d, *J* = 1.3 Hz, 3H), 1.62 – 1.59 (m, 3H), 0.32 (s, 9H).
^{13}C NMR (101 MHz, $CDCl_3$) δ 179.5, 143.9, 136.8, 135.2, 133.3, 133.0, 131.4, 130.5, 125.8, 124.3, 122.3, 102.5, 99.0, 39.8, 32.3, 26.1, 25.7, 17.7, 16.2, -0.7.
HRMS (ESI) *m/z* calc. for $C_{22}H_{30}NaOSi$ $[M+Na]^+$: 361.1958; found: 361.1954.

(*E*)-2-(2-(3,7-dimethylocta-2,6-dien-1-yl)phenyl)-4-(trimethylsilyl)but-3-yn-2-ol (S14)

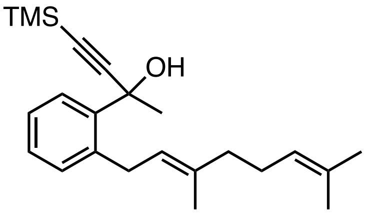

To a solution of **S13** (357 mg, 1.05 mmol, 1 equiv) in dry THF (20 mL) under an atmosphere of argon, was added methylmagnesium bromide (3 M in Et_2O, 0.75 mL, 2.25 mmol, 2 equiv) dropwise at 0 °C and the mixture was stirred for 1 h. The reaction was quenched by addition of 20 mL of a saturated solution of NH_4Cl and the layers were separated. The aqueous layer was extracted with Et_2O (3 x 15 mL) and the combined organic phases were dried over Na_2SO_4. The solvent was removed under reduced pressure affording crude **S14** (355 mg, 1.00 mmol, 95 % yield) which was used in the next step without further purification.

^{1}H NMR (500 MHz, $CDCl_3$) δ 7.71 (dt, *J* = 7.8, 1.0 Hz, 1H), 7.28 – 7.25 (m, 2H), 7.25 – 7.20 (m, 1H), 5.32 (ddt, *J* = 6.9, 5.6, 1.3 Hz, 1H), 5.15 – 5.09 (m, 1H), 3.92 – 3.72 (m, 2H), 2.50 (s, 1H), 2.18 – 2.02 (m, 4H), 1.86 (s, 3H), 1.78 (s, 3H), 1.70 (s, 5H), 1.62 (s, 4H), 0.20 (s, 9H).
^{13}C NMR (101 MHz, $CDCl_3$) δ 141.8, 139.6, 136.0, 131.5, 131.1, 127.8, 125.7, 124.8, 124.4, 124.2, 109.5, 88.8, 69.6, 39.7, 31.8, 31.5, 26.6, 25.7, 17.7, 16.3, -0.2.
HRMS (ESI) *m/z* calc. for $C_{23}H_{34}NaOSi$ $[M+Na]^+$: 377.2271; found: 377.2268.

(*E*)-2-(2-(3,7-dimethylocta-2,6-dien-1-yl)phenyl)but-3-yn-2-ol (S15)

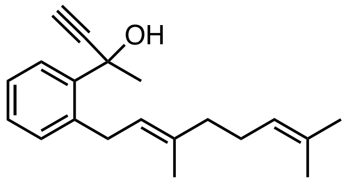

To a solution of **S14** (294 mg, 829 µmol, 1 equiv) in MeOH (1 mL) was added potassium carbonate (300 mg, 2.17 mmol, 2.6 equiv) and the mixture was stirred for 30 min at 23 ºC. Water (10 mL) was added and the aqueous layer was extracted with CH_2Cl_2 (3 x 10 mL). The combined organic layers were dried over Na_2SO_4 and concentrated under reduced pressure. The crude product was purified by column chromatography on silica with cyclohexane/EtOAc 80:1 to 80:2 affording **S15** (157 mg, 556 µmol, 67 % yield) as a yellow oil.

^{1}H NMR (500 MHz, $CDCl_3$) δ 7.71 (dt, *J* = 7.8, 1.0 Hz, 1H), 7.28 (dd, *J* = 3.9, 1.0 Hz, 2H), 7.25 – 7.20 (m, 1H), 5.34 – 5.30 (m, 1H), 5.14 – 5.09 (m, 1H), 3.91 – 3.71 (m, 2H), 2.69 (s, 1H), 2.55 (s, 1H), 2.17 – 2.05 (m, 4H), 1.91 (s, 3H), 1.78 (s, 3H), 1.70 (s, 3H), 1.62 (s, 3H).
^{13}C NMR (126 MHz, $CDCl_3$) δ 141.3, 139.7, 136.2, 131.5, 131.3, 128.1, 125.8, 125.1, 124.2 (2C), 88.0, 72.9, 69.6, 39.7, 31.9, 31.5, 26.6, 25.7, 17.7, 16.4.
HRMS (ESI) *m/z* calc. for $C_{20}H_{26}NaO$ $[M+Na]^+$: 305.1876; found: 305.1870.

(*E*)-1-(3,7-dimethylocta-2,6-dien-1-yl)-2-(2-methoxybut-3-yn-2-yl)benzene (16)

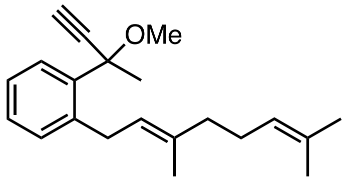

To a solution of **S15** (142 mg, 503 µmol, 1 equiv) in dry THF (10 mL) was added sodium hydride (60 % dispersion in mineral oil, 24 mg, 60.3 µmol, 1.2 equiv) and dimethyl sulfate (57 µl, 603 µmol, 1.2 equiv) at 0 ºC and the mixture was stirred for 5 h at 23 ºC. After adding sat. NH_4Cl (10 mL), the aqueous phase was extracted with diethyl ether (3 x 10 mL) and the combined organic layers were dried over Na_2SO_4. The solvent was removed under reduced pressure and the residue was purified by column chromatography on silica with cyclohexane/EtOAc 80:1 affording **16** (135 mg, 455 µmol, 91 % yield) as a colorless oil.

^{1}H NMR (500 MHz, $CDCl_3$) δ 7.78 (dt, *J* = 7.8, 1.0 Hz, 1H), 7.29 – 7.27 (m, 2H), 7.17 – 7.22 (m, 1H), 5.28 – 5.34 (m, 1H), 5.11 – 5.16 (m,1H), 3.83 – 3.67 (m, 2H), 3.29 (s, 3H), 2.76 (s, 1H), 2.19 – 2.06 (m, 4H), 1.86 (s, 3H), 1.76 (d, *J* = 1.1 Hz, 3H), 1.71 (d, *J* = 1.3 Hz, 3H), 1.63 (s, 3H).
^{13}C NMR (126 MHz, $CDCl_3$) δ 140.1, 138.2, 135.6, 131.4, 131.2, 128.0, 127.8, 125.5, 124.3, 124.2, 84.9, 77.7, 75.2, 52.3, 39.7, 30.9, 30.2, 26.6, 25.7, 17.7, 16.2.
HRMS (ESI) *m/z* calc. for $C_{21}H_{28}NaO$ $[M+Na]^+$: 319.2032; found: 319.2023.

1.3 General procedure for gold(I)-catalyzed cyclization:

To a solution of the cyclization precursor in CH_2Cl_2 (0.05 M) was added [(JohnPhos)Au(MeCN)]SbF_6 (2 mol%). The reaction was followed by TLC and GCMS analysis and quenched by addition of few drops of Et_3N. The crude material was concentrated under reduced pressure and purified by preparative TLC.

1.3.1 Characterization of cyclization products 5a, 11-14, 17-18, 20 and 22

(4b*S*,4b^1*S*,6a*S*,8a*S*,9a*S*)-6a,9,9-trimethyl-4b,4b^1,6a,7,8,8a,9,9a-octahydro-5*H*-benzo[*g*]cyclopropa[4,5]cyclohepta[1,2,3-*cd*]isobenzofuran (11)

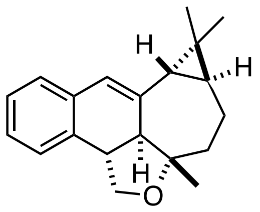

45% yield as a single diastereomer.
^{1}H NMR (400 MHz, $CDCl_3$) δ = 7.21 – 7.09 (m, 3H), 6.93 (d, *J* = 7.2 Hz, 1H), 6.20 (t, *J* = 2.4 Hz, 1H), 4.33 (t, *J* = 7.7 Hz, 1H), 3.99 (dd, *J* = 10.0, 7.5 Hz, 1H), 3.54 (dt, *J* = 17.5, 9.0 Hz, 1H), 2.66 (dt, *J* = 17.0, 2.5 Hz, 1H), 2.30 (ddd, *J* = 13.0, 4.1, 2.1 Hz, 1H), 2.14 (ddt, *J* = 12.9, 4.4, 2.0 Hz, 1H), 1.70 (td, *J* = 12.9, 4.8 Hz, 1H), 1.39 (d, *J* = 0.9 Hz, 3H), 1.28 – 1.23 (m, 1H), 1.20 (s, 3H), 1.18 (s, 3H), 0.97 (dt, *J* = 8.0, 2.2 Hz, 1H), 0.11 (ddd, *J* = 11.8, 8.0, 2.0 Hz, 1H) ppm.
^{13}C NMR (101 MHz, $CDCl_3$) δ = 140.1, 136.4, 136.2, 126.7, 126.7, 126.6, 124.8, 121.6, 84.3, 67.1, 58.5, 44.9, 42.7, 35.7, 34.8, 30.9, 26.5, 23.3, 22.5, 22.1 ppm.
HRMS (APCI) *m/z* calc. for $C_{20}H_{25}O$ $[M+H]^+$: 281.1900, found: 281.1892.

(4b*S*,4b^1*S*,6a*S*,8a*S*,9a*S*)-6a,9,9-trimethyl-4b,4b^1,6a,7,8,8a,9,9a-octahydro-5*H*-benzo[*g*]cyclopropa[4,5]cyclohepta[1,2,3-*cd*]isobenzofuran-5-one (5a)

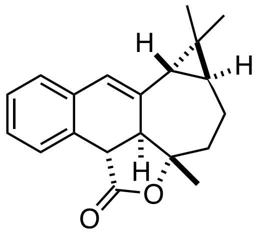

22% yield as a single diastereomer.
^{1}H NMR (400 MHz, $CDCl_3$) δ = 8.12 – 8.04 (m, 1H), 7.27 – 7.20 (m, 2H), 7.15 – 7.08 (m, 1H), 6.24 (t, *J* = 2.5 Hz, 1H), 3.82 (dd, *J* = 18.9, 1.1 Hz, 1H), 3.18 (dt, *J* = 18.9, 2.5 Hz, 1H), 2.46 (ddd, *J* = 13.1, 4.2, 2.1 Hz, 1H), 2.27 (ddt, *J* = 13.2, 4.9, 2.0 Hz, 1H), 1.94 – 1.81 (m, 1H), 1.64 (d, *J* = 0.9 Hz, 3H), 1.40 – 1.34 (m, 1H), 1.21 (s, 3H), 1.20 (s, 3H), 0.99 (dt, *J* = 8.0, 2.2 Hz, 1H), 0.18 (ddd, *J* = 11.9, 8.0, 2.0 Hz, 1H) ppm.
^{13}C NMR (101 MHz, $CDCl_3$) δ = 173.3, 137.8, 135.8, 130.5, 127.5, 127.3, 127.1, 124.8, 122.8, 88.1, 57.0, 43.3, 41.1, 35.5, 34.8, 32.2, 26.5, 23.2, 22.0, 19.6 ppm.
HRMS (ESI) *m/z* calc. for $C_{20}H_{22}O_2Na$ $[M+Na]^+$: 317.1512, found: 317.1511.

(4b*S*,4b1*S*,6a*R*,8a*S*,9a*R*)-6a,9,9-trimethyl-4b,4b1,6a,7,8,8a,9,9a-octahydro-5*H*-benzo[*g*]cyclopropa[4,5]cyclohepta[1,2,3-*cd*]isobenzofuran (14) and (4b*S*,4b1*S*,6a*R*,8a*R*,9a*R*)-6a,9,9-trimethyl-4b,4b1,6a,7,8,8a,9,9a-octahydro-5*H*-benzo[*g*]cyclopropa[4,5]cyclohepta[1,2,3-*cd*]isobenzofuran (13)

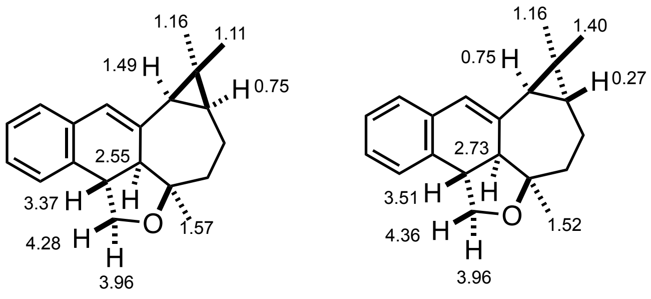

major isomer
14

minor isomer
13

38% yield, 1.4:1 mixture of diastereomers.
^{1}H NMR (400 MHz, $CDCl_3$) δ = 7.24 – 7.01 (m, 3H **14**, 3H **13**), 6.92 (dt, *J* = 7.4, 1.3 Hz, 1H **14**), 6.88 (dt, *J* = 7.4, 1.3 Hz, 1H **13**), 6.27 (t, *J* = 2.7 Hz, 1H **14**), 5.98 (t, *J* = 2.0 Hz, 1H **13**), 4.36 (t, *J* = 7.4 Hz, 1H **13**), 4.29 (t, *J* = 7.5 Hz, 1H **14**), 4.02 – 3.92 (m, 1H **14**, 1H **13**), 3.51 (ddd, *J* = 17.3, 10.5, 7.3 Hz, 1H **13**), 3.37 (ddd, *J* = 17.4, 10.2, 7.7 Hz, 1H **14**), 2.73 (dt, *J* = 16.6, 2.2 Hz, 1H **13**), 2.55 (dt, *J* = 16.6, 2.5 Hz, 1H **14**), 2.09 – 1.75 (m, 3H **14**, 3H **13**), 1.57 (s, 3H **14**), 1.52 (s, 3H **13**), 1.52 – 1.48 (m, 1H **14**), 1.46 – 1.41 (m, 1H **14**), 1.40 (s, 3H **13**), 1.33 – 1.25 (m, 1H **13**), 1.16 (s, 3H **14**, 3H **13**), 1.11 (s, 3H **14**), 0.78 – 0.72 (m, 1H **14**, 1H **13**), 0.27 (ddd, *J* = 11.9, 7.4, 2.2 Hz, 1H **13**) ppm.
^{13}C NMR (101 MHz, $CDCl_3$) δ = 145.7, 138.3, 136.3, 136.2, 136.0, 135.8, 128.8, 127.0, 126.8, 126.7, 126.0, 125.9, 125.8, 124.5, 124.3, 113.6, 85.2, 81.8, 68.2, 67.3, 57.9, 55.3, 45.5, 45.4, 41.2, 40.5, 36.8, 34.4, 30.5, 30.2, 29.8, 28.9, 28.8, 26.8, 24.8, 23.6, 22.4, 20.5, 20.4, 17.0 ppm.
HRMS (APCI) *m/z* calc. for $C_{20}H_{25}O$ $[M+H]^+$: 281.1900, found: 281.1898.

(*E*)-2-(2,6-dimethylhepta-1,5-dien-1-yl)naphthalene (12)

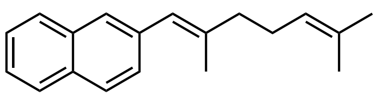

^{1}H NMR (500 MHz, $CDCl_3$) δ = 7.82 – 7.76 (m, 3H), 7.67 (s, 1H), 7.46 – 7.40 (m, 2H), 7.38 (dd, *J* = 8.4, 1.7 Hz, 1H), 6.42 (s, 1H), 5.24 – 5.17 (m, 1H), 2.27 – 2.23 (m, 4H), 1.95 (d, *J* = 1.4 Hz, 3H), 1.73 (s, 3H), 1.66 (s, 3H) ppm.
^{13}C NMR (126 MHz, $CDCl_3$) δ 139.7, 136.4, 133.6, 132.0, 127.9, 127.8, 127.7, 127.5, 127.3, 126.0, 125.5, 125.1, 124.1, 41.0, 27.0, 25.9, 18.2, 17.9 ppm.
HRMS (APCI) *m/z* calc. for $C_{19}H_{23}$ $[M+H]^+$: 251.1794, found: 251.1789.

(*Z*)-2-(2,6-dimethylhepta-1,5-dien-1-yl)naphthalene (12')

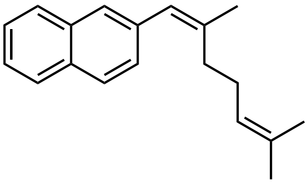

^{1}H NMR (400 MHz, $CDCl_3$) δ = 7.82 – 7.74 (m, 3H), 7.65 (s, 1H), 7.47 – 7.39 (m, 2H), 7.35 (dd, *J* = 8.5, 1.8 Hz, 1H), 6.44 (s, 1H), 5.17 – 5.10 (m, 1H), 2.36 – 2.29 (m, 2H), 2.28 – 2.19 (m, 2H), 1.95 (d, *J* = 1.5 Hz, 3H), 1.69 (d, *J* = 1.4 Hz, 3H), 1.63 (s, 3H) ppm.
^{13}C NMR (101 MHz, $CDCl_3$) δ = 140.0, 133.6, 132.0, 132.0, 129.5, 127.9, 127.7, 127.6, 127.6, 127.0, 126.0, 125.9, 125.5, 124.2, 32.9, 26.9, 25.9, 24.3, 17.9 ppm.

HRMS (APCI) *m/z* calc. for $C_{19}H_{23}$ $[M+H]^+$: 251.1794, found: 251.1791.

(1a*S*,4*R*,4a*S*,10b*R*)-4-methoxy-1,1,4-trimethyl-1,1a,2,3,4,4a,5,10b-octahydrocyclopropa[3,4]cyclohepta[1,2-*b*]naphthalene (17a)

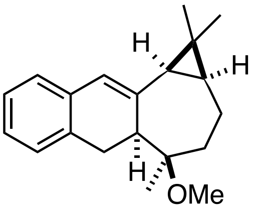

^{1}H NMR (500 MHz, $CDCl_3$) δ = 7.07 – 6.97 (m, 3H), 6.91 (d, *J* = 7.1 Hz, 1H), 6.32 (d, *J* = 2.2 Hz, 1H), 3.03 – 2.93 (m, 2H), 2.75 (s, 3H), 2.18 (bs, 1H), 1.93 (ddd, *J* = 14.5, 6.6, 1.5 Hz, 1H), 1.62 – 1.55 (m, 1H), 1.40 – 1.36 (m, 1H), 1.35 – 1.28 (m, 1H), 1.27 (s, 3H), 1.24 (s, 3H), 1.22 – 1.12 (m, 1H), 1.12 (s, 3H), 0.75 (ddd, *J* = 11.3, 9.4, 5.4 Hz, 1H) ppm.
^{13}C NMR (101 MHz, $CDCl_3$) δ = 137.8, 136.1, 135.3, 128.6, 126.0, 125.9, 125.5, 124.7, 78.1, 48.7 (2C), 37.5, 33.0, 31.3, 29.1, 28.4, 24.9, 23.0, 19.5, 18.1 ppm.
HRMS (ESI) *m/z* calc. for $C_{20}H_{26}ONa$ $[M+Na]^+$: 305.1876, found: 305.1861.

(1a*S*,4*R*,4a*S*,10b*R*)-4-methoxy-1,1,4,10-tetramethyl-1,1a,2,3,4,4a,5,10b-octahydrocyclopropa[3,4]cyclohepta[1,2-*b*]naphthalene (17b)

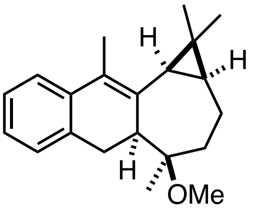

^{1}H NMR (400 MHz, $CDCl_3$) δ 7.22 (d, *J* = 7.7 Hz, 1H), 7.17 (td, *J* = 7.6, 7.0, 2.3 Hz, 1H), 7.13 – 7.05 (m, 2H), 2.93 (dd, *J* = 15.2, 6.5 Hz, 1H), 2.85 (s, 3H), 2.83 – 2.75 (m, 1H), 2.42 (br s, 1H), 2.09 (t, *J* = 1.6 Hz, 3H), 1.87 (ddd, *J* = 14.0, 5.4, 3.2 Hz, 1H), 1.79 – 1.67 (m, 1H), 1.55 – 1.48 (m, 1H), 1.44 (d, *J* = 9.6 Hz, 1H), 1.38 (s, 3H), 1.34 – 1.23 (m, 1H), 1.21 (s, 3H), 1.08 (s, 3H), 0.77 (ddd, *J* = 10.7, 9.1, 5.3 Hz, 1H).

^{13}C NMR (101 MHz, $CDCl_3$) δ 137.9, 137.2, 133.4, 132.7, 126.0, 125.7, 125.6, 122.3, 77.8, 48.4, 36.6, 32.4, 31.2, 28.6, 25.5, 23.0, 20.8, 17.3, 16.9.
HRMS (ESI) *m/z* calc. for $C_{21}H_{28}NaO$ $[M+Na]^+$: 319.2032; found: 319.2038.

(1a*S*,4*S*,4a*S*,10b*R*)-4-methoxy-1,1,4-trimethyl-1,1a,2,3,4,4a,5,10b-octahydrocyclopropa[3,4]cyclohepta[1,2-*b*]naphthalene (18)

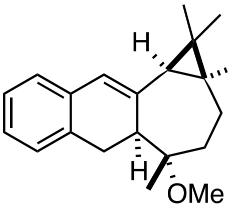

^{1}H NMR (500 MHz, $CDCl^3$) δ = 7.11 – 7.04 (m, 3H), 6.94 – 6.89 (m, 1H), 6.35 (d, *J* = 2.1 Hz, 1H), 3.25 (s, 3H), 3.22 (d, *J* = 16.4 Hz, 1H), 2.96 (dd, *J* = 16.4, 9.1 Hz, 1H), 2.54 (d, *J* = 9.0 Hz, 1H), 1.86 – 1.80 (m, 1H), 1.78 – 1.69 (m, 1H), 1.63 (dd, *J* = 13.0, 6.6 Hz, 1H), 1.43 – 1.36 (m, 1H), 1.19 (s, 3H), 1.13 (s, 3H), 0.87 – 0.81 (m, 2H), 0.75 (s, 3H) ppm.
^{13}C NMR (101 MHz, $CDCl_3$) δ = 137.4, 135.5, 134.4, 129.1, 127.3, 127.1, 126.0, 125.3, 80.1, 48.6, 45.0, 38.8, 32.6, 29.0, 28.9, 28.7, 21.5, 20.1, 19.7, 18.2 ppm.
HRMS (APCI) *m/z* calc. for $C_{19}H_{23}$ $[M\text{-}MeO]^+$: 251.1794, found: 251.1793.

***tert*-butyl(((1a*S*,4*R*,4a*S*,5*S*,10b*R*)-4-methoxy-1,1,4-trimethyl-1,1a,2,3,4,4a,5,10b-octahydrocyclopropa[3,4]cyclohepta[1,2-*b*]naphthalen-5-yl)methoxy)dimethylsilane (20)**

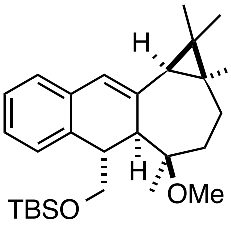

37% yield as a single diastereomer.
^{1}H NMR (500 MHz, $CDCl_3$) δ = 7.09 – 6.99 (m, 3H), 6.92 (dd, *J* = 6.7, 1.4 Hz, 1H), 6.27 (d, *J* = 2.2 Hz, 1H), 3.46 (d, *J* = 7.5 Hz, 2H), 3.19 (t, *J* = 7.6 Hz, 1H), 2.73 (s, 3H), 2.38 (s, 1H), 2.00 (dd, *J* = 14.6, 7.1 Hz, 1H), 1.61 – 1.55 (m, 1H), 1.35 (dd, *J* = 14.6, 11.6 Hz, 1H), 1.26 (s, 4H), 1.24 (s, 3H), 1.18 (dt, *J* = 14.4, 11.5 Hz, 1H), 1.12 (s, 3H), 0.89 (s, 9H), 0.78 (ddd, *J* = 11.2, 9.5, 5.6 Hz, 1H), -0.02 (s, 3H), -0.04 (s, 3H) ppm.
^{13}C NMR (126 MHz, $CDCl_3$) δ = 136.5, 135.2, 134.6, 127.8, 127.6, 126.1, 126.0, 125.2, 78.4, 66.2, 50.7, 49.0, 43.7, 38.1, 33.7, 29.1, 28.7, 26.1, 24.8, 23.0, 19.3, 18.5, 18.3, -5.1, -5.4 ppm.
HRMS (ESI) *m/z* calc. for $C_{27}H_{42}O_2SiNa$ $[M+Na]^+$: 449.2846, found: 449.2834.

***tert*-butyl(((1a*S*,4*S*,4a*S*,5*S*,10b*R*)-4-methoxy-1,1,4-trimethyl-1,1a,2,3,4,4a,5,10b-octahydrocyclopropa[3,4]cyclohepta[1,2-*b*]naphthalen-5-yl)methoxy)dimethylsilane (22)**

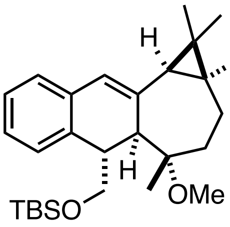

38% yield as a single diastereomer.
^{1}H NMR (500 MHz, $CDCl_3$) δ = 7.14 – 7.07 (m, 3H), 6.96 – 6.91 (m, 1H), 6.30 (d, J = 2.2 Hz, 1H), 3.53 – 3.43 (m, 2H), 3.35 (dd, J = 9.6, 6.0 Hz, 1H), 3.32 (s, 3H), 2.83 (s, 1H), 1.98 – 1.91 (m, 1H), 1.88 – 1.80 (m, 1H), 1.51 (dd, J = 12.9, 7.1 Hz, 1H), 1.30 – 1.24 (m, 1H), 1.20 (s, 3H), 1.13 (s, 3H), 0.88 (s, 9H), 0.89 – 0.82 (m, 2H), 0.71 (s, 3H), -0.03 (s, 3H), -0.03 (s, 3H) ppm.
^{13}C NMR (101 MHz, $CDCl_3$) δ = 136.0, 135.7, 134.0, 128.8, 128.2, 127.1, 126.7, 125.6, 79.6, 66.3, 48.3, 45.3, 41.2, 38.9, 33.0, 29.0, 28.7, 26.1, 21.4, 21.4, 20.1, 18.6, 18.2, -5.2, -5.5 ppm.
HRMS (ESI) *m/z* calc. for $C_{27}H_{42}O_2SiNa$ $[M+Na]^+$: 449.2846, found: 449.2848.

1.3.2 General procedure for TBS-deprotection of cyclization products 20 and 22

To a solution of TBS-protected substrate (1 equiv.) in THF (0.1M) was added HCl (10% solution in H_2O, same volume as THF). The mixture was stirred for 1-2 h at 23 ºC. The reaction was quenched by sat. $NaHCO_3$. The aqueous phase was extracted with EtOAc (x2) and the combined organic layers were washed with brine, dried over $MgSO_4$ and concentrated. The crude material was purified by column chromatography on silica.

((1a*S*,4*R*,4a*S*,5*S*,10b*R*)-4-methoxy-1,1,4-trimethyl-1,1a,2,3,4,4a,5,10b-octahydrocyclopropa[3,4]cyclohepta[1,2-*b*]naphthalen-5-yl)methanol (21)

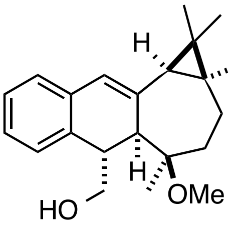

^{1}H NMR (400 MHz, $CDCl_3$) δ = 7.12 – 7.00 (m, 3H), 6.95 (dd, J = 7.1, 1.5 Hz, 1H), 6.28 (d, J = 2.2 Hz, 1H), 3.63 – 3.45 (m, 2H), 3.17 (t, J = 7.5 Hz, 1H), 2.72 (s, 3H), 2.17 (s, 1H), 2.00 (dd, J = 14.7, 7.1 Hz, 1H), 1.62 – 1.57 (m, 1H), 1.36 – 1.31 (m, 2H), 1.26 (s, 3H), 1.25 (s, 4H), 1.21 – 1.14 (m, 1H), 1.11 (s, 3H), 0.78 (ddd, J = 11.2, 9.5, 5.5 Hz, 1H) ppm.
^{13}C NMR (101 MHz, $CDCl_3$) δ = 136.0, 135.5, 134.5, 127.6, 127.4, 126.5, 126.2, 125.5, 78.2, 66.5, 51.8, 48.9, 43.5, 37.8, 33.7, 29.0, 28.7, 24.8, 23.2, 19.2, 18.2 ppm.
HRMS (ESI) *m/z* calc. for $C_{21}H_{28}O_2Na$ $[M+Na]^+$: 335.1982, found: 335.1988.

((1a*S*,4*S*,4a*S*,5*S*,10b*R*)-4-methoxy-1,1,4-trimethyl-1,1a,2,3,4,4a,5,10b-octahydrocyclopropa[3,4]cyclohepta[1,2-*b*]naphthalen-5-yl)methanol (23)

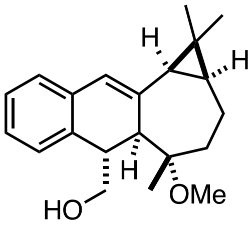

^{1}H NMR (500 MHz, $CDCl_3$) δ 7.17 – 7.09 (m, 3H), 7.02 – 6.92 (m, 1H), 6.31 (d, *J* = 2.3 Hz, 1H), 3.63 – 3.57 (m, 1H), 3.55 – 3.49 (m, 1H), 3.38 (t, *J* = 7.5 Hz, 1H), 3.28 (s, 3H), 2.61 (s, 1H), 1.88 – 1.76 (m, 2H), 1.66 (dd, *J* = 12.9, 7.2 Hz, 1H), 1.43 – 1.29 (m, 3H), 1.20 (s, 3H), 1.13 (s, 3H), 0.91 – 0.77 (m, 1H, together with ^{1}H-grease), 0.72 (s, 3H).
^{13}C NMR (126 MHz, $CDCl_3$) δ 136.0, 135.5, 134.0, 128.6, 128.2, 127.3, 127.0, 126.0, 79.6, 66.8, 48.7, 47.7, 41.2, 38.9, 33.0, 28.9, 28.7, 21.6, 20.1, 20.1, 18.2.
HRMS (ESI) *m/z* calc. for $C_{21}H_{28}O_2Na$ $[M+Na]^+$: 335.1982, found: 335.1988.

1.4 Control experiments

1.4.1 Parallel reactions with diastereomerically pure 10b

The two diastereomers of **10b** were separated by collection of the fractions from chiral HPLC: column: Chiralpak IC 250x4.6mm, 5µm, method: Hex / DCM / IPA 84:15:1, flow: 1mL/min, sample: 1 mg/mL in mobile phase.

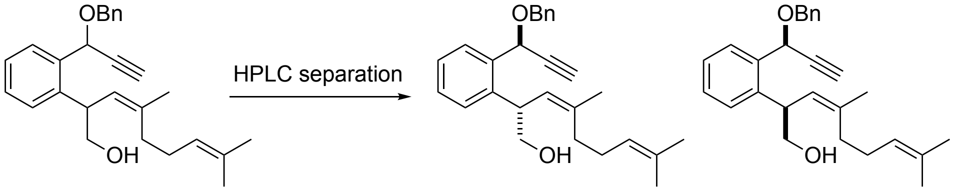


```
=====================================================================
Acq. Operator   : SYSTEM                          Seq. Line :    6
Acq. Instrument : NP HPLC                          Location :    5
Injection Date  : 2/7/2020 8:59:53 PM                   Inj :    1
                                                Inj Volume : 5.000 µl
Acq. Method     : C:\Chem32\1\Methods\Chiral IC_Hex-DCM-IPA.M
Last changed    : 2/7/2020 5:51:43 PM by SYSTEM
Analysis Method : C:\Chem32\1\Methods\Chiral IC_Hex-DCM-IPA.M
Last changed    : 2/10/2020 4:45:56 PM by SYSTEM
                  (modified after loading)
Sample Info     : Chiralpak IC 250x4.6mm, 5µm
                  Hex / DCM / IPA 84:15:1
                  1mL/min
                  Sample: 1 mg/ml in mobile phase (one bottle)
```

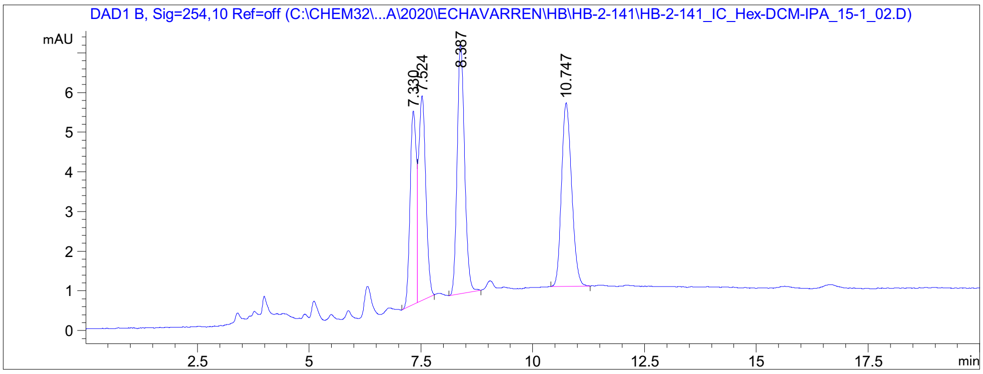


```
=====================================================================
                         Area Percent Report
=====================================================================

Sorted By             :      Signal
Multiplier            :      1.0000
Dilution              :      1.0000
Do not use Multiplier & Dilution Factor with ISTDs


Signal 1: DAD1 B, Sig=254,10 Ref=off
```

Peak #	RetTime [min]	Type	Width [min]	Area [mAU*s]	Height [mAU]	Area %
1	7.330	BV	0.1492	48.15791	4.87785	19.1503
2	7.524	VB	0.1652	57.17340	5.16423	22.7353
3	8.387	BB	0.1802	73.24988	6.27372	29.1282
4	10.747	BB	0.2479	72.89285	4.62499	28.9862
Totals :				251.47404	20.94079	

Collection of peaks:

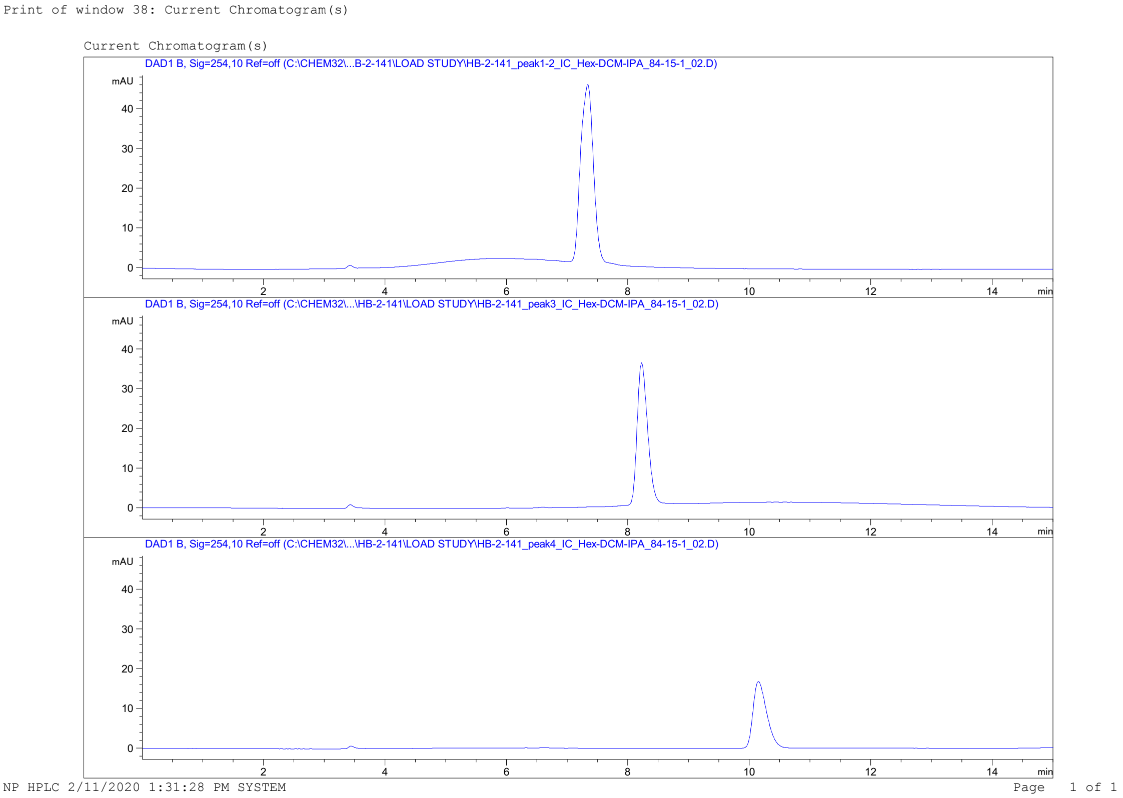

The first fraction corresponds to both enantiomers of one of the diastereomers while the two other fractions are the separated enantiomers of the other diastereomer. We could not assign by NMR the relative configuration of each diastereomer.

Control experiment with separated diastereomers:

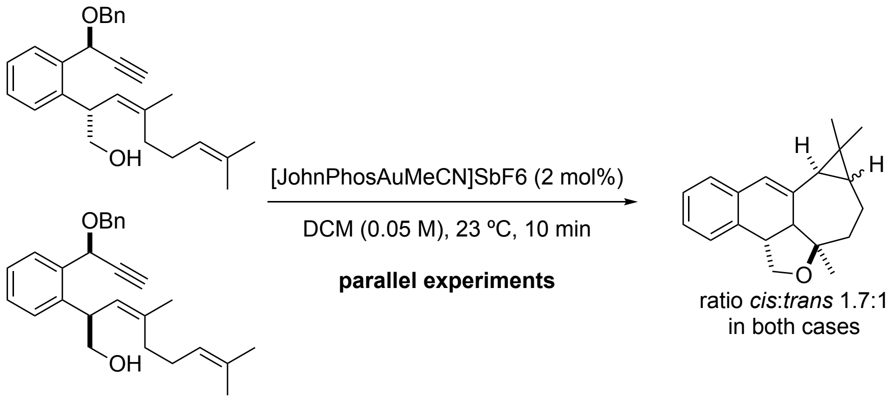

^{1}H-NMR of the crude reaction mixtures compared to the isolated products **12’** and **13+14**:

The green dots indicate the olefinic protons of the two pentacyclic products (cis/trans-fused cyclopropanes **13** and **14**) while the orange signals are form the naphthalene byproduct **12’**, which only appear in one of the crudes.

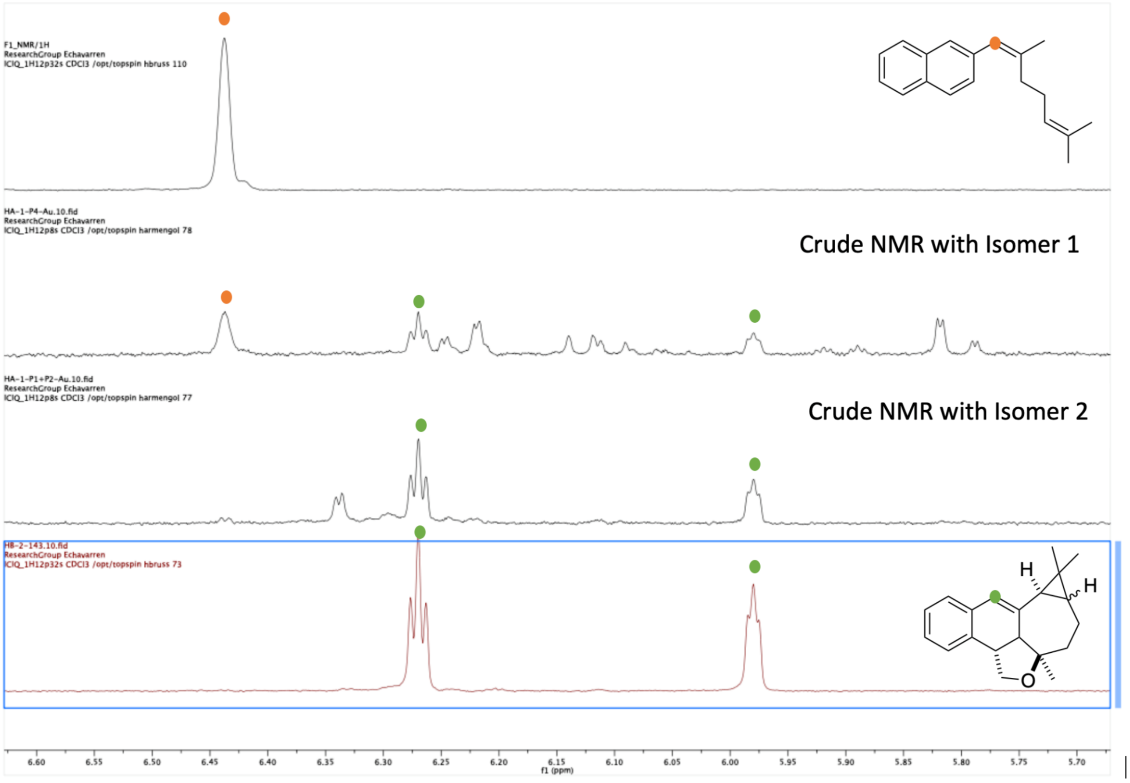

1.4.2 Synthesis and reactivity of 7,12-dien-1-enyes

To further prove the need of the internal nucleophile, we prepared a new substrate with a longer chain. Dienyne **S16** underwent decomposition under the standard reaction conditions at room temperature in the presence of Au(I) catalysts. Lowering the temperature to -20 ºC allowed the isolation of tetracyclic compound **S17** in low yield, together with naphthalene **S18** as the main product (conditions a, Scheme 7). Changing the catalyst to [IPrAuPhCN]SbF_6 with a more donating ligand improved the selectivity towards desired tetracyclic compound **S17** (conditions b).

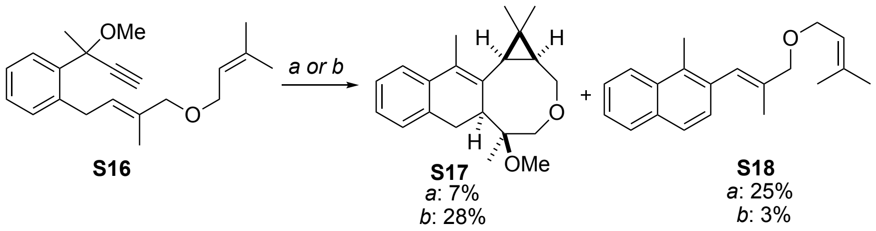

a. [(JohnPhos)Au(MeCN)]SbF_6 (5 mol%), CH_2Cl_2, -20 ºC, 8 h; b. [(IPr)Au(PhCN)] SbF_6 (5 mol%), CH_2Cl_2, -20 ºC, 8 h;

The structure of **S17** and its relative configuration was assigned by NOE experiments as a *cis*-fused cyclopropane. With this experiment we could prove that longer and more flexible chain and thus larger ring formation does not translate into easier formation of *trans*-fused cyclopropanes, since they were not obtained in this case.

2-(2-bromophenyl)-4-(trimethylsilyl)but-3-yn-2-ol (S19)

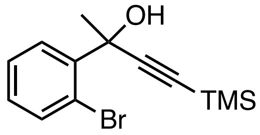

To a solution of 1-(2-bromophenyl)-3-(trimethylsilyl)prop-2-yn-1-one[2] (0.650 g, 2.31 mmol, 1 equiv) in dry THF (9 mL) at 0 ºC was added MeMgBr (1 mL, 3 M, 1.3 equiv) and the reaction was allowed to warm to 23 ºC over 12 h. The reaction was quenched by the addition of sat. NH_4Cl (10 mL). The aqueous phase was extracted with EtOAc (3 x 10 mL) and the combined organic layers were washed with brine and dried over $MgSO_4$. The solvent was removed under reduced pressure and the crude material was used in the next step without further purification.

^{1}H NMR (500 MHz, $CDCl_3$) δ 7.80 (dd, *J* = 7.9, 1.7 Hz, 1H), 7.61 (dd, *J* = 7.9, 1.3 Hz, 1H), 7.33 (ddd, *J* = 7.9, 7.3, 1.3 Hz, 1H), 7.15 (ddd, *J* = 7.9, 7.3, 1.7 Hz, 1H), 3.03 (br s, 1H), 1.93 (s, 3H), 0.18 (s, 9H).
^{13}C NMR (126 MHz, $CDCl_3$) δ 142.7, 135.1, 129.3, 127.6, 127.2, 121.2, 107.6, 89.9, 70.0, 29.9, -0.1 (3C).
HRMS (ESI) *m/z* calc. for $C_{13}H_{17}BrNaOSi$ $[M+Na]^+$: 319.0124; found: 319.0129.

(3-(2-bromophenyl)-3-methoxybut-1-yn-1-yl)trimethylsilane (S20)

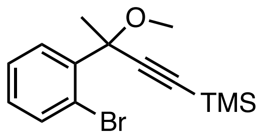

To a solution of crude **S19** (0.687 g, 2.31 mmol, 1 equiv) in THF (23 mL) at 0 ºC was added NaH (0.120 g, 60% in mineral oil, 3 mmol, 1.3 equiv). The mixture was stirred at this temperature for 30 min. Me_2SO_4 (0.3 mL, 3 mmol, 1.3 equiv) was added dropwise and the resulting mixture was stirred at 23 ºC for 2h. The reaction was quenched by addition of sat. NH_4Cl. The aqueous phase was extracted with EtOAc (2 x 50 mL) and the combined organic layers were washed with brine and dried over Na_2SO_4. The solvent was removed under reduced pressure and the residue was purified by column chromatography on silica with cyclohexane/EtOAc 100:1 to 100:2 affording **S20** (0.639 mg, 2.05 mmol, 89 % yield over 2 steps) as a colorless oil.

^{1}H NMR (500 MHz, $CDCl_3$) δ 7.85 (dd, *J* = 7.9, 1.7 Hz, 1H), 7.61 (dd, *J* = 7.9, 1.3 Hz, 1H), 7.31 (ddd, *J* = 8.0, 7.3, 1.3 Hz, 1H), 7.14 (td, *J* = 7.6, 1.7 Hz, 1H), 3.26 (s, 3H), 1.87 (s, 3H), 0.24 (s, 9H).
^{13}C NMR (75 MHz, $CDCl_3$) δ 139.6, 135.6, 130.0, 129.3, 127.3, 120.8, 105.0, 92.7, 77.9, 52.5, 28.9, 0.0 (3C).
HRMS (ESI) *m/z* calc. for $C_{14}H_{19}BrNaOSi$ $[M+Na]^+$: 333.0281; found: 333.0287.

(*E*)-3-methyl-4-((3-methylbut-2-en-1-yl)oxy)but-2-en-1-ol (S21)

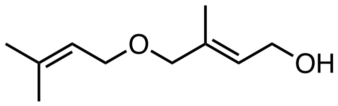

To a solution of (*E*)-4-((*tert*-butyldimethylsilyl)oxy)-2-methylbut-2-en-1-ol[3] (0.80 g, 3.70 mmol, 1 equiv) in DMF (20 mL) at 0 ºC was added NaH (0.177 g, 60% dispersion in mineral oil, 4.44 mmol, 1.2 equiv) and the mixture was stirred for 30 min. After this time prenyl bromide (0.56 mL, 4.81 mmol, 1.3 equiv) was added dropwise and the mixture was stirred at 23 ºC for 5 h. The reaction diluted with Et_2O and it was quenched by addition of sat. NH_4Cl (20 mL). The aqueous phase was extracted with Et_2O (3 x 30 mL) and the combined organic layers were washed with brine and dried over Na_2SO_4. The solvent was evaporated, and the residue was diluted in 20 mL of THF. HCl (15 mL, 10% in water) was added and the biphasic system was vigorously stirred for 2 h at 23 ºC. After this time, the two phases were separated, and the aqueous phase was extracted with EtOAc (2 x 30 mL). The combined organic layers were combined, washed with sat. $NaHCO_3$ and dried over Na_2SO_4. The solvent was evaporated under reduced pressure and the residue was purified by column chromatography on silica with cyclohexane/EtOAc 10:1 affording **S21** (0.415 g, 2.44 mmol, 66 % yield over two steps) as a colorless oil.

^{1}H NMR (500 MHz, $CDCl_3$) δ 5.66 (thex, *J* = 6.7, 1.3 Hz, 1H), 5.35 (thept, *J* = 7.0, 1.4 Hz, 1H), 4.20 (dq, *J* = 6.8, 0.9 Hz, 2H), 3.92 (dt, *J* = 6.9, 0.9 Hz, 2H), 3.85 (br s, 2H), 1.74 (br s, 3H), 1.70 (br s, 3H), 1.66 (br s, 3H), 1.47 (br s, 1H).
^{13}C NMR (126 MHz, $CDCl_3$) δ 137.2, 136.2, 126.1, 121.2, 75.4, 66.6, 59.3, 25.9, 18.2, 14.2.
HRMS (ESI) *m/z* calc. for $C_{10}H_{18}NaO_2$ $[M+Na]^+$: 193.1199; found: 193.1198.

(*E*)-4-bromo-2-methyl-1-((3-methylbut-2-en-1-yl)oxy)but-2-ene (S22)

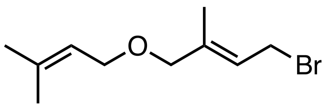

To a solution of **S21** (0.415 g, 2.44 mmol, 1 equiv) in diethylether (5 mL) at -20 ºC, was added PBr_3 (0.12 mL, 1.22 mmol, 0.5 equiv) dropwise. The mixture was stirred at this temperature for 4 h. The reacting mixture was poored into 10 mL of ice-cold water and the aqueous phase was extracted with Et_2O (3 x 15 mL). The combined organic layers were washed with sat. $NaHCO_3$ and brine and dried over $MgSO_4$. The solvent was evaporated affording **S22** (0.426 g, 1.83 mmol, 75 % yield) as a colorless oil. The crude material was used without further purification.

^{1}H NMR (400 MHz, $CDCl_3$) δ 5.79 (thex, *J* = 8.4, 1.4 Hz, 1H), 5.34 (thept, *J* = 6.9, 1.4 Hz, 1H), 4.02 (d, *J* = 8.4 Hz, 2H), 3.92 (dt, *J* = 7.0, 0.9 Hz, 2H), 3.88 (br s, 2H), 1.75 (br s, 6H), 1.67 (br s, 3H).
^{13}C NMR (101 MHz, $CDCl_3$) δ 139.8, 137.4, 122.4, 121.1, 74.7, 66.6, 28.3, 26.0, 18.2, 13.8.
HRMS (ESI) *m/z* calc. for $C_{10}H_{18}BrO$ $[M+H]^+$: 233.0536; found: 233.0537.

(*E*)-1-(2-methoxybut-3-yn-2-yl)-2-(3-methyl-4-((3-methylbut-2-en-1-yl)oxy)but-2-en-1-yl)benzene (S16)

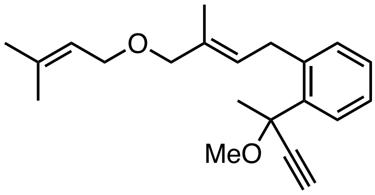

To a solution of **S20** (200 mg, 642 µmol, 1 equiv) in THF (4.5 mL) at -78 ºC, was slowly added *n*BuLi (283 µL, 2.5 M, 1.1 equiv) and the mixture was stirred for 1 h at this temperature. A solution of **S22** (180 mg, 771 µmol, 1.2 equiv) in THF (2 mL) was added and the resulting solution was allowed to reach 23 ºC over 14 h. The reaction was quenched by addition of sat. NH_4Cl. The aqueous phase was extracted with EtOAc (3 x 10 mL) and the combined organic layers were dried over MgSO4. The solvent was evaporated and the residue was redissolved in methanol (8 mL). Anhydrous K_2CO_3 (180 mg, 1.23 mmol, 2 equiv.) was added and the mixture was vigorously stirred for 1.5 h at 23 ºC. EtOAc (5 mL) was added and the reaction was quenched with brine (10 mL). The aqueous phase was extracted with EtOAc (2 x 10 mL) and the combined organic layers were dried over $MgSO_4$. The solvent was evaporated and the residue was purified by column chromatography on silica with cyclohexane/EtOAc 30:1 to 20:1 affording **S16** (148 mg, 474 µmol, 74 % yield over 2 steps) as a pale-orange oil.

^{1}H NMR (500 MHz, $CDCl_3$) δ 7.76 (d, *J* = 8.0 Hz, 1H), 7.25 – 7.22 (m, 2H), 7.20 – 7.15 (m, 1H), 5.56 (thex, *J* = 7.0, 1.3 Hz, 1H), 5.36 (thept, *J* = 7.0, 1.5 Hz, 1H), 3.94 – 3.87 (m, 4H), 3.86 – 3.71 (m, 2H), 3.26 (s, 3H), 2.74 (s, 1H), 1.83 (s, 3H), 1.80 (q, *J* = 1.0 Hz, 3H), 1.74 (q, *J* = 1.2 Hz, 3H), 1.65 (br s, 3H).
^{13}C NMR (126 MHz, $CDCl_3$) δ 139.6, 138.4, 136.9, 133.2, 131.4, 128.3, 128.0, 127.8, 125.9, 121.5, 84.9, 77.8, 76.3, 75.5, 66.1, 52.4, 30.8, 30.3, 25.9, 18.1, 14.3.
HRMS (ESI) *m/z* calc. for $C_{21}H_{28}NaO_2$ $[M+Na]^+$: 335.1982; found: 335.1984.

(1a*S*,5*S*,5a*S*,11b*R*)-5-methoxy-1,1,5,11-tetramethyl-1,1a,2,4,5,5a,6,11b-octahydrocyclopropa[*c*]naphtho[2,3-*e*]oxocine (S17)

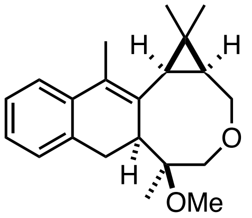

^{1}H NMR (500 MHz, $CDCl_3$) δ 7.19 – 7.14 (m, 2H), 7.07 – 7.03 (m, 2H), 3.94 (dd, *J* = 11.3, 4.8 Hz, 1H), 3.78 (d, *J* = 11.7 Hz, 1H), 3.54 (dddt, *J* = 13.3, 9.0, 4.4, 2.1 Hz, 1H), 3.23 (d, *J* = 11.7 Hz, 1H), 3.14 (s, 3H), 2.96 (t, *J* = 11.6 Hz, 1H), 2.80 (dd, *J* = 15.3, 13.0 Hz, 1H), 2.59 (dd, *J* = 8.8, 2.3 Hz, 1H), 2.53 (dd, *J* = 15.3, 9.0 Hz, 1H), 2.22 (ddd, *J* = 11.8, 8.7, 4.9 Hz, 1H), 2.00 (d, *J* = 2.3 Hz, 3H), 1.18 (s, 3H), 1.15 (s, 3H), 1.06 (s, 3H).
^{13}C NMR (126 MHz, $CDCl_3$) δ 139.7, 137.9, 134.8, 128.3, 126.5, 125.9, 122.7, 121.4, 75.7, 74.5, 67.2, 48.6, 46.8, 44.3, 39.1, 36.1, 27.0, 24.0, 22.8, 20.0, 13.8.
HRMS (ESI) *m/z* calc. for $C_{21}H_{28}NaO_2$ $[M+Na]^+$: 335.1982; found: 335.1987.

(*E*)-1-methyl-2-(2-methyl-3-((3-methylbut-2-en-1-yl)oxy)prop-1-en-1-yl)naphthalene (S18)

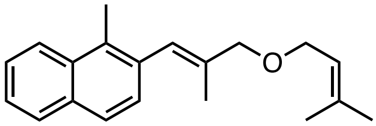

^{1}H NMR (500 MHz, $CDCl_3$) δ 8.05 (d, *J* = 8.4 Hz, 1H), 7.82 (d, *J* = 8.0 Hz, 1H), 7.66 (d, *J* = 8.4 Hz, 1H), 7.51 (ddd, *J* = 8.4, 6.8, 1.5 Hz, 1H), 7.46 (ddd, *J* = 8.0, 6.8, 1.3 Hz, 1H), 7.29 (d, *J* = 8.4 Hz, 1H), 6.73 (s, 1H), 5.45 (dddd, *J* = 8.3, 5.5, 2.8, 1.4 Hz, 1H), 4.11 (d, *J* = 1.3 Hz, 2H), 4.06 (dt, *J* = 6.9, 0.9 Hz, 2H), 2.59 (s, 3H), 1.79 (d, *J* = 1.3 Hz, 3H), 1.73 (s, 3H), 1.70 (d, *J* = 1.4 Hz, 3H).
^{13}C NMR (126 MHz, $CDCl_3$) δ 137.2, 136.4, 134.2, 133.0, 132.7, 131.7, 128.5, 128.0, 127.2, 126.0, 125.6, 125.3, 124.3, 121.4, 75.8, 66.5, 26.0, 18.2, 15.6, 15.5.
HRMS (ESI) *m/z* calc. for $C_{20}H_{24}NaO$ $[M+Na]^+$: 303.1719; found: 303.1718.

2. Theoretical DFT Computations

2.1 Computational methods

Calculations were performed by means of the Gaussian 09 suite of programs.[5] For the study of *trans*-fused bicyclo[5.1.0]octanes the presented energies were obtained using B3LYP[6] functional, while for the study of cyclopropyl gold(I) carbene intermediates, M06[7] was used instead. All reported geometries and energies include the dispersion correction GD3.[8] Both methods (B3LYP-D3 and M06-D3) provide very similar results for this type of systems as can be seen in the benchmark study provided in section 2.2 of this Supporting Information. The 6-31G(d) basis sets[9] were employed for all atoms (C, H, P, and O) except for gold for which the SDD[10] basis sets together with the corresponding ECP were used instead. Full geometry optimizations were carried out in dichloromethane, through an implicit polarizable continuum model (PCM) as implemented in Gaussian 09.[11] The stationary points were characterized by vibrational analysis. Transition states were identified by the presence of one imaginary frequency while minima by a full set of real frequencies. The connectivity of the transition states was confirmed by the relaxation of each transition state towards both the reactant and the product. Reported energies in the main text correspond to free energies (G) in solution, computed at 298 K and 1 atm.

For mechanistic comparison, some test calculations were run with $InCl_3$ instead of our gold catalyst. For these calculations the M06[7]-D3[8] functional was used together with the 6-31G(d) basis sets[9] for all atoms (C, H, P, and O) except for In and Cl for which the LANL2DZ basis set[12] was utilized to describe In and Cl with ECP and addition polarization function (ζd = 0.143 for In, ζd = 0.640 for Cl).[13] Geometry optimizations were carried out in dichloromethane, through the use of PCM[11], and reported energies also correspond to free energies (G) in solution.

A small benchmark[14] study on the performance of different DFT methods was performed on the study of cyclopropyl gold(I) carbene intermediates and results obtained are presented in section 2.2 of this Supporting Information. Evaluation of the methods was performed by Single Point (SP) energies on structures optimized with the BP86-D3[15] functional and 6-31G(d) basis sets[9] for all atoms except for gold for which the SDD[10] basis sets and ECP were used instead. For the different methods (B97D-D3,[16] BP86-D3,[15] TPSS-D3,[17] PBE-D3,[18] B3LYP-D3,[6] B3PW91-D3,[19] BMK-D3,[20] M06,[21] M06L,[21] M062X,[21]M06HF,[21] PBE,[22] and PBE0[22]) were tested with the larger basis sets 6-311+G(d,p) for all atoms, except for Au. Results of these functional were compared between them and between SP calculations using the DLPNO-CCSD(T),[23] as implemented in ORCA[24]. In this case, the energies presented correspond to potential energies in solution (E) and in $kcal.mol^{-1}$.

NBO analysis[25] was carried out as implemented in Gaussian 09, with BP86[15]-D3[8]/6-31G(d) (C, H, P), SDD[10] (Au) level, based on Natural Orbitals (NO), which are used to calculate the distribution electron density in atoms and bonds.
Quantum Theory Atoms in Molecules (QTAIM)[26] analysis was carried out with Multiwfn software[27] with M06[7]-D3[8]/6-31G(d) (C, H, P), SDD[10] (Au) level CH_2Cl_2 (PCM).[11] The bond critical points (BCPs) and the ring critical points (RCPs) were located and analyzed using Laplacian maps.

2.2 Results

Exploring the Intermediates of Cycloisomerization Reactions

Different angles and bond lengths were observed when comparing **Int2** with **Int3** (Table S1). Remarkably, the angle between (C_2-C_3-C_4) is 115.9º for **Int3** whereas at **Int2** is 69.6º. A longer distance between (C_2-C_4) was also observed for open carbocation species **Int3** 2.584 Å *vs* 1.734 Å for **Int2**. Interestingly, the cationic or carbenic character of gold(I) carbenes was also clear when comparing the (C_1-C_2) distances bearing 1.417 Å for the carbonic double bond of **Int2** and 1.351 Å for **Int3**.

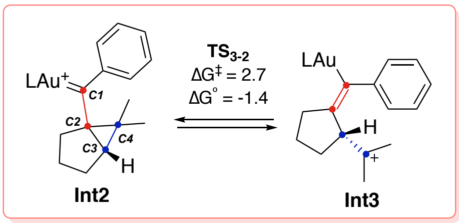

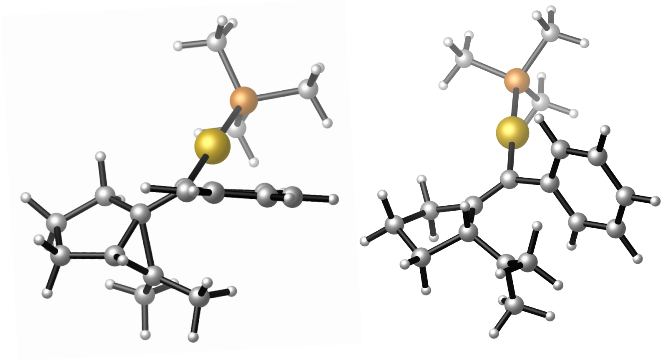

Int2 **Int3**

	d(C_1-C_2)	d(C_2-C_3)	d(C_3-C_4)	d(C_2-C_4)	angle(C_2-C_3-C_4)
**Int2**	1.417	1.552	1.487	1.734	69.6
**Int3**	1.351	1.575	1.474	2.584	115.9

Table S1. Calculated bond distances and angles of **Int2** and **Int3** by DFT at the BP86-D3/6-31G(d) (C, H, P) and SDD (Au) level in CH_2Cl_2 (PCM). Distances expressed in Å and angles in degrees.

Benchmark of Density Functional Theory Methods

These results prompted us to compute these intermediates and TSs using different DFT functionals. In order to discard any artifact associated to the DFT method of choice, we performed a benchmark of DFT functionals.[14] Hence, we compared the performance of twelve different functionals on the intermediates and TSs mentioned before. In addition, we used larger basis sets for this benchmark to increase accuracy.

Very similar computed energies were obtained when changing the basis set from 6-31G(d) to the larger 6-311+G(d,p) (Table S2). Although the relative energies could have changed depending on the method used, no important changes were observed when using pure methods B97D-D3,[16] BP86-D3,[15] TPSS-D3,[17] PBE-D3.[18] Interestingly, better results were observed when using the hybrid version of PBE, PBE0.[22]
However, when using Grimme's B97D-D3, **Int1** was destabilized with respect to the other pure functionals, thus, same energy was observed for **Int2** and **Int3** in this case, 6.2 kcal/mol. Similarly, hybrid functionals B3LYP-D3,[6] B3PW91-D3,[19] BMK-D3,[20] or Minnesota functionals[21] do not show many differences, despite slightly higher barriers were found with respect to initial BP86 functional. Likewise, comparing energies obtained from Minnesota functionals (M06, M06L, M062X, M06HF) similar energies were obtained with all of them including the non-hybrid, M06L.

	**Int1**	**Int2**	**Int3**	**$TS_{1\text{-}2}$**	**$TS_{1\text{-}3}$**	**$TS_{2\text{-}3}$**
**BP86**	0.0	1.7	2.7	10.3	12.9	4.5
**B3LYP**	0.0	7.5	9.2	13.5	16.5	9.8
**B3PW91**	0.0	1.2	4.6	12.8	16.1	5.9
**B97D**	0.0	6.2	6.2	10.5	13.0	7.6
**BMK**	0.0	1.4	6.9	16.6	20.7	7.5
**TPSS**	0.0	1.7	4.9	10.8	13.6	6.4
**M062X**	0.0	5.5	11.5	15.9	19.4	11.8
**M06HF**	0.0	4.5	8.7	14.7	19.7	9.8
**M06**	0.0	3.4	8.4	14.8	18.0	9.4
**M06L**	0.0	4.1	9.9	14.8	17.3	10.9
**PBE0**	0.0	-1.8	3.3	12.3	15.6	4.1
**PBE**	0.0	-0.6	2.0	9.6	12.0	3.3
**DLPNO-CCSD(T)**	0.0	3.4	8.2	14.3	18.1	8.4

Table S2. DFT functional benchmark regarding **Int1**, **Int2**, **Int3**, **$TS_{1\text{-}2}$**, **$TS_{1\text{-}3}$**, **and $TS_{2\text{-}3}$**. L=PMe_3 on BP86-D3/6-31G(d),SDD(Au) with PCM geometries.. Dispersion GD3 and solvent CH_2Cl_2 (PCM) was included in all functionals. Finally, single points calculations in DLPNO-CCSD(T) Energies of single points related to **Int1** in kcal/mol, (6-311+G(d,p)) basis set and SDD (Au) level.

In addition, we used domain pair natural orbital method, DLPNO-CCSD(T),[23] as implemented in ORCA as reference high accurate energies in the faster and easier way. This method has recently been used as calibration for benchmark studies of the performance of density functionals and thus, to select the most appropriate functional for the system we studied. We calculated the energies for **Int1**, **Int2**, **Int3**, **$TS_{1\text{-}2}$**, **$TS_{1\text{-}3}$**, **and $TS_{2\text{-}3}$** (Table S2, last row). The averages of the differences between each single point energy and the one obtained with DLPNO-CCSD(T) show that, the most appropriated functionals for this system are B3LYP-D3 and M06-D3, giving slightly better results for M06-D3. Thus, we used M06-D3 for the study of cyclopropyl gold(I) carbene intermediates. For the study of trans-fused bicyclo[5.1.0]octanes as B3LYP-D3 calculations were already performed at the end of the benchmark study and this suggests B3LYP-D3 results are

accurate, they were not repeated except for some tests detailed bellow that confirm very similar results for both systems also in trans-fused bicyclo[5.1.0]octanes.

We optimized the intermediates and TSs at M06-D3/6-31G(d) (C, H, P) and SDD (Au) level in order to compare them with the single points (Table S3). Very similar energies were obtained for both cases, although lower activation barriers were observed for the new optimizations than for single point calculations.

Method	Int1	Int2	Int3	TS_{1-2}	TS_{1-3}	TS_{2-3}
**M06-D3/6-311+G****	0.0	3.4	8.4	14.8	18.0	9.4
**M06-D3/6-31-G***	0.0	2.4	7.0	13.1	14.8	8.5

Table S3. Free energies related to **Int1** in kcal/mol.

Substrate Effect on the Carbenic or Cationic Character of the Intermediates

To further investigate how the nature of the substrate can influence the formation of **Int2**, **Int3** and **Int4**, seven different enynes bearing substituents at the alkyne or alkene moiety were tested (Table S4).

Enyne	Method	Species	d(C_1-C_2)	d(C_2-C_3)	d(C_2-C_4)	A(C_2-C_3-C_4)	$\Delta G^{\ddagger}$	ΔG^{o}
**24**	BP86-D3	**24-Int2**	1.417	1.552	1.734	69.6	9.7	0.8
**24**	BP86-D3	**24-Int3**	1.651	1.575	2.584	115.9	10.8	2.8
**24**	M06-D3	**24-Int2**	1.413	1.536	1.652	66.5	13.1	2.4
**24**	M06-D3	**24-Int3**	1.338	1.555	2.549	115.4	14.8	7.0
**25**	BP86-D3	**25-Int2**	1.387	1.727	1.645	61.5	-	-7.9
**25**	BP86-D3	**25-Int3**	1.372	1.618	1.915	77.0	-	-5.8
**25**	M06-D3	**25-Int2**	1.375	1.667	1.636	62.9	5.2	-6.1
**25**	M06-D3	**25-Int3**	1.338	1.590	2.353	101.8	8.0	-1.0
**26**	BP86-D3	**26-Int2**	1.414	1.608	1.672	65.8	8.1	-2.4
**26**	BP86-D3	**26-Int3**	-	-	-	-	-	-
**26**	M06-D3	**26-Int2**	1.404	1.589	1.630	64.7	13.5	-2.6
**26**	M06-D3	**26-Int3**	1.335	1.552	2.534	115.3	-	-0.7
**27**	BP86-D3	**9-Int4**	1.424	1.688	1.540	57.7	7.6	-7.4
**27**	M06-D3	**9-Int4**	1.416	1.632	1.527	58.8	13.9	-5.6
**28**	BP86-D3	**10-Int4**	1.413	1.795	1.545	55.3	6.6	-6.6
**28**	M06-D3	**10-Int4**	1.409	1.667	1.534	57.9	8.8	-6.8
**29**	BP86-D3	**11-Int4**	1.398	1.747	1.541	56.4	3.8	-9.1
**29**	M06-D3	**11-Int4**	1.385	1.674	1.531	58.1	13.0	-7.4
**30**	BP86-D3	**12-Int4**	1.377	1.948	1.568	52.5	3.9	-9.0
**30**	M06-D3	**12-Int4**	1.372	1.758	1.561	57.4	8.9	-7.0
**31**	BP86-D3	**13-Int4**	1.395	1.746	1.565	57.5	2.4	-7.3
**31**	M06-D3	**13-Int4**	1.382	1.678	1.559	59.2	7.1	-8.5

Table S4. Calculated bond distances and angles of optimized **Int2, Int3, Int4**, $\mathbf{TS_{1\text{-}2}}$, $\mathbf{TS_{1\text{-}3}}$ and $\mathbf{TS_{1\text{-}4}}$ by DFT at the BP86-D3 and M06-D3L=PMe_3. Free energies in kcal/mol and referred to **Int1**. Distances expressed in Å and angles in degrees.

For those enynes having a terminal alkene moiety (R^2 and R^3 = H, enynes **27**, **28, 29, 30**), only intermediate **Int4** was observed (similar results were obtained when using BP86-D3 instead of M06-D3). Despite all these substrates delivered the same intermediate **Int4**, some structural differences were observed depending on the R^1 and R^4 substituents. Hence, for R^1 = Ph (enynes **27**, **28**), the distance between C1–C2 is longer than in the case of R^1 = H (enynes **29**, **30**) due to

conjugation of phenyl ring. Likewise, having a methyl substituent as R^4 (enynes **28**, **30**) leads to higher distances between C2–C3.

On the other hand, we observed the formation of both **Int2** and **Int3** for enyne **25** that has a disubstituted alkene moiety (R^2 and R^3 = Me). Very similar structures to those observed for our first studied enyne **24** were found. For both substrates, **Int3** showed a clearly larger angle (C_2-C_3-C_4) corresponding to a stabilized carbocation species. However, for substrate **25** that has a terminal alkyne, this angle is smaller (101.8º *vs* 115.4º).

Furthermore, for monosubstituted alkenes (R^2 = Me and R^3 = H, enynes **31**, **26**), the alkyne moiety was found to determine the formation of **Int2**, **Int3** or **Int4**. Thus, terminal alkyne **31** (R^1 = H) delivered **Int4**, whereas enyne **26** (R^1 = Ph) led to the formation of **Int2** together with **Int3**. It is important to highlight that for substrate **26** no transition state was found to connect **26-Int1** with **26-Int3**. However, an alternative pathway was encountered that connects directly **26-Int1** with the Wheland intermediate **26-IntA** (Table S5).

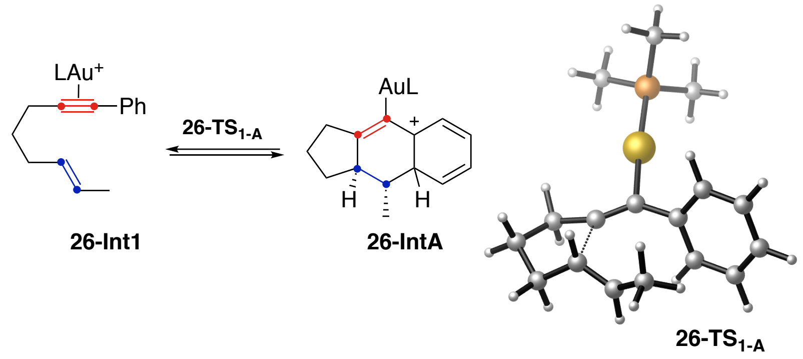

Method	Int1	IntA	$TS_{1\text{-}A}$
**BP86-D3**	0.0	-10.1	11.2
**M06-D3**	0.0	-7.0	17.4

Table S5. Computed free energies for the formation of Wheland intermediate **26-IntA** and transition states by DFT at the BP86-D3 and M06-D3. L=PMe_3. Free energies in kcal/mol respect to **Int1**.

Finally, for more accurate comparison, we decided to explore the cyclization of aryl tethered 1,6-enynes at the same level of theory (BP86-D3/6-31G(d) (C, H, P) and SDD (Au) in CH_2Cl_2 (PCM), L = PH_3) (Scheme S1). We found that 6-*endo*-dig pathways (**32-TS$_{1\text{-}3a}$**, **32-TS$_{1\text{-}2a}$**) are more favored than 5-*exo*-dig routes (**32-TS$_{1\text{-}3b}$**, **32-TS$_{1\text{-}2b}$**) by at least 0.7 kcal/mol. Similarly, the lowest energy barrier corresponds to the formation of cyclopropyl gold carbene **32-Int2a**. In addition, as we observed before, intermediates of type **Int3** were found to be connected to **Int2** via **32-TS$_{3\text{-}2}$**. Interestingly, no formation intermediates of type **Int4** were observed for these aryl enynes.

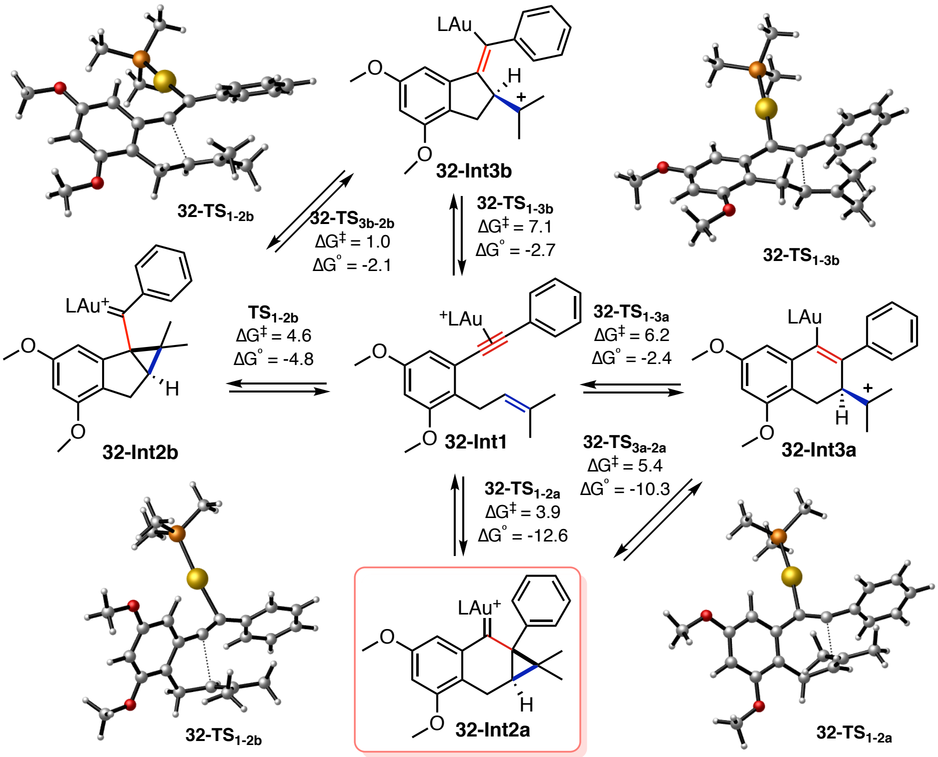

Scheme S1. Calculated formation of cyclopropyl gold(I) carbenes **Int2** and open carbocations **Int3** for the 5-*exo*-dig and the 6-*endo*-dig pathways of aryl tethered 1,6-enyne **32**. L=PMe3. DFT calculations performed with BP86-D3/6-31G(d) (C, H, P) and SDD (Au) in CH_2Cl_2 (PCM). Free energies in kcal/mol.

Trans-fused Cyclopropanes by Gold(I)-catalyzed Cyclization Cascade

We started computing the simpler enynes **S27** instead of a dienyne. Moreover, since this substrate does not contain an internal nucleophile such in substrates **4a**, **9**, and **10**, we will later compute these other systems in order to compare the reaction pathways and to computationally confirm that the formation of the fused tetrahydrofuran ring plays a crucial role in the final cyclopropanation step. Calculations were run using PMe_3 as model ligand for gold(I). Two possible cyclopropyl gold carbenes could be formed when using enyne **S27** as substrate, depending on the orientation of the alkene moiety. Thus, one possible intermediate would have the hydrogen in *syn* to the methoxy group and the other one in *anti*. Interestingly, we observed the formation of both **Int2** and **Int3** types of intermediates (Scheme S2). Hence, when the hydrogen and methoxy group are in *syn*, formation of cyclopropylgold(I) carbene **S27-Int2** was observed. In contrast, opened carbocation species **S27-Int3** were preferentially formed when the methoxy group and the hydrogen are in *anti*-position. This fact is probably due to steric hindrance, since the alkene is placed very close to the methoxy group and the closed carbene (type of **Int2**) is not that favorable. Noteworthy, in the absence of the alkyl chain tethered to the alkene, formation of cyclopropyl gold carbene **S27-Int2** is 3.7 kcal/mol more favorable than formation of the opened carbocation species **S27-Int3**.

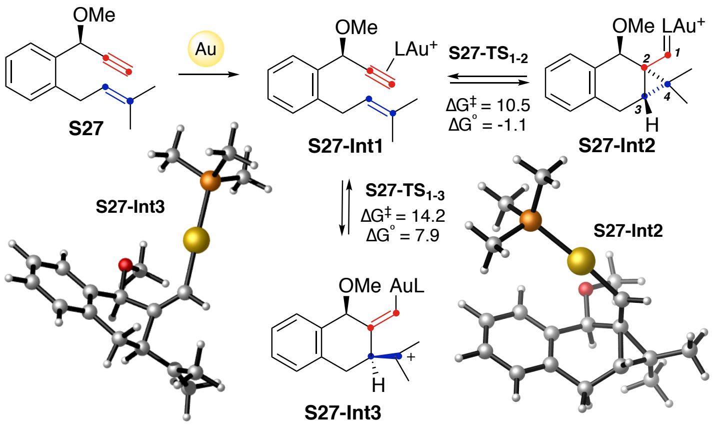

Scheme S2. Calculated formation of cyclopropyl gold(I) carbene **S27-Int2** and open carbocation **S27-Int3** for enyne **S27**. L=PMe_3. DFT calculations performed with B3LYP-D3/6-31G(d) (C, H, O, P) and SDD (Au) in CH_2Cl_2 (PCM). Free energies in kcal/mol.

Although B3LYP was the method of choice for this system, we decided to compute the same pathways using the best alternative functionals found in the aforementioned DFT benchmark. Also, we wanted to proof the existence of these intermediates when using other methods and thus, discard possible computational artifacts (Table S6). Both **S27-Int2** and **S27-Int3** were found as minima when BP86-D3 and M06-D3 were used. In three cases, **S27-Int2** is more stable than **S27-Int3** (Scheme S1 and Table S6). The same tendency was observed for enyne **S27** than with the previously studied substrate **24**. Thus, formation of **S27-Int2** was always favored over **S27-Int3** and **S27-TS$_{1-2}$** was lower than **S27-TS$_{1-3}$** for all the cases. Computed results using BP86-D3 were lower in energy for TSs and intermediates than B3LYP-D3 and M06-D3.

Method	S27-Int1	S27-Int2	S27-Int3	S27-TS$_{1-2}$	S27-TS$_{1-3}$
**BP86-D3**	0.0	-6.0	2.2	7.5	11.1
**M06-D3**	0.0	-6.2	6.2	11.9	16.7

Table S6. Computed free energies of **S27-Int2**, **S27-Int3**, **S27-TS$_{1-2}$**, **S27-TS$_{1-3}$**, related to **S27-Int1**. DFT calculations performed with BP86-D3 or M06-D3. Free energies in kcal/mol. L=PMe_3.

2.3 Coordinates and Energies of Relevant Intermediates and Transition States (B3LYP, and M06, L=PMe_3)

24-Int1

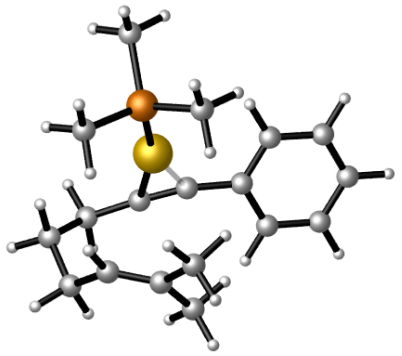

E = - 1179.30316221 Hartrees
G = -1178.968694 Hartrees

6	-0.901773	0.246583	1.197415
6	0.004756	0.849549	1.822143
6	-2.716360	0.865288	-1.332630
1	-3.372848	0.722032	-2.213105
1	-3.261971	1.543686	-0.651142
6	-1.420327	1.475467	-1.808920
1	-0.951820	0.938930	-2.648828
6	-0.786728	2.583631	-1.349954
6	-1.255205	3.420656	-0.184914
1	-1.403302	4.474470	-0.489467
1	-0.477716	3.426396	0.603871
1	-2.189599	3.056250	0.270193
6	0.514469	3.047694	-1.966300
1	1.330034	3.020317	-1.215091
1	0.442512	4.097416	-2.311495
1	0.814323	2.423714	-2.826019
6	0.793890	1.679007	2.692573
6	2.161323	1.403732	2.939954
6	0.172811	2.789009	3.320311
6	2.893171	2.228666	3.803130
1	2.633183	0.543973	2.452547
6	0.915659	3.600927	4.186404
1	-0.882519	3.000077	3.122389
6	2.274204	3.326606	4.427297
1	3.949696	2.012422	3.991756
1	0.431984	4.454470	4.672372
1	2.850195	3.968838	5.101451
15	2.437690	-0.597100	-1.720915
6	1.613724	-0.947203	-3.326303
1	2.364915	-1.258060	-4.072522
1	1.095339	-0.040855	-3.677955
1	0.873783	-1.751654	-3.187605
6	3.693848	0.696172	-2.075785
1	4.376163	0.344681	-2.868413
1	4.268155	0.908646	-1.159785
1	3.187405	1.617972	-2.403219
6	3.386077	-2.117080	-1.314603
1	2.683209	-2.950114	-1.153297
1	3.964101	-1.951564	-0.391301

24-TS1-3

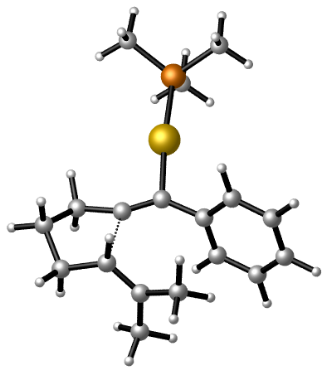

E = - 1179.28460928 Hartrees
G = -1178.951557 Hartrees

6	-0.677957	0.978214	-0.553512
6	0.504346	1.110872	-0.049176
6	-3.227046	2.029214	-0.955065
1	-3.946637	2.713342	-1.444764
1	-3.749343	1.578427	-0.092021
6	-2.016070	2.825715	-0.519885
1	-1.445978	3.266971	-1.351840
6	-1.829699	3.418273	0.717121
6	-2.634391	3.059954	1.937269
1	-3.451993	3.796835	2.070555
1	-2.008604	3.112906	2.844700
1	-3.090511	2.059280	1.877446
6	-0.898725	4.593024	0.857624
1	-0.278981	4.535841	1.768509
1	-1.515271	5.511281	0.951507
1	-0.240408	4.712397	-0.017375
6	1.137948	2.016080	0.912555
6	2.174431	2.898251	0.531186
6	0.699248	1.991403	2.257403
6	2.718828	3.784177	1.470149
1	2.524431	2.896283	-0.506471
6	1.263219	2.867493	3.196337
1	-0.090583	1.291776	2.547826
6	2.264089	3.773944	2.802592
1	3.502104	4.485564	1.163861
1	0.912358	2.850478	4.233622
1	2.696744	4.464756	3.533802
15	3.273838	-2.045781	-1.455680
6	2.897989	-2.749102	-3.113351
1	3.653075	-3.508589	-3.379657
1	2.903011	-1.941765	-3.863627
1	1.898431	-3.212374	-3.091968
6	4.996126	-1.410763	-1.573276
1	5.673958	-2.219911	-1.895551
1	5.312823	-1.032458	-0.587877
1	5.032048	-0.585408	-2.302306
6	3.369861	-3.495382	-0.327570
1	2.380377	-3.976413	-0.266065
1	3.666462	-3.155892	0.678061

1	4.071790	-2.364744	-2.142848
79	0.931038	0.016318	-0.089703
6	-2.159515	-0.446694	0.850392
1	-2.100832	-1.474595	1.256859
1	-2.959775	0.072643	1.413853
6	-2.544179	-0.514395	-0.646836
1	-1.793311	-1.117277	-1.193107
1	-3.496547	-1.072214	-0.695697

24-Int2

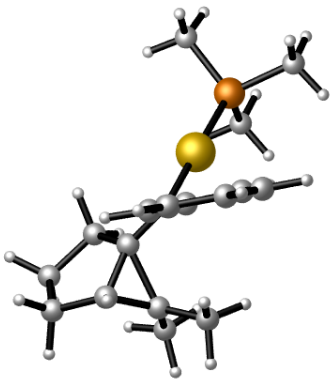

E = -1179.30573929 Hartrees
G = -1178.967494 Hartrees

79	-1.500902	7.009305	-7.052687
15	-2.652805	4.983686	-7.325717
6	-1.002499	9.740447	-5.903887
6	-0.472940	8.771197	-6.792003
6	0.779224	8.960553	-7.520120
6	1.679057	7.875432	-7.713410
6	1.095369	10.216548	-8.109441
6	2.879134	8.058328	-8.406889
1	1.431952	6.900033	-7.279523
6	2.272627	10.378796	-8.848571
6	3.177199	9.308895	-8.981355
1	4.105441	9.444529	-9.546179
1	3.576476	7.221833	-8.519283
1	2.489429	11.342894	-9.320142
1	0.385563	11.044421	-8.021268
6	-1.492648	11.946466	-4.870167
1	-1.277474	12.404772	-3.888985
1	-1.558652	12.766429	-5.605728
6	-0.358381	11.019284	-5.306148
1	0.475201	11.486003	-5.840716
6	0.028610	9.822891	-4.511997
6	1.472981	9.364257	-4.595099
1	2.003409	9.827177	-3.741832
1	1.984937	9.685366	-5.513179
1	1.565632	8.269050	-4.496710
6	-0.661192	9.347303	-3.249853
1	-1.662407	9.765028	-3.087452
1	-0.032969	9.645273	-2.389638
1	-0.727587	8.245284	-3.240411
6	-2.502461	9.763004	-5.567541
1	-3.042769	9.687486	-6.526843
1	-2.805977	8.885540	-4.967947

1	4.110818	-4.219292	-0.708358
79	1.757842	-0.442310	-0.768937
6	-1.695958	0.113587	-1.202668
1	-1.169054	-0.569627	-1.893941
1	-2.162211	-0.515828	-0.418770
6	-2.776012	0.930534	-1.929585
1	-2.349439	1.385847	-2.842810
1	-3.611013	0.278764	-2.236668

24-$TS_{1\text{-}2}$

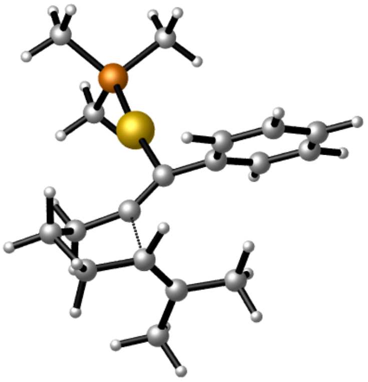

E = -1179.28834677 Hartrees
G = -1178.953159 Hartrees

79	-1.770734	6.774781	-7.015017
15	-2.854973	4.847724	-7.690763
6	-1.247206	9.188854	-5.628786
6	-0.619948	8.482638	-6.515903
6	0.653071	8.600738	-7.246351
6	1.666218	7.627044	-7.095449
6	0.865698	9.694780	-8.115201
6	2.892230	7.778643	-7.757494
1	1.489262	6.769911	-6.436518
6	2.091918	9.833710	-8.784368
6	3.111449	8.883864	-8.599784
1	4.068754	8.997620	-9.119133
1	3.679353	7.030070	-7.617171
1	2.249679	10.688671	-9.450593
1	0.066695	10.429118	-8.260679
6	-1.720650	11.890461	-4.785413
1	-1.783786	12.044562	-3.694604
1	-1.809286	12.884512	-5.257674
6	-0.392449	11.272219	-5.159493
1	-0.006966	11.501196	-6.161261
6	0.462319	10.604796	-4.300807
6	1.848318	10.206684	-4.736536
1	2.585048	10.720521	-4.088838
1	2.057640	10.464016	-5.785403
1	2.011597	9.122727	-4.600687
6	0.135558	10.273125	-2.867896
1	-0.833759	10.662874	-2.524422
1	0.925215	10.670708	-2.203524
1	0.138062	9.175088	-2.731308
6	-2.502672	9.520219	-4.917760
1	-3.281247	8.817232	-5.265408
1	-2.391100	9.374653	-3.826924

6	-2.807900	11.109799	-4.854468
1	-3.153547	10.940501	-3.821265
1	-3.620361	11.651160	-5.366306
6	-1.614466	3.603342	-7.962743
1	-0.787674	3.414956	-7.258387
1	-1.191488	3.885317	-8.940601
1	-2.222023	2.688518	-8.072032
6	-4.067668	5.093399	-8.500306
1	-4.573010	4.115336	-8.576897
1	-3.696262	5.396952	-9.492716
1	-4.781930	5.851771	-8.140362
6	-3.387369	4.332995	-5.767336
1	-3.912000	3.382366	-5.963737
1	-4.098847	5.070700	-5.361830
1	-2.586198	4.166861	-5.028884

24-Int3

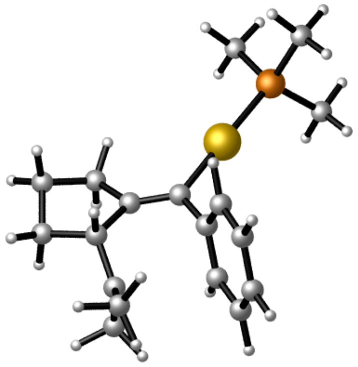

E = -1179.30322281 Hartrees
E = -1178.965207 Hartrees

6	-0.708217	1.290388	-0.610748
6	0.549840	1.104821	-0.154368
6	-2.853334	2.398561	-1.081858
1	-3.296033	3.350045	-1.418586
1	-3.555614	1.922134	-0.376183
6	-1.463015	2.654363	-0.385500
1	-0.920945	3.426832	-0.962797
6	-1.591567	3.094508	1.015102
6	-2.441892	2.322459	1.968699
1	-3.466348	2.752080	1.906982
1	-2.117536	2.453626	3.012697
1	-2.507675	1.252525	1.717453
6	-1.209716	4.487788	1.367266
1	-1.259452	4.692901	2.446239
1	-1.924886	5.159584	0.842535
1	-0.207444	4.749431	0.981595
6	1.106492	2.059892	0.813490
6	2.058493	3.041563	0.437209
6	0.628164	2.053896	2.154536
6	2.460750	4.017891	1.353187
1	2.441486	3.043815	-0.588944
6	1.080981	3.008567	3.088707
1	-0.024482	1.236148	2.471866
6	1.972682	4.006051	2.683444
1	3.161206	4.799865	1.040769
1	0.718010	2.974543	4.121162
1	2.308992	4.768654	3.393251

6	-2.889299	10.977950	-5.238250
1	-3.828669	11.251539	-4.728009
1	-3.052213	11.075044	-6.327114
6	-1.803084	3.777979	-8.754552
1	-0.894589	3.493169	-8.199610
1	-1.509463	4.337734	-9.657248
1	-2.360135	2.871102	-9.045802
6	-4.375084	5.164936	-8.676421
1	-4.832203	4.209319	-8.985874
1	-4.111317	5.755034	-9.568879
1	-5.091813	5.738706	-8.066965
6	-3.389024	3.770793	-6.298496
1	-3.880331	2.862889	-6.688712
1	-4.092207	4.325441	-5.656455
1	-2.507245	3.486489	-5.701836

24-TS$_{3\text{-}2}$

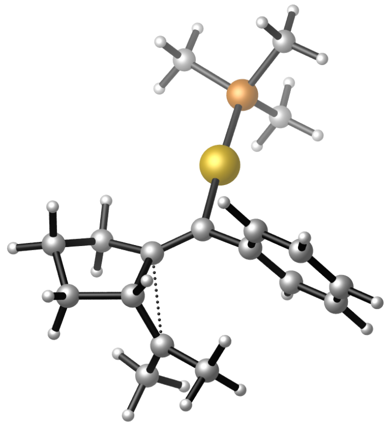

E = -1179.29900272 Hartrees
E = -1178.960965 Hartrees

6	-0.739782	1.458426	-0.559955
6	0.545883	1.308181	-0.155967
6	-2.791872	2.599280	-1.406062
1	-2.985870	3.534676	-1.955370
1	-3.708016	2.353986	-0.842032
6	-1.585808	2.817622	-0.428899
1	-0.959018	3.673989	-0.718786
6	-1.874550	2.824003	0.998177
6	-2.808746	1.845778	1.621314
1	-3.724147	2.397577	1.923396
1	-2.382543	1.439506	2.555527
1	-3.111718	1.024311	0.957910
6	-1.315209	3.878866	1.882754
1	-1.095763	3.522275	2.901132
1	-2.134090	4.630309	1.977314
1	-0.442994	4.395672	1.457014
6	1.238401	2.315235	0.685311
6	1.755551	3.499984	0.110817
6	1.390300	2.104816	2.075144
6	2.356478	4.476074	0.921445
1	1.662347	3.656915	-0.969733
6	2.002184	3.077657	2.879525
1	1.007517	1.178027	2.517337
6	2.476824	4.271861	2.308474
1	2.732341	5.399753	0.467672
1	2.101452	2.906572	3.957090

15	3.227661	-2.158947	-1.392941
6	2.504864	-3.301175	-2.645149
1	3.225814	-4.096098	-2.902757
1	2.246145	-2.729823	-3.551554
1	1.586463	-3.753604	-2.236785
6	4.807339	-1.581163	-2.146137
1	5.438424	-2.442740	-2.424181
1	5.344820	-0.948350	-1.421147
1	4.584748	-0.981783	-3.044077
6	3.747870	-3.256366	-0.007173
1	2.854013	-3.709586	0.451704
1	4.272691	-2.656122	0.753921
1	4.417507	-4.052487	-0.375861
79	1.794062	-0.432926	-0.747641
6	-1.551280	0.420966	-1.526579
1	-0.925591	-0.163398	-2.222173
1	-2.132071	-0.304040	-0.919692
6	-2.510066	1.411341	-2.213158
1	-1.991814	1.939489	-3.034992
1	-3.412419	0.931673	-2.628285

1	2.947898	5.034485	2.937495
15	3.102803	-2.111070	-1.225516
6	2.492949	-3.130314	-2.633564
1	3.184813	-3.967287	-2.830631
1	2.414151	-2.496920	-3.532222
1	1.493640	-3.526230	-2.389632
6	4.813574	-1.626481	-1.707556
1	5.415015	-2.522844	-1.937587
1	5.280924	-1.070430	-0.878580
1	4.770142	-0.973083	-2.594090
6	3.334407	-3.310254	0.153986
1	2.355071	-3.718457	0.452631
1	3.780856	-2.789036	1.016570
1	3.997009	-4.133501	-0.163933
79	1.718538	-0.304551	-0.689666
6	-1.598521	0.497959	-1.365128
1	-0.964194	-0.247971	-1.873153
1	-2.292729	-0.063235	-0.707488
6	-2.389195	1.417856	-2.311787
1	-1.729356	1.767391	-3.126749
1	-3.264829	0.924815	-2.765799

24-In-Int2

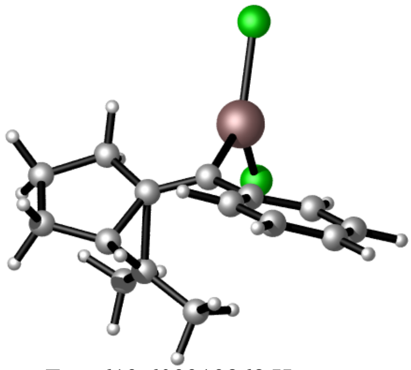

E = -613.609319868 Hartrees
E = -613.368953 Hartrees

6	-1.013846	9.791902	-5.975267
6	-0.458122	8.890406	-6.868693
6	0.786589	9.008017	-7.578125
6	1.583858	7.869488	-7.808767
6	1.216527	10.254058	-8.080050
6	2.804616	7.982373	-8.451989
1	1.270230	6.893649	-7.428749
6	2.416976	10.353090	-8.762682
6	3.220582	9.224516	-8.931033
1	4.169840	9.310865	-9.455108
1	3.427083	7.102995	-8.595025
1	2.732820	11.312119	-9.166191
1	0.576212	11.126280	-7.967037
6	-1.580822	11.876678	-4.808945
1	-1.380718	12.287250	-3.811565
1	-1.684632	12.728126	-5.490586
6	-0.413242	11.038947	-5.295681
1	0.381047	11.569428	-5.819931
6	0.044702	9.856810	-4.560623
6	1.477124	9.440306	-4.684319

24-In-Int3

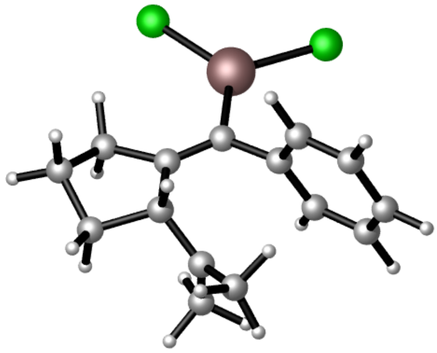

E = -613.619052439 Hartrees
E = -613.381060 Hartrees

6	-0.677549	1.415925	-0.715635
6	0.548541	1.221772	-0.211141
6	-2.882555	2.397777	-1.026225
1	-3.412177	3.326270	-1.263052
1	-3.502326	1.813557	-0.332830
6	-1.480516	2.688137	-0.396440
1	-1.030180	3.535342	-0.937491
6	-1.615966	3.021354	1.025728
6	-2.179189	2.050089	1.968529
1	-3.250509	2.314015	2.055210
1	-1.772725	2.181486	2.977228
1	-2.108490	1.009115	1.639717
6	-1.419295	4.401464	1.461668
1	-1.682303	4.574841	2.507812
1	-1.997936	5.066426	0.799840
1	-0.367606	4.697067	1.287184
6	1.126267	2.203153	0.732298
6	1.750185	3.362952	0.251207
6	1.027854	2.008698	2.118386

1	1.997485	9.882123	-3.822913
1	1.977283	9.809777	-5.583266
1	1.596620	8.350797	-4.615200
6	-0.628029	9.271202	-3.358444
1	-1.640667	9.629934	-3.173554
1	-0.018119	9.541791	-2.484873
1	-0.633662	8.173793	-3.413052
6	-2.490762	9.718986	-5.615967
1	-3.059524	9.673634	-6.555589
1	-2.739764	8.814246	-5.032757
6	-2.846528	10.997796	-4.836824
1	-3.188713	10.759269	-3.824056
1	-3.674929	11.525356	-5.319562
49	-1.612921	7.069391	-7.004080
17	-3.147541	6.443842	-8.607220
17	-1.312242	5.662369	-5.171557
6	2.205165	4.331316	1.136732
1	1.851328	3.507478	-0.824298
6	1.497762	2.975090	3.004943
1	0.587602	1.084905	2.496400
6	2.072152	4.143108	2.516268
1	2.667680	5.238859	0.754056
1	1.409931	2.813693	4.077193
1	2.432689	4.903775	3.205279
6	-1.474742	0.578072	-1.680649
1	-0.851094	0.107664	-2.451103
1	-1.972130	-0.237017	-1.128587
6	-2.519975	1.540976	-2.235547
1	-2.082112	2.168562	-3.023654
1	-3.395586	1.034685	-2.655042
49	1.704311	-0.465158	-0.708612
17	1.200335	-2.059240	-2.328989
17	3.689797	-0.896890	0.427184

30-In-Int4

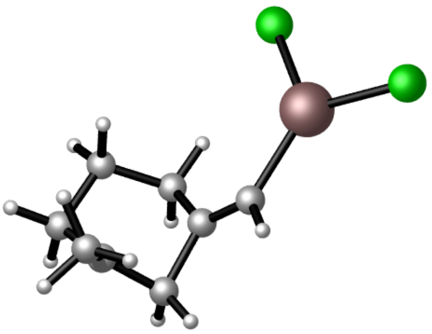

E = -343.460852054 Hartrees
G = -343.323227 Hartrees

6	-0.845650	1.205055	0.030082
6	0.466447	1.067692	0.253976
6	-2.588933	2.587545	-1.705681
1	-2.657534	3.221491	-2.595103
1	-3.583292	2.460223	-1.255765
6	-1.651652	3.127118	-0.714652
6	-1.593996	2.467893	0.577178
6	-1.728382	0.365609	-0.842907
1	-1.231198	-0.571647	-1.123876
1	-2.651170	0.097019	-0.308387
6	-2.049593	1.172053	-2.095234
1	-1.138012	1.293164	-2.697853
1	-2.807630	0.686475	-2.718317
1	0.984599	1.844079	0.820293
1	-2.570051	2.162321	0.969219
6	-0.700052	4.168907	-1.086674
1	-0.368608	4.075486	-2.128711
1	-1.276888	5.112629	-1.046703
1	0.142466	4.268724	-0.396724
1	-0.996347	2.990506	1.327385
49	1.639740	-0.552627	-0.395426
17	0.965000	-2.052018	-2.043988
17	3.778837	-0.959678	0.400935

30-Int4

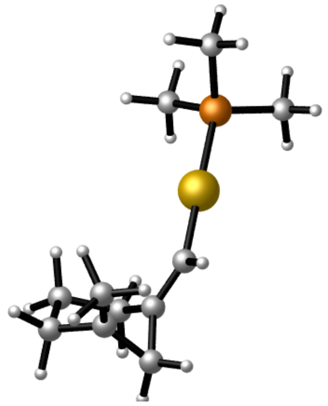

E = -908.382142047 Hartrees
G = -908.135865 Hartrees

6	-1.038553	1.382503	-0.001878
6	0.325747	1.309379	0.122884
6	-2.423879	2.519820	-1.797737
1	-2.266606	3.169613	-2.666080
1	-3.470486	2.628805	-1.478659
6	-1.516871	2.941067	-0.661060
6	-1.849250	2.530596	0.678214
15	3.009971	-1.930604	-1.261951
6	2.280742	-3.049231	-2.505578
1	3.004251	-3.823986	-2.790339
1	1.995315	-2.477891	-3.395805
1	1.384367	-3.526484	-2.094438
6	4.550212	-1.337420	-2.039554
1	5.175298	-2.189739	-2.335115
1	5.106148	-0.710986	-1.333392
1	4.312699	-0.739130	-2.926195
6	3.579992	-3.045095	0.065709
1	2.719815	-3.533916	0.536315
1	4.116598	-2.470443	0.828731
1	4.249558	-3.810824	-0.347235
79	1.557235	-0.205127	-0.523844
6	-1.874259	0.383527	-0.770797
1	-1.336817	-0.567891	-0.865616
1	-2.820276	0.181520	-0.248123

26-TS1-A

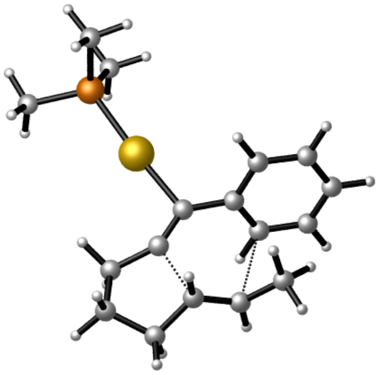

E = -1139.21816081 Hartrees
G = -1138.902158 Hartrees

6	-0.631042	1.292792	-0.708782
6	0.484287	1.267818	-0.066203
6	-3.038477	2.339910	-1.107295
1	-3.744330	3.078640	-1.510938
1	-3.525002	1.862585	-0.242317
6	-1.775006	3.036242	-0.669495
1	-1.209130	3.517066	-1.477342
6	-1.640410	3.605117	0.571117
6	-0.773223	4.771370	0.851841
1	-0.276890	4.716129	1.827954
1	-1.412198	5.669479	0.877256
1	-0.024672	4.921416	0.064729
6	1.033294	2.045065	1.029076
6	2.226104	2.773139	0.900382
6	0.348613	2.053262	2.254411
6	2.686751	3.541851	1.958335
1	2.766804	2.746386	-0.045435
6	0.832902	2.805199	3.321061
1	-0.553191	1.452325	2.363028
6	1.990896	3.561280	3.170112
1	3.596788	4.127708	1.845103
1	0.298797	2.804469	4.269347
1	2.364240	4.157188	4.000622
15	3.186447	-2.036787	-1.422390
6	2.418651	-3.313612	-2.472305
1	3.162107	-4.075410	-2.740768
1	2.024271	-2.855602	-3.386106
1	1.592094	-3.789921	-1.933681
6	4.610577	-1.440063	-2.391110
1	5.251490	-2.285475	-2.672743
1	5.192626	-0.728463	-1.795344
1	4.259361	-0.934898	-3.297427
6	3.929719	-2.964775	-0.041195
1	3.143388	-3.456025	0.542132
1	4.478050	-2.280236	0.615076
1	4.620527	-3.723825	-0.430615
79	1.721182	-0.337195	-0.751836
6	-1.575211	0.457251	-1.502998
1	-0.991127	-0.106727	-2.242385
1	-2.027995	-0.281212	-0.823081
6	-2.656802	1.303694	-2.148675
1	-2.261275	1.806390	-3.043094
1	-3.507174	0.689349	-2.464052
6	-2.124231	1.049494	-2.119138
1	-1.201015	0.999039	-2.717593
1	-2.930031	0.583185	-2.697088
1	0.783305	2.183483	0.600679
1	-1.336503	3.022167	1.505001
1	-2.872244	2.221635	0.893965
6	-0.514534	4.013356	-0.938788
1	-1.079855	4.934936	-1.139266
1	0.152226	4.215545	-0.094769
1	0.078519	3.794496	-1.835096

26-Int1

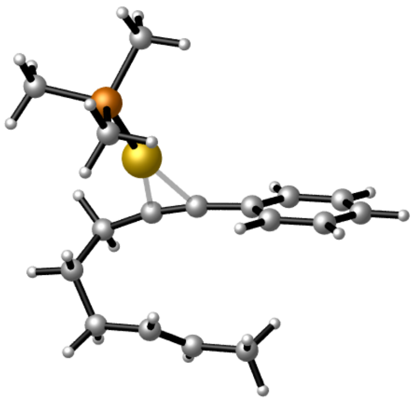

E = -1139.24482632 Hartrees
G = -1138.929412 Hartrees

6	-0.796499	0.458243	0.236615
6	0.072606	1.010346	0.918654
6	-2.378038	2.141530	-1.984160
1	-2.476960	2.428137	-3.043371
1	-3.372593	2.284718	-1.531715
6	-1.393813	3.042743	-1.307598
1	-0.363014	3.013706	-1.685872
6	-1.683574	3.856213	-0.290484
6	-0.709148	4.754500	0.394486
1	-0.589180	4.484469	1.455082
1	-1.040698	5.802203	0.373821
1	0.281171	4.701251	-0.076749
6	0.888320	1.808313	1.787114
6	1.974713	2.535576	1.277583
6	0.547014	1.915322	3.142683
6	2.705506	3.363555	2.118243
1	2.223260	2.462756	0.218413
6	1.282790	2.750444	3.972998
1	-0.298976	1.349841	3.528375
6	2.359953	3.473031	3.463848
1	3.543978	3.931398	1.720944
1	1.014227	2.837356	5.023548
1	2.934050	4.125033	4.118969
15	2.835945	-1.702515	-1.561702
6	2.135236	-3.014270	-2.610449
1	2.944412	-3.574109	-3.096416
1	1.490805	-2.572179	-3.377818
1	1.537586	-3.698697	-1.998919
6	3.887065	-0.712284	-2.667849
1	4.624812	-1.359582	-3.158740

1	-2.298167	3.258618	1.373982

26-IntA

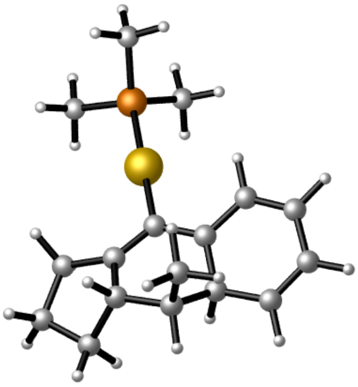

E = -1139.26531603 Hartrees
G = -1138.941015 Hartrees

6	-0.654075	1.509427	-0.733780
6	0.547964	1.263607	-0.135877
6	-2.899088	2.049559	-0.388472
1	-3.713801	2.782556	-0.424021
1	-2.997492	1.485020	0.553487
6	-1.515214	2.711468	-0.453752
1	-1.511458	3.332783	-1.369483
6	-1.071667	3.569918	0.730637
6	-0.302375	4.800134	0.267225
1	0.026143	5.421947	1.109107
1	-0.937499	5.418257	-0.379285
1	0.586091	4.519925	-0.317037
6	0.885402	2.054993	0.989025
6	2.209121	2.229358	1.445749
6	-0.229245	2.715683	1.737521
6	2.475974	3.011881	2.540510
1	3.019210	1.754501	0.892464
6	0.158092	3.500673	2.932550
1	-0.911197	1.920828	2.092023
6	1.444907	3.643085	3.310318
1	3.512231	3.163364	2.839008
1	-0.648992	3.966746	3.498214
1	1.716070	4.226017	4.186611
15	3.244351	-1.987261	-1.570604
6	2.448949	-3.223081	-2.652790
1	3.177133	-3.988138	-2.952060
1	2.053444	-2.731882	-3.548767
1	1.618788	-3.702940	-2.122666
6	4.677692	-1.408617	-2.542029
1	5.290139	-2.263691	-2.856669
1	5.289058	-0.732976	-1.933473
1	4.332941	-0.865145	-3.428716
6	3.994224	-2.984249	-0.237978
1	3.208553	-3.481350	0.341779
1	4.566447	-2.334887	0.434047
1	4.664258	-3.742217	-0.663797
79	1.813952	-0.265431	-0.811120
6	-1.413926	0.595075	-1.646614
1	-0.995970	0.614361	-2.664460
1	-1.305803	-0.442697	-1.296806
1	4.409688	0.059903	-2.092994
1	3.267987	-0.226111	-3.429490
6	3.984144	-2.549471	-0.432746
1	3.428622	-3.240023	0.210903
1	4.495103	-1.814353	0.198118
1	4.729091	-3.110899	-1.011169
79	1.205599	-0.457193	-0.462049
6	-1.978668	0.055294	-0.530599
1	-2.028328	-1.040431	-0.584339
1	-2.863695	0.378692	0.040931
6	-2.020654	0.654741	-1.943156
1	-1.051808	0.481608	-2.439469
1	-2.768621	0.093959	-2.518898
1	-2.713171	3.872948	0.082678

LAu

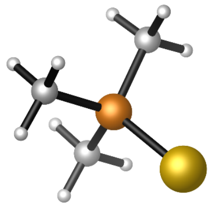

E = -596.765373204 Hartrees
G = -596.683236 Hartrees

79	6.284485	-0.341989	-1.494417
15	7.258370	-1.879404	-2.845827
6	8.986848	-1.437200	-3.240007
1	9.407478	-2.195856	-3.908702
1	9.012170	-0.460536	-3.729449
1	9.571274	-1.394368	-2.317632
6	7.296186	-3.533143	-2.070015
1	6.275600	-3.866796	-1.867040
1	7.785310	-4.237264	-2.751077
1	7.853316	-3.482026	-1.131409
6	6.375839	-2.053648	-4.435414
1	6.886306	-2.807201	-5.044424
1	5.345650	-2.366804	-4.249461

S27-Int1

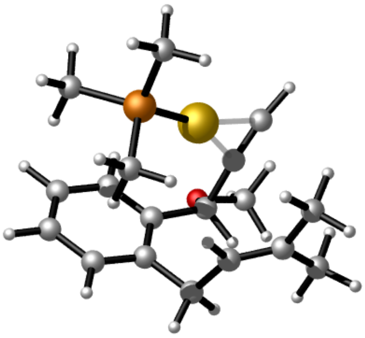

E = -1254.40405230 Hartrees
G = -1254.054442 Hartrees

6	1.318611	1.744657	0.114567

6	-2.869447	1.101190	-1.591992
1	-3.111886	1.663254	-2.503863
1	-3.595154	0.285028	-1.512548
1	-1.965406	3.893035	1.286739

S27-TS$_{1-2}$

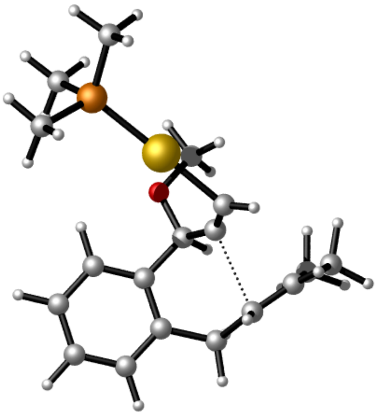

E = -1254.38829242 Hartrees
G = -1254.037760 Hartrees

6	1.232823	2.467085	-1.048773
6	0.003225	2.185534	-1.650661
1	-0.210058	2.587278	-2.637928
6	-0.950215	1.398713	-0.998652
1	-1.901469	1.192306	-1.480744
6	-0.677647	0.881544	0.268670
1	-1.415341	0.270931	0.781525
6	0.548954	1.150297	0.882128
1	0.772977	0.753719	1.866218
6	1.495446	1.941645	0.229885
6	3.879361	1.392379	0.084341
6	4.848718	3.086684	-1.569064
6	3.608132	2.630208	-1.918309
1	3.560969	1.729865	-2.528073
79	3.901837	-1.047206	1.477058
15	3.169663	-2.614773	3.027635
6	4.483581	-3.701920	3.701270
1	4.058232	-4.396135	4.433216
1	5.251009	-3.089459	4.182746
1	4.942848	-4.267388	2.885698
6	2.388623	-1.836442	4.494072
1	2.033778	-2.606146	5.187227
1	1.545834	-1.220758	4.167843
1	3.116947	-1.196457	4.999553
6	1.894465	-3.752402	2.361356
1	1.043142	-3.168624	2.000062
1	1.559627	-4.443846	3.141447
1	2.312455	-4.319695	1.525297
6	4.560162	0.322638	-0.014711
1	5.292780	-0.012535	-0.736343
8	2.868203	1.837763	2.206701
6	4.004976	2.304255	2.934219
1	4.927449	1.830875	2.574704
1	3.837051	2.022589	3.974836
1	4.098147	3.395750	2.856782
6	6.080596	2.355267	-2.029191
1	5.842952	1.418342	-2.539949
6	0.206143	0.890575	0.078533
1	-0.146000	0.529382	-0.884563
6	-0.458303	0.503355	1.243020
1	-1.318402	-0.157274	1.180922
6	-0.020876	0.977070	2.482145
1	-0.535393	0.691894	3.395034
6	1.083701	1.825417	2.540438
1	1.431460	2.204780	3.495697
6	1.756304	2.207824	1.371735
6	4.191453	2.272325	1.901103
6	4.570631	1.662102	-1.433890
6	3.294936	1.252713	-1.332980
1	3.109581	0.179377	-1.319562
79	4.000950	-0.049485	1.783408
15	3.098811	-2.160235	1.505218
6	4.305358	-3.520178	1.724327
1	3.807695	-4.484052	1.577148
1	4.725842	-3.476586	2.732498
1	5.114402	-3.411418	0.996672
6	1.737596	-2.487069	2.684764
1	1.317494	-3.481794	2.504668
1	0.963677	-1.726628	2.549260
1	2.117824	-2.427900	3.708006
6	2.369335	-2.402396	-0.157142
1	1.597416	-1.645730	-0.321332
1	1.927269	-3.401438	-0.228719
1	3.147312	-2.291567	-0.917122
6	5.251227	1.795640	2.305385
1	6.238356	1.596245	2.674809
8	2.749894	4.069114	2.503524
6	3.737113	5.099267	2.560518
1	4.710894	4.708413	2.885147
1	3.379679	5.824517	3.293249
1	3.851959	5.589970	1.584082
6	5.695385	0.655775	-1.485819
1	5.326971	-0.375158	-1.464605
1	6.367990	0.792853	-0.626606
1	6.306005	0.788279	-2.388713
6	5.022440	3.100783	-1.470422
1	5.602128	3.340280	-0.567518
1	4.201574	3.817900	-1.549533
1	5.691317	3.268950	-2.323935
6	2.992991	3.087851	1.517231
1	3.238770	3.566535	0.561219
6	2.039452	2.081433	-1.184258
1	1.360141	1.862778	-2.017746
1	2.253328	3.153241	-1.241631

1	6.753039	2.146324	-1.188567
1	6.637168	2.988592	-2.732103
6	5.114146	4.341475	-0.782403
1	5.643882	4.097389	0.147720
1	4.215446	4.906836	-0.531076
1	5.777146	5.000663	-1.356175
6	2.862158	2.202482	0.843846
1	3.122565	3.265106	0.753126
6	2.276144	3.326433	-1.742900
1	1.912537	3.589830	-2.744128
1	2.393181	4.274176	-1.205601

S27-Int2

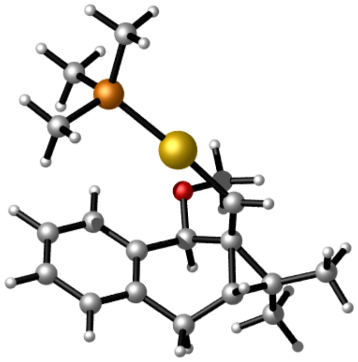

E = -1254.41262414 Hartrees
G = -1254.056248 Hartrees

6	1.486764	1.678850	-0.712080
6	0.315219	0.993603	-1.040808
1	0.029076	0.892144	-2.084550
6	-0.491056	0.445067	-0.038633
1	-1.399542	-0.086529	-0.306618
6	-0.128773	0.589541	1.301839
1	-0.755782	0.173947	2.085795
6	1.047059	1.267022	1.638937
1	1.346799	1.372287	2.675070
6	1.861229	1.800780	0.637552
6	4.284443	2.004270	-0.007064
6	4.883909	2.941834	-1.102995
6	3.840788	2.024637	-1.612698
1	4.187490	1.171645	-2.189726
79	3.849918	-0.764244	1.281945
15	2.710480	-2.431467	2.493953
6	3.687795	-3.919214	2.938437
1	3.068048	-4.630010	3.494583
1	4.541649	-3.621807	3.553760
1	4.059290	-4.395845	2.026833
6	2.018894	-1.823885	4.082147
1	1.457537	-2.619726	4.582534
1	1.354500	-0.978861	3.881373
1	2.831337	-1.486667	4.731867
6	1.254397	-3.066551	1.573482

S27-Int3

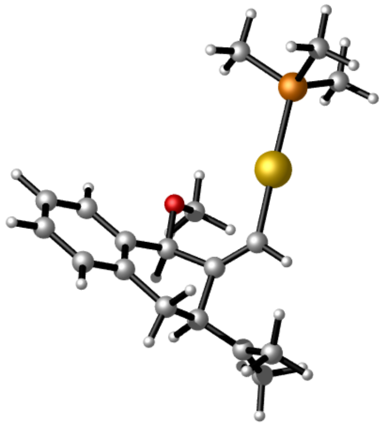

E = -1254.39282795 Hartrees
G = -1254.041865 Hartrees

6	0.700195	3.047518	0.996010
6	-0.398818	3.896663	0.813788
1	-0.256437	4.970789	0.907929
6	-1.662047	3.389212	0.513901
1	-2.499925	4.066601	0.375305
6	-1.842297	2.008748	0.399202
1	-2.822265	1.600204	0.169012
6	-0.758563	1.151598	0.585321
1	-0.888554	0.078539	0.497340
6	0.515603	1.655494	0.876726
6	2.991811	1.397880	0.795966
6	4.420038	2.909425	2.052938
6	3.020990	2.602070	1.837454
1	2.628654	2.173610	2.769095
79	3.560674	-0.056935	-1.852199
15	3.310955	-1.463742	-3.714233
6	1.660644	-2.266509	-3.819276
1	1.601060	-2.906300	-4.705903
1	1.491362	-2.870135	-2.922874
1	0.885022	-1.497053	-3.871936
6	4.497654	-2.865769	-3.780171
1	4.309024	-3.488103	-4.661354
1	5.518985	-2.476593	-3.822200
1	4.392433	-3.473793	-2.876930
6	3.507343	-0.627709	-5.339407
1	4.509600	-0.195033	-5.408814
1	3.360814	-1.340847	-6.157312
1	2.771818	0.177407	-5.423709
6	3.809420	1.221152	-0.250559
1	4.683042	1.870283	-0.317720
8	1.456225	-0.476847	0.309181
6	2.094525	-1.645265	0.820876
1	1.686183	-1.910156	1.806375
1	1.887563	-2.448895	0.111651
1	3.179932	-1.506726	0.899805
6	5.222012	2.080752	2.969139
1	4.685703	1.212550	3.353917
1	6.164757	1.782788	2.493061
1	5.516002	2.729679	3.812263
6	5.064952	4.015599	1.331831
1	4.559626	4.953543	1.609745
1	6.136859	4.095244	1.513952
1	4.866850	3.909726	0.253571

1	0.602258	-2.226014	1.319701
1	0.704116	-3.790861	2.182730
1	1.587743	-3.545585	0.648650
6	4.715151	0.681882	0.137510
1	5.543310	0.399310	-0.513247
8	3.524513	2.263843	2.297321
6	4.589494	3.084778	2.767829
1	5.520428	2.895840	2.215747
1	4.738766	2.828215	3.818211
1	4.334330	4.150745	2.685628
6	6.320984	2.685734	-1.547996
1	6.538480	1.633351	-1.742106
1	7.020873	3.050582	-0.789279
1	6.508194	3.231731	-2.478305
6	4.609644	4.436305	-0.963087
1	5.206038	4.850723	-0.144216
1	3.565058	4.692263	-0.786683
1	4.914471	4.932810	-1.889719
6	3.169157	2.504933	0.950821
1	3.034203	3.585168	0.812894
6	2.356298	2.326555	-1.775061
1	2.054791	1.984972	-2.769532
1	2.203076	3.411883	-1.753317

S27-TS$_{1-3}$

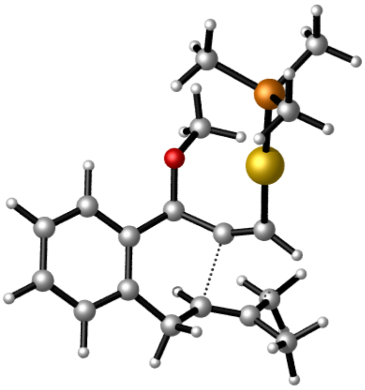

E = -1254.38026719 Hartrees
G = -1254.031808 Hartrees

6	0.721555	2.996312	0.895236
6	-0.422938	3.760672	0.630208
1	-0.357172	4.843567	0.703240
6	-1.634368	3.167264	0.279511
1	-2.503916	3.786491	0.078507
6	-1.721651	1.776944	0.201420
1	-2.660205	1.297064	-0.060666
6	-0.595799	0.998478	0.467622
1	-0.657816	-0.081723	0.410639
6	0.628372	1.591863	0.802909
6	3.112367	1.258962	0.616217
6	4.284318	3.000404	2.239521
6	2.922429	2.933232	2.143841
1	2.414373	2.402192	2.946829
79	3.580357	-0.010610	-1.930761
15	3.198687	-1.376729	-3.772844
6	1.505730	-2.083703	-3.797024

6	1.671568	0.671900	1.093021
1	1.680167	0.369422	2.154727
6	2.055987	3.665854	1.303433
1	2.466514	4.119702	0.393586
1	1.941046	4.469981	2.040788

9a-TS$_{6-7b}$

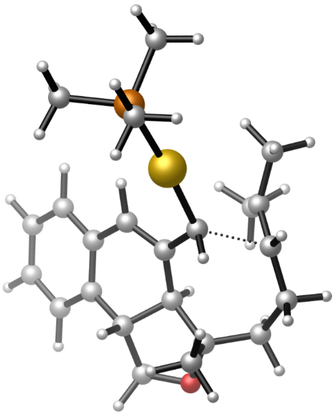

E = -1448.56561761 Hartrees
G = -1448.114332 Hartrees

6	3.940652	5.472908	1.743546
1	4.714688	6.220237	1.948218
1	2.958864	5.959529	1.813478
6	4.115400	4.783000	0.383218
1	5.186424	4.715561	0.139659
6	3.403302	5.328853	-0.827407
6	3.120260	6.679672	-1.019900
1	3.379916	7.399697	-0.248311
6	2.506402	7.116651	-2.199437
1	2.282385	8.171210	-2.332841
6	2.188808	6.199350	-3.204202
1	1.717920	6.536897	-4.123001
6	2.480025	4.846673	-3.027136
1	2.234337	4.130310	-3.807726
6	3.078363	4.391981	-1.840977
6	3.376845	2.970245	-1.651489
1	3.315783	2.331861	-2.530570
6	3.699029	2.428788	-0.448338
6	4.042247	0.983942	-0.341273
1	4.927372	0.848179	0.293216
6	3.405901	0.013567	1.269363
6	3.556018	0.656250	2.633822
1	4.557322	0.386561	2.983568
1	2.865922	0.077911	3.267758
6	3.329881	2.147998	2.929360
1	2.277066	2.427732	2.829317
1	3.581222	2.290118	3.986737
6	4.181920	3.129468	2.111978
6	3.592705	3.383929	0.718526
1	2.513553	3.525609	0.895654
6	5.678986	2.790516	2.158826
1	6.265187	3.552312	1.638616
1	5.920931	1.821484	1.713509
1	6.005393	2.772552	3.203717
8	4.012595	4.429102	2.737171

1	1.375436	-2.724291	-4.675313
1	1.343500	-2.669767	-2.888134
1	0.774371	-1.271064	-3.825243
6	4.323598	-2.823548	-3.859796
1	4.079247	-3.443922	-4.728344
1	5.357447	-2.475967	-3.939826
1	4.221927	-3.417751	-2.947349
6	3.381610	-0.528849	-5.389339
1	4.400889	-0.145073	-5.487949
1	3.172923	-1.226694	-6.206805
1	2.682243	0.310243	-5.439352
6	4.007151	1.268004	-0.293852
1	4.905259	1.866214	-0.385546
8	1.615631	-0.573541	0.445468
6	2.356084	-1.659760	1.004911
1	2.112587	-1.792024	2.067541
1	2.058931	-2.548801	0.446276
1	3.436840	-1.506869	0.891265
6	5.017505	2.228147	3.298473
1	4.344417	1.650570	3.937305
1	5.740672	1.544467	2.835616
1	5.596439	2.914790	3.929712
6	5.138291	3.823357	1.327632
1	5.537534	4.676319	1.893341
1	6.009389	3.249786	0.984739
1	4.602720	4.217585	0.462342
6	1.816673	0.667385	1.098183
1	1.887858	0.484374	2.179071
6	2.005113	3.734168	1.254443
1	2.520130	4.044245	0.338330
1	1.735167	4.660712	1.779307

9a-Int6

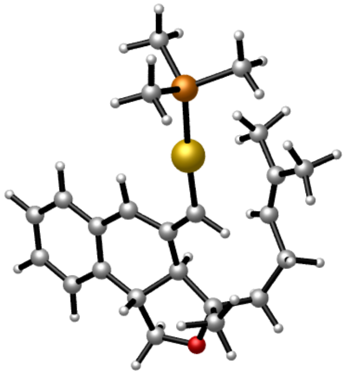

E = -1448.58726727 Hartrees
G = -1448.138627 Hartrees

6	3.388852	5.110505	1.743491
1	3.713677	6.124441	1.996129
1	2.297081	5.117576	1.599494
6	4.087512	4.524362	0.523885
1	5.158552	4.772347	0.585239
6	3.619292	4.879120	-0.857427
6	3.007524	6.081468	-1.190688
1	2.790925	6.813687	-0.419044
6	2.672893	6.357604	-2.523223
1	2.184830	7.297104	-2.765422
6	2.968913	5.446861	-3.547046
1	2.712874	5.680775	-4.575174

79	4.735765	-0.030299	-2.092336
15	5.630083	-1.028646	-3.999750
6	7.196950	-1.940288	-3.707578
1	7.566317	-2.374759	-4.642243
1	7.947079	-1.253909	-3.304355
1	7.023577	-2.738069	-2.979837
6	4.518592	-2.248310	-4.804634
1	3.583348	-1.755694	-5.085635
1	4.994575	-2.662640	-5.699438
1	4.293229	-3.057591	-4.104322
6	6.027446	0.178638	-5.324969
1	6.439417	-0.335494	-6.199352
1	5.117407	0.712424	-5.613199
1	6.755451	0.904450	-4.951864
1	4.041220	-0.868006	1.188225
6	2.420234	0.064726	0.277376
6	1.255880	1.022026	0.300256
1	1.348482	1.812428	1.040372
1	1.114646	1.475627	-0.685998
1	0.354058	0.444728	0.532905
6	2.149884	-1.174776	-0.553403
1	1.825516	-0.908957	-1.563386
1	3.015486	-1.837412	-0.616669
1	1.331460	-1.724476	-0.070095

9a-Int7a

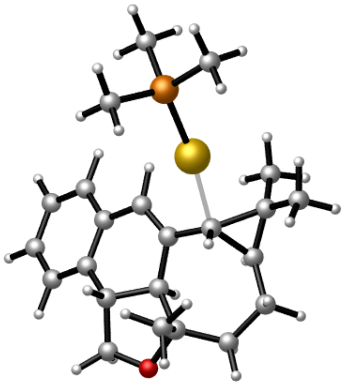

E = -1448.59199111 Hartrees
G = -1448.139731 Hartrees

6	3.006744	6.067276	-2.765890
1	2.764324	7.079738	-3.076222
6	2.845132	5.002400	-3.654778
1	2.476920	5.181449	-4.660991
6	3.156805	3.703996	-3.248615
1	3.026713	2.873133	-3.938547
6	3.624874	3.450631	-1.950226
6	3.932109	2.078725	-1.518486
1	3.967270	1.311239	-2.289373
6	4.091578	1.756385	-0.216958
6	4.292437	0.408062	0.410583
1	5.169583	0.361584	1.056075
6	3.073382	0.189973	1.415601
1	2.213539	0.770131	1.082489
6	3.390710	0.434382	2.877678
1	4.335547	-0.042681	3.157909
1	2.613518	-0.017661	3.506577
6	3.427711	1.957340	3.169674

6	3.595369	4.248609	-3.236024
1	3.833227	3.531104	-4.016368
6	3.908117	3.938210	-1.891575
6	4.523357	2.693201	-1.559661
1	4.902032	2.077318	-2.372782
6	4.650280	2.203468	-0.253700
6	5.326851	0.999094	-0.047949
1	5.447611	0.728479	0.996567
6	2.931438	-0.012580	1.408008
1	2.138063	0.604891	0.985586
6	3.422972	0.419192	2.768904
1	4.459084	0.102913	2.937916
1	2.836505	-0.114098	3.532377
6	3.265133	1.923170	3.063373
1	2.228857	2.221535	2.858535
1	3.424719	2.086285	4.135657
6	4.180148	2.923751	2.327608
6	3.920417	3.021298	0.799727
1	2.851473	2.800347	0.651542
6	3.301427	-1.104289	0.705951
6	2.647972	-1.438027	-0.612306
1	3.401590	-1.512399	-1.407925
1	1.909018	-0.686543	-0.908376
1	2.145067	-2.413612	-0.559883
6	4.320648	-2.109225	1.181536
1	3.815885	-3.025613	1.518051
1	4.933426	-1.744965	2.010333
1	4.990434	-2.399287	0.362094
6	5.652206	2.779926	2.732150
1	6.295414	3.430414	2.131032
1	6.020917	1.755372	2.639308
1	5.751589	3.078039	3.780195
8	3.759659	4.240293	2.813505
79	6.215641	-0.266497	-1.385473
15	7.236369	-1.838267	-2.816336
6	8.979001	-1.453081	-3.248069
1	9.387517	-2.230863	-3.901413
1	9.024776	-0.487066	-3.758753
1	9.575740	-1.396559	-2.333308
6	7.302333	-3.523639	-2.089578
1	6.286680	-3.870923	-1.879171
1	7.788826	-4.218603	-2.781644
1	7.863016	-3.491166	-1.151130
6	6.393535	-2.062548	-4.432203
1	6.913177	-2.818363	-5.029941
1	5.360888	-2.381085	-4.263545
1	6.385191	-1.112894	-4.974679

1	2.410720	2.359166	3.083124
1	3.738904	2.113921	4.208886
6	4.331399	2.818350	2.256433
6	3.852445	2.867191	0.785136
1	2.756264	2.982564	0.851690
6	3.340545	-0.949542	0.549076
6	2.351044	-1.215764	-0.569891
1	2.801050	-1.827533	-1.358300
1	1.991303	-0.283992	-1.015319
1	1.490275	-1.758244	-0.161064
6	4.003190	-2.181472	1.142941
1	3.249627	-2.716101	1.734359
1	4.834775	-1.930399	1.806558
1	4.370067	-2.860594	0.366928
6	5.820738	2.504433	2.474413
1	6.455848	3.022015	1.749438
1	6.048760	1.436036	2.423630
1	6.098166	2.853479	3.473699
8	4.124303	4.202307	2.659099
79	5.674198	-0.673095	-1.135693
15	7.209524	-1.493336	-2.621840
6	8.767604	-0.530358	-2.637375
1	9.466840	-0.964870	-3.359145
1	8.551287	0.504654	-2.915496
1	9.215797	-0.543441	-1.640316
6	7.693620	-3.225948	-2.276936
1	6.808253	-3.865889	-2.320608
1	8.424841	-3.562172	-3.019384
1	8.130307	-3.291182	-1.276742
6	6.604631	-1.483398	-4.350539
1	7.379098	-1.877151	-5.016788
1	5.707418	-2.103502	-4.426616
1	6.355183	-0.460020	-4.643513

9aa-Int5

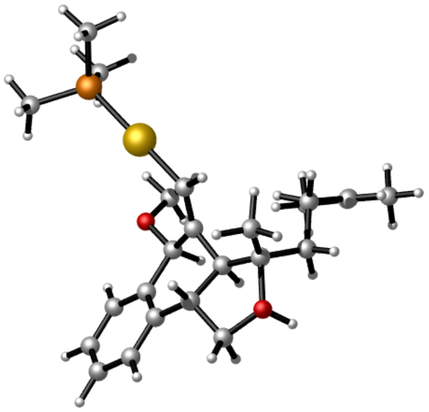

E = -1564.29165613 Hartrees
G = -1563.794700 Hartrees

6	3.702467	4.201601	1.974822
1	3.918430	5.233285	1.702855

9a-TS$_{6\text{-}7a}$

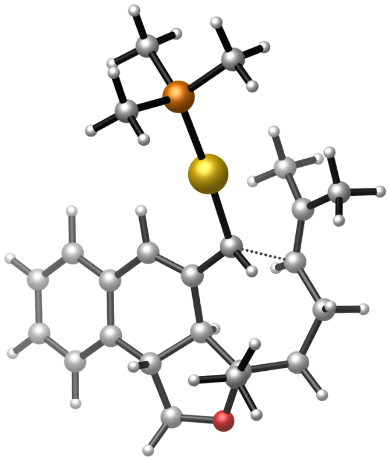

E = -1448.58026045 Hartrees
G = -1448.130782 Hartrees

6	3.707025	5.154749	1.679166
1	4.273309	6.062484	1.912311
1	2.651595	5.428807	1.539027
6	4.237315	4.401772	0.457547
1	5.335660	4.472227	0.438433
6	3.733392	4.745203	-0.919406
6	3.308402	6.016722	-1.297417
1	3.286503	6.817995	-0.563737
6	2.913777	6.271344	-2.616204
1	2.575375	7.265241	-2.894908
6	2.962081	5.254022	-3.573292
1	2.662780	5.453597	-4.598023
6	3.398417	3.980785	-3.210098
1	3.437449	3.185615	-3.950845
6	3.775590	3.704892	-1.884673
6	4.212117	2.366785	-1.497571
1	4.426910	1.660066	-2.296507
6	4.326935	1.963991	-0.200065
6	4.767988	0.614149	0.171308
1	5.311235	0.580894	1.110747
6	3.105541	0.133553	1.292618
1	2.336259	0.767418	0.857513
6	3.423799	0.431709	2.739848
1	4.406966	0.038494	3.017466
1	2.700034	-0.127543	3.350289
6	3.296548	1.923522	3.108808
1	2.258654	2.242520	2.954724
1	3.503510	2.036517	4.178918
6	4.195220	2.903595	2.331777
6	3.846880	2.969539	0.829365
1	2.744013	2.947697	0.788182
6	3.348796	-1.047346	0.603708
6	2.563972	-1.343184	-0.643858
1	3.140440	-1.941024	-1.354187
1	2.222214	-0.427606	-1.134482
1	1.678157	-1.926368	-0.355354
6	4.151522	-2.165513	1.203427
1	3.474251	-2.769176	1.824525
1	4.957513	-1.807526	1.848086
1	4.573306	-2.816056	0.433501
1	2.953324	4.145827	2.766110
6	3.410123	3.250834	0.823466
1	4.054232	3.513453	-0.025145
6	1.968183	3.222335	0.358742
6	1.279636	4.422260	0.138655
1	1.780476	5.370444	0.317705
6	-0.036856	4.418010	-0.321176
1	-0.557231	5.357264	-0.484622
6	-0.674304	3.201516	-0.573493
1	-1.698468	3.185374	-0.935552
6	0.005851	2.002350	-0.360544
1	-0.481222	1.053998	-0.559093
6	1.326791	1.994463	0.102534
6	3.548550	0.816484	0.343224
6	5.667359	-1.045950	4.160538
1	6.522467	-0.959456	4.832006
6	5.739303	-0.193998	2.921956
1	5.002380	-0.510555	2.180826
1	6.722620	-0.310179	2.450168
6	5.520972	1.308475	3.252911
1	4.661020	1.394829	3.927202
1	6.402134	1.700171	3.774710
6	5.247824	2.120405	1.995913
6	3.861862	1.884227	1.385017
1	3.210774	1.682565	2.249735
6	4.662742	-1.861828	4.518143
6	4.725844	-2.655521	5.801620
1	3.877802	-2.409122	6.455571
1	5.651326	-2.472101	6.356881
1	4.656725	-3.733026	5.597330
6	3.400256	-2.078462	3.717645
1	3.295779	-3.137063	3.443096
1	3.353174	-1.488215	2.799451
1	2.518974	-1.827593	4.323677
6	6.450540	2.301666	1.094488
1	6.213779	2.866347	0.190973
1	6.831377	1.320933	0.800205
1	7.244184	2.817098	1.642213
79	3.998638	-1.022292	-2.103056
15	3.555898	-2.435574	-3.930256
6	2.540936	-1.660597	-5.253902
1	2.349692	-2.373768	-6.062799
1	1.588970	-1.325939	-4.831571
1	3.068867	-0.790307	-5.654466
6	2.610733	-3.947974	-3.478879
1	2.397856	-4.553680	-4.366061
1	3.191616	-4.539983	-2.765627
1	1.669348	-3.655560	-3.004211
6	5.031226	-3.078431	-4.819582
1	5.652016	-3.652905	-4.125870
1	4.730552	-3.719546	-5.654931
1	5.619840	-2.238895	-5.200858
6	4.396358	0.230182	-0.518376
1	5.443814	0.496837	-0.385970
8	1.502851	-0.320073	-0.515630
6	1.444607	-1.624285	0.055294
1	1.094104	-2.296172	-0.731161
1	2.431088	-1.960969	0.397033
1	0.738848	-1.649916	0.898555
8	4.990814	3.651022	2.513148

6	5.681884	2.719184	2.670571
1	6.315426	3.364136	2.054802
1	6.027625	1.689593	2.547381
1	5.831369	2.999674	3.717664
8	3.823141	4.237619	2.782503
79	5.836766	-0.549643	-1.238989
15	7.183719	-1.726176	-2.745301
6	8.804884	-0.923348	-3.056820
1	9.393361	-1.516402	-3.764110
1	8.645077	0.078293	-3.465569
1	9.350883	-0.835144	-2.113269
6	7.610850	-3.426620	-2.198679
1	6.694471	-4.005039	-2.051046
1	8.235001	-3.919794	-2.951080
1	8.154021	-3.378773	-1.250650
6	6.442905	-1.949904	-4.410432
1	7.126169	-2.509923	-5.057650
1	5.499620	-2.496144	-4.319438
1	6.241498	-0.970784	-4.854179

9aa-TS_{1a-5}

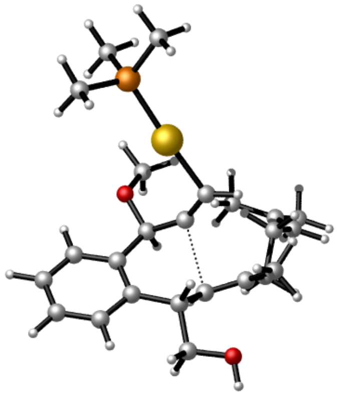

E = -1564.25358617 Hartrees
G = -1563.763505 Hartrees

6	3.693907	4.749701	1.288293
1	3.555200	5.487822	0.488197
1	2.929671	4.935179	2.059226
6	3.518749	3.322059	0.721174
1	4.246180	3.221502	-0.093394
6	2.111250	3.081409	0.149823
6	1.363761	4.165797	-0.337040
1	1.782851	5.164273	-0.311631
6	0.080485	4.012134	-0.861289
1	-0.459446	4.882138	-1.223883
6	-0.498403	2.747187	-0.907994
1	-1.500304	2.607925	-1.303489
6	0.224357	1.650658	-0.441676
1	-0.213843	0.661068	-0.480103
6	1.519203	1.796150	0.072444
6	3.677010	0.523512	0.249252
6	5.588502	-1.335790	4.366147
1	6.484860	-1.139047	4.956809
6	5.478126	-0.519032	3.107597
1	4.704952	-0.907480	2.439049
1	6.424379	-0.579540	2.554011
6	5.162381	0.969879	3.422614
1	4.230592	1.011890	3.996892
1	5.044119	3.721785	3.487994
6	2.022751	0.654252	0.368191
1	1.746344	0.352646	1.396181

9aa-Int1a

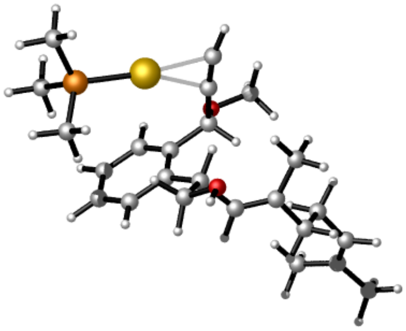

E = -1564.27304488 Hartrees
G = -1563.787559 Hartrees

6	3.627388	3.086344	-0.498327
1	3.334433	3.046163	-1.559118
1	2.884327	3.695661	0.036859
6	3.676495	1.660963	0.072062
1	4.583507	1.233912	-0.364581
6	2.492441	0.793123	-0.348311
6	1.257344	1.361299	-0.689213
1	1.131642	2.438631	-0.652095
6	0.171097	0.571711	-1.074822
1	-0.772048	1.043533	-1.335203
6	0.300605	-0.816095	-1.116406
1	-0.538297	-1.440248	-1.410029
6	1.516539	-1.405285	-0.764274
1	1.624194	-2.483427	-0.776937
6	2.602767	-0.615273	-0.376920
6	4.938620	-1.149239	-1.087330
6	4.683942	-0.222499	5.860181
1	5.561284	0.154889	6.389298
6	4.792011	-0.165556	4.360536
1	3.987396	-0.727302	3.873529
1	5.734395	-0.637697	4.049654
6	4.759078	1.293976	3.832169
1	3.822356	1.757452	4.162943
1	5.579002	1.857163	4.300445
6	4.874334	1.365663	2.324066
6	3.791960	1.654499	1.582652
1	2.859058	1.885407	2.098937
6	3.643280	-0.635872	6.600987
6	3.699084	-0.597186	8.110633
1	2.894626	0.032415	8.516651
1	4.654410	-0.209192	8.479063
1	3.550172	-1.600212	8.535099
6	2.343809	-1.166652	6.044312
1	2.156869	-2.186815	6.407498
1	2.312160	-1.186169	4.952314
1	1.500763	-0.554899	6.394360
6	6.240927	1.052888	1.760668
1	6.311794	1.225876	0.685113
1	6.528023	0.011983	1.961130
1	6.997069	1.682354	2.246989

1	5.957932	1.364132	4.070365
6	5.057088	1.845197	2.196772
6	3.842365	2.326823	1.814593
1	3.011756	2.130387	2.491380
6	4.709544	-2.230251	4.845482
6	4.968752	-2.955885	6.144817
1	4.164727	-2.761953	6.868550
1	5.918513	-2.658650	6.600949
1	4.988871	-4.043624	5.988558
6	3.409850	-2.605743	4.175224
1	3.386856	-3.681131	3.950642
1	3.226966	-2.064165	3.244558
1	2.563530	-2.412014	4.848521
6	6.337813	2.145265	1.480044
1	6.189585	2.619462	0.509154
1	6.949300	1.243336	1.357078
1	6.919099	2.844114	2.093291
79	3.985617	-0.876312	-2.175358
15	3.378101	-2.028675	-4.096782
6	2.369285	-1.036558	-5.263342
1	2.114913	-1.637033	-6.142911
1	1.452064	-0.710978	-4.764917
1	2.934481	-0.153709	-5.574453
6	2.371988	-3.520719	-3.742757
1	2.090122	-4.018627	-4.676219
1	2.950776	-4.209734	-3.121467
1	1.470324	-3.226827	-3.198202
6	4.803425	-2.627156	-5.082598
1	5.406921	-3.306194	-4.473999
1	4.450722	-3.152475	-5.976060
1	5.422102	-1.776341	-5.380777
6	4.673424	0.174015	-0.448935
1	5.737485	0.351961	-0.384099
8	1.633852	-0.606324	-0.104769
6	1.831533	-1.850027	0.568576
1	1.320564	-2.604458	-0.031806
1	2.896921	-2.105249	0.635057
1	1.396601	-1.822045	1.576378
8	5.009919	4.847741	1.816568
1	5.127917	5.752124	2.145794
6	2.214170	0.511742	0.551149
1	2.078887	0.388969	1.634079

9aa-TS$_{1b\text{-}3}$

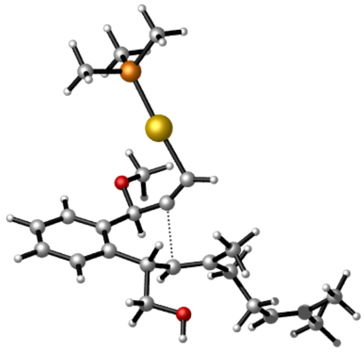

E = -1564.25172581 Hartrees
G = -1563.764369 Hartrees

6	3.788351	4.597356	1.335607
1	3.832612	5.361416	0.549852
79	4.176619	-0.351322	-3.171258
15	2.704295	0.493951	-4.744103
6	2.098214	2.166261	-4.309441
1	1.385416	2.514038	-5.064128
1	1.608098	2.117968	-3.333830
1	2.940824	2.860904	-4.252421
6	1.203148	-0.542347	-4.908271
1	0.523876	-0.098268	-5.643428
1	1.486845	-1.547695	-5.231113
1	0.706984	-0.607002	-3.935931
6	3.412904	0.629835	-6.427188
1	3.733239	-0.357947	-6.769143
1	2.659494	1.028228	-7.114120
1	4.278465	1.297221	-6.406731
6	5.878874	-1.123920	-1.881374
1	6.838180	-1.179211	-2.359724
8	3.698396	-2.664102	0.264823
6	4.777194	-3.298095	0.953382
1	4.446801	-4.315413	1.168767
1	5.683386	-3.335676	0.334172
1	5.002128	-2.776857	1.894195
8	4.936071	3.627500	-0.350568
1	4.894127	4.572740	-0.562269
6	3.919197	-1.298088	-0.001789
1	4.336757	-0.803026	0.887835

9aa-Int3

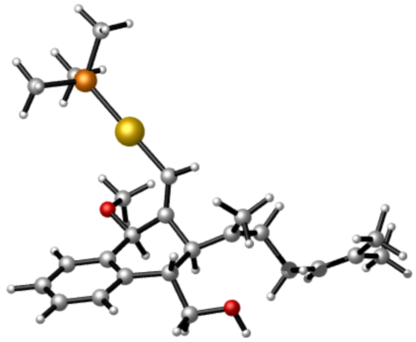

E = -1564.27825180 Hartrees
G = -1563.786203 Hartrees

6	3.381438	4.174910	2.184245
1	3.382078	5.219519	1.851160
1	2.455986	3.997050	2.750144
6	3.436182	3.221445	0.980317
1	4.332340	3.464705	0.397235
6	2.220143	3.395520	0.077319
6	1.965727	4.661702	-0.469449
1	2.649296	5.480551	-0.262113
6	0.860196	4.889168	-1.287816
1	0.684838	5.879763	-1.697576
6	-0.011699	3.838257	-1.577749
1	-0.877893	4.002215	-2.212655
6	0.239291	2.568748	-1.058576
1	-0.420064	1.740965	-1.293407
6	1.350567	2.332876	-0.240332
6	3.140102	0.756242	0.419181
6	5.134709	0.281009	5.698033
1	4.165475	-0.079204	6.042330

1	2.961427	4.855841	2.015324
6	3.522043	3.206803	0.710561
1	4.323568	3.036082	-0.018524
6	2.173587	3.141205	-0.026023
6	1.667248	4.300215	-0.636246
1	2.229971	5.224560	-0.584986
6	0.451886	4.314414	-1.319844
1	0.101009	5.236716	-1.773907
6	-0.302121	3.147681	-1.408152
1	-1.256078	3.139602	-1.927380
6	0.182115	1.978253	-0.824667
1	-0.393019	1.063641	-0.895696
6	1.409551	1.953566	-0.149759
6	3.311417	0.372299	0.251994
6	5.187709	0.578845	5.989802
1	4.188704	0.668994	6.420072
6	5.388167	1.354322	4.715788
1	6.448314	1.444677	4.461612
1	5.007944	2.377389	4.840203
6	4.628490	0.699914	3.527758
1	5.062406	-0.292505	3.346224
1	3.580639	0.550358	3.813589
6	4.716080	1.532554	2.274471
6	3.613317	2.180090	1.814118
1	2.716631	2.109002	2.428647
6	6.065853	-0.220019	6.616612
6	5.687192	-0.945247	7.886781
1	5.789780	-2.032463	7.761936
1	4.658152	-0.731863	8.193874
1	6.356163	-0.664366	8.712346
6	7.478831	-0.481030	6.153258
1	8.200125	-0.165925	6.919963
1	7.734778	0.026816	5.220194
1	7.637377	-1.557916	6.004092
6	6.071679	1.639764	1.642968
1	6.034776	1.981660	0.606375
1	6.605620	0.682936	1.692123
1	6.663146	2.378160	2.196161
79	3.550212	-1.013376	-2.162006
15	2.886868	-1.954958	-4.177152
6	2.031556	-0.763197	-5.278508
1	1.725116	-1.259677	-6.204922
1	1.150484	-0.364632	-4.767844
1	2.707249	0.063869	-5.513584
6	1.712640	-3.349851	-3.974804
1	1.406420	-3.731069	-4.954482
1	2.192882	-4.150508	-3.405547
1	0.831854	-3.005565	-3.425798
6	4.269752	-2.628237	-5.175627
1	4.780405	-3.410913	-4.607798
1	3.890127	-3.046200	-6.113745
1	4.982858	-1.828893	-5.395214
6	4.293467	-0.145892	-0.353171
1	5.363995	-0.160146	-0.207050
8	1.140676	-0.443113	-0.215390
6	1.144954	-1.685938	0.488129
1	0.499032	-2.357226	-0.080038
1	2.155221	-2.111726	0.536917
1	0.747949	-1.563068	1.504642
8	5.032439	4.532686	2.021862
6	5.080294	1.233810	4.543374
1	5.981001	1.844652	4.456582
1	4.231339	1.915552	4.636409
6	4.887692	0.417473	3.184329
1	5.732778	-0.270332	3.099109
1	3.951925	-0.142549	3.262070
6	4.859336	1.358008	2.060165
6	3.562721	1.778451	1.524057
1	2.828366	1.651382	2.332453
6	6.241049	-0.182096	6.306126
6	6.139814	-1.138562	7.468793
1	6.667320	-2.075984	7.245236
1	5.101882	-1.379066	7.718608
1	6.621099	-0.716609	8.361542
6	7.651880	0.180555	5.915284
1	8.185209	0.618333	6.769528
1	7.712610	0.883686	5.081078
1	8.207399	-0.724033	5.633458
6	6.128364	1.828799	1.476662
1	6.051594	1.937412	0.389013
1	6.980051	1.202973	1.748572
1	6.287878	2.846490	1.865723
79	3.448810	-1.093595	-2.022333
15	2.936106	-2.321900	-3.958231
6	2.234217	-1.305127	-5.319389
1	1.994538	-1.931190	-6.185154
1	1.326185	-0.806882	-4.967483
1	2.959465	-0.540831	-5.613035
6	1.685617	-3.645930	-3.701113
1	1.481613	-4.171328	-4.640163
1	2.058268	-4.359194	-2.960216
1	0.759067	-3.202686	-3.324563
6	4.355268	-3.194819	-4.734459
1	4.785355	-3.899591	-4.016876
1	4.030136	-3.738714	-5.627612
1	5.122787	-2.466547	-5.012136
6	3.946406	0.016127	-0.358682
1	5.011985	0.055776	-0.131513
8	1.001074	-0.016532	-0.568515
6	0.713742	-1.258187	0.069854
1	0.267072	-1.903637	-0.689106
1	1.625531	-1.733030	0.452247
1	0.001419	-1.115547	0.895157
8	4.533063	3.905232	2.985479
1	4.421954	4.326790	3.852072
6	1.620130	0.921888	0.280550
1	1.172552	0.822822	1.285533

1	5.215489	5.414145	2.382158
6	1.846926	0.600945	0.436144
1	1.616263	0.556696	1.508087

9aa-Int1b

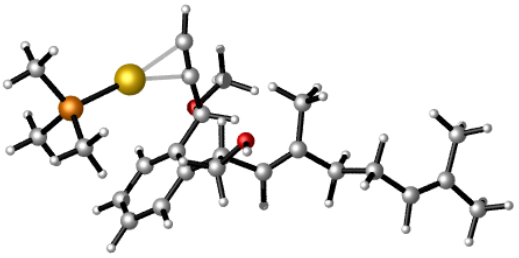

E = -1564.27204468 Hartrees
G = -1563.786146 Hartrees

6	4.030046	2.762885	-0.289692
1	3.829611	2.781576	-1.372180
1	3.506104	3.615338	0.167188
6	3.527119	1.435713	0.301862
1	4.261160	0.701979	-0.042928
6	2.152342	1.021268	-0.215263
6	1.230663	1.971070	-0.677039
1	1.491228	3.024533	-0.665266
6	-0.029492	1.596638	-1.150019
1	-0.720819	2.357373	-1.501145
6	-0.395322	0.251031	-1.163057
1	-1.373386	-0.051425	-1.525663
6	0.501720	-0.711572	-0.696143
1	0.224060	-1.758866	-0.689105
6	1.760734	-0.336137	-0.218717
6	3.807818	-1.676733	-0.718976
6	5.143729	2.168502	6.259841
1	4.325995	2.859276	6.474725
6	5.391634	1.936030	4.793272
1	6.356579	1.448303	4.624892
1	5.434601	2.902075	4.270500
6	4.262852	1.090142	4.149179
1	4.259369	0.103352	4.634855
1	3.296429	1.557301	4.375326
6	4.417267	0.918800	2.652652
6	3.525481	1.473773	1.815610
1	2.694427	2.035980	2.243535
6	5.767836	1.603714	7.305724
6	5.365177	1.934677	8.724143
1	5.048165	1.030628	9.263297
1	4.545481	2.659682	8.762471
1	6.214208	2.348052	9.286858
6	6.899328	0.609185	7.200799
1	7.792683	0.984330	7.719292
1	7.180288	0.374536	6.171191
1	6.626633	-0.332312	7.697583
6	5.630874	0.131425	2.216206
1	5.609163	-0.151769	1.161127
1	5.731202	-0.782687	2.815524
1	6.546074	0.716064	2.371527
79	3.554754	-0.711376	-2.854637
15	2.637473	0.548150	-4.565704
6	2.730594	2.349872	-4.250930

9aa-TS1c-2

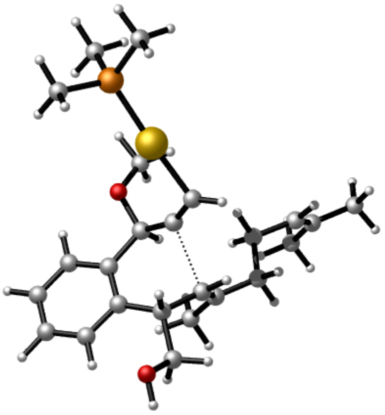

E = -1564.25159275 Hartrees
G = -1563.763481 Hartrees

6	0.634854	-0.672694	-4.637540
1	1.480594	-0.318094	-5.243625
1	-0.227186	-0.025493	-4.859529
6	1.013430	-0.536204	-3.147561
1	1.665649	-1.395530	-2.940409
6	-0.167231	-0.633112	-2.170342
6	-1.342132	-1.281496	-2.586789
1	-1.381721	-1.700546	-3.584380
6	-2.443660	-1.431028	-1.745173
1	-3.332325	-1.939183	-2.109157
6	-2.396270	-0.930505	-0.446283
1	-3.246225	-1.034160	0.222041
6	-1.240203	-0.288791	-0.007884
1	-1.192940	0.108860	0.998880
6	-0.125018	-0.142323	-0.844243
6	2.382327	0.303829	-0.804539
6	3.567415	-0.128088	-0.704578
1	4.419431	-0.149197	-1.369682
79	3.782521	-1.161846	1.155063
15	4.156754	-2.282053	3.153552
6	3.637934	-1.300904	4.614467
1	3.838455	-1.859562	5.534613
1	2.568475	-1.083975	4.543263
1	4.188988	-0.356491	4.633164
6	5.915099	-2.703282	3.456384
1	6.278461	-3.353763	2.656066
1	6.014994	-3.216440	4.418383
1	6.513068	-1.787966	3.466837
6	3.251889	-3.871278	3.295297
1	3.447563	-4.326656	4.271554
1	3.580240	-4.550085	2.503299
1	2.179090	-3.692195	3.183810
8	1.078069	0.490868	1.148303
6	1.653757	1.590115	1.858336
1	2.711615	1.726226	1.599890
1	1.103221	2.517559	1.653028
1	1.575105	1.340289	2.917474
8	0.360556	-2.041171	-4.928002
1	0.093462	-2.099241	-5.859241
6	1.041339	0.661211	-0.261602
1	0.876410	1.725488	-0.480433
6	1.536502	2.012050	-3.007945
6	1.899209	0.696277	-3.026600

1	2.297662	2.899543	-5.093066
1	2.175798	2.579198	-3.337401
1	3.775119	2.644435	-4.118748
6	0.860475	0.179622	-4.811872
1	0.456152	0.803145	-5.616044
1	0.741938	-0.875826	-5.071501
1	0.322406	0.379518	-3.881131
6	3.441398	0.277019	-6.188345
1	3.349518	-0.775645	-6.468793
1	2.960509	0.901448	-6.948036
1	4.501168	0.536329	-6.119418
6	4.754094	-2.016594	-1.428868
1	5.661360	-2.427451	-1.829057
8	2.010523	-2.613543	0.524396
6	2.746550	-3.579456	1.275537
1	2.061761	-4.408198	1.461693
1	3.616308	-3.947631	0.715265
1	3.083858	-3.158208	2.232519
8	5.428050	2.821970	-0.028182
1	5.734215	3.717088	-0.240375
6	2.718911	-1.421279	0.276593
1	3.212017	-1.073798	1.198511
1	2.941886	0.498707	-3.259291
6	0.116944	2.507302	-2.956860
1	-0.077996	3.092484	-3.865537
1	-0.622684	1.709147	-2.898706
1	-0.042278	3.193899	-2.115451
6	2.600528	3.082958	-3.027398
1	2.310279	3.869706	-3.735468
1	3.554225	2.668370	-3.373043
6	2.818192	3.744476	-1.636165
1	3.127739	2.960228	-0.932764
1	1.869294	4.145193	-1.267224
6	3.877469	4.811866	-1.688296
1	4.897798	4.434200	-1.773402
6	3.702012	6.142965	-1.680651
6	4.882035	7.082842	-1.758151
1	4.911949	7.745533	-0.881934
1	5.834939	6.546757	-1.814087
1	4.804049	7.736642	-2.638057
6	2.362739	6.835548	-1.601508
1	1.514868	6.147792	-1.552463
1	2.319487	7.492234	-0.721786
1	2.218519	7.482513	-2.477639

9aa-Int2

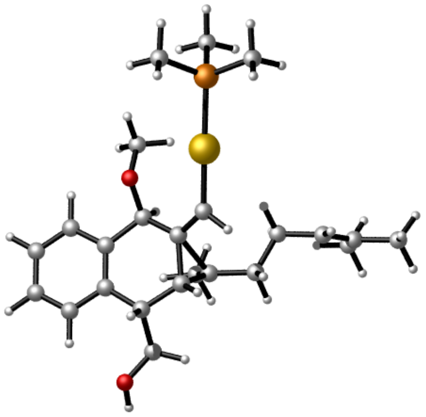

E = -1564.27609729 Hartrees
G = -1563.781710 Hartrees

6	0.038597	0.376007	-4.809825
1	0.919059	0.929962	-5.171039
1	-0.741555	1.109170	-4.562750
6	0.429407	-0.420242	-3.544516
1	0.877091	-1.351337	-3.916500
6	-0.743591	-0.823201	-2.661485
6	-1.872080	-1.398524	-3.276010
1	-1.882737	-1.492884	-4.355869
6	-2.954882	-1.845913	-2.526772
1	-3.814737	-2.282523	-3.027215
6	-2.926035	-1.738776	-1.133332
1	-3.760899	-2.091146	-0.534275
6	-1.814549	-1.177570	-0.515917
1	-1.788355	-1.100890	0.565378
6	-0.717303	-0.714613	-1.262228
6	1.695229	0.205922	-1.286503
6	2.965389	0.128012	-0.757554
1	3.740881	0.374341	-1.484688
79	3.687916	-0.392267	1.076579
15	4.572250	-1.008919	3.170006

9aa-Int1c

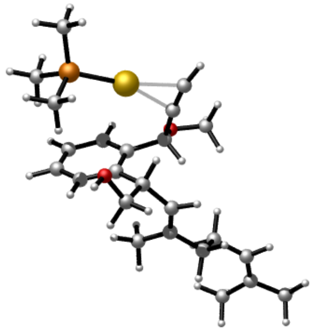

E = -1564.27345491 Hartrees
G = -1563.786711 Hartrees

6	1.171081	-1.184178	-2.726523
1	1.677960	-1.151908	-3.700700
1	0.133537	-0.855183	-2.885682
6	1.893631	-0.220630	-1.778038
1	2.886997	-0.670686	-1.637032
6	1.266139	-0.098105	-0.387477
6	0.023195	-0.672407	-0.086240
1	-0.500197	-1.241762	-0.844060
6	-0.562145	-0.534664	1.173176
1	-1.533824	-0.981060	1.365710
6	0.099035	0.173599	2.176297
1	-0.348956	0.290449	3.158721
6	1.351731	0.726428	1.911353
1	1.880802	1.274163	2.682125
6	1.934594	0.596748	0.645765
6	4.371492	0.111806	0.440531
6	5.353593	-0.630142	0.442059
1	6.330779	-1.064921	0.352345
79	3.718252	-2.064918	1.095161
15	2.409444	-3.863961	1.732122
6	1.176669	-3.398595	3.002856
1	0.565049	-4.268128	3.264283

6	3.833401	-0.085981	4.575627
1	4.248166	-0.439395	5.525227
1	2.749546	-0.231851	4.575989
1	4.043472	0.981279	4.461397
6	6.383538	-0.773275	3.357426
1	6.906873	-1.356973	2.594704
1	6.708970	-1.098683	4.350914
1	6.630881	0.283502	3.221856
6	4.288270	-2.777008	3.577284
1	4.696005	-3.010800	4.566177
1	4.773674	-3.405842	2.825611
1	3.214326	-2.984242	3.566752
8	0.701936	-0.833809	0.679675
6	0.472273	-0.160777	1.917398
1	1.060196	0.763928	1.991904
1	-0.590804	0.079131	2.052988
1	0.789951	-0.847482	2.704862
8	-0.405541	-0.544941	-5.799912
1	-0.803295	-0.038002	-6.525281
6	0.413071	-0.053120	-0.476648
1	0.040083	0.918877	-0.128125
6	1.537152	1.690664	-2.384563
6	1.575366	0.317883	-2.852974
1	2.522751	0.061117	-3.322362
6	0.247526	2.425610	-2.121336
1	0.136078	3.160518	-2.928484
1	-0.634780	1.787184	-2.131463
1	0.279819	2.985920	-1.182855
6	2.763395	2.570987	-2.491868
1	2.533126	3.290134	-3.291260
1	3.624247	1.996402	-2.845607
6	3.142801	3.357958	-1.217258
1	3.286751	2.628293	-0.405116
1	2.313017	4.002177	-0.914868
6	4.410516	4.143825	-1.411998
1	5.304427	3.529427	-1.534929
6	4.555608	5.477122	-1.473483
6	5.916175	6.098414	-1.684547
1	6.170004	6.772705	-0.854891
1	6.706600	5.345604	-1.767702
1	5.927466	6.711313	-2.596519
6	3.431968	6.477125	-1.347303
1	2.447890	6.019686	-1.219680
1	3.608217	7.146364	-0.494162
1	3.392551	7.116802	-2.239397
1	0.541385	-2.602040	2.608309
1	1.693182	-3.031012	3.893597
6	3.374432	-5.243486	2.454461
1	4.099018	-5.606529	1.720618
1	2.700890	-6.059207	2.736370
1	3.910456	-4.889857	3.339274
6	1.456567	-4.586377	0.347307
1	0.792809	-5.371205	0.724774
1	2.151609	-5.012159	-0.381632
1	0.882523	-3.799283	-0.147224
8	3.610963	2.147361	1.413768
6	4.748813	2.960302	1.129124
1	5.675028	2.370182	1.119090
1	4.635542	3.473029	0.163894
1	4.803360	3.700018	1.929448
8	1.226130	-2.499116	-2.171805
1	0.784155	-3.106556	-2.785794
6	3.328452	1.184252	0.422671
1	3.373163	1.643862	-0.576674
6	1.404886	2.226715	-2.509749
6	2.133355	1.097611	-2.507878
1	3.033289	1.085145	-3.123429
6	0.118852	2.438992	-1.740649
1	-0.521643	3.158205	-2.264078
1	-0.445997	1.516829	-1.600588
1	0.319402	2.851176	-0.742959
6	1.904430	3.438748	-3.267616
1	1.115238	3.814408	-3.934008
1	2.758492	3.170611	-3.901546
6	2.322457	4.592037	-2.318445
1	3.088539	4.204199	-1.630491
1	1.468436	4.879877	-1.697652
6	2.879003	5.769584	-3.072058
1	3.853562	5.590663	-3.530764
6	2.310871	6.969703	-3.270777
6	3.010012	8.037167	-4.079998
1	3.169677	8.943303	-3.478468
1	3.982268	7.701208	-4.455238
1	2.397368	8.341601	-4.940355
6	0.961759	7.384743	-2.734734
1	0.463083	6.603753	-2.155785
1	1.057314	8.273103	-2.095004
1	0.294707	7.668894	-3.560368

9ab-Int5

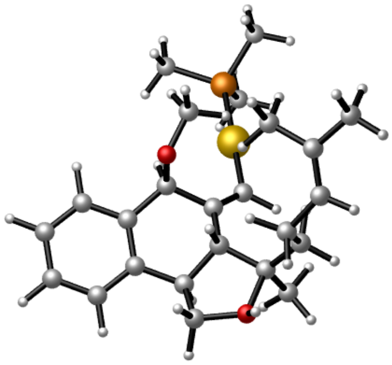

E = -1564.29627700 Hartrees
G = -1563.797108 Hartrees

6	4.120260	3.917468	-3.124795
1	5.056546	4.408494	-3.381328
1	3.265999	4.569637	-3.309198
6	4.065695	3.249553	-1.755375
1	5.062673	2.869810	-1.501681
6	3.573998	4.132195	-0.629468
6	4.003082	5.462502	-0.518606
1	4.692591	5.871827	-1.252967
6	3.558188	6.269134	0.527381
1	3.896378	7.298871	0.598260
6	2.680332	5.746592	1.482075
1	2.329681	6.367855	2.301176
6	2.259267	4.422791	1.379739
1	1.571482	4.011761	2.114411
6	2.699821	3.600995	0.333760
6	2.901449	1.276940	-0.717328
6	3.608230	1.402822	-3.284805
6	3.114874	2.058695	-1.998312
1	2.136160	2.502704	-2.227375
79	2.837404	-1.046312	1.285500
15	2.403017	-2.233848	3.275626
6	3.810271	-3.214877	3.936313
1	3.522980	-3.718063	4.865570
1	4.659012	-2.552482	4.129391
1	4.111182	-3.962841	3.197025
6	1.914113	-1.148582	4.678072
1	1.722034	-1.741627	5.578378
1	1.009674	-0.595288	4.408046
1	2.714878	-0.431055	4.879560
6	1.030560	-3.451684	3.158319
1	0.111952	-2.937220	2.861596
1	0.874075	-3.948508	4.121556
1	1.272699	-4.200711	2.398847
6	3.194988	-0.011245	-0.460221
1	3.653548	-0.575612	-1.273537
8	0.779146	2.288316	-0.076745
6	0.006450	1.149800	0.279568
1	0.069328	0.952184	1.360927
1	0.330152	0.248383	-0.255830
1	-1.028299	1.377568	0.011492
8	4.013213	2.752996	-4.076912
1	3.379304	2.943071	-4.797870
6	2.537416	0.677690	-4.094332
1	2.158654	-0.085291	-3.402599

9ab-TS$_{1a-5}$

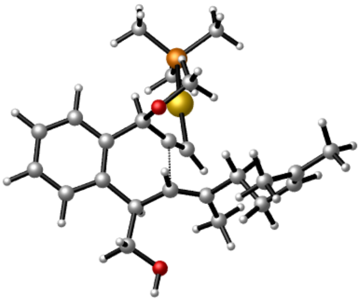

E = -1564.25087812 Hartrees
G = -1563.764848 Hartrees

6	4.332824	4.140615	-2.706203
1	5.208375	4.683698	-2.324956
1	3.560660	4.879922	-2.970723
6	3.787370	3.192912	-1.615873
1	4.619190	2.523812	-1.357696
6	3.357830	3.932221	-0.340417
6	3.790705	5.247601	-0.103831
1	4.426728	5.744481	-0.825693
6	3.427633	5.961515	1.038171
1	3.787079	6.977928	1.171097
6	2.608179	5.370412	1.996687
1	2.313671	5.911860	2.890582
6	2.172575	4.064710	1.790838
1	1.530510	3.593481	2.531135
6	2.530927	3.339649	0.644873
6	2.791706	0.894122	0.004909
6	2.690828	1.220270	-2.863925
6	2.639156	2.379619	-2.171459
1	1.661303	2.834470	-2.039227
79	3.311759	-1.077575	1.857293
15	3.193782	-2.271677	3.843015
6	4.816299	-2.936064	4.376356
1	4.700379	-3.505892	5.304128
1	5.512051	-2.108508	4.539701
1	5.218471	-3.587536	3.595851
6	2.582725	-1.280657	5.259115
1	2.550757	-1.900520	6.161148
1	1.579159	-0.907417	5.037836
1	3.249646	-0.429921	5.423737
6	2.079054	-3.723984	3.746764
1	1.067378	-3.392789	3.496589
1	2.065917	-4.246164	4.708822
1	2.431927	-4.403938	2.966557
6	3.527558	-0.119225	-0.060194
1	4.166664	-0.617748	-0.773120
8	0.635156	2.019849	-0.013350
6	-0.116970	0.810122	0.069484
1	-0.198795	0.464473	1.110408
1	0.331908	0.014553	-0.539111
1	-1.111371	1.041054	-0.316320
8	4.702896	3.346718	-3.825661
1	5.059521	3.943180	-4.501817
6	1.409130	0.603616	-3.374617
1	0.545566	1.110021	-2.930327
1	1.360580	-0.449541	-3.060349

1	3.002427	0.142496	-4.929172
6	4.925066	0.647347	-3.257390
1	5.651177	1.085544	-2.570145
1	5.352314	0.626523	-4.263406
1	4.741369	-0.385251	-2.952564
6	2.177101	2.174394	0.274384
1	2.249016	1.726416	1.276043
6	1.345738	1.515895	-4.609106
1	1.671640	2.161734	-5.439471
1	0.978223	2.179906	-3.822775
6	0.233702	0.630805	-5.115134
1	0.374763	0.236134	-6.121061
6	-0.862350	0.262982	-4.433604
6	-1.888626	-0.655278	-5.052958
1	-2.007700	-1.567637	-4.452414
1	-2.875058	-0.172257	-5.083225
1	-1.619449	-0.948970	-6.072531
6	-1.182594	0.694846	-3.023049
1	-1.273426	-0.184644	-2.371459
1	-0.436914	1.357009	-2.577120
1	-2.154241	1.206213	-2.988580

6	3.956342	0.488441	-3.208648
1	4.825943	0.854185	-2.659661
1	4.182102	0.619296	-4.272869
1	3.840537	-0.590504	-3.036447
6	1.918895	1.942794	0.579211
1	1.802573	1.601964	1.623594
6	1.274994	0.665723	-4.919227
1	2.128445	0.147559	-5.375351
1	1.343831	1.713194	-5.231037
6	-0.002739	0.022563	-5.389145
1	0.006670	-1.068932	-5.374463
6	-1.138516	0.629204	-5.769090
6	-2.347655	-0.173500	-6.188995
1	-3.212887	0.058204	-5.551815
1	-2.645699	0.075719	-7.217224
1	-2.165526	-1.252039	-6.138790
6	-1.339903	2.124768	-5.816863
1	-2.173396	2.417142	-5.163153
1	-0.459093	2.693218	-5.508706
1	-1.615081	2.446088	-6.830984

9ab-Int1a

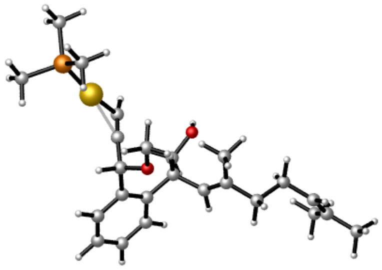

E = -1564.26158377 Hartrees
G = -1563.776581 Hartrees

6	3.936317	2.613645	-2.046390
1	4.666994	2.814607	-1.246838
1	3.897123	3.505657	-2.689972
6	2.554178	2.352995	-1.416565
1	2.621763	1.369766	-0.957064
6	2.270641	3.394745	-0.336945
6	2.154901	4.745091	-0.698795
1	2.247995	5.023709	-1.744499
6	1.926103	5.743725	0.248296
1	1.845031	6.779497	-0.069000
6	1.801002	5.408729	1.596777
1	1.617223	6.175459	2.343333
6	1.909680	4.072634	1.978663
1	1.806698	3.803434	3.026874
6	2.143174	3.068693	1.030035
6	3.622583	1.115134	1.286738
6	0.727248	1.275450	-2.858797
6	1.466180	2.323697	-2.465327
1	1.289774	3.278841	-2.962413
79	4.486793	-0.541690	2.735101
15	4.640986	-2.059720	4.481677
6	6.152679	-3.089704	4.390733
1	6.172042	-3.789482	5.232454
1	7.035974	-2.446763	4.429449
1	6.159019	-3.647379	3.450477

9ab-TS_{1b-3}

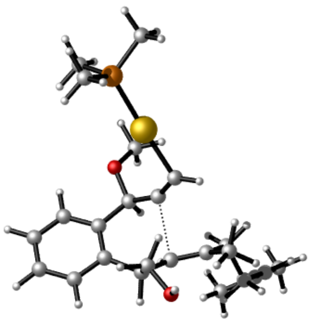

E = -1564.24870522 Hartrees
G = -1563.760591 Hartrees

6	6.796639	3.573175	1.171922
1	6.999206	2.541354	0.858094
1	7.500769	4.227335	0.642253
6	5.363210	3.982405	0.781968
1	5.135573	4.841432	1.431604
6	5.337176	4.530533	-0.653409
6	5.839362	5.829321	-0.834964
1	6.205069	6.371093	0.034241
6	5.878418	6.440173	-2.085084
1	6.273926	7.446712	-2.185978
6	5.393015	5.752080	-3.197306
1	5.405237	6.212343	-4.181023
6	4.882190	4.465541	-3.040956
1	4.498921	3.929769	-3.900407
6	4.856255	3.839019	-1.785637
6	4.962706	1.524265	-0.784889
6	5.750908	0.564053	-0.551751
1	6.087386	0.046542	0.336038
6	0.508811	1.113637	3.137471
1	0.543121	0.126863	3.601792
6	1.840940	1.809314	3.035988
1	2.403602	1.656313	3.966106
1	1.726904	2.891946	2.917497

6	4.677405	-1.262985	6.130083
1	4.765833	-2.026260	6.909871
1	3.756269	-0.693297	6.280385
1	5.530410	-0.581452	6.186654
6	3.237161	-3.235755	4.526106
1	2.301358	-2.682760	4.644320
1	3.360912	-3.929305	5.363894
1	3.202030	-3.797540	3.588905
6	4.761409	0.792709	0.944748
1	5.699553	0.715316	0.428481
8	1.250392	0.842964	0.921414
6	1.077400	-0.455889	1.481113
1	0.932234	-0.405454	2.569567
1	1.934355	-1.108353	1.262280
1	0.185107	-0.873770	1.011827
8	4.293850	1.452462	-2.788967
1	5.114546	1.647217	-3.267394
6	-0.279637	1.445279	-3.979577
1	-0.506206	2.507956	-4.127608
1	-1.226000	0.953400	-3.710946
6	0.830253	-0.120164	-2.292256
1	1.556994	-0.204503	-1.483348
1	1.106871	-0.839525	-3.073952
1	-0.144928	-0.441805	-1.901399
6	2.244020	1.636344	1.540263
1	2.096746	1.640438	2.629772
6	0.215842	0.864419	-5.330324
1	0.445342	-0.202072	-5.199763
1	1.160023	1.354212	-5.590241
6	-0.813618	1.016495	-6.417756
1	-1.685771	0.368867	-6.306328
6	-0.803253	1.866429	-7.457083
6	-1.946359	1.903093	-8.444823
1	-2.402636	2.902650	-8.479990
1	-1.592785	1.688989	-9.463538
1	-2.729742	1.179285	-8.196952
6	0.302702	2.850454	-7.755185
1	-0.094145	3.875016	-7.776962
1	1.117547	2.822517	-7.027898
1	0.729207	2.663353	-8.750655

6	2.679037	1.272191	1.843153
1	2.773851	0.182447	1.934193
1	2.126164	1.472432	0.918203
6	4.055798	1.900158	1.794312
6	4.232640	2.995231	1.002698
1	3.314884	3.353767	0.536319
6	-0.677207	1.551869	2.686295
6	-1.928182	0.720734	2.847430
1	-2.687792	1.265655	3.425403
1	-1.730842	-0.229522	3.354198
1	-2.381458	0.501399	1.870403
6	-0.894632	2.873499	1.989660
1	-1.325315	2.710472	0.992058
1	0.020722	3.457669	1.866643
1	-1.618734	3.487875	2.542268
6	5.082712	1.249372	2.667989
1	5.930166	1.895791	2.890020
1	5.443076	0.320798	2.199290
1	4.617088	0.937405	3.610472
79	6.692333	-0.006215	-2.388420
15	7.787114	-0.712443	-4.308403
6	7.962793	-2.533068	-4.438182
1	8.474654	-2.796077	-5.369702
1	8.539621	-2.905352	-3.587093
1	6.972111	-2.995861	-4.423069
6	6.916639	-0.202370	-5.840726
1	6.850181	0.888521	-5.874425
1	7.457811	-0.563877	-6.721103
1	5.904579	-0.616519	-5.838241
6	9.488506	-0.048891	-4.474625
1	9.940048	-0.398095	-5.408964
1	9.453493	1.044075	-4.472209
1	10.094504	-0.385317	-3.628885
8	4.262671	1.849076	-3.011968
6	3.289358	0.821837	-3.211352
1	3.455842	-0.025558	-2.533826
1	2.273982	1.213830	-3.066616
1	3.412063	0.485919	-4.242225
8	6.943201	3.737753	2.582788
1	7.846249	3.472357	2.820082
6	4.236503	2.435374	-1.719974
1	3.190343	2.507577	-1.391859

9ab-Int3

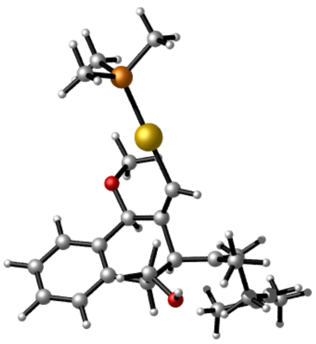

E = -1564.26822499 Hartrees
G = -1563.774696 Hartrees

6	6.779407	3.560314	1.179055

9ab-Int1b

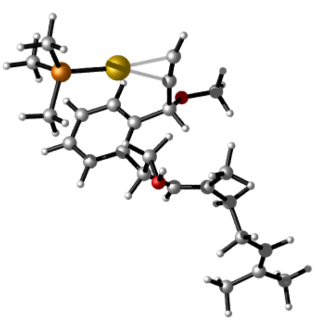

E = -1564.26611105 Hartrees
G = -1563.777769 Hartrees

6	6.376494	1.881071	0.263434
1	6.144417	0.807623	0.252644

1	7.109687	2.600688	0.757821
1	7.396419	4.346459	0.728222
6	5.302124	3.819747	0.827966
1	4.909306	4.451578	1.635017
6	5.185151	4.606058	-0.481832
6	5.578830	5.952563	-0.476676
1	5.944108	6.390062	0.449754
6	5.511133	6.733493	-1.627636
1	5.822300	7.773749	-1.596644
6	5.035294	6.171076	-2.815098
1	4.977104	6.767848	-3.720972
6	4.628846	4.838848	-2.832884
1	4.257464	4.392892	-3.749139
6	4.701823	4.044668	-1.679758
6	4.796808	1.796643	-0.600230
6	5.677783	0.789950	-0.682674
1	6.018917	0.354727	0.257250
6	0.634230	1.176895	3.074913
1	0.562865	0.269366	3.673498
6	2.017364	1.749551	2.960814
1	2.556144	1.639000	3.907092
1	2.005193	2.813564	2.710974
6	2.822016	0.967058	1.829469
1	2.880902	-0.084115	2.127816
1	2.263745	1.062525	0.895667
6	4.156744	1.577174	1.742733
6	4.373050	2.581908	0.702842
1	3.375999	2.978962	0.470981
6	-0.480191	1.648330	2.490016
6	-1.810003	0.967260	2.703484
1	-2.535668	1.663989	3.144607
1	-1.728361	0.095184	3.359439
1	-2.235840	0.640298	1.744933
6	-0.533836	2.857430	1.589944
1	-0.917307	2.572603	0.601026
1	0.433995	3.343517	1.444924
1	-1.233017	3.603228	1.990760
6	5.184830	1.162288	2.698588
1	5.707054	2.034434	3.118021
1	5.975221	0.646803	2.124555
1	4.809585	0.478164	3.460846
79	6.587265	0.007636	-2.361494
15	7.658543	-0.901989	-4.241573
6	8.101766	-2.679776	-4.097018
1	8.592807	-3.027426	-5.011913
1	8.776154	-2.819278	-3.247151
1	7.195588	-3.267751	-3.925169
6	6.653548	-0.812363	-5.778456
1	6.417522	0.233162	-5.997182
1	7.199959	-1.246840	-6.622154
1	5.717400	-1.359216	-5.632800
6	9.240705	-0.079575	-4.688778
1	9.676577	-0.545203	-5.578924
1	9.056677	0.980489	-4.885616
1	9.942712	-0.164234	-3.854327
8	4.459537	2.080864	-3.037217
6	3.536791	1.073579	-3.446729
1	3.515477	0.236476	-2.737923
1	2.525697	1.492437	-3.549612
1	3.882984	0.710265	-4.416146

1	7.196797	2.042246	-0.451873
6	5.173085	2.721855	-0.187477
1	5.461045	3.753930	0.058692
6	5.029021	2.690457	-1.715145
6	5.369700	3.847247	-2.435231
1	5.716199	4.720027	-1.887726
6	5.264796	3.910797	-3.824560
1	5.533656	4.824936	-4.345999
6	4.802743	2.801082	-4.535565
1	4.705014	2.837949	-5.616531
6	4.460504	1.641448	-3.843576
1	4.096268	0.772344	-4.381636
6	4.568412	1.576083	-2.446178
6	5.335508	-0.729686	-1.891990
6	6.129546	-1.669646	-1.937182
1	6.637473	-2.611168	-1.851166
6	0.209569	2.383062	3.547476
1	-0.109007	1.425482	3.963863
6	1.656298	2.435821	3.131793
1	2.274002	1.975105	3.914864
1	2.012332	3.466008	3.025548
6	1.900191	1.697021	1.790585
1	1.507796	0.672836	1.874304
1	1.313198	2.196995	1.011615
6	3.367903	1.643513	1.405246
6	3.818314	2.495961	0.467329
1	3.073236	3.170648	0.042305
6	-0.719472	3.343705	3.419239
6	-2.145411	3.114641	3.863263
1	-2.442127	3.849052	4.625526
1	-2.292472	2.112779	4.280011
1	-2.843221	3.242198	3.023449
6	-0.464250	4.712519	2.834998
1	-1.120980	4.885807	1.971149
1	0.567104	4.858927	2.505103
1	-0.702759	5.497070	3.566652
6	4.175137	0.596748	2.144504
1	5.197914	0.922176	2.337762
1	4.207965	-0.346642	1.579851
1	3.705579	0.362745	3.106015
79	7.340143	0.017309	-2.867965
15	8.909033	1.448799	-3.787950
6	8.460668	2.014286	-5.470424
1	9.207960	2.725866	-5.836091
1	8.410646	1.154959	-6.144714
1	7.480082	2.495439	-5.432610
6	9.105191	2.973873	-2.792020
1	9.434190	2.712204	-1.782645
1	9.845469	3.630516	-3.260573
1	8.141342	3.486226	-2.730817
6	10.583298	0.719624	-3.924857
1	11.273220	1.455805	-4.349495
1	10.933236	0.420602	-2.933156
1	10.546697	-0.161980	-4.570138
8	3.050623	-0.276356	-2.392852
6	2.445710	-1.344775	-1.663239
1	3.101651	-2.224770	-1.616585
1	2.188738	-1.029173	-0.642690
1	1.534453	-1.606243	-2.203630
8	6.758623	2.317443	1.564202

8	6.917418	3.593977	2.603873
1	7.829311	3.343636	2.823403
6	4.193229	2.600980	-1.755011
1	3.097435	2.622103	-1.613585
1	7.502894	1.767820	1.857526
6	4.209868	0.251184	-1.780205
1	4.028269	0.412102	-0.711277

9ab-TS$_{1c-2}$

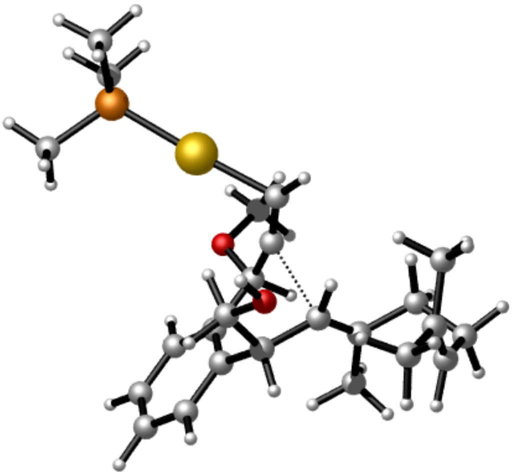

E = -1564.25648322 Hartrees
G = -1563.767707 Hartrees

6	2.536190	-0.079113	-3.381497
6	2.923392	-1.030991	-4.339193
1	3.778462	-0.809203	-4.973065
6	2.249386	-2.238760	-4.500121
1	2.578656	-2.949015	-5.253027
6	1.147340	-2.517615	-3.692753
1	0.600589	-3.449371	-3.804711
6	0.737747	-1.584230	-2.742595
1	-0.126773	-1.790869	-2.123816
6	1.422180	-0.372240	-2.565286
6	1.788530	1.478187	-0.842816
6	2.396121	1.957701	0.147407
1	2.999176	2.817402	0.395583
79	2.425465	0.278526	1.497385
15	2.578505	-1.446995	3.040879
6	3.750635	-1.122960	4.412105
1	3.771729	-1.977589	5.096414
1	3.438191	-0.227522	4.956387
1	4.751427	-0.957627	4.003920
6	3.131849	-3.027023	2.291877
1	2.433876	-3.318685	1.502233
1	3.170813	-3.811713	3.054490
1	4.124908	-2.893578	1.853925
6	0.976200	-1.829087	3.847045
1	1.098486	-2.657488	4.552259
1	0.243303	-2.104142	3.083502
1	0.615649	-0.945426	4.380696
8	0.072410	-0.115389	-0.587485
6	-0.927190	0.648112	0.090452
1	-0.477091	1.418722	0.729125
1	-1.612528	1.119108	-0.626386
1	-1.476387	-0.059452	0.713585
6	0.814884	0.610638	-1.557230
1	0.131929	1.283672	-2.096414

9ab-Int2

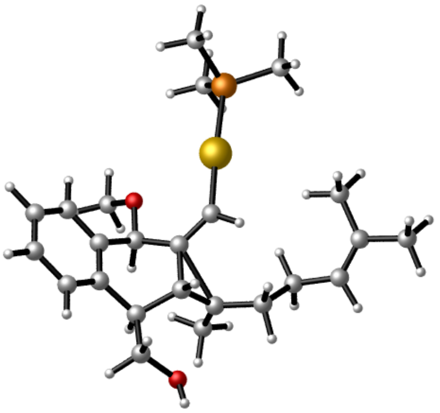

E = -1564.28730302 Hartrees
G = -1563.794081 Hartrees

6	2.615265	4.653731	-2.734027
1	2.240781	5.678813	-2.605780
1	1.935583	4.123505	-3.417240
6	2.666008	3.945713	-1.382693
1	3.335559	4.543777	-0.752536
6	1.351873	3.790747	-0.646928
6	0.137299	4.342453	-1.059316
1	0.083549	4.942297	-1.962708
6	-1.024572	4.124275	-0.311368
1	-1.964166	4.560593	-0.638025
6	-0.978177	3.342984	0.843859
1	-1.882077	3.166657	1.420055
6	0.232313	2.779595	1.259728
1	0.275572	2.154400	2.145220
6	1.394104	3.010752	0.525854
6	3.196706	1.617176	-0.336785
6	4.750244	2.413228	-1.571009
6	3.294529	2.544743	-1.588868
1	2.842991	2.057360	-2.454647
79	3.246116	-1.169466	1.028177
15	3.168367	-2.891934	2.622327
6	3.841823	-2.429114	4.268405
1	3.755740	-3.267217	4.967775
1	3.288985	-1.570049	4.659513
1	4.894308	-2.149754	4.164399
6	1.475725	-3.511409	2.979881
1	1.511682	-4.313087	3.724912
1	1.026068	-3.891592	2.058166
1	0.859022	-2.690497	3.356928
6	4.112889	-4.396799	2.153133
1	3.724958	-4.789352	1.208863
1	4.024512	-5.162871	2.930525
1	5.166855	-4.137663	2.016761
6	3.371041	0.273617	-0.423882
1	3.627649	-0.081996	-1.422537
8	2.677927	1.564885	2.037890

6	1.681461	3.081955	-3.326674
6	2.770196	2.455144	-2.813136
1	3.394187	3.042379	-2.149946
6	1.372209	4.487506	-2.870001
1	1.828140	4.659009	-1.889356
1	0.289004	4.625469	-2.754892
6	0.807162	2.530502	-4.418410
1	0.861163	3.191985	-5.293991
1	1.091639	1.526374	-4.736335
1	-0.248127	2.517283	-4.112931
6	4.631747	0.802302	-2.383063
1	4.281813	0.474474	-1.394199
1	5.160450	-0.040942	-2.849666
6	3.418245	1.162782	-3.269639
1	3.830078	1.358902	-4.269683
8	5.477674	1.944791	-2.274982
1	6.252517	1.695412	-1.748053
6	1.910349	5.565487	-3.863965
1	1.262829	5.590093	-4.747499
1	1.802863	6.543225	-3.377187
6	3.336542	5.316378	-4.302030
1	3.453739	4.921964	-5.311579
6	4.443452	5.435909	-3.553868
6	4.443312	5.915735	-2.122965
1	3.484960	6.339385	-1.810653
1	4.683706	5.083692	-1.444759
1	5.218214	6.677882	-1.967130
6	5.793548	5.010655	-4.077744
1	5.756335	4.750325	-5.141051
1	6.545607	5.799812	-3.941473
1	6.142873	4.127152	-3.525834
6	2.911867	2.252025	3.265261
1	2.180826	3.055240	3.424333
1	3.924759	2.677351	3.292174
1	2.811244	1.509289	4.059485
8	3.953881	4.653422	-3.229221
1	3.946381	4.988826	-4.138929
6	5.419319	1.446329	-2.486027
1	4.680784	0.866217	-3.044014
1	5.924066	2.090857	-3.225945
6	5.615781	3.315468	-0.765656
1	5.154219	3.669765	0.156112
1	5.770364	4.198949	-1.404699
1	6.587511	2.875238	-0.541338
6	2.741624	2.392618	0.901255
1	3.456623	3.207357	1.099372
6	6.472021	0.497706	-1.843311
1	7.338673	1.084360	-1.516867
1	6.031464	0.052363	-0.946977
6	6.912685	-0.551793	-2.825451
1	7.515519	-0.177617	-3.654048
6	6.620176	-1.862617	-2.803174
6	5.786452	-2.550331	-1.748546
1	5.460568	-1.894003	-0.938402
1	4.885739	-2.987847	-2.201559
1	6.345584	-3.384895	-1.303720
6	7.129512	-2.784735	-3.885871
1	7.754285	-3.581032	-3.458174
1	6.294080	-3.285380	-4.394684
1	7.719800	-2.253009	-4.638832

9ab-Int1c

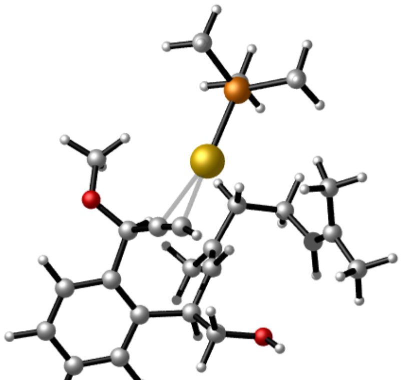

E = -1564.26731024 Hartrees
G = -1563.776094 Hartrees

6	0.224472	-1.332453	-1.928246
6	-0.437224	-2.515839	-2.300241
1	0.149603	-3.319074	-2.739598
6	-1.807151	-2.693368	-2.124632
1	-2.276533	-3.625249	-2.426939
6	-2.564568	-1.665695	-1.563240
1	-3.634619	-1.781587	-1.417527
6	-1.939270	-0.475160	-1.200613
1	-2.523282	0.339459	-0.790422
6	-0.560669	-0.292827	-1.380470
6	0.848511	0.988101	0.211877

9ab-TS$_{2-5}$

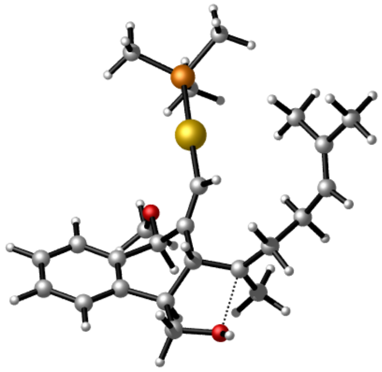

E = -1564.28489795 Hartrees
G = -1563.789574 Hartrees

6	2.772097	4.572784	-2.645751
1	2.492716	5.630593	-2.583047
1	2.115603	4.073004	-3.367224
6	2.700493	3.895233	-1.281845
1	3.333134	4.486361	-0.608498
6	1.358548	3.734849	-0.616937
6	0.159327	4.268431	-1.090763
1	0.144584	4.864296	-1.999032
6	-1.031396	4.037478	-0.394194
1	-1.962169	4.458930	-0.762859
6	-1.023365	3.261394	0.766147
1	-1.949649	3.074994	1.302375

6	1.468639	0.827774	1.263523
1	1.835586	0.439716	2.194714
79	2.380662	2.772031	0.557851
15	3.408401	4.837402	0.308737
6	5.078538	4.950999	1.049243
1	5.479406	5.957488	0.893257
1	5.742566	4.217918	0.586390
1	5.013537	4.745643	2.121136
6	2.426833	6.166317	1.100434
1	1.426478	6.187789	0.659347
1	2.915706	7.134330	0.950335
1	2.338144	5.963649	2.171097
6	3.585455	5.342986	-1.443650
1	4.044212	6.335375	-1.497963
1	2.597414	5.371113	-1.910973
1	4.207794	4.623324	-1.980003
8	-1.012567	2.032607	-0.798249
6	-0.765744	3.308377	-1.385305
1	0.098624	3.807723	-0.923932
1	-0.597593	3.220438	-2.467347
1	-1.659743	3.907975	-1.204007
6	0.016965	1.083492	-1.015842
1	0.670818	1.403958	-1.833794
6	2.593445	0.669165	-3.440989
6	2.462472	-0.029962	-2.303346
1	2.956587	0.366552	-1.418736
6	3.413188	1.942372	-3.434032
1	3.451370	2.334048	-2.410540
1	2.930178	2.709462	-4.055952
6	1.998363	0.261943	-4.764117
1	2.775910	0.190654	-5.536247
1	1.472480	-0.695147	-4.719267
1	1.283203	1.020611	-5.110650
6	2.374356	-2.113036	-0.917981
1	2.208789	-1.545677	0.009834
1	1.871246	-3.084761	-0.808933
6	1.751832	-1.350789	-2.104848
1	1.947105	-1.973902	-2.986471
8	3.763213	-2.277492	-1.183536
1	4.166361	-2.715398	-0.418275
6	4.872977	1.742444	-3.934046
1	4.856349	1.489994	-5.000665
1	5.390502	2.708115	-3.856542
6	5.604976	0.661783	-3.173122
1	5.672739	-0.303838	-3.672462
6	6.081664	0.745032	-1.922154
6	5.998603	1.994223	-1.079442
1	5.778242	2.887739	-1.670142
1	5.207153	1.891740	-0.320795
1	6.937069	2.170161	-0.537240
6	6.679549	-0.452385	-1.225452
1	6.727839	-1.325111	-1.883816
1	7.691370	-0.236508	-0.855071
1	6.071504	-0.730478	-0.352902
6	0.174399	2.715924	1.239945
1	0.184319	2.094535	2.129110
6	1.366426	2.958948	0.559727
6	3.231738	1.580470	-0.220169
6	4.794491	2.564420	-1.741494
6	3.345732	2.496313	-1.472030
1	2.838154	2.003872	-2.306247
79	3.300761	-1.201484	1.130754
15	3.139336	-2.918142	2.725608
6	3.792988	-2.470862	4.384740
1	3.671385	-3.303758	5.085133
1	3.255584	-1.596117	4.762623
1	4.854271	-2.219432	4.300436
6	1.419531	-3.477086	3.057762
1	1.412053	-4.274974	3.807891
1	0.972120	-3.845766	2.130243
1	0.826276	-2.632031	3.419005
6	4.036608	-4.463840	2.294334
1	3.656427	-4.853196	1.345481
1	3.901074	-5.217385	3.077251
1	5.102756	-4.246824	2.180859
6	3.459757	0.256657	-0.307641
1	3.756944	-0.081839	-1.303446
8	2.593084	1.530133	2.127268
6	2.771489	2.222748	3.359257
1	2.035378	3.028316	3.482090
1	3.782619	2.647901	3.431059
1	2.634160	1.485148	4.153074
8	4.153297	4.460450	-3.039150
1	4.209688	4.370912	-4.004142
6	5.404463	1.638000	-2.728175
1	4.636222	1.054888	-3.240960
1	5.916687	2.249380	-3.482966
6	5.727505	3.333266	-0.874764
1	5.248527	4.125381	-0.302524
1	6.536925	3.752558	-1.479257
1	6.190728	2.629851	-0.169669
6	2.705377	2.353830	0.990089
1	3.411625	3.172483	1.214608
6	6.478178	0.681114	-2.092169
1	7.362234	1.276738	-1.837224
1	6.081904	0.273822	-1.159827
6	6.844475	-0.404742	-3.060094
1	7.357453	-0.060732	-3.959139
6	6.589622	-1.719227	-2.942886
6	5.879219	-2.369810	-1.780089
1	5.616301	-1.683123	-0.972760
1	4.950839	-2.848187	-2.121853
1	6.501379	-3.169377	-1.355436
6	7.022243	-2.684424	-4.021038
1	7.706452	-3.441625	-3.614148
1	6.157218	-3.230930	-4.421549
1	7.524112	-2.179668	-4.852505

9b-Int7b

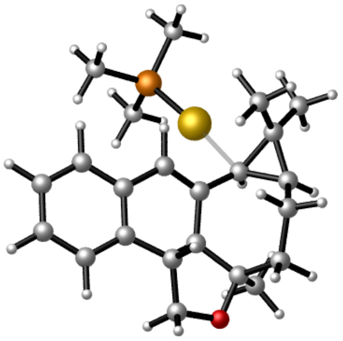

E = -1448.59953985 Hartrees
G = -1448.145193 Hartrees

6	1.496944	1.400805	-4.047265
1	0.471179	1.598402	-3.703821
1	1.478422	1.263068	-5.133642
6	2.107289	0.220764	-3.295562
1	2.966486	-0.151847	-3.875530
6	1.279615	-0.975076	-2.912360
6	0.130510	-1.387733	-3.582624
1	-0.252583	-0.796665	-4.410305
6	-0.529542	-2.562744	-3.202022
1	-1.429152	-2.871314	-3.726964
6	-0.025425	-3.340204	-2.157183
1	-0.529443	-4.257618	-1.866653
6	1.131014	-2.938817	-1.485051
1	1.525131	-3.542455	-0.670361
6	1.788021	-1.752378	-1.842351
6	2.977888	-1.296932	-1.111430
1	3.465408	-2.025677	-0.468099
6	3.432764	-0.023471	-1.171769
6	4.573154	0.536129	-0.370737
1	4.311880	1.374122	0.296309
6	5.779226	1.037780	-1.250787
1	6.166457	2.008296	-0.945083
6	5.610264	0.889627	-2.747900
1	6.598631	0.958629	-3.219341
1	5.228153	-0.107221	-2.985537
6	4.686106	1.956336	-3.371619
1	4.501879	1.678567	-4.413934
1	5.211345	2.918590	-3.403431
6	3.300969	2.189687	-2.690674
6	2.663082	0.932595	-2.057427
1	1.800102	1.292969	-1.470357
6	6.039325	-0.070060	-0.307414
6	6.263295	-1.492310	-0.789183
1	6.042330	-2.202731	0.015528
1	5.651059	-1.754804	-1.652017
1	7.316806	-1.610784	-1.068215
6	6.895867	0.294167	0.900329
1	7.948974	0.313734	0.594611
1	6.641505	1.282211	1.297310
1	6.791973	-0.439594	1.707956
6	3.338813	3.397764	-1.753535
1	2.366885	3.540686	-1.270328
1	4.104091	3.285282	-0.979812
1	3.573540	4.299456	-2.328659

9b-Int6

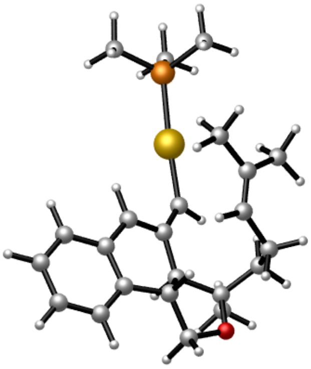

E = -1448.58346222 Hartrees
G = -1448.134787 Hartrees

6	3.141845	4.746298	-1.886322
1	3.640236	5.723787	-1.791479
1	2.098155	4.910056	-2.169127
6	3.318675	3.916189	-0.621488
1	2.628875	3.067521	-0.686016
6	3.147089	4.504725	0.746469
6	2.353901	5.605098	1.040277
1	1.863484	6.151249	0.240660
6	2.170666	6.006005	2.371415
1	1.553000	6.873617	2.584132
6	2.755991	5.298042	3.431301
1	2.591554	5.616643	4.455202
6	3.543136	4.189352	3.159474
1	4.010321	3.631463	3.966081
6	3.771251	3.791390	1.819006
6	4.636747	2.697836	1.531904
1	4.972023	2.075771	2.359024
6	5.162565	2.427711	0.255619
6	6.134441	1.442895	0.123272
1	6.621551	1.441330	-0.854990
6	2.871872	0.495686	-1.061788
1	2.230264	1.078447	-0.400871
6	3.070699	1.040748	-2.459920
1	2.894823	0.234482	-3.183727
1	2.312616	1.802126	-2.673153
6	4.457321	1.636232	-2.798067
1	4.538976	1.656595	-3.891888
1	5.242909	0.957735	-2.449745
6	4.788238	3.077568	-2.349285
6	4.751963	3.405914	-0.828972
1	5.405347	4.283065	-0.690717
6	3.380458	-0.640071	-0.558223
6	3.085042	-1.065272	0.858826
1	4.015770	-1.145581	1.438965
1	2.427063	-0.356932	1.373414
1	2.612587	-2.057111	0.884507
6	4.296320	-1.566686	-1.318032
1	3.953719	-2.606812	-1.236382
1	4.379074	-1.317406	-2.379169
1	5.307865	-1.534314	-0.885428
6	6.126458	3.501080	-2.969994
1	6.334058	4.552311	-2.745998
1	6.955443	2.896975	-2.583471

8	2.335775	2.526986	-3.728976
79	3.846072	-0.320207	1.781587
15	2.988700	-1.149812	3.725017
6	4.277998	-1.639851	4.928252
1	3.808162	-2.024546	5.839309
1	4.893830	-0.770808	5.174509
1	4.912470	-2.413657	4.487912
6	1.964886	-2.639011	3.431300
1	1.143172	-2.383354	2.757034
1	1.560442	-3.007961	4.379389
1	2.576623	-3.416732	2.966872
6	1.904214	0.036055	4.601444
1	1.525929	-0.425166	5.519774
1	1.065450	0.307804	3.955129
1	2.470582	0.937238	4.850360

1	6.094389	3.381895	-4.057438
8	3.755283	3.947731	-2.902852
79	6.707226	-0.043947	1.396393
15	7.338219	-1.867573	2.756464
6	9.110704	-2.327521	2.628955
1	9.327782	-3.189667	3.267710
1	9.729844	-1.480706	2.938610
1	9.347543	-2.574714	1.590135
6	6.420562	-3.404341	2.344013
1	5.347954	-3.230283	2.467923
1	6.735237	-4.223483	2.998742
1	6.612643	-3.675385	1.302044
6	7.041601	-1.620445	4.551099
1	7.339398	-2.512480	5.111684
1	5.978864	-1.423610	4.719573
1	7.618855	-0.760772	4.902929

9b-TS$_{6\text{-}7b}$

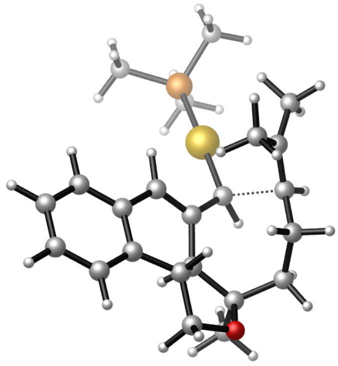

E = -1448.57804943 Hartrees
G = -1448.127928 Hartrees

6	2.186350	1.175532	-4.573244
1	1.138617	1.516453	-4.554983
1	2.448245	0.896954	-5.597392
6	2.426398	0.086475	-3.538982
1	3.428194	-0.327008	-3.732804
6	1.484677	-1.071160	-3.354347
6	0.640787	-1.570430	-4.342397
1	0.598019	-1.083682	-5.312944
6	-0.152244	-2.697229	-4.091745
1	-0.815867	-3.071448	-4.866027
6	-0.086348	-3.344657	-2.854526
1	-0.696136	-4.223217	-2.665724
6	0.767004	-2.862463	-1.863040
1	0.823882	-3.363668	-0.899766
6	1.549744	-1.718216	-2.091944
6	2.436026	-1.192286	-1.059601
1	2.640733	-1.828803	-0.200903
6	2.953207	0.077458	-1.085564
6	3.795112	0.598854	-0.013951
1	3.786489	1.691566	-0.009231
6	5.611734	0.743249	-0.757787
1	5.974494	1.505738	-0.068095
6	5.502746	1.232889	-2.194269
1	6.528901	1.457961	-2.513733
1	5.148757	0.442136	-2.859027
6	4.636798	2.491242	-2.355267

9b-Int7a

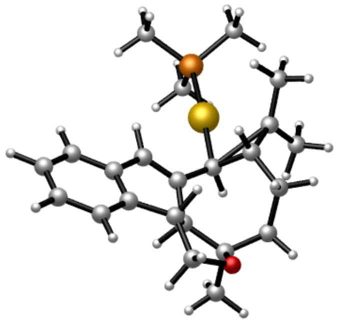

E = -1448.57750726 Hartrees
G = -1448.124763 Hartrees

6	2.890650	4.825132	-1.768256
1	3.503079	5.737776	-1.846121
1	1.843264	5.088075	-1.938920
6	3.155990	4.094036	-0.461423
1	2.397900	3.298210	-0.376565
6	3.246638	4.804712	0.863838
6	2.663807	6.039681	1.137787
1	2.127929	6.568314	0.353529
6	2.764681	6.603830	2.415767
1	2.316254	7.572900	2.616188
6	3.435379	5.917247	3.430023
1	3.508359	6.348724	4.424539
6	4.012745	4.672514	3.167748
1	4.532180	4.136152	3.958756
6	3.938843	4.105477	1.887984
6	4.513064	2.784945	1.586636
1	4.711624	2.114543	2.420927
6	4.764613	2.387693	0.321149
6	5.099054	1.015637	-0.144122
1	5.865653	1.021948	-0.924436
6	3.822410	0.329990	-0.686327
1	3.030613	0.512991	0.045574
6	3.324060	0.780685	-2.084356
1	3.097475	-0.093492	-2.706322
1	2.375097	1.312565	-1.955401
6	4.290978	1.697027	-2.840023

1	5.033632	3.106411	-3.168993
1	4.717607	3.106125	-1.450137
6	3.149107	2.265903	-2.703405
6	2.480898	0.928251	-2.255387
1	1.433749	1.177870	-2.017373
6	6.015723	-0.559322	-0.433338
6	5.792670	-1.690823	-1.364599
1	6.178909	-2.634420	-0.974740
1	4.703867	-1.792285	-1.533574
1	6.226990	-1.494377	-2.351143
6	6.675636	-0.862217	0.869105
1	7.716287	-1.145062	0.647059
1	6.682306	-0.012555	1.554939
1	6.215831	-1.729400	1.356657
6	2.325991	3.473859	-2.240063
1	1.308273	3.418728	-2.640418
1	2.264507	3.507141	-1.145452
1	2.784525	4.404757	-2.590187
8	3.084604	2.207864	-4.160721
79	3.626943	-0.109462	1.951936
15	3.381110	-0.850859	4.164195
6	4.880898	-1.689652	4.815317
1	4.717106	-2.027753	5.843761
1	5.724263	-0.993535	4.792156
1	5.119293	-2.549908	4.183125
6	2.030312	-2.073474	4.399691
1	1.078236	-1.626240	4.099591
1	1.972028	-2.384159	5.447960
1	2.219476	-2.948674	3.771124
6	3.027713	0.473262	5.386793
1	2.938281	0.051196	6.393152
1	2.093682	0.975499	5.119680
1	3.837766	1.208278	5.371558

1	3.932247	1.805601	-3.870155
1	5.278982	1.229633	-2.918707
6	4.452526	3.141740	-2.313446
6	4.504624	3.426151	-0.767972
1	5.274833	4.200541	-0.629826
6	4.310202	-1.077780	-0.454780
6	3.448395	-2.006812	0.352006
1	3.980108	-2.904828	0.674067
1	2.987029	-1.512341	1.210080
1	2.633974	-2.320720	-0.322850
6	5.180966	-1.719447	-1.496972
1	4.506121	-2.086428	-2.286489
1	5.878113	-1.019592	-1.961711
1	5.726353	-2.582568	-1.106859
6	5.671604	3.776651	-2.999498
1	5.703801	4.853588	-2.803304
1	6.602476	3.333173	-2.625745
1	5.623762	3.625678	-4.083101
8	3.260472	3.856688	-2.753616
79	5.876297	-0.401639	1.220723
15	6.895833	-1.947141	2.757594
6	8.711997	-2.102334	2.539901
1	9.120622	-2.824349	3.254327
1	9.183322	-1.128409	2.698626
1	8.929318	-2.436420	1.521357
6	6.260907	-3.663344	2.608808
1	5.193602	-3.680197	2.846829
1	6.794930	-4.327169	3.296384
1	6.401797	-4.013801	1.582110
6	6.667480	-1.507716	4.525172
1	7.150611	-2.250964	5.167536
1	5.599369	-1.466348	4.756928
1	7.107084	-0.524347	4.715293

9b-TS$_{6\text{-}7a}$

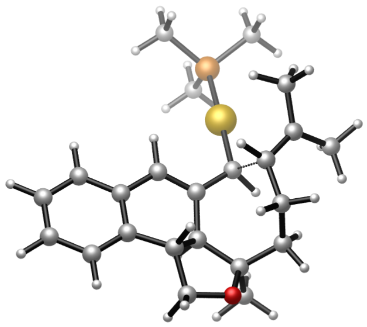

E = -1448.57198018 Hartrees
G = -1448.120759 Hartrees

6	3.315047	5.015649	-1.768723
1	4.066828	5.815545	-1.673778
1	2.353611	5.464956	-2.031184
6	3.287004	4.138165	-0.526812
1	2.404529	3.484936	-0.611142
6	3.301457	4.705563	0.866021
6	2.848402	5.976322	1.207646
1	2.498166	6.651635	0.431740
6	2.841492	6.389164	2.546171
1	2.497560	7.387818	2.800017
6	3.266603	5.518267	3.553476

9ba-Int5

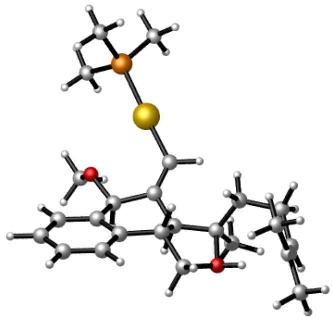

E = -1564.29916745 Hartrees
G = -1563.800204 Hartrees

6	4.229104	2.976869	2.922773
1	5.043864	3.699333	2.959148
1	3.397599	3.297900	3.549112
6	3.793794	2.536220	1.527341
1	4.653576	2.588314	0.849165
6	2.659451	3.322993	0.913301
6	2.640186	4.721725	0.996705
1	3.433137	5.239885	1.530981
6	1.623943	5.459406	0.391656
1	1.619504	6.542881	0.468161
6	0.619876	4.794851	-0.316858

1	3.251434	5.836702	4.591701
6	3.707937	4.237207	3.224100
1	4.036689	3.555342	4.004717
6	3.745677	3.818081	1.883300
6	4.206635	2.482871	1.519159
1	4.270403	1.737181	2.309115
6	4.579285	2.143957	0.249116
6	5.016010	0.790472	-0.109070
1	5.566652	0.773172	-1.048830
6	3.304939	0.214852	-0.915563
1	2.638870	0.433296	-0.084912
6	3.009654	1.032708	-2.181854
1	2.532455	0.354407	-2.898966
1	2.246855	1.773421	-1.925734
6	4.166262	1.751302	-2.892046
1	3.850159	1.973463	-3.917154
1	5.033618	1.090599	-2.987476
6	4.608190	3.104455	-2.299975
6	4.544856	3.287410	-0.753098
1	5.398705	3.931141	-0.486239
6	3.732998	-1.116370	-0.950965
6	3.358486	-2.065810	0.138552
1	4.167860	-2.763333	0.376210
1	3.024683	-1.554815	1.044331
1	2.518407	-2.667252	-0.244023
6	4.573233	-1.666418	-2.051601
1	4.300277	-2.706326	-2.258878
1	4.528039	-1.083873	-2.972830
1	5.617843	-1.682707	-1.702624
6	5.999582	3.461300	-2.837229
1	6.251966	4.495786	-2.581920
1	6.764350	2.804548	-2.405022
1	6.027792	3.358290	-3.927162
8	3.658621	4.090078	-2.804031
79	5.957094	-0.407145	1.337952
15	7.097781	-1.702251	2.924562
6	8.911710	-1.810692	2.657533
1	9.371797	-2.441290	3.425303
1	9.347046	-0.808216	2.700522
1	9.109882	-2.237547	1.670222
6	6.535625	-3.450590	2.978937
1	5.468789	-3.482575	3.218148
1	7.095374	-4.007307	3.737588
1	6.689944	-3.912907	1.999765
6	6.920948	-1.112593	4.654904
1	7.470653	-1.768539	5.337698
1	5.862647	-1.105864	4.931223
1	7.311886	-0.094381	4.735191

1	-0.174640	5.358505	-0.797868
6	0.637605	3.403676	-0.407802
1	-0.136648	2.884958	-0.962953
6	1.649346	2.651246	0.202875
6	2.901200	0.424364	0.471126
6	4.596404	0.486150	2.566847
6	3.423832	1.054123	1.751330
1	2.580602	1.062159	2.462297
79	2.906765	-1.445471	-2.009140
15	2.341353	-2.460305	-4.054346
6	2.746610	-1.462590	-5.545369
1	2.441972	-1.989901	-6.455594
1	2.228172	-0.500797	-5.491416
1	3.824095	-1.276429	-5.576554
6	0.547893	-2.823461	-4.244733
1	0.348478	-3.288094	-5.216085
1	0.225823	-3.498014	-3.446137
1	-0.021520	-1.892683	-4.165726
6	3.156219	-4.078180	-4.369455
1	2.891300	-4.779567	-3.572860
1	2.841873	-4.488858	-5.334811
1	4.241795	-3.943927	-4.368930
6	3.455106	-0.584491	-0.223288
1	4.363888	-0.996484	0.215712
8	1.013368	0.740310	-1.109399
6	-0.081116	-0.164797	-1.004690
1	-0.927693	0.298475	-0.477679
1	-0.383905	-0.409160	-2.025526
1	0.205373	-1.091023	-0.489469
8	4.732209	1.710822	3.558337
1	5.700872	1.777261	3.848530
6	4.219682	-0.697330	3.432959
1	3.291368	-0.501627	3.974792
1	4.063821	-1.558012	2.773852
1	5.002070	-0.950138	4.151718
6	5.962774	0.361934	1.879063
1	5.879039	-0.427720	1.130229
1	6.174594	1.281149	1.326682
6	1.573119	1.119889	0.137165
1	0.866785	0.820186	0.932783
6	7.165989	0.023955	2.796194
1	7.066506	-1.002732	3.160504
1	8.057340	0.032429	2.159244
6	7.364800	0.942623	3.991598
1	7.096599	0.523855	4.962784
6	7.897493	2.186509	3.980069
6	8.384767	2.880452	2.733173
1	9.447705	3.129814	2.845209
1	8.269983	2.282931	1.826452
1	7.858486	3.832718	2.588378
6	8.073216	2.970268	5.256713
1	9.138450	3.174806	5.425762
1	7.574649	3.945465	5.189190
1	7.681943	2.437093	6.127990

9ba-TS1a-5

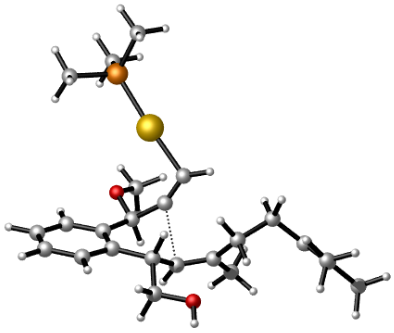

E = -1564.24784951 Hartrees
G = -1563.758383 Hartrees

6	4.094677	4.555077	1.510665
1	4.345052	5.258686	0.707478
1	3.260281	4.981744	2.089016
6	3.659291	3.196600	0.911034
1	4.487456	2.857052	0.276925
6	2.395445	3.327462	0.040260
6	2.162511	4.524346	-0.656864
1	2.876127	5.336263	-0.585773
6	1.034117	4.718868	-1.452906
1	0.898433	5.663922	-1.971012
6	0.089378	3.703290	-1.569763
1	-0.802287	3.839467	-2.174786
6	0.301729	2.497343	-0.904224
1	-0.419658	1.695481	-0.998716
6	1.442448	2.289123	-0.118346
6	3.005696	0.442463	0.476327
6	4.357051	1.409051	2.659936
6	3.452419	2.222847	2.049186
1	2.492977	2.340375	2.551279
79	3.195628	-0.978894	-1.909421
15	2.527105	-1.872625	-3.943547
6	1.685032	-0.655332	-5.026770
1	1.373208	-1.135187	-5.960296
1	0.808531	-0.254466	-4.510111
1	2.369812	0.167459	-5.250074
6	1.338070	-3.257681	-3.762022
1	1.026935	-3.620871	-4.746898
1	1.810245	-4.071707	-3.205137
1	0.461412	-2.912287	-3.207042
6	3.903761	-2.545271	-4.950671
1	4.401656	-3.345701	-4.396409
1	3.521009	-2.939834	-5.897600
1	4.628406	-1.751738	-5.152845
6	3.950220	-0.202265	-0.060321
1	4.996070	-0.370985	0.143781
8	0.778760	-0.031059	-0.169634
6	0.520474	-1.237382	0.550039
1	0.028360	-1.024510	1.508382
1	-0.143193	-1.830032	-0.081629
1	1.445276	-1.800453	0.729200
8	5.236625	4.328428	2.327620
1	5.530466	5.187585	2.667762
6	3.943988	0.653198	3.894438
1	2.902166	0.849645	4.162741
1	4.068833	-0.427708	3.759722

9ba-Int1a

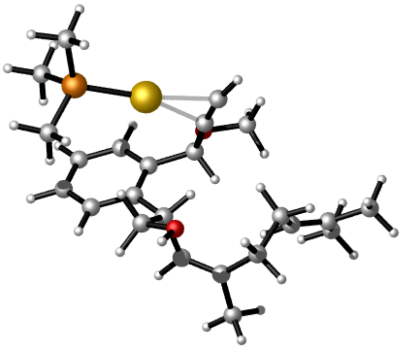

E = -1564.27378225 Hartrees
G = -1563.783491 Hartrees

6	4.402997	3.044567	-1.148187
1	4.058468	2.521729	-2.052864
1	3.996685	4.065301	-1.166348
6	3.905076	2.280128	0.094486
1	4.583557	1.426145	0.165491
6	2.474348	1.775188	-0.090601
6	1.524630	2.576464	-0.741554
1	1.812827	3.555829	-1.110744
6	0.206975	2.150790	-0.921165
1	-0.503464	2.795972	-1.430011
6	-0.191713	0.903887	-0.439249
1	-1.214860	0.563458	-0.568775
6	0.732595	0.093984	0.222334
1	0.433464	-0.871884	0.611689
6	2.051211	0.522792	0.402990
6	4.009620	-0.998484	0.144632
6	5.010415	3.154948	2.245449
6	3.996231	3.103633	1.366910
1	3.118455	3.721028	1.560271
79	3.550198	-0.727386	-2.150982
15	2.439797	-0.082857	-4.078024
6	2.568170	1.709185	-4.435291
1	2.013689	1.946337	-5.348900
1	2.147951	2.269815	-3.595961
1	3.617869	1.987420	-4.562399
6	0.645193	-0.429266	-3.965010
1	0.145753	-0.119783	-4.888937
1	0.490961	-1.499689	-3.805434
1	0.229249	0.119075	-3.115448
6	3.028687	-0.943431	-5.583210
1	2.912734	-2.022785	-5.453307
1	2.445979	-0.614479	-6.449606
1	4.085820	-0.716507	-5.745178
6	4.878308	-1.573191	-0.510937
1	5.736713	-2.122305	-0.848233
8	2.350603	-1.417615	1.801641
6	3.166040	-2.156098	2.716412
1	3.688797	-1.484552	3.410220
1	2.487944	-2.807749	3.269522
1	3.905920	-2.770251	2.185695
8	5.826455	3.067561	-1.094193
1	6.141953	3.743675	-1.713191
6	4.912073	4.032704	3.471287
1	3.936888	4.522817	3.558233
1	5.085547	3.440901	4.380112

1	4.583521	0.936285	4.739053
6	5.783047	1.280143	2.186683
1	5.826833	1.478171	1.109388
1	6.334553	2.111970	2.641130
6	1.586780	0.902577	0.531029
1	1.258230	0.927138	1.577132
6	6.521911	-0.048052	2.495687
1	5.888493	-0.900182	2.220702
1	7.389343	-0.090830	1.825499
6	6.974657	-0.209370	3.931631
1	6.385274	-0.885956	4.548542
6	8.013297	0.414993	4.508879
6	8.919351	1.388254	3.793752
1	9.971558	1.100482	3.922581
1	8.718363	1.462290	2.721841
1	8.818025	2.394590	4.223707
6	8.352851	0.191417	5.962889
1	9.373631	-0.200874	6.072189
1	8.323339	1.137916	6.520859
1	7.663188	-0.509639	6.444267

9ba-TS_{1b-3}

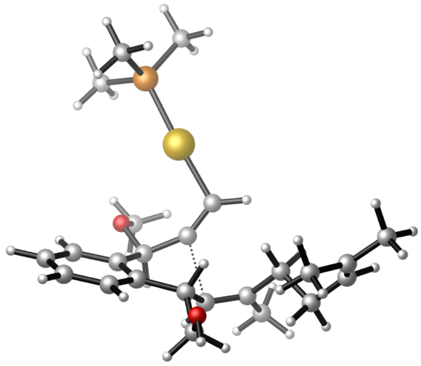

E = -1564.25496360 Hartrees
G = -1563.763850 Hartrees

6	3.492129	0.263764	-4.741662
1	3.555893	1.325792	-5.009325
1	2.679360	-0.181397	-5.332576
6	3.175887	0.139967	-3.229483
1	4.148576	0.182572	-2.724409
6	2.502402	-1.192955	-2.866388
6	2.855220	-2.340801	-3.594914
1	3.608676	-2.254799	-4.369627
6	2.276173	-3.582760	-3.339529
1	2.575909	-4.447194	-3.925460
6	1.315167	-3.705182	-2.337713
1	0.846642	-4.663323	-2.131679
6	0.960853	-2.583739	-1.590175
1	0.223422	-2.670748	-0.801683
6	1.551097	-1.335330	-1.829291
6	2.263314	0.721618	-0.594717
6	3.151810	1.076927	0.243606
1	3.920225	1.837562	0.202703
6	6.367226	3.873522	-3.138011
1	6.502444	4.803789	-2.584702
6	4.935841	3.514107	-3.434092
1	4.380354	4.414925	-3.722675
1	4.868648	2.819483	-4.274937

1	5.688857	4.809682	3.454479
6	6.317851	2.387370	2.111863
1	6.667300	2.437998	1.076877
1	7.071448	2.888218	2.731623
6	3.039024	-0.399573	1.116473
1	3.634439	0.192233	1.826606
6	6.260345	0.895485	2.539781
1	5.761565	0.330557	1.740499
1	7.284063	0.509387	2.570876
6	5.538341	0.650436	3.837649
1	4.532965	1.072876	3.877671
6	5.962737	-0.017872	4.922952
6	7.315872	-0.671335	5.063910
1	7.203550	-1.750614	5.237446
1	7.958753	-0.536356	4.191017
1	7.844397	-0.270853	5.939804
6	5.070730	-0.171859	6.133120
1	4.888942	-1.233362	6.352806
1	5.547210	0.252619	7.027793
1	4.101428	0.318729	5.996008

9ba-Int3

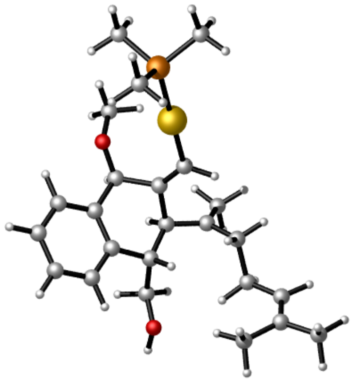

E = -1564.27137108 Hartrees
G = -1563.779811 Hartrees

6	2.988395	0.718060	-4.652279
1	2.825079	1.803885	-4.733934
1	2.105465	0.220166	-5.074843
6	3.158607	0.321799	-3.167986
1	4.232115	0.376390	-2.952159
6	2.696890	-1.109598	-2.897283
6	3.235606	-2.141520	-3.680347
1	3.964442	-1.890416	-4.444302
6	2.841168	-3.466044	-3.497634
1	3.271603	-4.248154	-4.116869
6	1.894750	-3.779844	-2.519963
1	1.578740	-4.808519	-2.370143
6	1.355972	-2.765604	-1.729597
1	0.626514	-2.998288	-0.962144
6	1.752284	-1.433389	-1.901748
6	2.245316	0.729895	-0.833643
6	3.020521	0.933686	0.243536
1	3.783945	1.707265	0.167066
6	6.437675	3.696732	-3.286445
1	6.646286	4.647690	-2.796797
6	4.983533	3.364376	-3.416647
1	4.410943	4.248825	-3.713627

6	4.247615	2.872614	-2.190627
1	4.338958	3.572413	-1.349308
1	4.808046	1.969919	-1.930159
6	2.797891	2.566427	-2.434050
6	2.346819	1.337126	-2.822940
1	1.293073	1.270650	-3.086861
6	7.461209	3.158571	-3.446328
6	7.452474	1.835815	-4.174985
1	7.995790	1.920046	-5.126888
1	7.987411	1.079756	-3.583809
1	6.456350	1.439259	-4.385913
6	8.838953	3.651172	-3.070720
1	9.470136	3.761712	-3.963642
1	8.807572	4.615580	-2.553516
1	9.348692	2.927743	-2.419163
6	1.837822	3.704270	-2.235006
1	0.805259	3.426879	-2.463697
1	1.887305	4.063686	-1.198981
1	2.120139	4.552210	-2.872334
79	3.174358	-0.177663	1.959244
15	3.305164	-1.487892	3.873143
6	4.390209	-2.956464	3.693525
1	4.395541	-3.534814	4.623205
1	5.407942	-2.631588	3.459866
1	4.023110	-3.582115	2.875253
6	1.677286	-2.157099	4.391980
1	0.998649	-1.328327	4.612036
1	1.790194	-2.784115	5.282575
1	1.254529	-2.751604	3.577285
6	3.948752	-0.596443	5.341029
1	3.985617	-1.268266	6.204578
1	3.296677	0.252190	5.565619
1	4.953847	-0.222247	5.127821
8	0.569140	-0.686488	0.273757
6	-0.259403	0.230852	0.988707
1	0.314661	1.100494	1.332904
1	-1.099681	0.566232	0.366255
1	-0.636467	-0.314782	1.855077
8	4.738849	-0.384742	-4.989941
1	4.910831	-0.349117	-5.944767
6	1.106852	-0.170712	-0.932237
1	0.329206	0.423234	-1.430250

1	4.807019	2.578472	-4.150609
6	4.435648	2.880344	-1.988075
1	4.680724	3.666290	-1.267982
1	4.973054	1.963722	-1.738482
6	2.988825	2.661665	-2.052943
6	2.415605	1.337871	-2.279038
1	1.395480	1.467869	-2.662878
6	7.476826	2.934444	-3.672469
6	7.364822	1.575518	-4.317128
1	7.928086	1.558559	-5.259936
1	7.828546	0.818582	-3.669501
1	6.341868	1.257584	-4.529301
6	8.893088	3.415155	-3.469737
1	9.420946	3.480368	-4.430726
1	8.935442	4.395875	-2.985944
1	9.456894	2.701377	-2.853544
6	2.081568	3.815794	-1.888322
1	1.050535	3.518285	-1.685939
1	2.443368	4.525407	-1.138555
1	2.083807	4.354508	-2.853334
79	3.031508	-0.143961	2.001645
15	3.089543	-1.383819	3.995279
6	4.109448	-2.910683	3.903544
1	4.077732	-3.451206	4.855315
1	5.144552	-2.644497	3.671085
1	3.726901	-3.554528	3.106314
6	1.435765	-1.982427	4.531628
1	0.780153	-1.125298	4.711000
1	1.516834	-2.576269	5.448179
1	0.999264	-2.595902	3.738064
6	3.744164	-0.492037	5.462994
1	3.729576	-1.140872	6.344987
1	3.130978	0.393700	5.653020
1	4.771022	-0.170872	5.265746
8	0.724573	-0.929593	0.204372
6	-0.240395	-0.143854	0.899518
1	0.151857	0.852805	1.136147
1	-1.160997	-0.044184	0.306909
1	-0.458353	-0.671833	1.829703
8	4.179103	0.343417	-5.336935
1	4.041504	0.470485	-6.289443
6	1.161885	-0.349682	-0.999935
1	0.290429	0.105400	-1.501508

9ba-Int1b

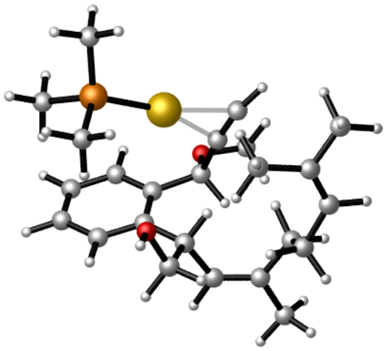

E = -1564.28235145 Hartrees
G = -1563.789428 Hartrees

9ba-TS$_{1c-2}$

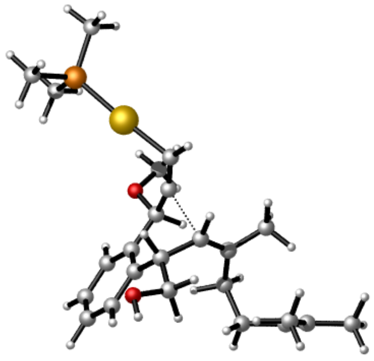

E = -1564.25179227 Hartrees
G = -1563.761877 Hartrees

6	3.904110	-0.968258	-3.304455
1	4.488645	-0.321352	-3.969328
1	3.401978	-1.712121	-3.940010
6	2.876210	-0.096163	-2.570942
1	3.472427	0.589577	-1.960518
6	1.949324	-0.880462	-1.635533
6	1.801828	-2.269500	-1.751265
1	2.373546	-2.809441	-2.496019
6	0.949972	-2.990626	-0.912585
1	0.858720	-4.066290	-1.035164
6	0.223769	-2.329701	0.077285
1	-0.440961	-2.879992	0.736706
6	0.357827	-0.948453	0.217843
1	-0.205387	-0.420668	0.978178
6	1.204039	-0.224431	-0.628916
6	2.608325	1.655583	0.242858
6	3.593372	2.158294	0.783372
1	4.355656	2.815520	1.155535
6	5.695259	3.469958	-2.749803
1	5.706097	4.554791	-2.867643
6	4.738687	2.738685	-3.653765
1	4.815179	3.122339	-4.679735
1	4.988921	1.676592	-3.702217
6	3.273021	2.887826	-3.163958
1	2.970398	3.937788	-3.260818
1	3.268616	2.670462	-2.091965
6	2.282066	2.001194	-3.898107
6	2.100504	0.701544	-3.603194
1	1.373049	0.125978	-4.175399
6	6.474579	2.938991	-1.792392
6	6.574733	1.465789	-1.471932
1	7.616854	1.125628	-1.553739
1	6.271962	1.280285	-0.431095
1	5.960310	0.821056	-2.103290
6	7.352222	3.814895	-0.928468
1	8.411609	3.546017	-1.045498
1	7.241615	4.877230	-1.168754
1	7.115875	3.678203	0.136779
6	1.512703	2.664155	-5.013393
1	0.849941	1.965341	-5.533425
1	0.906416	3.495374	-4.628535
1	2.201419	3.097998	-5.751404
79	3.954344	-0.054077	1.128201
15	4.718835	-2.156757	1.725862
6	5.675194	-2.993591	0.410907
1	5.942604	-4.004011	0.737128
1	6.583449	-2.420114	0.205906
1	5.072899	-3.028234	-0.499311
6	3.340585	-3.290798	2.132409
1	2.777780	-2.892887	2.980985
1	3.739620	-4.278229	2.385956
1	2.671808	-3.369122	1.271051
6	5.806607	-2.142443	3.199951
1	6.125308	-3.163876	3.431715
1	5.264748	-1.725615	4.053138
1	6.685490	-1.523447	3.000117
8	0.238233	1.754503	0.352208
6	0.073783	3.172300	0.321368
1	0.927086	3.687649	0.782373
1	-0.052302	3.531388	-0.709319
6	0.504040	-0.923392	-4.607026
1	1.336369	-0.619938	-5.258295
1	-0.385868	-0.357893	-4.919274
6	0.870080	-0.569466	-3.152340
1	1.562363	-1.366386	-2.846811
6	-0.295924	-0.610719	-2.157471
6	-1.484097	-1.269475	-2.512519
1	-1.553008	-1.742482	-3.483872
6	-2.564398	-1.369866	-1.636554
1	-3.465697	-1.887676	-1.952546
6	-2.479521	-0.808755	-0.364660
1	-3.312447	-0.874089	0.329408
6	-1.308672	-0.155042	0.013138
1	-1.233767	0.290555	0.997953
6	-0.214848	-0.056375	-0.857898
6	2.292909	0.442694	-0.921723
6	3.496445	0.064354	-0.856294
1	4.329888	0.047682	-1.544379
79	3.782627	-0.918680	1.024451
15	4.238318	-2.019169	3.016294
6	3.004499	-1.655263	4.324436
1	3.264237	-2.189571	5.244128
1	2.013561	-1.969602	3.985533
1	2.985286	-0.579386	4.519267
6	5.861973	-1.580994	3.747814
1	6.660100	-1.856510	3.052996
1	6.002665	-2.112173	4.694703
1	5.904440	-0.502646	3.924377
6	4.255284	-3.846749	2.867495
1	4.453681	-4.300643	3.844129
1	5.032970	-4.147898	2.160197
1	3.286283	-4.190287	2.495111
8	1.049598	0.669470	1.062111
6	1.612641	1.810934	1.712859
1	2.650211	1.982362	1.398260
1	1.013417	2.708319	1.510514
1	1.595491	1.588485	2.780862
8	0.288231	-2.328974	-4.698322
1	0.028148	-2.531570	-5.611078
6	0.960087	0.786680	-0.351847
1	0.765496	1.839321	-0.606641
6	1.330726	2.015439	-3.189309
6	1.705813	0.702600	-3.170025
1	2.743385	0.511709	-3.430897
6	2.386607	3.078895	-3.324301
1	2.361152	3.746946	-2.452630
1	3.393451	2.661649	-3.415818
1	2.176133	3.702413	-4.201226
6	-0.090304	2.515482	-3.092177
1	-0.104298	3.479890	-2.568769
1	-0.714861	1.821685	-2.526144
6	-0.771637	2.699741	-4.486254
1	-1.826383	2.927432	-4.282222
1	-0.758591	1.739908	-5.008199
6	-0.193518	3.798461	-5.334989
1	-0.415250	4.806911	-4.981422
6	0.557539	3.677823	-6.442162
6	1.055993	4.897659	-7.180478
1	0.697486	4.899685	-8.219281

1	-0.828415	3.387535	0.896056
8	4.748615	-1.592546	-2.337583
1	5.469107	-2.039366	-2.809590
6	1.319497	1.285945	-0.422647
1	1.321386	1.783858	-1.404191

9ba-Int2

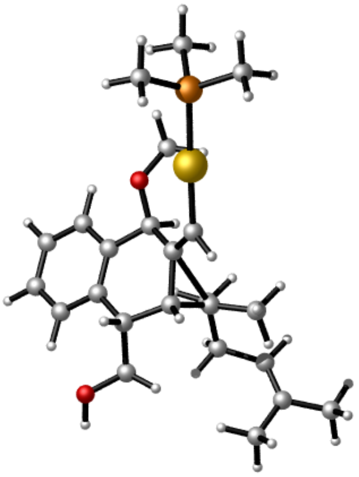

E = -1564.27412822 Hartrees
G = -1563.778697 Hartrees

6	-0.153882	-0.742356	-4.824858
1	0.724570	-0.370706	-5.375797
1	-0.945265	0.016190	-4.895987
6	0.222737	-0.968505	-3.347392
1	0.629564	-1.988858	-3.312657
6	-0.955092	-0.936960	-2.382215
6	-2.196821	-1.450266	-2.796337
1	-2.296467	-1.831144	-3.806716
6	-3.283034	-1.499711	-1.926930
1	-4.232532	-1.899095	-2.272400
6	-3.144745	-1.042631	-0.613568
1	-3.983933	-1.080395	0.075227
6	-1.918782	-0.538454	-0.191731
1	-1.800987	-0.185856	0.827627
6	-0.818315	-0.481459	-1.060843
6	1.691810	-0.032296	-1.406178
6	2.959797	-0.236014	-0.973364
1	3.707493	-0.224968	-1.770589
79	3.699605	-0.713567	0.881935
15	4.636440	-1.268086	2.965066
6	3.705378	-0.608273	4.405534
1	4.199683	-0.887929	5.341598
1	2.689166	-1.013084	4.398471
1	3.649697	0.481863	4.335997
6	6.348822	-0.648980	3.208221
1	6.998371	-1.062036	2.431243
1	6.727270	-0.942577	4.192868
1	6.355158	0.441793	3.127014
6	4.752304	-3.073629	3.286236
1	5.194368	-3.257931	4.270921
1	5.372193	-3.540905	2.515768
1	3.752458	-3.515262	3.247418
8	0.683641	-0.276512	0.805790
6	0.817609	0.779491	1.755393

1	0.732236	5.829204	-6.705249
1	2.153816	4.901596	-7.231438
6	0.994086	2.365718	-7.047489
1	0.611560	1.490605	-6.517922
1	0.673861	2.296781	-8.095888
1	2.090919	2.299515	-7.052312

9ba-Int1c

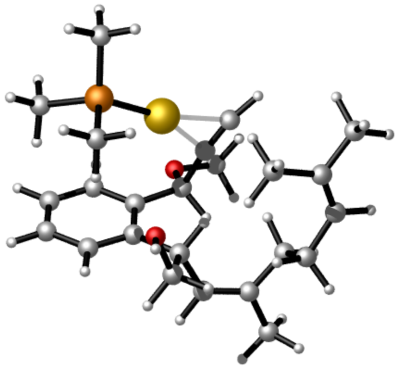

E = -1564.27486872 Hartrees
G = -1563.784277 Hartrees

6	2.259944	-0.625778	-3.246790
1	3.013301	-0.434968	-4.023355
1	1.286679	-0.705085	-3.750617
6	2.265448	0.553715	-2.265638
1	3.256323	0.493921	-1.793049
6	1.237311	0.441587	-1.143719
6	0.138458	-0.421818	-1.232304
1	0.024476	-1.056380	-2.102493
6	-0.824010	-0.486795	-0.223009
1	-1.673125	-1.155652	-0.331612
6	-0.692546	0.305964	0.916767
1	-1.437190	0.266805	1.706308
6	0.411854	1.150125	1.042644
1	0.528432	1.769985	1.923548
6	1.369350	1.222225	0.026391
6	3.803306	1.384081	0.546417
6	4.898062	0.911466	0.850753
1	5.937846	0.736728	1.051451
79	3.551565	-0.902105	1.098742
15	2.604679	-2.982407	1.459044
6	0.959156	-2.854786	2.252466
1	0.532517	-3.853710	2.389215
1	0.301015	-2.253677	1.620326
1	1.061213	-2.362792	3.223588
6	3.603849	-4.058720	2.554553
1	4.589058	-4.219811	2.108725
1	3.100710	-5.022151	2.686060
1	3.728657	-3.575643	3.527408
6	2.348361	-3.933785	-0.082375
1	1.812255	-4.862639	0.137922
1	3.321082	-4.167986	-0.523854
1	1.787504	-3.321778	-0.792371
8	2.296539	3.094889	1.224241
6	3.207844	4.193091	1.260854
1	4.225051	3.865433	1.513817
1	3.225851	4.720822	0.297467
1	2.843762	4.865806	2.039021

1	1.638133	1.459724	1.490976
1	-0.115988	1.352433	1.844151
1	1.044917	0.308941	2.713950
8	-0.588575	-1.986030	-5.363076
1	-0.949989	-1.821171	-6.248655
6	0.455027	0.167140	-0.526221
1	0.261326	1.249459	-0.478909
6	1.465542	1.367015	-3.042622
6	1.425338	-0.088369	-2.969596
1	2.311278	-0.512286	-3.440808
6	2.751593	2.043078	-3.350189
1	2.942735	2.882894	-2.675159
1	3.605968	1.366656	-3.368431
1	2.632067	2.490412	-4.351315
6	0.266654	2.234047	-2.877126
1	0.489949	3.072128	-2.207132
1	-0.578751	1.674594	-2.478117
6	-0.210709	2.842474	-4.259288
1	-1.205306	3.244982	-4.030054
1	-0.363386	2.034589	-4.975471
6	0.644223	3.937390	-4.816884
1	0.743210	4.809171	-4.169077
6	1.265042	3.968033	-6.011012
6	2.049804	5.184446	-6.438841
1	1.641125	5.601098	-7.369451
1	2.041167	5.971095	-5.678163
1	3.094298	4.917968	-6.649948
6	1.249947	2.849895	-7.024593
1	0.737112	1.947986	-6.682064
1	0.764061	3.182400	-7.951479
1	2.277324	2.571849	-7.294279

9bb-Int5

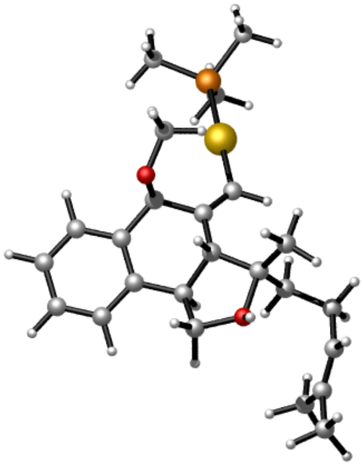

E = -1564.29084246 Hartrees
G = -1563.794540 Hartrees

6	2.916182	4.629618	-2.495396
1	2.964563	5.716576	-2.447951
1	2.283456	4.293117	-3.318510
6	2.621044	3.924963	-1.189980
1	3.219205	4.419004	-0.412031
6	1.203163	3.831777	-0.702226
6	0.112416	4.508860	-1.247609
1	0.246441	5.180344	-2.091652
6	-1.162127	4.326968	-0.700994

8	2.565365	-1.816949	-2.522669
1	2.594075	-2.555283	-3.151471
6	2.567080	2.156526	0.207197
1	2.770287	2.674862	-0.743713
6	1.310732	2.767959	-3.308454
6	2.268260	1.855288	-3.060896
1	3.237640	2.032439	-3.528846
6	1.636273	3.963954	-4.174913
1	1.496565	4.896658	-3.611057
1	2.664066	3.940697	-4.551427
1	0.951673	4.007192	-5.030684
6	-0.126062	2.718331	-2.819604
1	-0.530047	3.738757	-2.801599
1	-0.182443	2.343122	-1.796673
6	-1.047865	1.840290	-3.709754
1	-2.017616	1.760198	-3.198305
1	-0.635622	0.827996	-3.748598
6	-1.267110	2.409332	-5.086128
1	-1.876661	3.315224	-5.100890
6	-0.783665	1.973137	-6.260149
6	-1.090444	2.702689	-7.547300
1	-1.594389	2.040289	-8.265243
1	-1.728126	3.577785	-7.384568
1	-0.164803	3.040351	-8.034678
6	0.101465	0.762991	-6.432432
1	0.281840	0.223713	-5.500861
1	-0.336771	0.061083	-7.155313
1	1.078059	1.060307	-6.839087

9bb-TS$_{1a-5}$

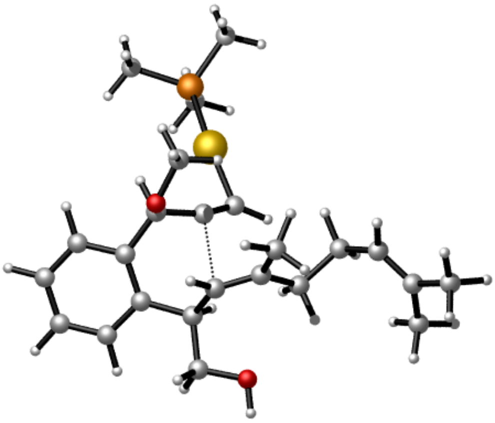

E = -1564.24652057 Hartrees
G = -1563.757227 Hartrees

6	4.287091	3.869440	-2.502548
1	5.182024	4.396421	-2.145227
1	3.527766	4.623576	-2.761888
6	3.738868	2.949457	-1.389526
1	4.559274	2.266243	-1.132430
6	3.339536	3.728608	-0.125714
6	3.844759	5.020117	0.100047
1	4.523939	5.467371	-0.615000
6	3.501438	5.772347	1.223446
1	3.917943	6.767725	1.348763

1	-2.011301	4.855563	-1.124355
6	-1.338693	3.467266	0.384500
1	-2.328700	3.324375	0.808621
6	-0.245533	2.786077	0.930384
1	-0.375623	2.111340	1.769512
6	1.027757	2.964977	0.391996
6	3.032884	1.580359	-0.213664
6	4.618466	2.749688	-1.949333
6	3.194259	2.501630	-1.430056
1	2.616983	2.072954	-2.260648
79	3.436741	-1.095996	1.286417
15	3.485279	-2.703178	2.999581
6	4.347630	-2.136226	4.522459
1	4.337609	-2.919440	5.287836
1	3.846675	-1.243544	4.908583
1	5.382567	-1.877413	4.280311
6	1.830373	-3.230646	3.605784
1	1.932268	-3.963879	4.412772
1	1.263935	-3.673774	2.781542
1	1.282983	-2.358105	3.974309
6	4.323713	-4.283987	2.575230
1	3.814123	-4.748485	1.725945
1	4.306807	-4.970160	3.428647
1	5.360681	-4.081987	2.291639
6	3.460315	0.305355	-0.219455
1	3.936080	-0.002156	-1.155793
8	1.912106	1.330039	1.948372
6	2.241222	1.745478	3.267636
1	1.727848	2.678788	3.537817
1	3.325853	1.881261	3.383412
1	1.909936	0.947817	3.936831
8	4.312637	4.165529	-2.751024
1	4.516094	4.102202	-3.706223
6	5.114390	1.809661	-3.022597
1	4.374533	1.680606	-3.818036
1	5.280542	0.829818	-2.566386
1	6.067538	2.144163	-3.444155
6	5.677374	3.178476	-0.936780
1	5.206996	3.756721	-0.135073
1	6.375259	3.852392	-1.444740
6	2.265334	2.251401	0.937988
1	2.929448	3.018156	1.376050
6	6.490599	2.013792	-0.316285
1	7.076846	1.520520	-1.096234
1	5.797746	1.268785	0.087084
6	7.369509	2.524806	0.794413
1	6.822854	2.860608	1.677811
6	8.707810	2.627356	0.812207
6	9.427310	3.166502	2.026359
1	10.131836	2.423144	2.424855
1	10.025131	4.050941	1.765537
1	8.735175	3.444664	2.827721
6	9.620951	2.235113	-0.324024
1	9.091492	1.862634	-1.204306
1	10.229919	3.094873	-0.635404
1	10.326171	1.457815	0.000488
6	2.628234	5.246998	2.173060
1	2.347296	5.820611	3.051134
6	2.124400	3.963900	1.981009
1	1.445257	3.540155	2.716925
6	2.465054	3.200396	0.855227
6	2.692861	0.762674	0.264080
6	2.539966	0.994863	-2.605458
6	2.557941	2.162061	-1.917190
1	1.605282	2.668857	-1.792678
79	3.432420	-1.002738	2.277526
15	3.504170	-1.989213	4.377260
6	5.209979	-2.178131	5.020351
1	5.186679	-2.655567	6.005344
1	5.679719	-1.194108	5.101736
1	5.793802	-2.793386	4.330538
6	2.603096	-1.036729	5.659266
1	2.683113	-1.542733	6.626971
1	1.549826	-0.952531	5.378001
1	3.031511	-0.033646	5.735375
6	2.779611	-3.672718	4.420614
1	1.727006	-3.625874	4.128053
1	2.860342	-4.086382	5.431132
1	3.313806	-4.318714	3.718464
6	3.498504	-0.201794	0.283642
1	4.174189	-0.694815	-0.397480
8	0.537025	1.909750	0.209633
6	-0.228685	0.709708	0.301823
1	-0.341090	0.389791	1.348133
1	0.229249	-0.104454	-0.275009
1	-1.210569	0.939052	-0.115538
8	4.618827	3.054934	-3.619218
1	4.973396	3.635402	-4.310211
6	1.219012	0.467931	-3.107850
1	0.392243	1.132209	-2.842240
1	1.005394	-0.525585	-2.695735
1	1.243970	0.350910	-4.198118
6	3.782192	0.216722	-2.965934
1	4.593874	0.484101	-2.280627
1	4.117590	0.584153	-3.942936
6	1.812729	1.821368	0.812032
1	1.682583	1.498335	1.860237
6	3.645576	-1.329637	-3.035547
1	4.656250	-1.745738	-2.937799
1	3.084485	-1.694752	-2.166942
6	2.996770	-1.859221	-4.297057
1	1.961497	-2.184467	-4.205070
6	3.569967	-1.942334	-5.507891
6	2.809431	-2.468387	-6.701393
1	2.764726	-1.714566	-7.500102
1	3.311537	-3.346072	-7.132295
1	1.783831	-2.752915	-6.444005
6	4.989748	-1.521734	-5.801937
1	5.544642	-1.215714	-4.911375
1	5.542498	-2.340291	-6.282971
1	5.002779	-0.681151	-6.509732

9bb-Int1a

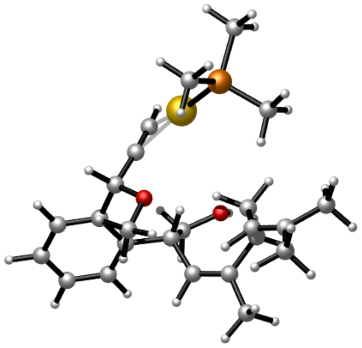

E = -1564.27317825 Hartrees
G = -1563.782591 Hartrees

6	4.975579	3.825989	-1.467497
1	5.298959	4.329990	-0.543527
1	4.853415	4.594421	-2.243945
6	3.627136	3.115855	-1.202974
1	3.875715	2.221981	-0.630700
6	2.754092	4.041952	-0.357503
6	2.443136	5.315119	-0.859805
1	2.799481	5.595239	-1.846901
6	1.687579	6.232283	-0.128910
1	1.469797	7.209380	-0.550629
6	1.214114	5.890332	1.137934
1	0.623137	6.593216	1.717301
6	1.495928	4.624943	1.648470
1	1.112252	4.343037	2.625720
6	2.256803	3.702101	0.917903
6	3.869166	2.037546	1.815765
6	3.033583	1.547442	-3.149520
6	2.896070	2.696827	-2.468823
1	2.170837	3.421566	-2.839161
79	4.425205	-0.226126	2.009350
15	4.201587	-2.530152	2.129905
6	5.533293	-3.328844	3.102260
1	5.355685	-4.407697	3.158129
1	5.552087	-2.908204	4.111284
1	6.497783	-3.142570	2.622060
6	2.631820	-3.012246	2.943487
1	2.557726	-4.103422	2.990552
1	1.789945	-2.613806	2.370710
1	2.600820	-2.598830	3.955054
6	4.185670	-3.377035	0.508172
1	3.353728	-2.999418	-0.092363
1	4.072505	-4.455886	0.656514
1	5.121930	-3.179520	-0.020173
6	5.067738	1.962622	2.077778
1	6.109126	2.141668	2.264040
8	1.974840	1.249526	0.710086
6	0.586809	1.339252	0.385024
1	0.389256	2.180360	-0.288597
1	-0.027237	1.443996	1.289992
1	0.338818	0.403248	-0.117139
8	5.934094	2.849324	-1.864722
1	6.725386	3.314996	-2.176754
6	2.192414	1.277203	-4.375371
1	1.466281	2.074112	-4.568112

9bb-TS$_{1b-3}$

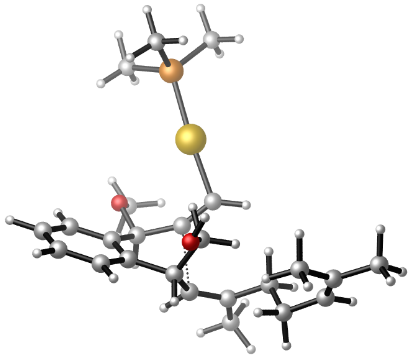

E = -1564.24880489 Hartrees
G = -1563.758370 Hartrees

6	2.506428	-1.304195	-2.906603
6	2.738023	-2.410993	-3.736174
1	3.476619	-2.318998	-4.525452
6	2.059663	-3.615279	-3.559110
1	2.262807	-4.453958	-4.219030
6	1.117010	-3.729537	-2.537785
1	0.571928	-4.657421	-2.389785
6	0.867546	-2.639723	-1.704950
1	0.131647	-2.721737	-0.914243
6	1.557379	-1.430017	-1.867429
6	2.309421	0.616779	-0.562184
6	3.159288	1.068624	0.258406
1	3.884810	1.869927	0.235492
6	6.418654	3.633924	-3.378440
1	6.462712	4.667588	-3.030843
6	5.027362	3.129674	-3.646231
1	4.507968	3.814396	-4.330313
1	5.038773	2.153971	-4.139679
6	4.195060	3.039681	-2.333871
1	4.127840	4.038798	-1.888914
1	4.739100	2.417044	-1.616527
6	2.812860	2.497394	-2.581576
6	2.507424	1.198491	-2.860144
1	1.467612	1.039725	-3.141438
6	7.577167	2.963265	-3.485361
6	7.720805	1.528277	-3.934024
1	8.349187	1.472103	-4.833690
1	8.239831	0.942416	-3.162026
1	6.778035	1.022655	-4.151583
6	8.889118	3.635938	-3.152895
1	9.562130	3.631396	-4.021690
1	8.752965	4.673820	-2.832385
1	9.413761	3.095415	-2.352469
6	1.712532	3.524182	-2.542552
1	0.730462	3.106348	-2.780609
1	1.666059	3.992254	-1.550131
1	1.929403	4.331683	-3.255032
79	3.227418	-0.226431	1.957192
15	3.399526	-1.538303	3.864763
6	4.493146	-2.997839	3.669504
1	4.512497	-3.577135	4.598435
1	5.505989	-2.665284	3.425733
1	4.121169	-3.625100	2.854716
6	1.781110	-2.216770	4.399376

1	1.647748	0.330130	-4.265387
1	2.826464	1.166682	-5.266233
6	4.020471	0.453098	-2.783324
1	4.944913	0.906368	-2.419795
1	4.271642	-0.112168	-3.690365
6	2.442235	2.317178	1.528269
1	1.911164	2.293544	2.492661
6	3.521060	-0.553890	-1.714376
1	4.345209	-1.246114	-1.509107
1	3.329227	-0.006335	-0.786242
6	2.267941	-1.300623	-2.088127
1	1.341948	-0.744979	-1.934987
6	2.166375	-2.534984	-2.610837
6	0.819939	-3.136019	-2.939207
1	0.751113	-3.383402	-4.007802
1	0.666039	-4.077777	-2.393561
1	-0.004786	-2.459341	-2.692036
6	3.341987	-3.424299	-2.937638
1	4.306870	-2.972750	-2.695064
1	3.268364	-4.381087	-2.402197
1	3.346298	-3.667813	-4.008869

9bb-Int3

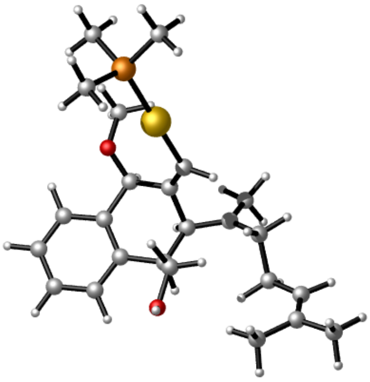

E = -1564.26625952 Hartrees
G = -1563.772765 Hartrees

6	2.584870	-1.286943	-3.050460
6	2.798120	-2.267201	-4.029439
1	3.569991	-2.101437	-4.773263
6	2.053668	-3.445752	-4.044397
1	2.237930	-4.192428	-4.811765
6	1.071754	-3.657182	-3.074156
1	0.484356	-4.571155	-3.076248
6	0.841128	-2.685144	-2.101968
1	0.078579	-2.839625	-1.345910
6	1.589228	-1.501248	-2.072775
6	2.394724	0.535456	-0.855555
6	3.187721	0.738578	0.208452
1	3.991604	1.464342	0.084469
6	6.349964	3.702137	-3.499205
1	6.383186	4.756053	-3.223769
6	4.969710	3.118375	-3.552481
1	4.297091	3.787374	-4.099725
1	4.948294	2.148404	-4.050817
6	4.405969	2.967216	-2.069292
1	4.451245	3.945069	-1.583695
1	5.066239	2.273989	-1.546860
6	3.028825	2.462206	-2.135398

1	1.100188	-1.391887	4.626768
1	1.907470	-2.843255	5.288465
1	1.353577	-2.813546	3.588921
6	4.053421	-0.639043	5.322548
1	4.107589	-1.310648	6.185310
1	3.395673	0.202935	5.555304
1	5.052045	-0.255253	5.096697
8	0.627240	-0.811881	0.269351
6	-0.223127	0.084075	0.987323
1	0.331616	0.961520	1.343297
1	-1.065188	0.408465	0.361782
1	-0.595001	-0.476962	1.845992
6	4.725054	-0.209446	-2.499203
1	5.242426	0.746274	-2.399150
1	4.616137	-0.640276	-1.496304
8	5.468651	-1.074721	-3.356563
1	6.309158	-1.277812	-2.917062
6	3.335745	-0.041641	-3.154942
1	3.542626	-0.001190	-4.234773
6	1.167664	-0.276945	-0.928943
1	0.398988	0.342117	-1.413806

9bb-Int1b

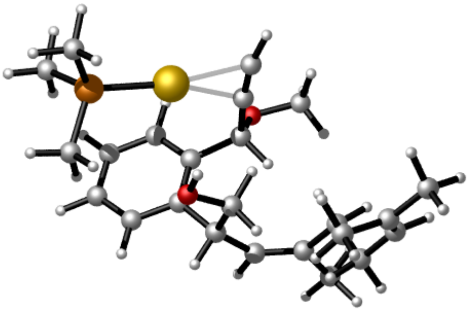

E = -1564.27242276 Hartrees
G = -1563.781873 Hartrees

6	2.331794	-1.341013	-1.521855
6	2.203095	-2.735795	-1.606987
1	2.845801	-3.277329	-2.295880
6	1.278046	-3.442062	-0.838182
1	1.206952	-4.521761	-0.933665
6	0.445485	-2.754345	0.046514
1	-0.285778	-3.287686	0.646908
6	0.553973	-1.368837	0.149171
1	-0.089941	-0.820288	0.828116
6	1.481470	-0.658849	-0.627363
6	2.458316	1.192520	0.721172
6	3.133853	1.635281	1.650010
1	3.648064	2.249188	2.364382
6	5.820585	3.865918	-3.391307
1	5.398711	4.872578	-3.415461
6	4.947898	2.812948	-4.018631
1	4.652175	3.134602	-5.026353
1	5.479109	1.864298	-4.142518
6	3.667848	2.563975	-3.176887
1	3.116373	3.510994	-3.095926
1	3.973367	2.308713	-2.161063
6	2.753601	1.519579	-3.786691

6	2.658933	1.064471	-2.323219	6	2.670673	0.218919	-3.453070
1	1.671809	1.080110	-2.801159	1	1.952963	-0.373326	-4.021479
6	7.512460	3.067915	-3.730663	6	7.004819	3.704822	-2.779237
6	7.659292	1.610874	-4.091178	6	7.731939	2.389187	-2.632723
1	8.179022	1.511811	-5.053696	1	8.748201	2.463351	-3.043198
1	8.292734	1.106794	-3.348063	1	7.846265	2.130584	-1.570404
1	6.722372	1.054789	-4.156181	1	7.231596	1.554542	-3.129283
6	8.819606	3.819360	-3.646310	6	7.728426	4.880564	-2.164612
1	9.356021	3.771497	-4.603577	1	8.717827	5.016011	-2.623684
1	8.678406	4.872215	-3.383503	1	7.169329	5.815099	-2.277637
1	9.479700	3.361758	-2.896698	1	7.905616	4.715102	-1.092311
6	1.938930	3.461437	-2.045140	6	1.878956	2.054359	-4.901351
1	0.939867	3.023794	-2.026152	1	1.269174	1.272256	-5.363528
1	2.087154	4.127545	-1.187529	1	1.205833	2.834450	-4.519590
1	2.019686	4.104457	-2.938634	1	2.483284	2.523268	-5.688829
79	3.177984	-0.255774	2.014515	79	3.467020	-0.607605	1.866868
15	3.207855	-1.380394	4.075812	15	4.029168	-2.759770	2.502846
6	4.132962	-2.968342	4.060397	6	4.687879	-3.758348	1.118170
1	4.094837	-3.444072	5.046060	1	4.914848	-4.770619	1.468602
1	5.175396	-2.779096	3.788568	1	5.589388	-3.286245	0.722278
1	3.692996	-3.638009	3.315837	1	3.941161	-3.793647	0.321521
6	1.534577	-1.840990	4.683644	6	2.584823	-3.693000	3.134523
1	0.930983	-0.937446	4.809458	1	2.182461	-3.191419	4.018823
1	1.602679	-2.368037	5.641053	1	2.885887	-4.712663	3.394833
1	1.047459	-2.487225	3.947538	1	1.813403	-3.723217	2.360400
6	3.951669	-0.439127	5.468092	6	5.292937	-2.839565	3.827778
1	3.923855	-1.031663	6.388437	1	5.499448	-3.884771	4.080645
1	3.395774	0.490998	5.617689	1	4.928692	-2.314883	4.715144
1	4.989522	-0.191552	5.227153	1	6.212971	-2.359002	3.483947
8	0.879152	-1.110175	0.189701	8	0.261122	1.360204	-0.187326
6	-0.062369	-0.383125	0.977292	6	0.174418	2.782277	-0.281299
1	0.313372	0.615878	1.231319	1	0.753211	3.275435	0.511496
1	-1.020395	-0.289771	0.446583	1	0.529026	3.133955	-1.260405
1	-0.206721	-0.953510	1.896626	1	-0.880961	3.033368	-0.163577
6	4.855033	-0.367860	-2.486899	6	4.543755	-0.058480	-1.636061
1	5.405989	0.537769	-2.209360	1	5.276405	0.351832	-2.342196
1	4.747877	-0.980682	-1.583590	1	4.226903	0.750072	-0.972224
8	5.551139	-1.061754	-3.517360	8	5.123652	-1.111030	-0.862081
1	6.369623	-1.420003	-3.139783	1	5.815655	-0.717958	-0.307067
6	3.461924	-0.033208	-3.054658	6	3.370033	-0.656551	-2.421745
1	3.612677	0.285161	-4.093337	1	3.826303	-1.471372	-3.001695
6	1.236931	-0.454172	-1.004856	6	1.557011	0.851962	-0.425571
1	0.357632	0.108199	-1.366507	1	1.981872	1.323653	-1.319987

9bb-TS$_{1c-2}$

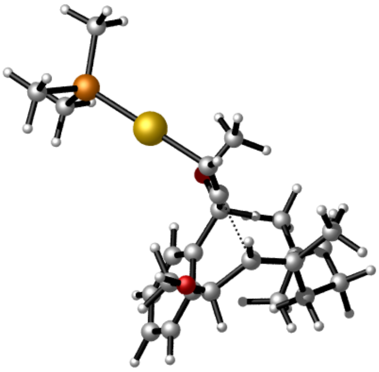

E = -1564.26056720 Hartrees
G = -1563.769491 Hartrees

9bb-Int2

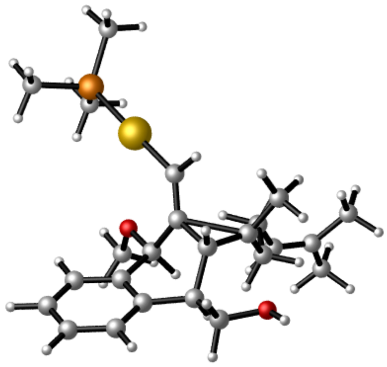

E = -1564.28566898 Hartrees
G = -1563.792408 Hartrees

6	2.801005	-0.565435	-3.367674
6	3.033728	-1.593910	-4.295290
1	3.925326	-1.541601	-4.915405
6	2.163567	-2.672125	-4.438465
1	2.375671	-3.448369	-5.167887
6	1.024030	-2.741886	-3.637371
1	0.333246	-3.574958	-3.729246
6	0.763536	-1.723210	-2.722158
1	-0.129669	-1.763666	-2.111315
6	1.634587	-0.634110	-2.575820
6	2.304401	1.157542	-0.907035
6	2.930899	1.463323	0.148900
1	3.723796	2.156901	0.389521
79	2.467949	-0.040123	1.597093
15	2.131245	-1.637918	3.249186
6	3.687020	-2.399368	3.850584
1	3.468814	-3.153206	4.614012
1	4.329662	-1.624230	4.276967
1	4.208984	-2.868968	3.012340
6	1.091051	-3.039893	2.684156
1	0.110561	-2.664538	2.378054
1	0.967619	-3.767244	3.493146
1	1.566595	-3.523045	1.826163
6	1.297328	-0.994321	4.749670
1	1.163524	-1.799581	5.479270
1	0.321372	-0.584166	4.475989
1	1.904036	-0.198731	5.190825
8	0.262107	-0.018813	-0.693559
6	-0.555426	0.973709	-0.070861
1	0.029326	1.601385	0.613180
1	-1.042353	1.606781	-0.821583
1	-1.311654	0.429726	0.497422
6	1.191280	0.491097	-1.639106
1	0.692366	1.257084	-2.248046
6	2.594630	2.718414	-3.394610
6	3.469662	1.879246	-2.766593
1	4.141229	2.328991	-2.044964
6	1.906154	2.351184	-4.688379
1	1.866765	1.265628	-4.810176
1	2.583754	2.716983	-5.478922
6	2.387603	4.112870	-2.872477
1	1.343459	4.266398	-2.570334
1	3.027932	4.325246	-2.012002
1	2.595179	4.853091	-3.656520
6	5.010106	-0.071198	-2.344720
1	4.600003	-0.298789	-1.350766
1	5.365443	-1.013272	-2.786246
6	3.892045	0.499291	-3.242639
1	4.340545	0.624065	-4.238436
8	6.057256	0.890468	-2.266508
1	6.733162	0.545180	-1.662839
6	0.503282	2.943707	-4.958666
1	0.529764	4.031426	-4.834385
1	0.282840	2.770303	-6.018204
6	-0.593046	2.359723	-4.096869
1	-0.901323	2.958506	-3.238643
6	-1.204779	1.178305	-4.289960
6	-0.884910	0.236665	-5.425693
1	-0.151979	0.638140	-6.129715
1	-0.488624	-0.709353	-5.035321
6	2.503831	4.444239	-2.818380
1	2.114525	5.464606	-2.695151
1	1.798649	3.882873	-3.449062
6	2.645131	3.774358	-1.455032
1	3.342402	4.399495	-0.884430
6	1.383457	3.620933	-0.633035
6	0.134635	4.140904	-0.978416
1	0.009025	4.709821	-1.894541
6	-0.969126	3.930183	-0.144853
1	-1.935951	4.342585	-0.418888
6	-0.831461	3.187750	1.028902
1	-1.691159	3.018243	1.671009
6	0.413284	2.654311	1.377775
1	0.528401	2.057454	2.276536
6	1.517058	2.879750	0.558182
6	3.248782	1.469115	-0.399888
6	4.746937	2.255214	-1.700355
6	3.291842	2.378507	-1.664839
1	2.816827	1.878425	-2.510554
79	3.228491	-1.259377	1.044873
15	3.025859	-2.883736	2.730694
6	3.797551	-2.397381	4.326239
1	3.661652	-3.185853	5.073582
1	3.335045	-1.472863	4.684016
1	4.866275	-2.218833	4.176755
6	1.287561	-3.283339	3.174342
1	1.256796	-4.034009	3.971162
1	0.766512	-3.667290	2.292650
1	0.781606	-2.374087	3.511442
6	3.778768	-4.512693	2.335315
1	3.296525	-4.927793	1.445629
1	3.654288	-5.206104	3.173669
1	4.844535	-4.382182	2.126883
6	3.407177	0.123419	-0.461684
1	3.633465	-0.260142	-1.457932
8	2.948965	1.511267	2.015888
6	3.251061	2.261913	3.190377
1	2.501101	3.041252	3.376525
1	4.243079	2.728947	3.114002
1	3.249462	1.551860	4.019884
8	3.814099	4.456934	-3.382781
1	3.754450	4.790124	-4.291530
6	5.369708	1.384869	-2.732573
1	5.502728	2.064529	-3.590898
1	4.721977	0.575657	-3.073444
1	6.351049	1.000570	-2.447643
6	5.675819	3.021497	-0.821533
1	5.185282	3.865874	-0.335524
1	6.451669	3.434130	-1.479366
6	2.895331	2.288629	0.844728
1	3.619804	3.112418	0.941071
6	6.395199	2.144118	0.259873
1	6.921323	1.324358	-0.236612
1	5.625769	1.684848	0.889631
6	7.318256	2.980431	1.101621
1	6.822654	3.559709	1.881714
6	8.649042	3.105101	0.971777
6	9.438740	3.996061	1.900165
1	10.215562	3.422134	2.423858
1	9.960465	4.780857	1.335171

1	-1.796343	-0.009540	-5.986905
6	-2.278511	0.685443	-3.350344
1	-2.543371	1.433391	-2.596499
1	-3.190383	0.422015	-3.902788
1	-1.952237	-0.225730	-2.830670

1	8.804115	4.477696	2.650781
6	9.480299	2.402089	-0.073360
1	8.895676	1.784172	-0.759290
1	10.033305	3.137384	-0.673191
1	10.234290	1.761164	0.403396

9bb-Int1c

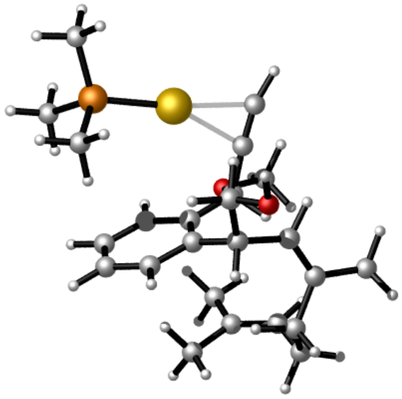

E = -1564.27668737 Hartrees
G = -1563.786229 Hartrees

6	2.306251	-0.351847	-2.122099
6	2.537469	-1.681798	-2.518373
1	3.467786	-1.912855	-3.031502
6	1.623312	-2.705350	-2.278748
1	1.842267	-3.717134	-2.607446
6	0.426173	-2.413382	-1.624024
1	-0.305766	-3.192319	-1.431325
6	0.163980	-1.101695	-1.234323
1	-0.769136	-0.863267	-0.739810
6	1.080045	-0.067313	-1.482111
6	1.438669	1.741650	0.205938
6	1.965273	2.197865	1.220728
1	2.386345	2.834980	1.974870
79	2.116467	-0.039347	1.597272
15	2.513342	-2.238358	2.197027
6	3.682547	-3.038180	1.036360
1	3.824111	-4.089102	1.308546
1	4.643536	-2.518194	1.070439
1	3.276569	-2.969399	0.023273
6	1.001246	-3.270086	2.153279
1	0.269471	-2.882173	2.866729
1	1.251202	-4.304972	2.408933
1	0.575351	-3.232168	1.147232
6	3.222093	-2.449492	3.872028
1	3.375291	-3.513809	4.077961
1	2.536770	-2.029307	4.613126
1	4.179133	-1.924136	3.933403
8	-0.696417	1.391770	-0.744231
6	-1.222491	2.717186	-0.699433
1	-0.794749	3.292592	0.132522
1	-1.032234	3.245230	-1.643333
1	-2.298391	2.613767	-0.550566
6	0.689111	1.339865	-1.017932
1	0.953655	2.057513	-1.805687
6	2.710764	2.660318	-3.718296
6	3.148999	2.090260	-2.584941

9bb-TS2-5

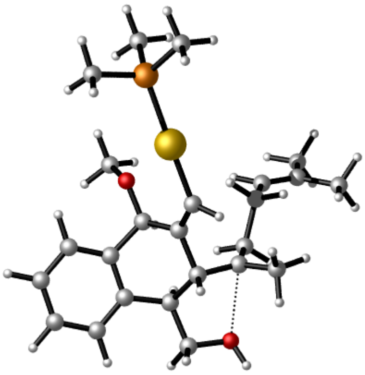

E = -1564.28466804 Hartrees
G = -1563.790183 Hartrees

6	2.700779	4.498034	-2.690204
1	2.318521	5.522275	-2.588440
1	2.078385	3.965491	-3.421590
6	2.679603	3.787252	-1.343108
1	3.329117	4.375400	-0.683181
6	1.350450	3.623893	-0.650267
6	0.136441	4.138315	-1.108923
1	0.095701	4.716764	-2.027195
6	-1.037840	3.910799	-0.383970
1	-1.978845	4.318846	-0.741579
6	-1.001656	3.156838	0.790549
1	-1.915613	2.974857	1.348923
6	0.209017	2.627665	1.248665
1	0.243502	2.022020	2.148071
6	1.383677	2.868182	0.538319
6	3.215425	1.475203	-0.272614
6	4.781822	2.397217	-1.732896
6	3.326402	2.380198	-1.537863
1	2.829697	1.887978	-2.378596
79	3.235136	-1.250896	1.171054
15	3.028908	-2.874562	2.855569
6	3.802777	-2.388375	4.450899
1	3.665599	-3.175015	5.200010
1	3.343265	-1.461926	4.807676
1	4.871838	-2.212485	4.300287
6	1.291814	-3.273864	3.306370
1	1.263242	-4.023079	4.104718
1	0.767457	-3.659212	2.427210
1	0.786901	-2.363965	3.643454
6	3.778879	-4.507305	2.468201
1	3.301332	-4.922814	1.576187
1	3.647152	-5.198831	3.307086
1	4.846371	-4.380360	2.266320
6	3.438991	0.147219	-0.323746
1	3.754074	-0.227459	-1.301300
8	2.662294	1.480325	2.100729

1	3.355203	2.736538	-1.731813
6	2.431195	1.877733	-4.983038
1	2.477660	0.803261	-4.785711
1	3.223702	2.103715	-5.713602
6	2.495657	4.152797	-3.794694
1	1.432936	4.395724	-3.925903
1	2.848110	4.660364	-2.891226
1	3.022999	4.580571	-4.657944
6	4.521737	0.458419	-1.266029
1	4.118907	0.832766	-0.313109
1	4.748635	-0.610081	-1.133570
6	3.467493	0.621852	-2.378331
1	3.963846	0.259589	-3.286713
8	5.677345	1.188883	-1.657852
1	6.331778	1.114750	-0.945937
6	1.068754	2.190586	-5.651078
1	1.072245	3.227947	-6.005519
1	0.994274	1.566692	-6.550157
6	-0.136714	1.982221	-4.761603
1	-0.548450	2.882208	-4.302692
6	-0.752319	0.817066	-4.502531
6	-0.288423	-0.517494	-5.032982
1	0.563544	-0.439491	-5.713037
1	0.006528	-1.170493	-4.201398
1	-1.103431	-1.029627	-5.562763
6	-1.997993	0.754966	-3.652627
1	-2.303365	1.744200	-3.297689
1	-2.830963	0.324799	-4.226674
1	-1.846497	0.114356	-2.777001

6	2.874053	2.206026	3.308578
1	2.131678	3.004789	3.436424
1	3.881464	2.645033	3.333646
1	2.774557	1.486643	4.124201
8	4.076072	4.512098	-3.085341
1	4.132045	4.668323	-4.041086
6	5.357422	1.575996	-2.825363
1	4.952894	1.960560	-3.770713
1	5.006425	0.539245	-2.750282
1	6.447779	1.592003	-2.854126
6	5.711469	3.101892	-0.824052
1	5.211771	3.844562	-0.201429
1	6.445184	3.621089	-1.451785
6	2.732665	2.275247	0.941223
1	3.446980	3.096351	1.122111
6	6.509799	2.108973	0.104998
1	7.018312	1.365722	-0.514448
1	5.775367	1.569766	0.711674
6	7.461995	2.866806	0.985128
1	6.999357	3.346804	1.847925
6	8.782350	3.033287	0.801444
6	9.606682	3.828235	1.784994
1	10.407788	3.208511	2.210653
1	10.100089	4.672987	1.284884
1	9.003000	4.221915	2.608790
6	9.566016	2.473236	-0.359687
1	8.956919	1.912937	-1.073095
1	10.060488	3.286939	-0.907165
1	10.365042	1.810900	-0.000394

9a-Int7b

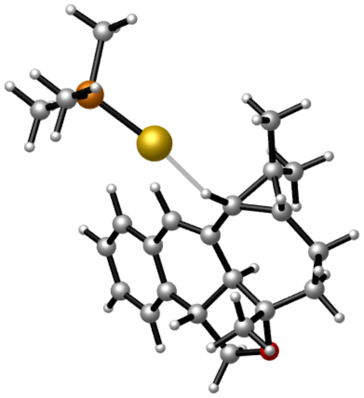

E = -1448.58561868 Hartrees
G = -1448.136303 Hartrees

6	3.813003	5.577092	1.774976
1	4.577617	6.333302	1.983515
1	2.833724	6.073302	1.736309
6	4.081271	4.788153	0.487880
1	5.167351	4.658030	0.367596
6	3.551893	5.253583	-0.840895
6	3.305701	6.584188	-1.170516
1	3.439875	7.357924	-0.418985
6	2.892519	6.931718	-2.462530
1	2.693961	7.971744	-2.705565
6	2.744545	5.942039	-3.436620
1	2.431184	6.208275	-4.442194

Enyne 32

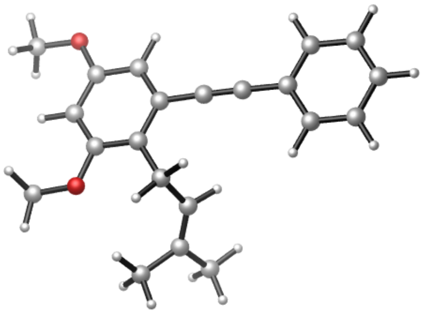

E = - 963.859114793 Hartrees
G = - 963.551049 Hartrees

6	-5.187467	-4.715371	-4.531455
6	-4.601578	-4.078714	-3.422135
6	-4.168274	-2.749952	-3.515275
6	-4.316619	-2.032730	-4.732263
6	-4.909456	-2.684618	-5.847004
6	-5.338899	-4.013477	-5.741429
1	-5.524684	-5.754571	-4.453388
1	-4.481628	-4.621491	-2.478098
1	-3.711609	-2.251466	-2.654086
1	-5.022626	-2.134143	-6.786801
1	-5.793954	-4.505235	-6.608200
6	-3.875493	-0.683496	-4.842354
6	-3.491072	0.479958	-4.953423

6	2.998384	4.606470	-3.118216
1	2.875803	3.834876	-3.875177
6	3.393847	4.242292	-1.822440
6	3.617911	2.835744	-1.466960
1	3.661504	2.126944	-2.296829
6	3.675043	2.389696	-0.190842
6	3.751208	0.933337	0.130746
1	4.580138	0.427309	-0.433557
6	3.562330	0.228718	1.482574
6	3.401836	0.820662	2.890212
1	4.332969	0.591426	3.420041
1	2.624344	0.232212	3.394497
6	3.099386	2.313031	3.116538
1	2.050493	2.549189	2.906930
1	3.253796	2.522210	4.182113
6	3.987610	3.262702	2.310122
6	3.480019	3.435759	0.873682
1	2.392338	3.601570	0.964565
6	5.474417	2.891939	2.427419
1	6.107854	3.666570	1.987987
1	5.700947	1.943785	1.930093
1	5.744872	2.802376	3.484908
8	3.802067	4.602749	2.841589
79	4.831071	-0.230423	-2.318141
15	5.564771	-0.973005	-4.352989
6	7.197685	-1.786669	-4.243303
1	7.502182	-2.122093	-5.240104
1	7.933513	-1.079801	-3.851767
1	7.131632	-2.645535	-3.570290
6	4.425107	-2.185803	-5.109407
1	3.440879	-1.729602	-5.240954
1	4.820575	-2.493504	-6.083246
1	4.331570	-3.058411	-4.458239
6	5.731284	0.396879	-5.552541
1	6.096833	0.001855	-6.506061
1	4.756630	0.869435	-5.700505
1	6.435061	1.139268	-5.167949
1	4.198732	-0.653723	1.542433
6	2.510275	0.074775	0.415140
6	1.175107	0.790984	0.524650
1	1.243024	1.753736	1.032140
1	0.756718	0.968582	-0.473452
1	0.464758	0.170316	1.082783
6	2.392050	-1.278740	-0.269907
1	2.031449	-1.162176	-1.301102
1	3.352139	-1.809007	-0.294729
1	1.678925	-1.919079	0.263691

6	-3.034741	1.819827	-5.124310
6	-3.290621	2.494069	-6.351663
6	-2.319972	2.446168	-4.075599
6	-2.777038	3.804221	-6.494678
6	-1.840962	3.754154	-4.246433
1	-2.130937	1.919840	-3.136410
6	-2.065665	4.442107	-5.457358
1	-1.692044	5.457250	-5.592383
6	-4.030308	1.818669	-7.495472
1	-4.805857	1.153937	-7.068744
1	-4.547366	2.579380	-8.101071
6	-3.086166	0.986322	-8.341908
1	-2.703240	0.089235	-7.832490
6	-2.651048	1.241173	-9.596975
6	-1.692149	0.295346	-10.29034
1	-0.749252	0.812845	-10.55852
1	-1.438516	-0.577149	-9.663298
1	-2.120426	-0.077872	-11.24220
6	-3.051607	2.445434	-10.42089
1	-3.658030	3.172305	-9.859780
1	-2.149109	2.975634	-10.78401
1	-3.612490	2.135154	-11.32504
8	-1.160555	4.282538	-3.177671
8	-3.028415	4.402930	-7.704982
6	-0.642570	5.613300	-3.307668
1	-0.138816	5.829595	-2.354008
1	0.090628	5.685429	-4.134247
1	-1.451915	6.351389	-3.470041
6	-2.528758	5.726740	-7.927764
1	-2.972485	6.453848	-7.220555
1	-1.425192	5.763235	-7.848661
1	-2.829290	5.984407	-8.953938

3. References

1. a) C. J. Davis, T. E. Hurst, A. M. Jacob, C. J. Moody, *J. Org. Chem.* **2005**, *70*, 4414–4422. b) K. Molawi, N. Delpont, A. M. Echavarren, *Angew. Chem. Int. Ed.* **2010**, *49*, 3617–3519.
2. A. Link, C. Sparr, *Angew. Chem. Int. Ed.* **2018**, 57, 7136–7139.
3. M. Völkert, K. Uwai, A. Tebbe, B. Popkirova, M. Wagner, J. Kuhlmann, H. Waldmann, *J. Am. Chem. Soc*. **2003**, *125*, 12749–12758.
4. T.-S. Mei, H. H. Patel, M. Sigman, *Nature* **2014**, *508*, 340–344.
5. Gaussian 09, Revision B.1, Frisch, M. J., Trucks, G. W., Schlegel, H. B., Scuseria, G. E., Robb, M. A., Cheeseman, J. R., Scalmani, G., Barone, V., Mennucci, B., Petersson, G. A., Nakatsuji, H., Caricato, M., Li, X., Hratchian, H. P., Izmaylov, A. F., Bloino, J., Zheng, G., Sonnenberg, J. L., Hada, M., Ehara, M., Toyota, K., Fukuda, R., Hasegawa, J., Ishida, M., Nakajima, T., Honda, Y., Kitao, O., Nakai, H., Vreven, T., Montgomery, J. A., Peralta, Jr. J. E., Ogliaro, F., Bearpark, M., Heyd, J. J., Brothers, E., Kudin, K. N., Staroverov, V. N., Kobayashi, R., Normand, J., Raghavachari, K., Rendell, A., Burant, J. C., Iyengar, S. S., Tomasi, J., Cossi, M., Rega, N., Millam, J. M., Klene, M., Knox, J. E., Cross, J. B., Bakken, V., Adamo, C., Jaramillo, J., Gomperts, R., Stratmann, R. E., Yazyev, O., Austin, A. J., Cammi, R., Pomelli, C., Ochterski, J. W., Martin, R.L., Morokuma, K., Zakrzewski, V. G., Voth, G. A., Salvador, P., Dannenberg, J. J., Dapprich, S., Daniels, A. D., Farkas, Ö., Foresman, J. B., Ortiz, J. V., Cioslowski, J., Fox, D. J. Gaussian, Inc., Wallingford CT **2009**.
6. a) A. D. Becke, *J. Chem. Phys*. **1993**, *98*, 5648-5652. b) C. Lee, W. Yang, R. G. Parr, *Phys. Rev. B*. **1988**, *37*, 785. c) P. J. Stephens, F. J. Devlin, C. F. Chabalowsky, M. J. Frisch, *J. Phys. Chem.* **1994**, *98*, 11623-11627.
7. Y. Zhao, D. G. Truhlar, *Theor. Chem. Account.* **2008**, *120*, 215–241.
8. S. Grimme, J. Antony, S. Ehrlich, H. Krieg, *J. Chem. Phys.* **2010**, *132*, 154104.
9. W. J. Hehre, R. Ditchfield, J. A. Pople, *J. Chem. Phys.* **1972**, *56*, 2257–2261.
10. D. Andrae, U. Haussermann, M. Dolg, H. Stoll, H. Preuss, *Theor. Chim. Acta* **1990**, *77*, 123–141.
11. M. T. Cancès, B. B. Mennucci, J. Tomasi, *J. Chem. Phys.* **1997**, *107*, 3032−3041.
12. P. J. Hay, W. R. Wadt, *J. Chem. Phys*. **1985**, *82*, 270–283.
13. A. Höllwarth, M. Böhme, S. Dapprich, A. W. Ehlers, A. Gobbi, V. Jonas, K. F. Köhler, R. Stegmann, A.Veldkamp, G. Frenking, *Chem. Phys. Lett.* **1993**, *208*, 237–240.
14. a) L. Goerigk, S. Grimme, *Phys. Chem. Chem. Phys*. **2011**, *13*, 6670−6688. b) L. Goerigk, H. Kruse, S. Grimme, *ChemPhysChem*. **2011**, *12*, 3421−3433. c) F. O. Evers, F. Formalik, A. Olejniczak, M. Fischer, *Theor. Chem. Acc*. **2016**, *135*, 257. d) S. Dohm, A. Hansen, M. Steinmetz, S.Grimme, M. P. Checinski, *J. Chem. Theory Comput.* **2018**, *14*, 2596−2608.
15. A. D. Becke, *Phys. Rev. A*. **1988**, *38*, 3098–3100.
16. S. Grimme, *J. Comput. Chem*. **2006**, *27,* 1787–1799.
17. J. Tao, J. P. Perdew, *Phys. Rev. Lett*. **2003**, *91*, 146401.
18. J. P. Perdew, K. Burke, M. Ernzerhof, *Phys. Rev. Lett*. **1996**, 77, 3865–3868.
19. A. D. Becke, *J. Chem. Phys*. **1993**, *98*, 5648–5652.
20. A. D. Boese, J. M. Martin, *J. Chem. Phys*. **2004**, *121*, 3405–3416.
21. Y. Zhao,D. G. Truhlar, *J. Chem. Phys.* **2006**, *125*, 104101: 1–18.
22. C. Adamo, *J. Chem. Phys*. **1999**, *110*, 6158–6170.
23. C. Riplinger, B. Sandhoefer, A. Hansen, F. Neese, *J. Chem. Phys*. **2013**, *139*, 134101.
24. Neese, F. *WIREs Comput. Mol. Sci*. **2018**, *8*, e1327.
25. (a) C. R. Landis, F. Weinhold, *Cambridge University Press*, **2005**. (b) C. R. Landis, F. Weinhold, *J. Am. Chem. Soc*. **2006**, *128*, 7335–7345.
26. (a) R. F. W. Bader, *Clarendon Press*, **1994**. (b) R. F. W. Bader, *Chem. Eur. J.* **2006**, *12*, 7769–7772.
27. T. Lu, F. Chen, *J. Comput. Chem*. **2012**, *33*, 580–592.

4. **NMRS**

^{1}H-NMR and ^{13}C-NMR of **S1**

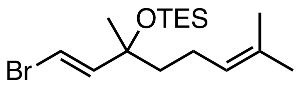

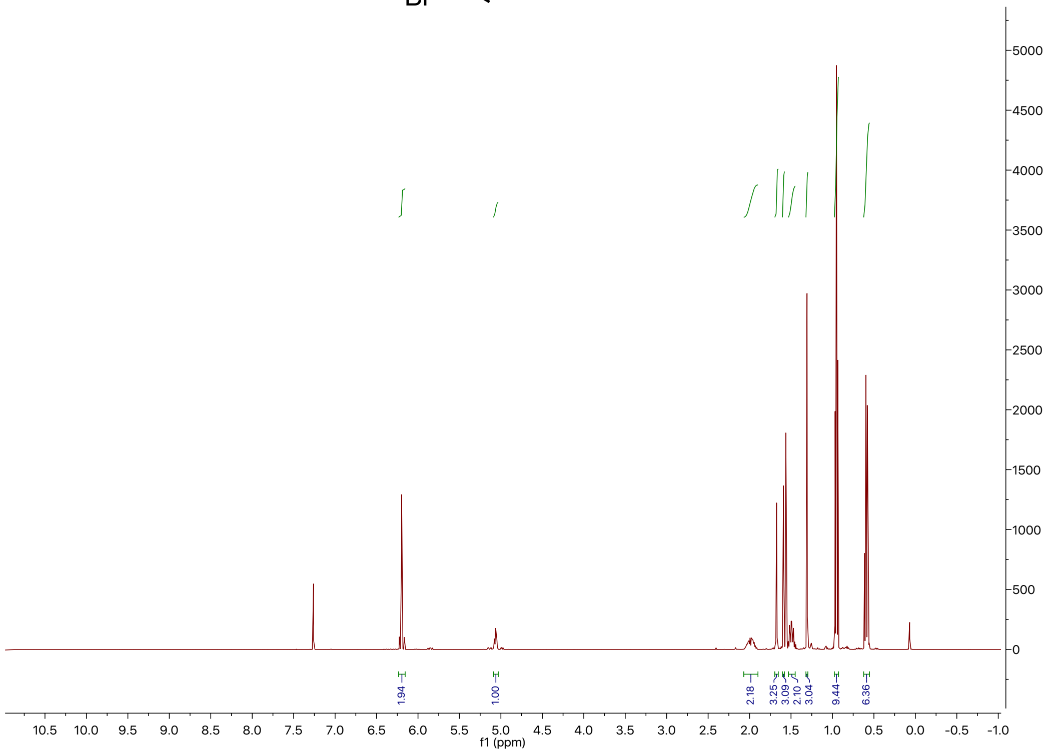

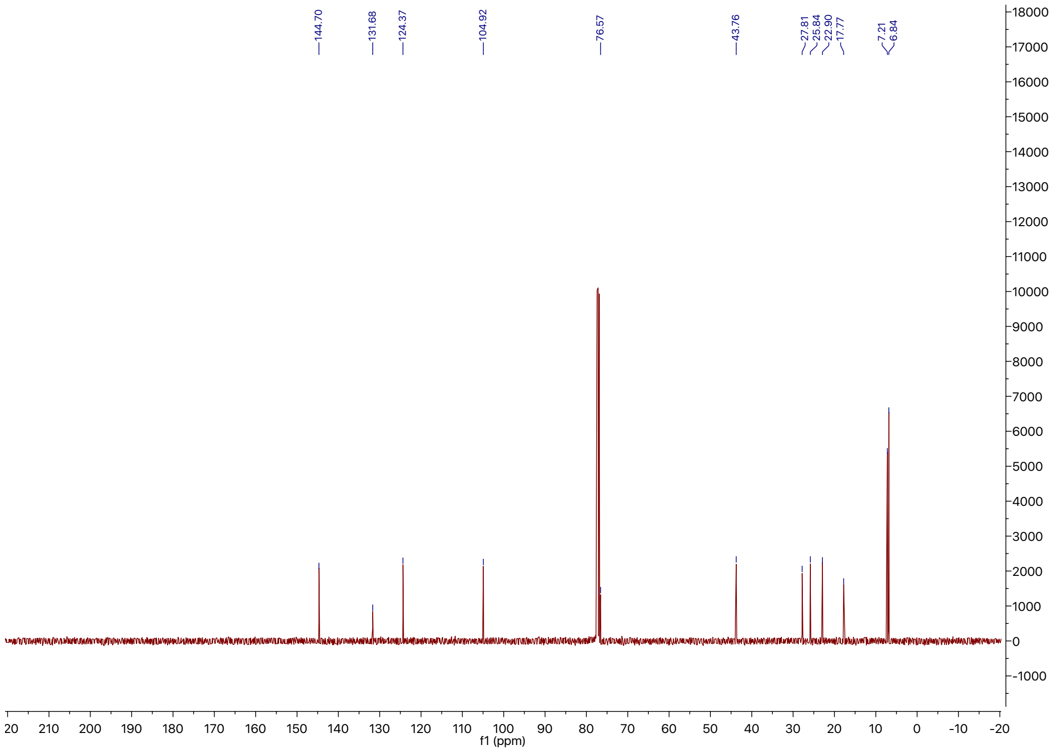

^{1}H-NMR and ^{13}C-NMR of **S2**

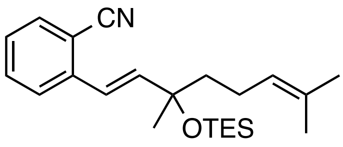

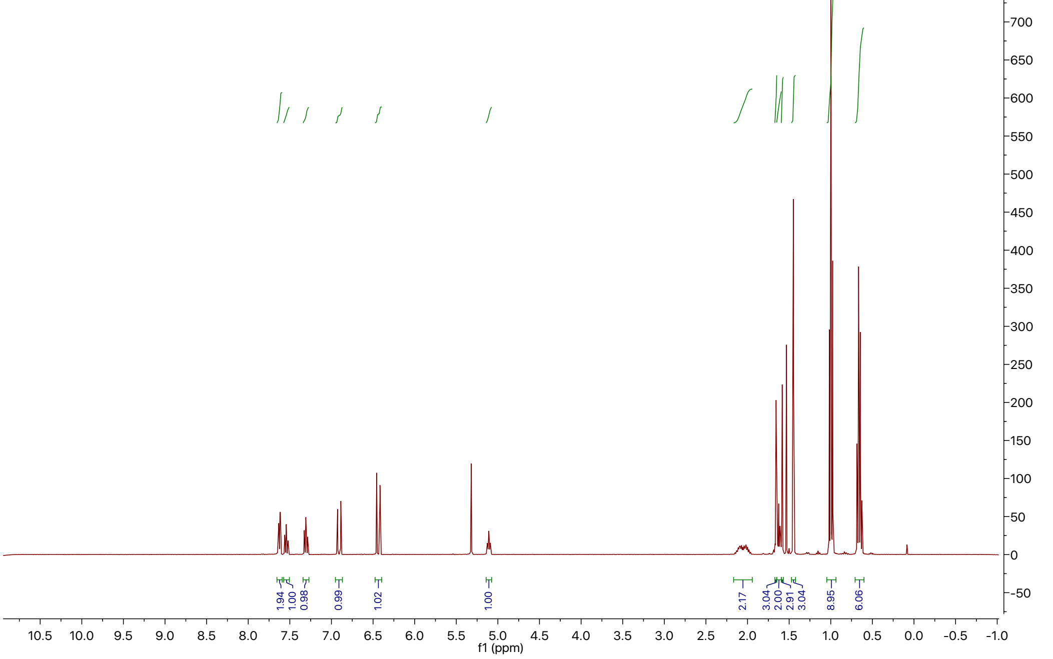

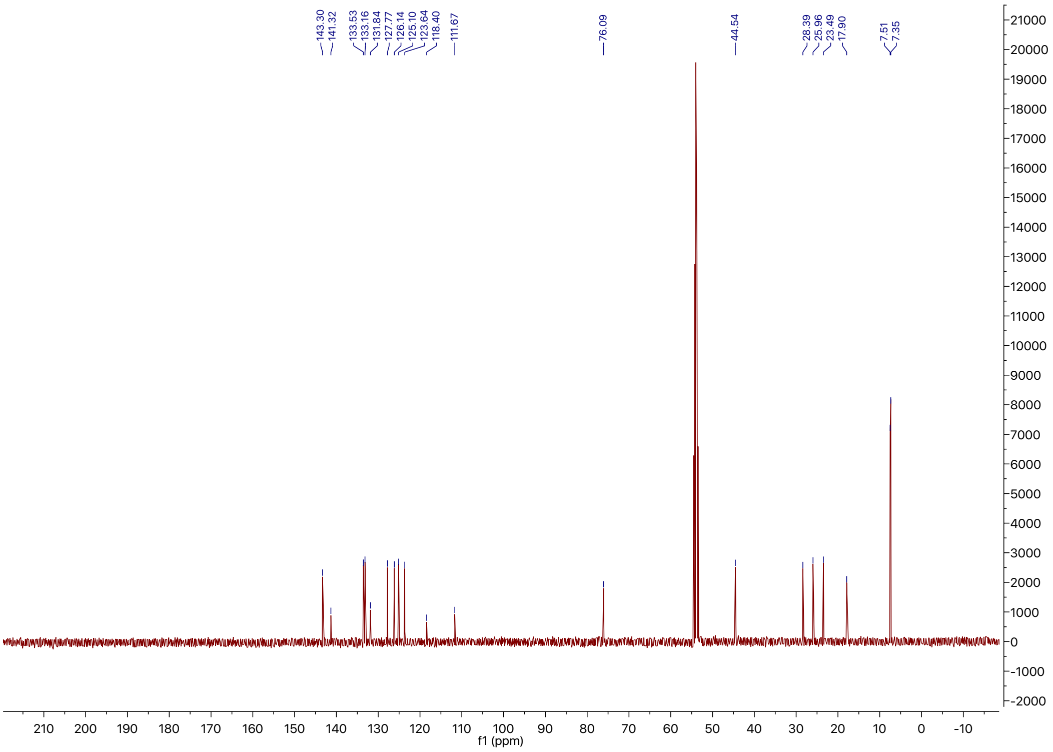

^{1}H-NMR and ^{13}C-NMR of **S3**

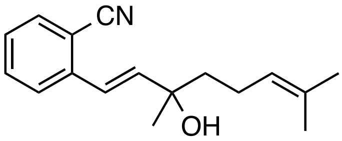

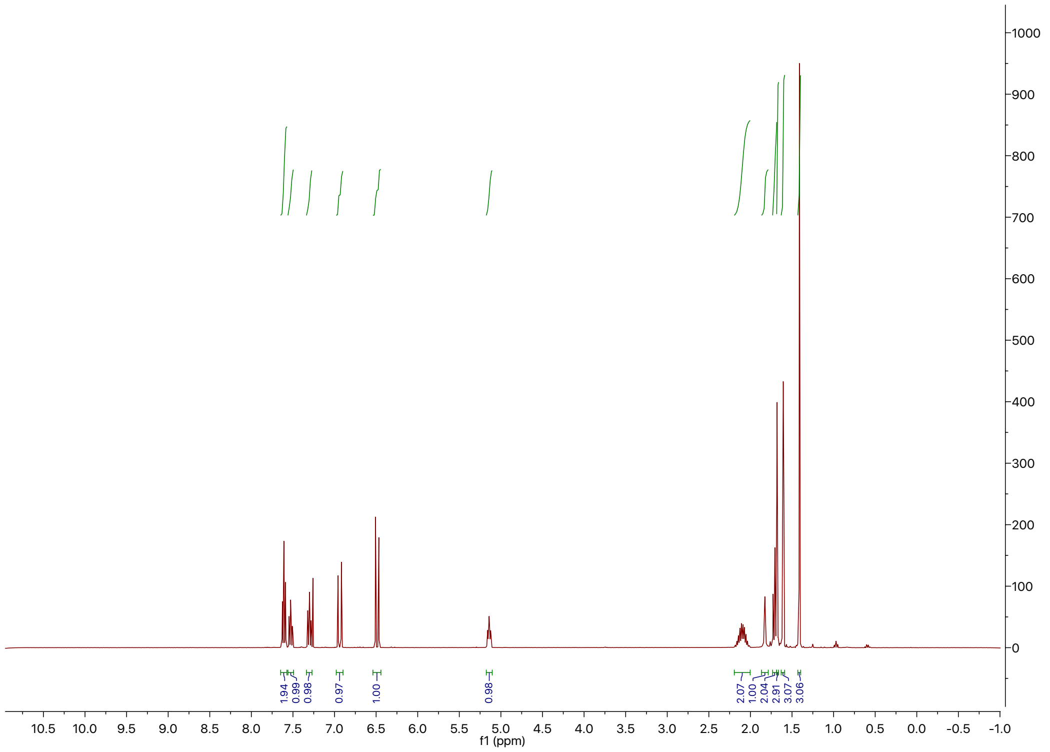

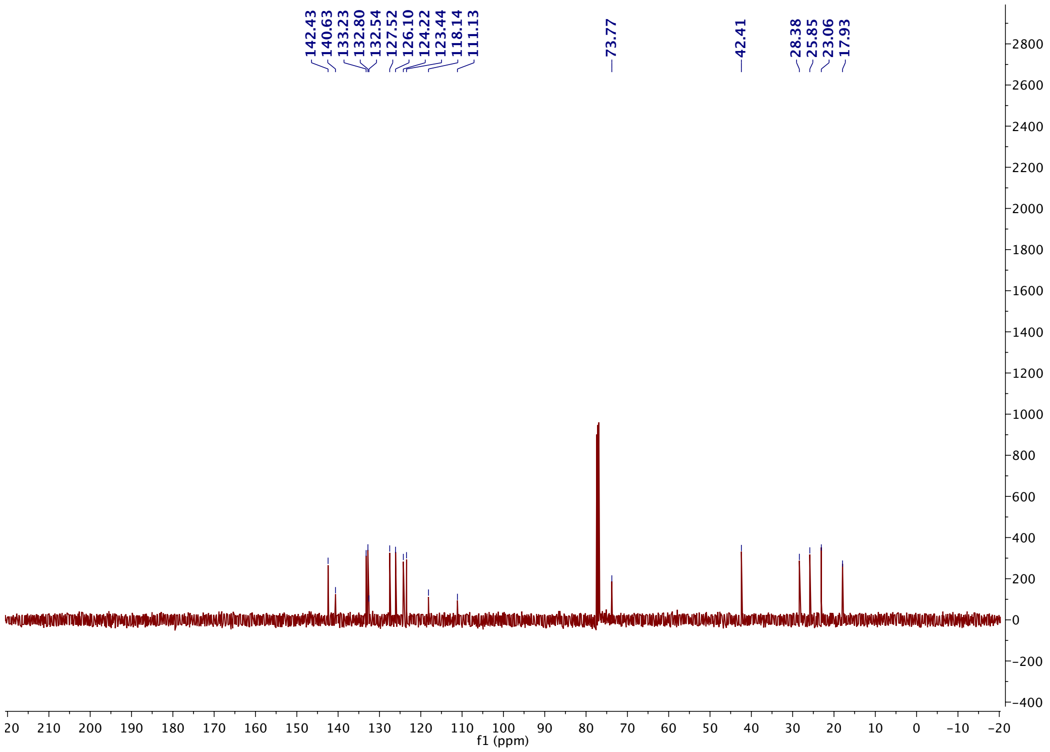

^{1}H-NMR and ^{13}C-NMR of **S4**

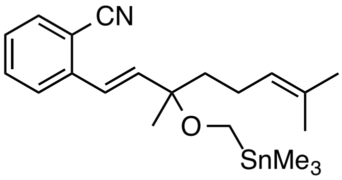

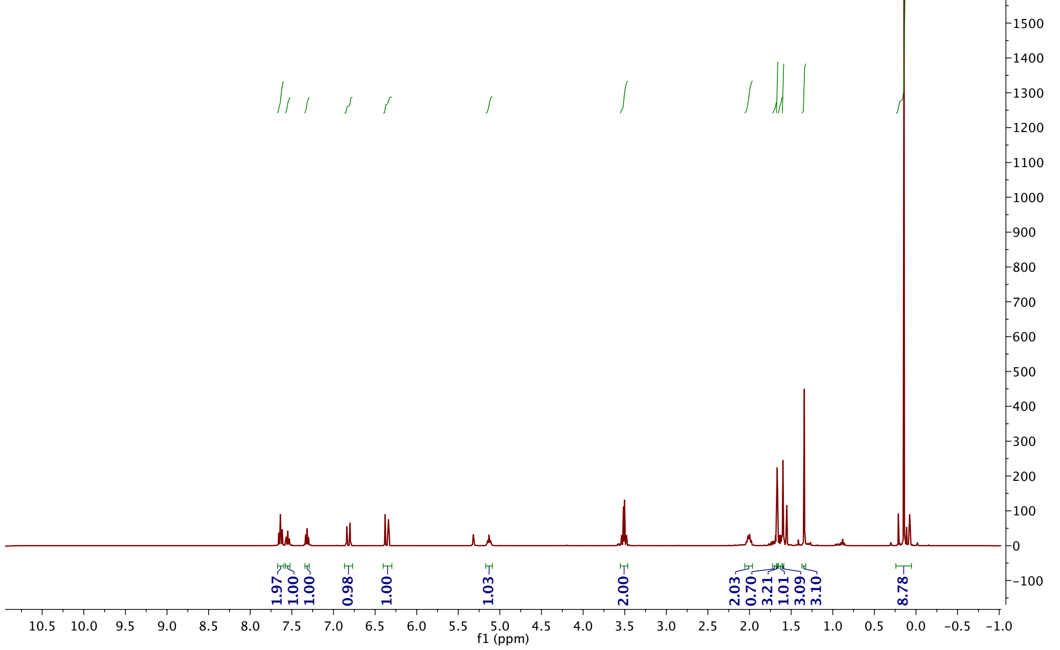

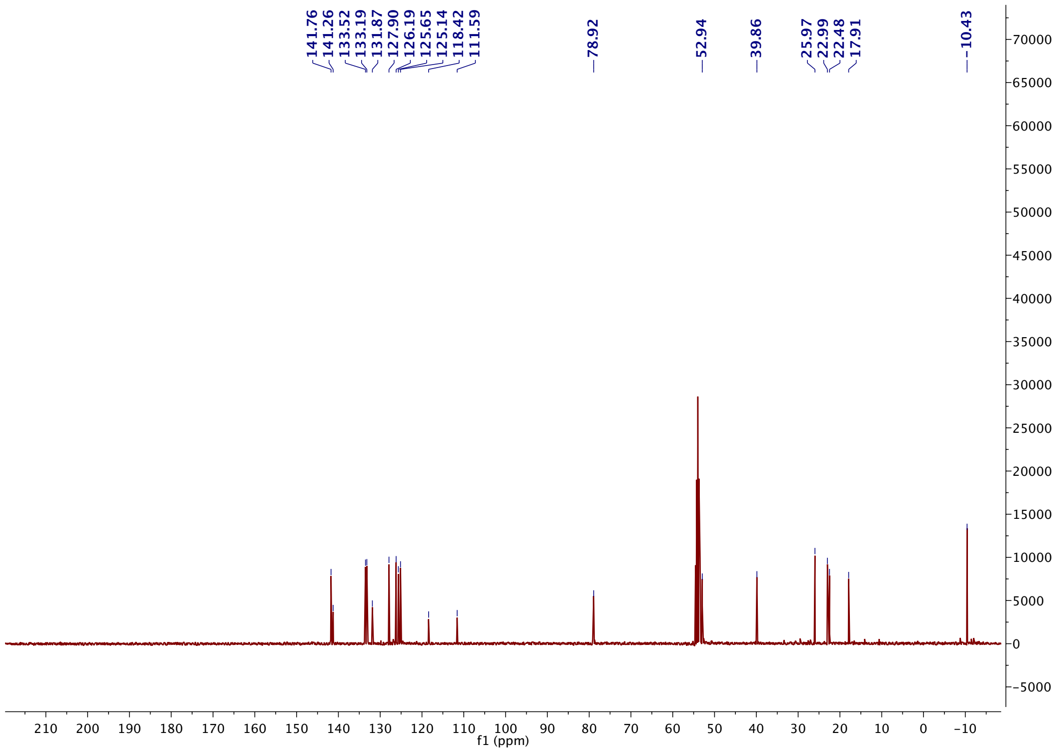

^{1}H-NMR and ^{13}C-NMR of **S5**

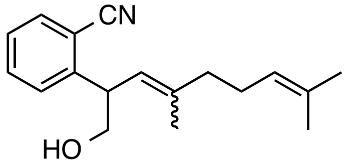

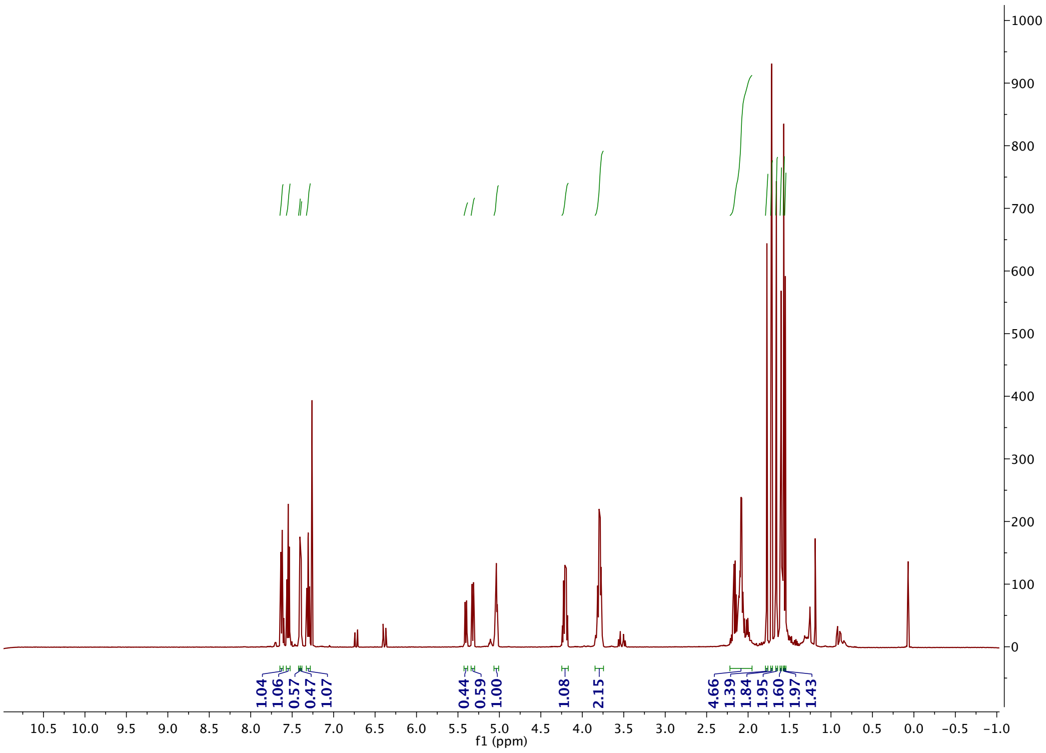

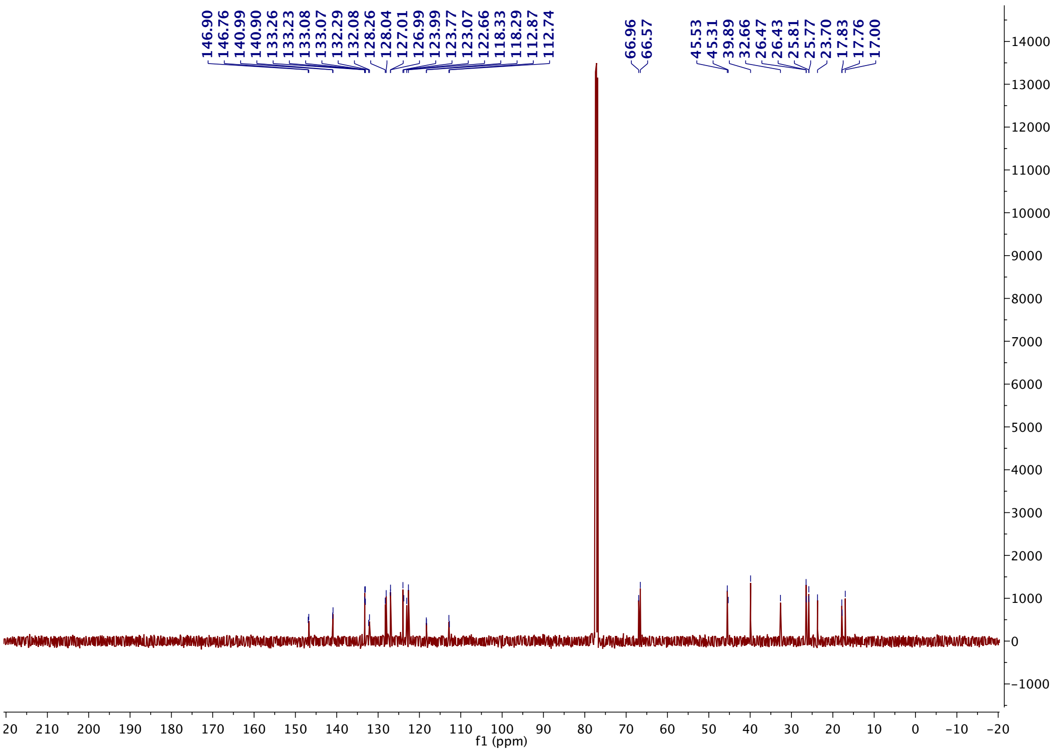

^{1}H-NMR and ^{13}C-NMR of **S6**

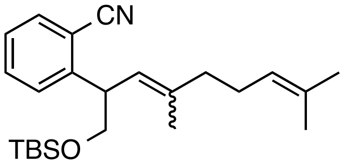

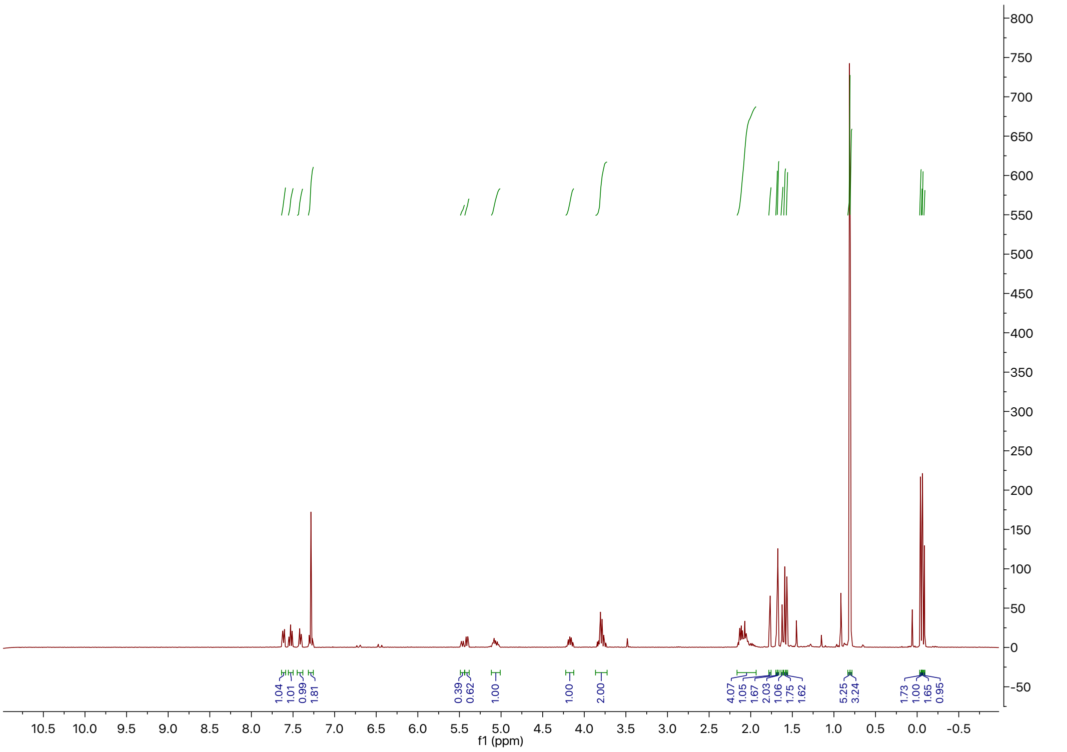

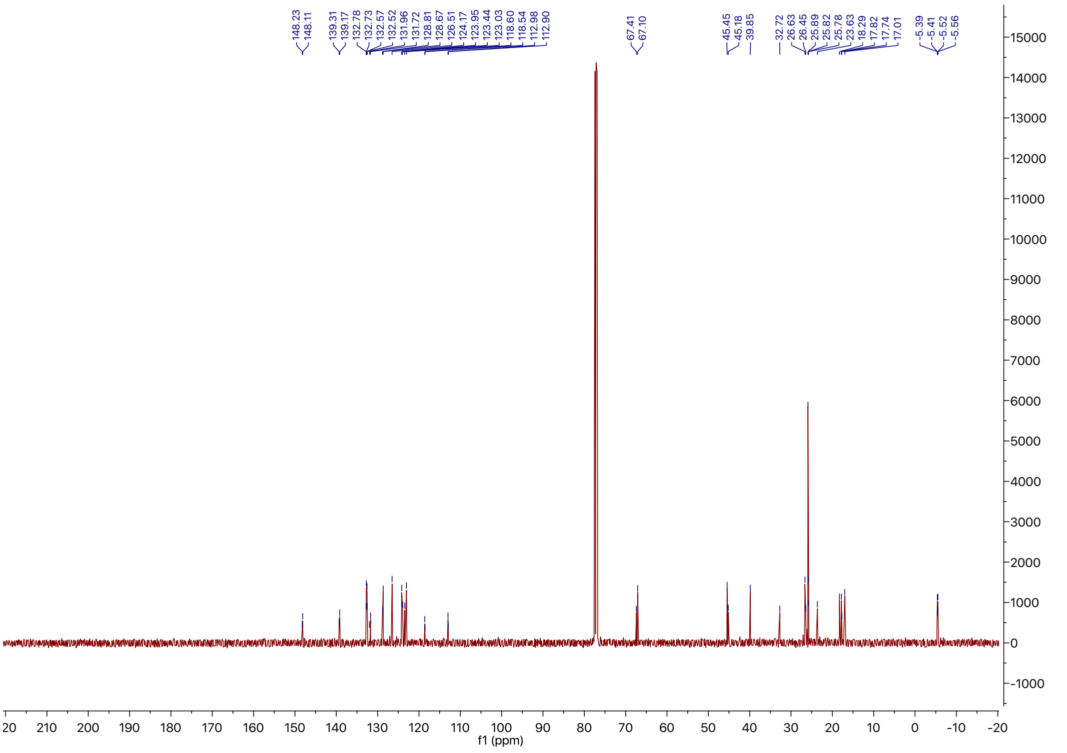

^{1}H-NMR and ^{13}C-NMR of **S7a**

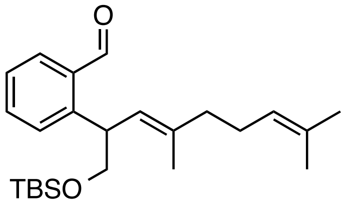

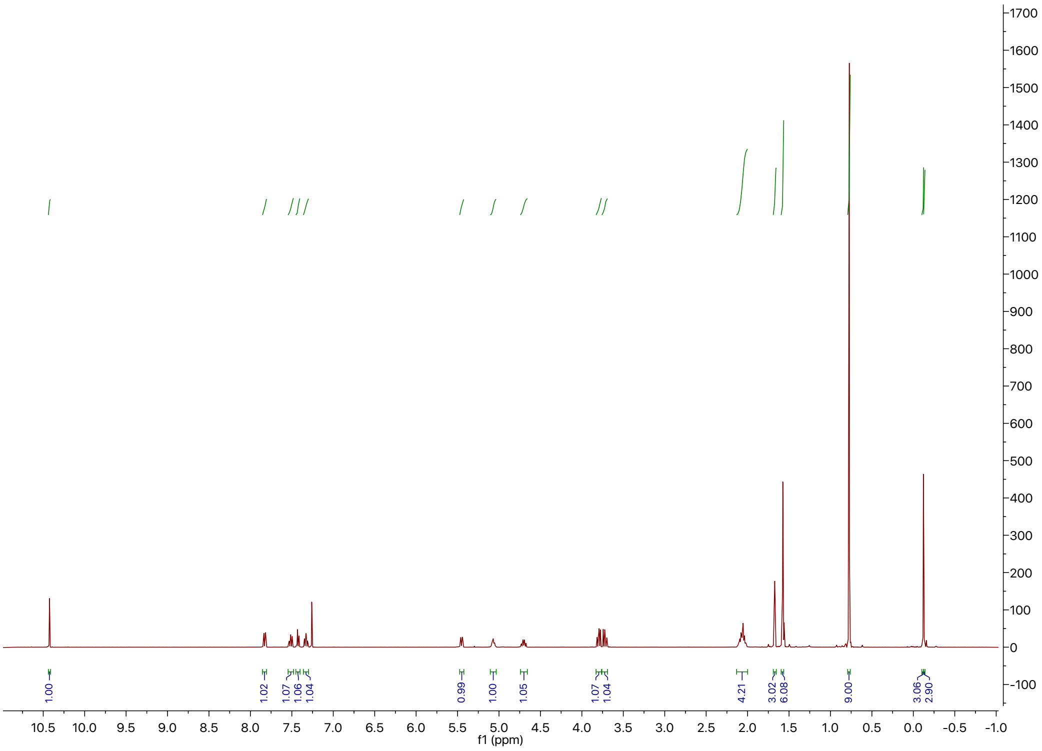

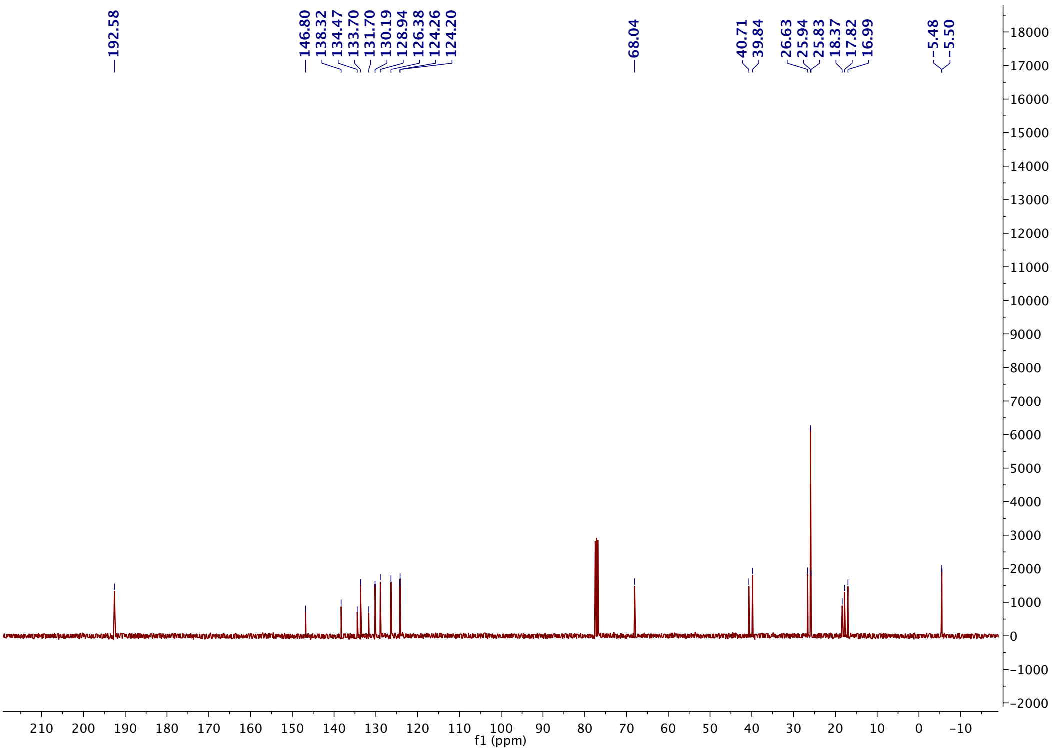

^{1}H-NMR and ^{13}C-NMR of **S7b**

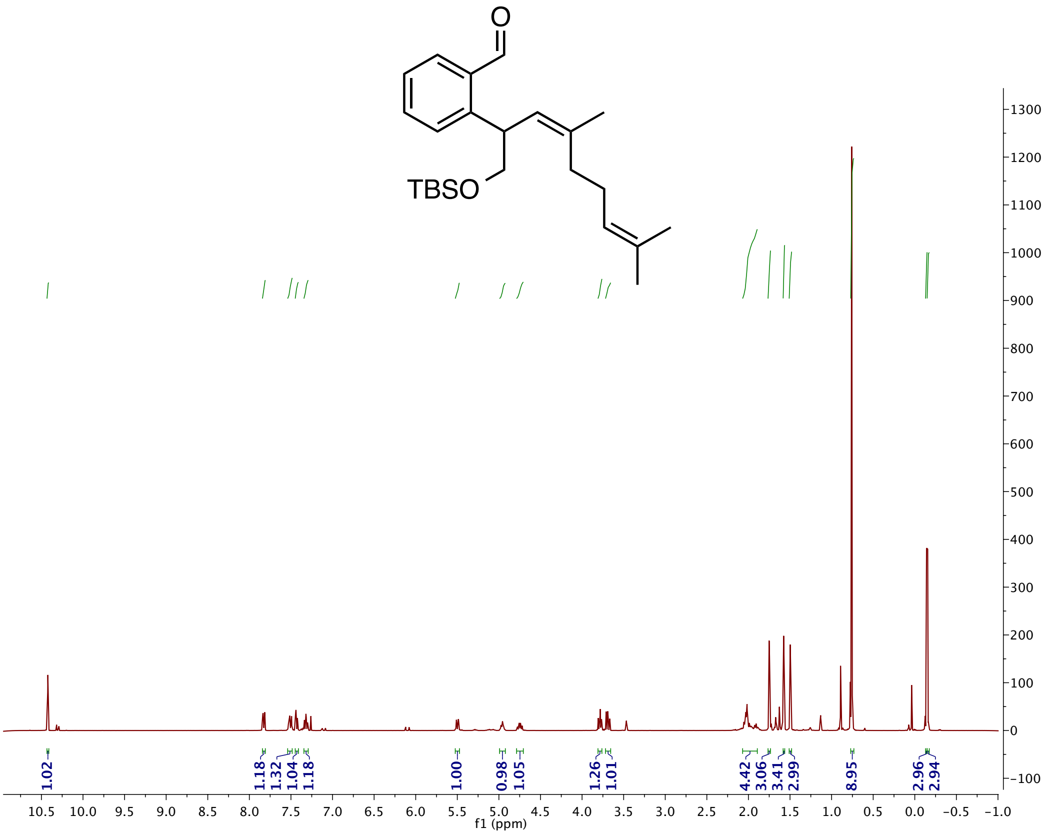

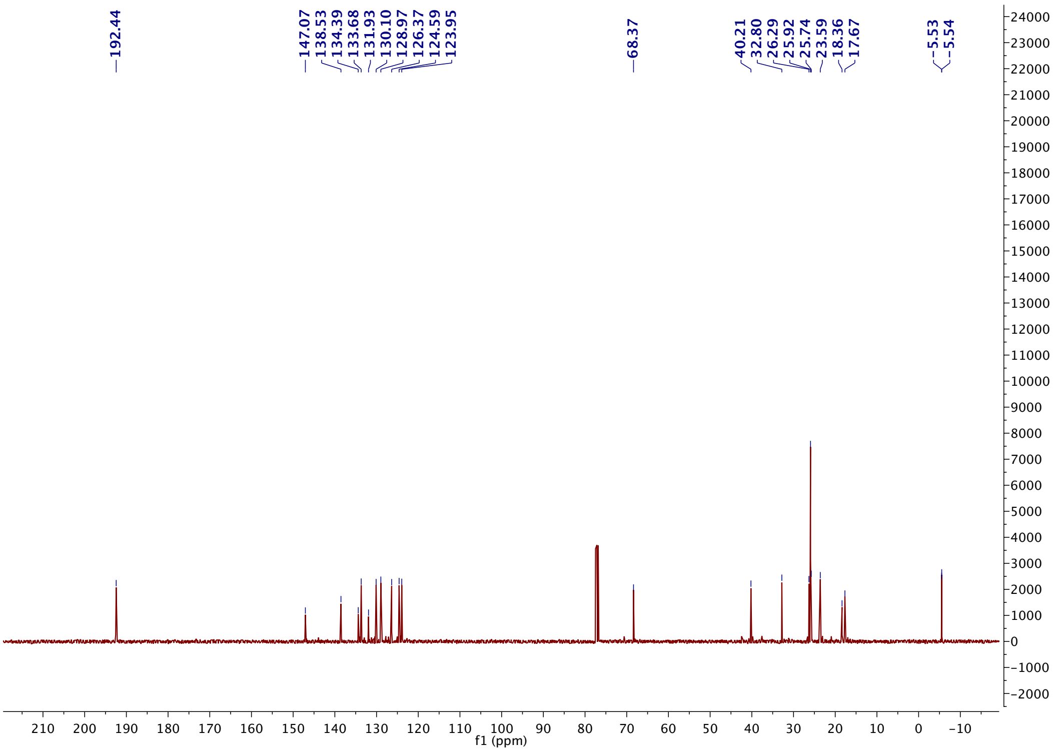

^{1}H-NMR and ^{13}C-NMR of **S8a**

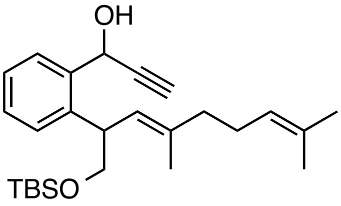

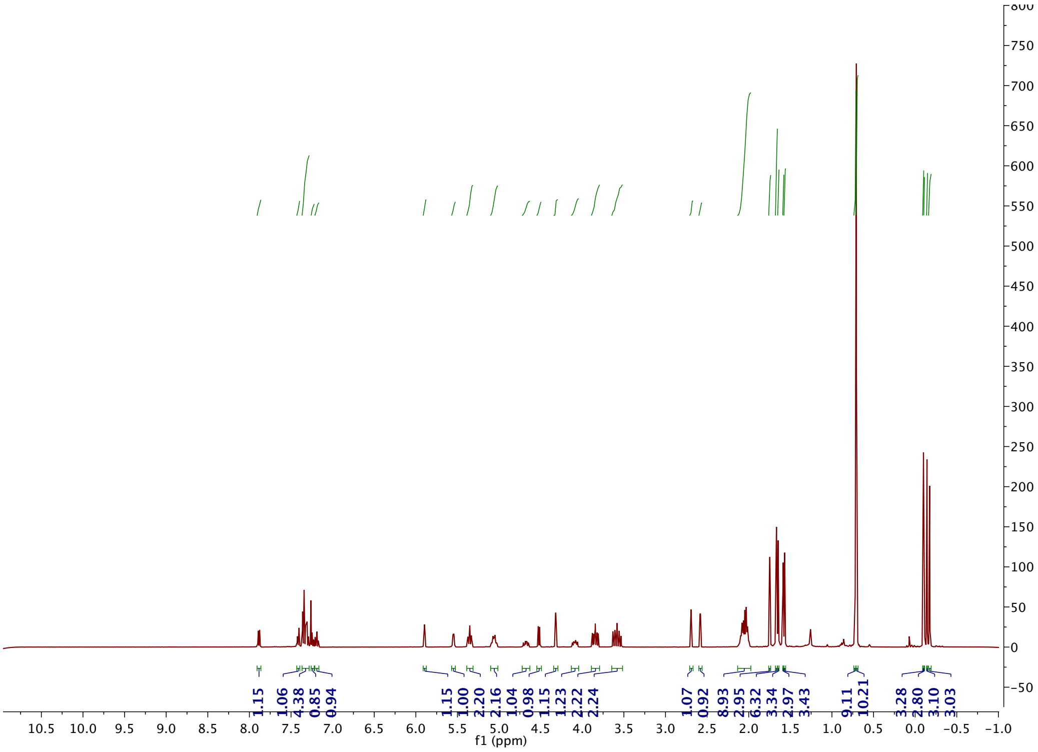

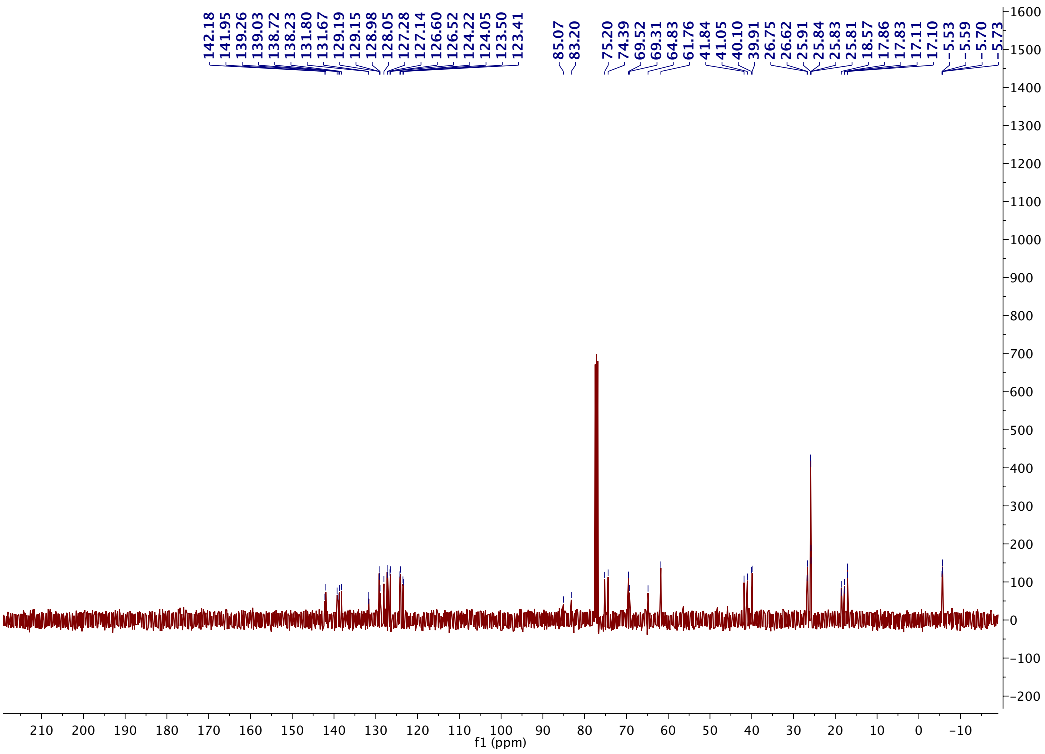

^{1}H-NMR and ^{13}C-NMR of **S8b**

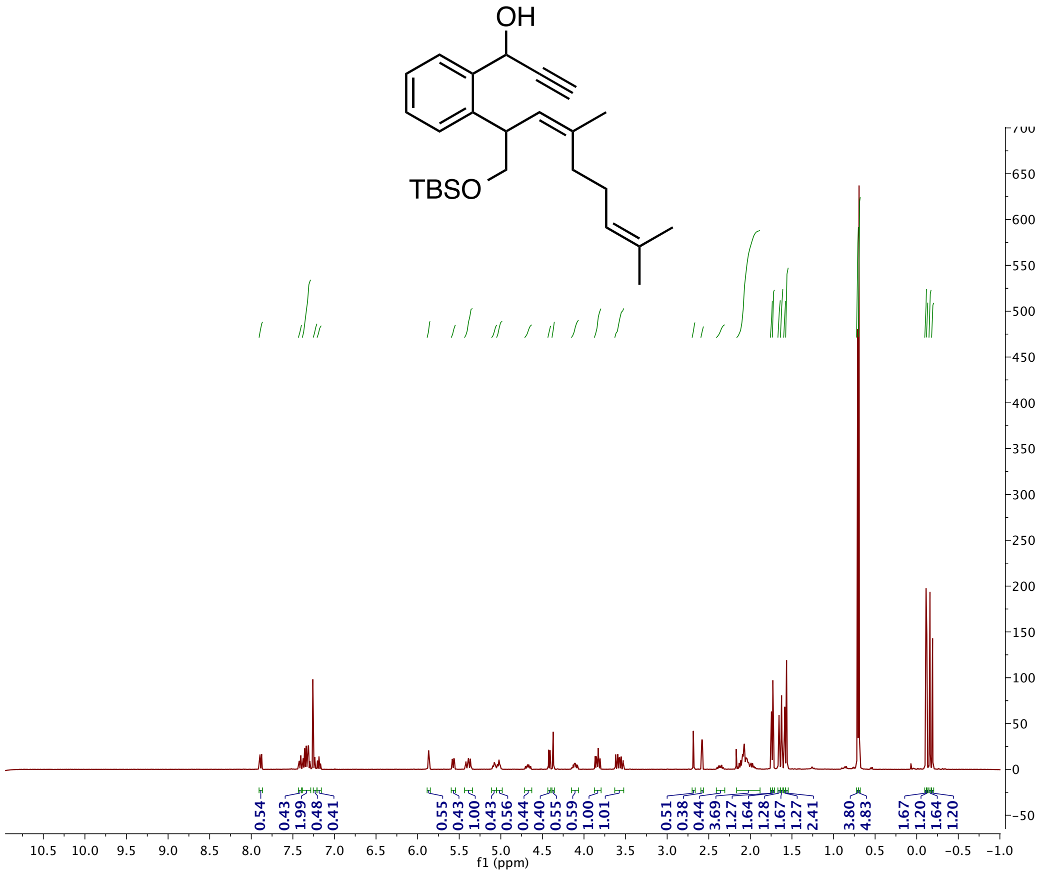

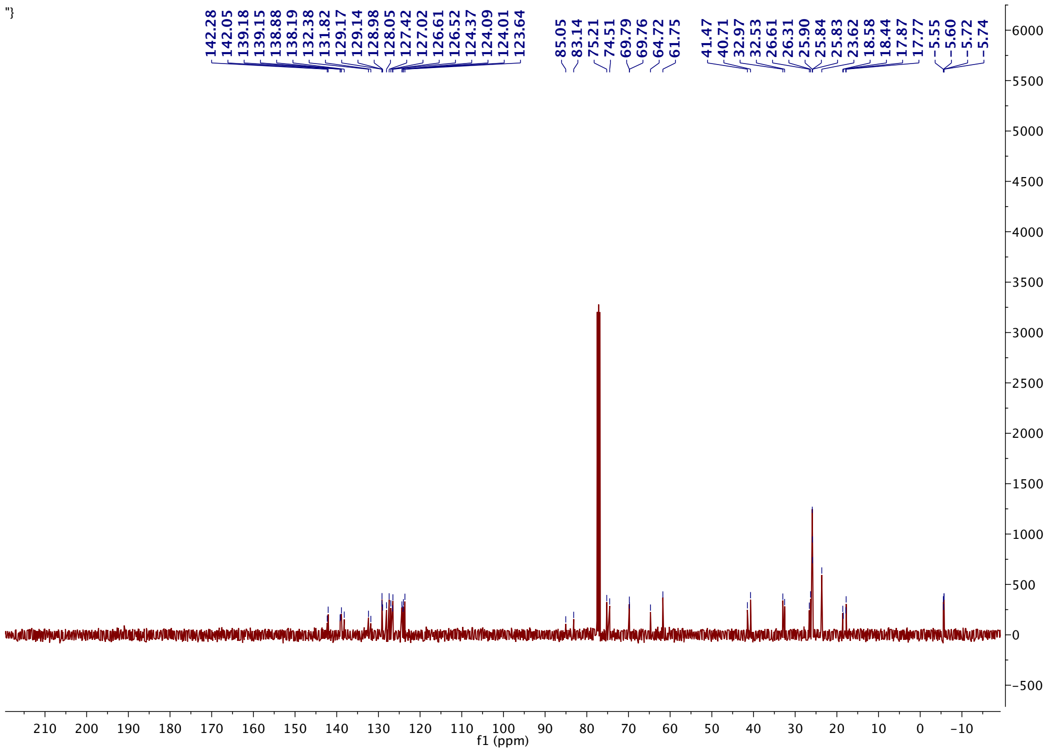

^{1}H-NMR and ^{13}C-NMR of **19a**

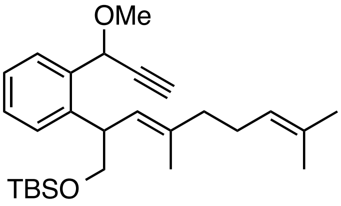

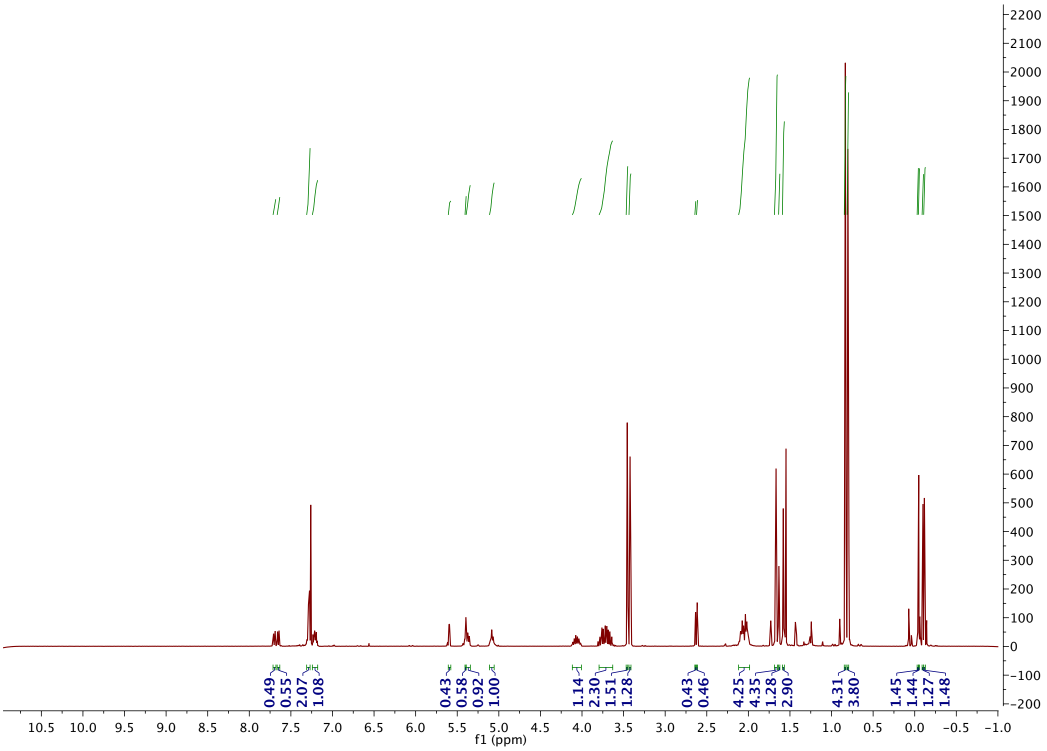

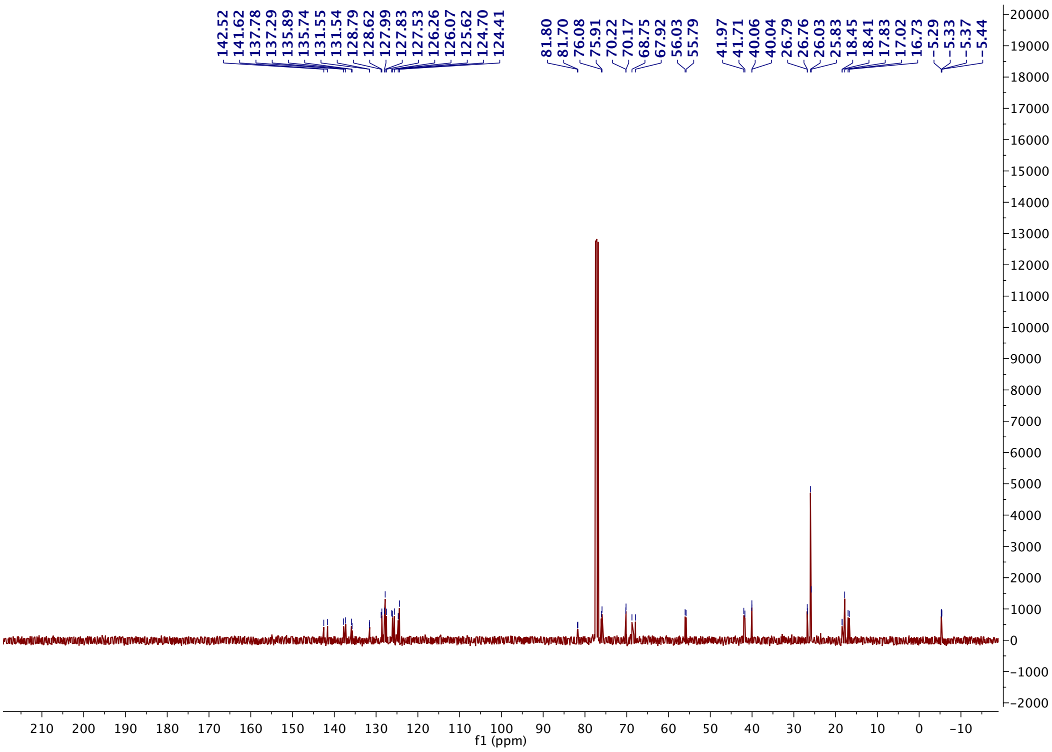

^{1}H-NMR and ^{13}C-NMR of **19b**

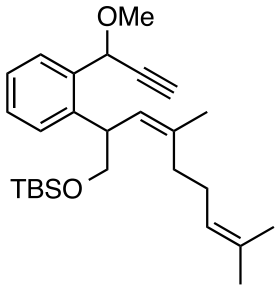

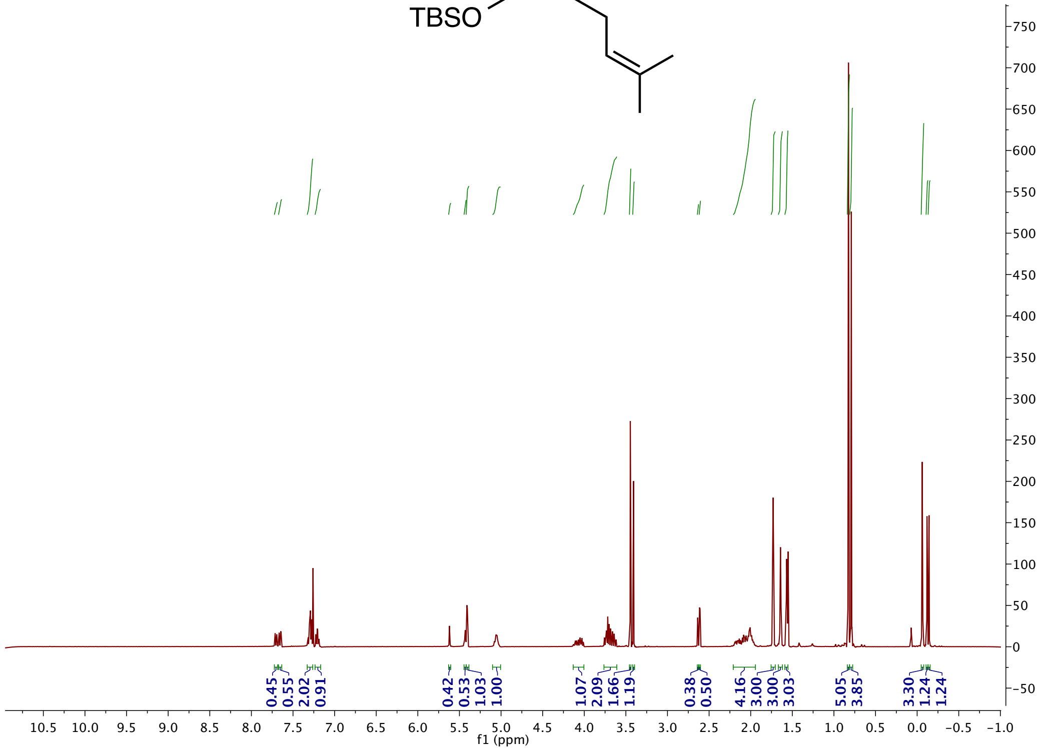

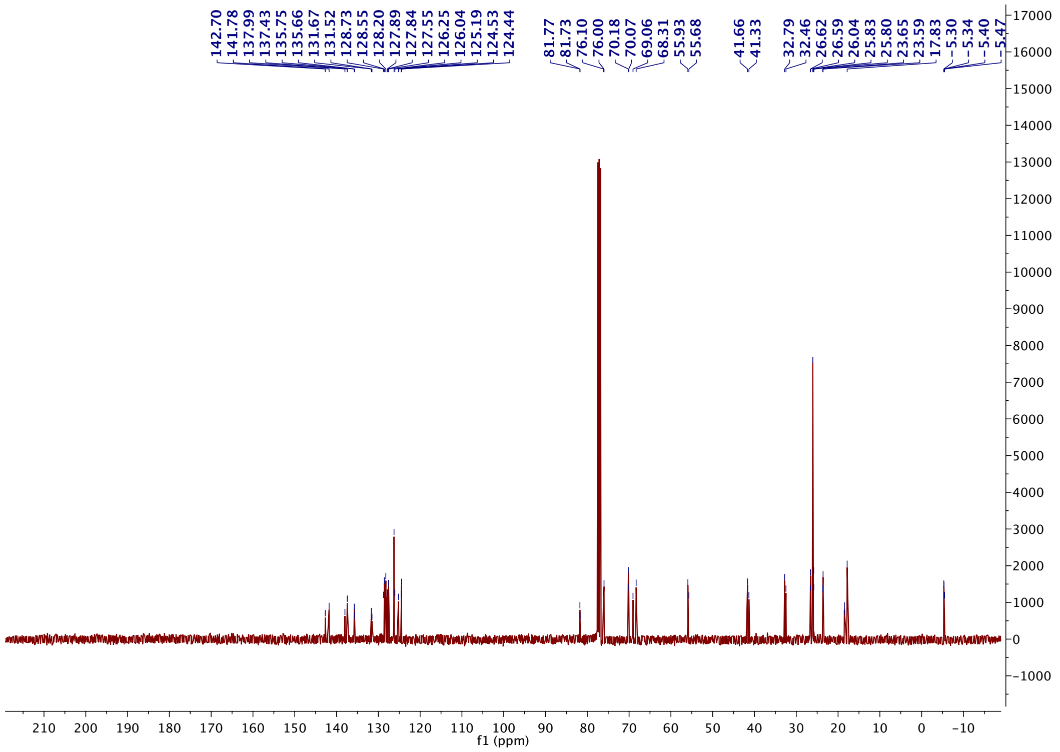

^{1}H-NMR and ^{13}C-NMR of **S9a**

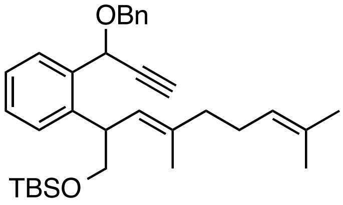

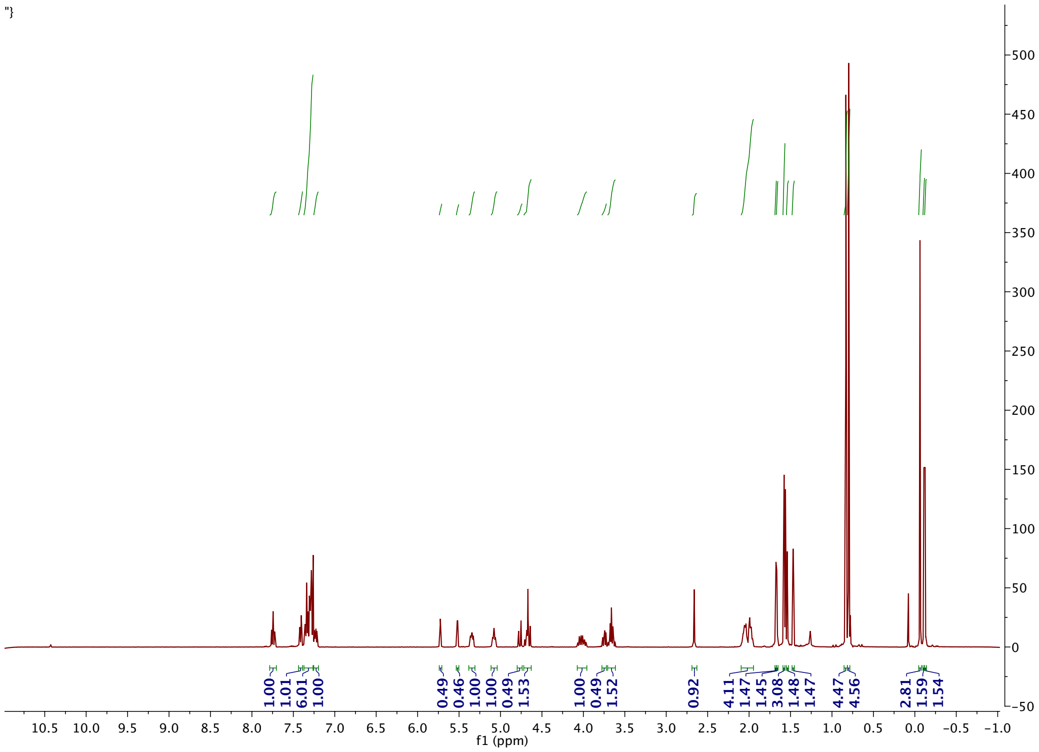

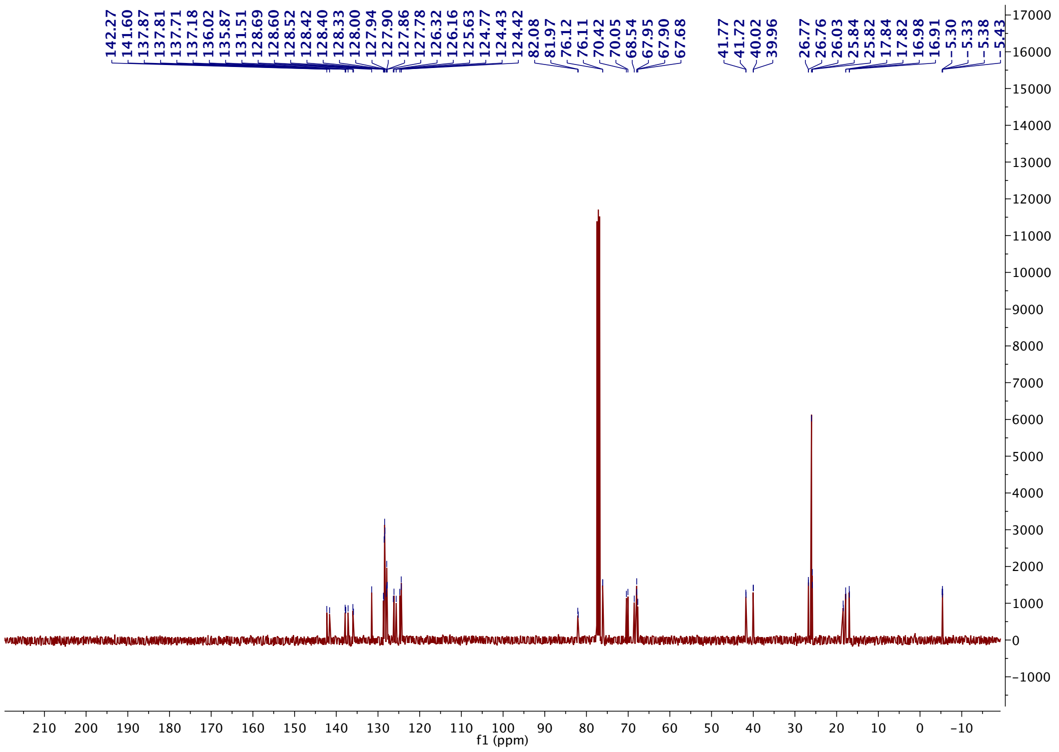

^{1}H-NMR and ^{13}C-NMR of **S9b**

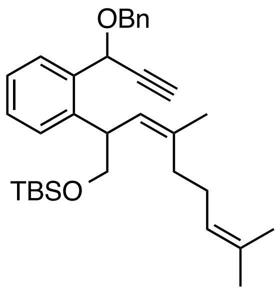

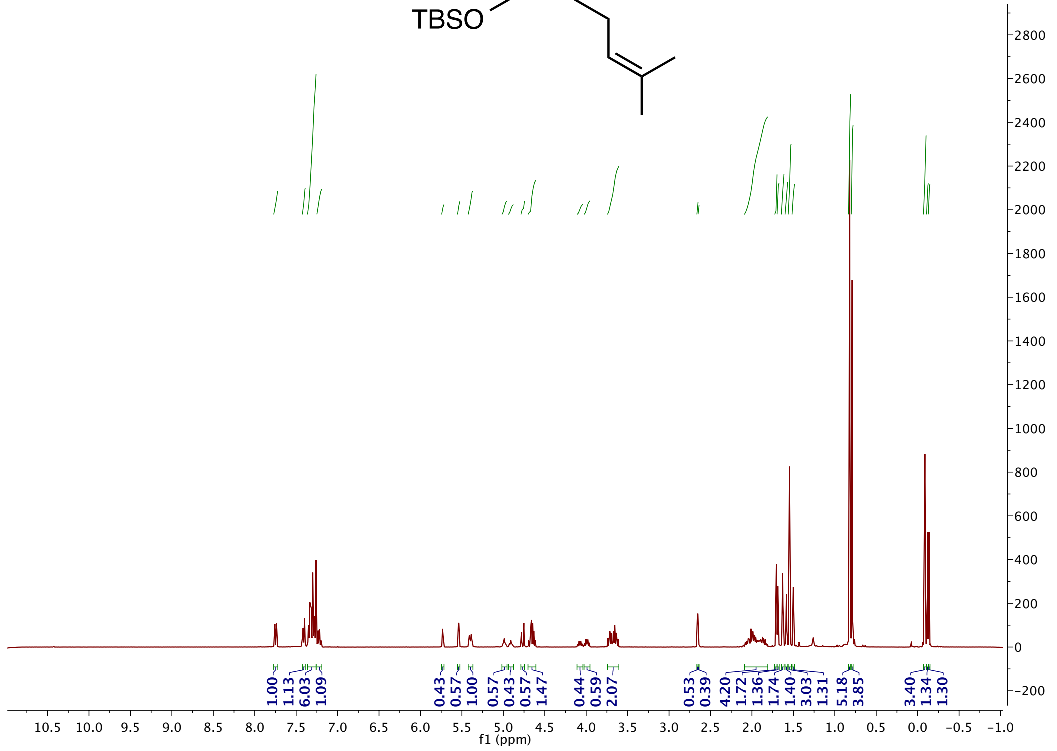

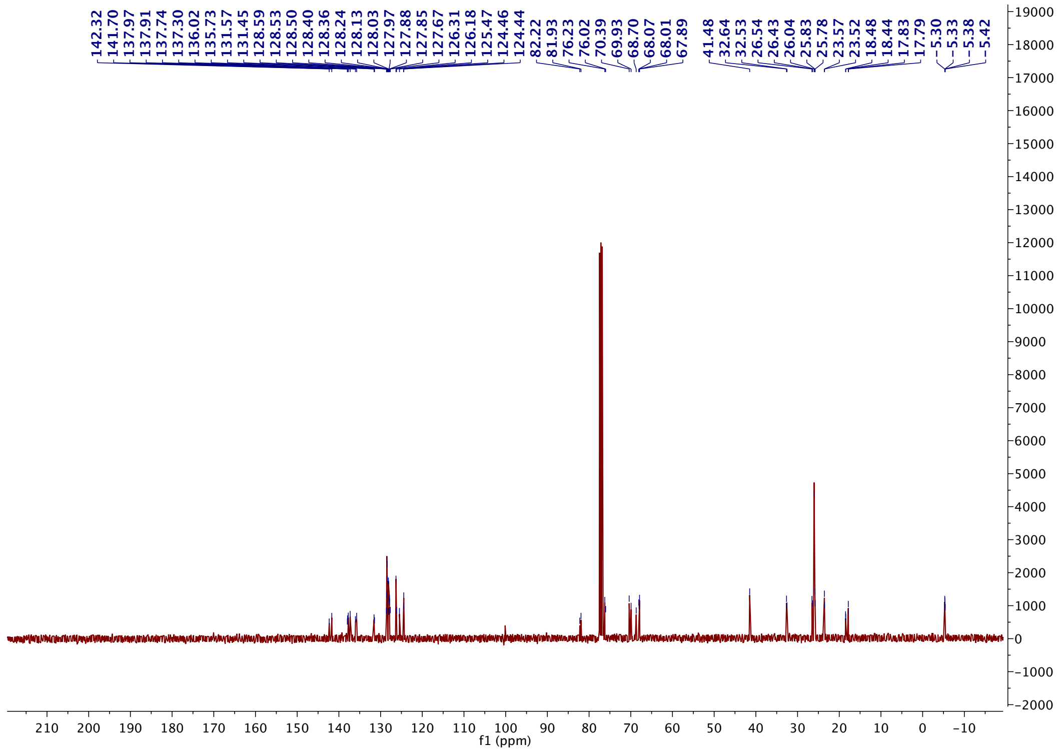

^{1}H-NMR and ^{13}C-NMR of **9a**

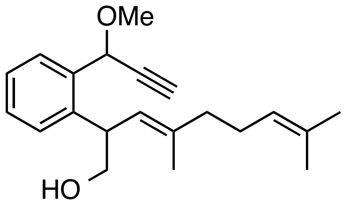

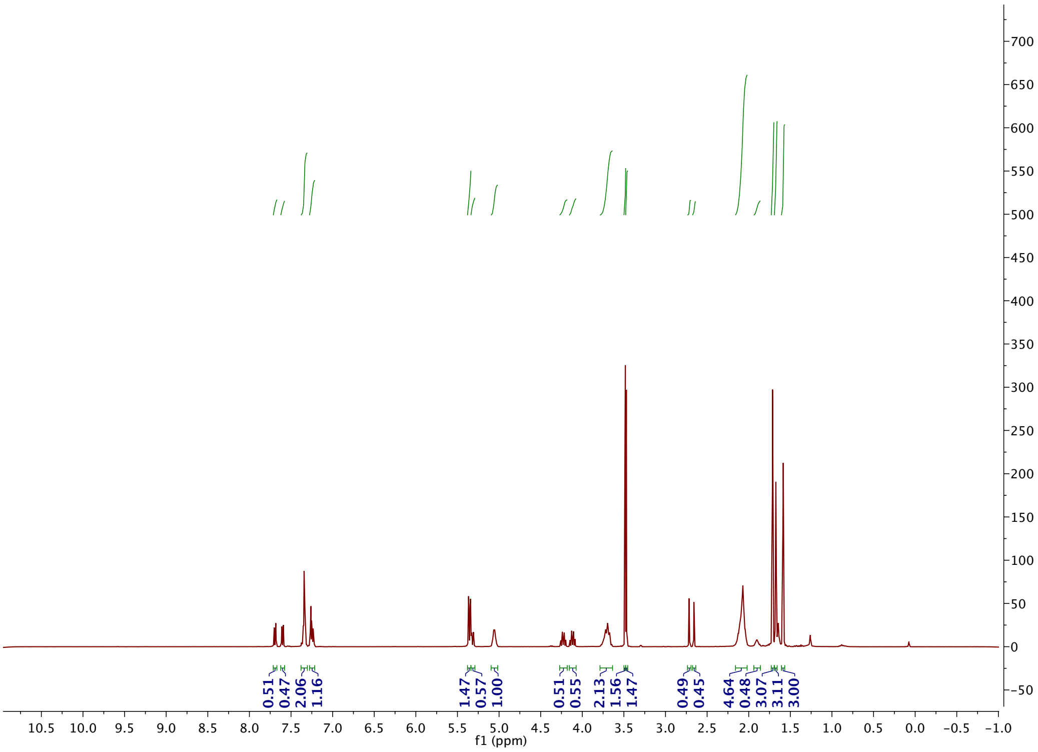

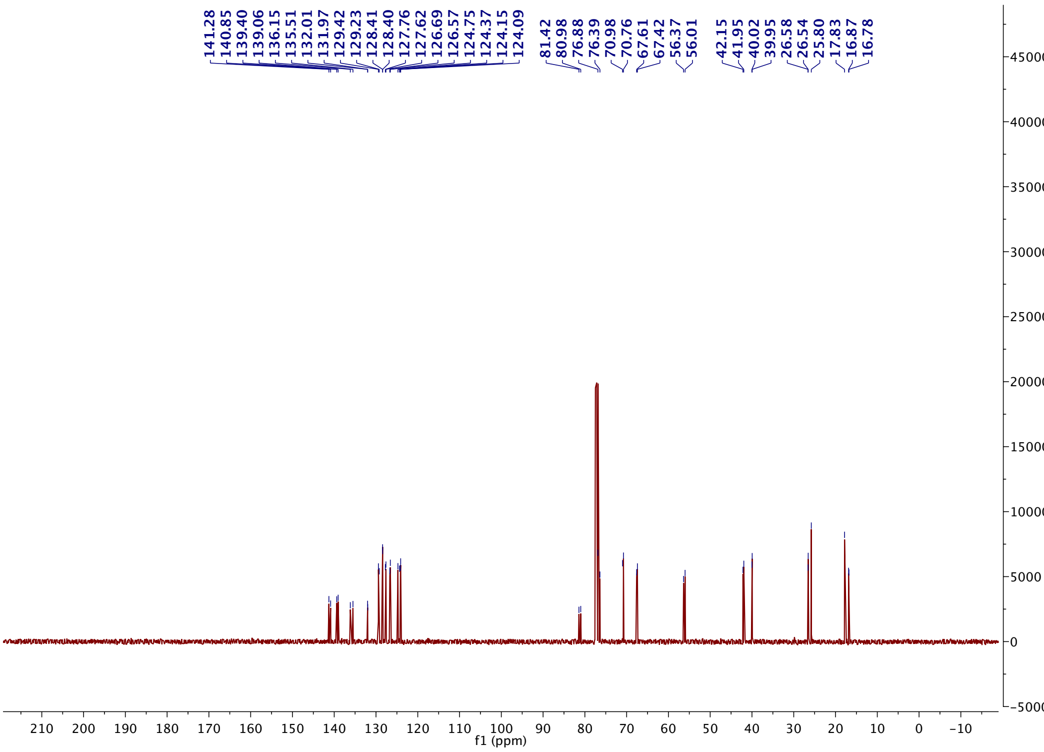

^{1}H-NMR and ^{13}C-NMR of **9b isomer A**

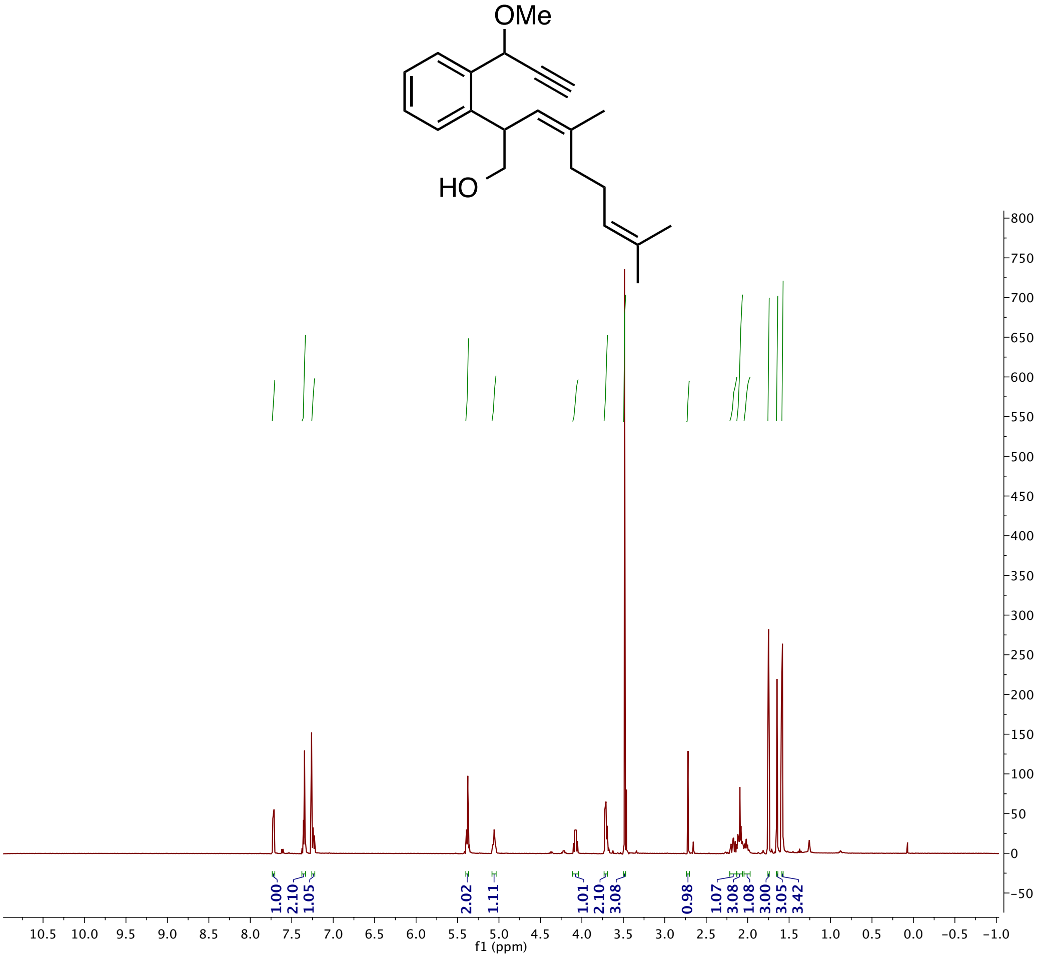

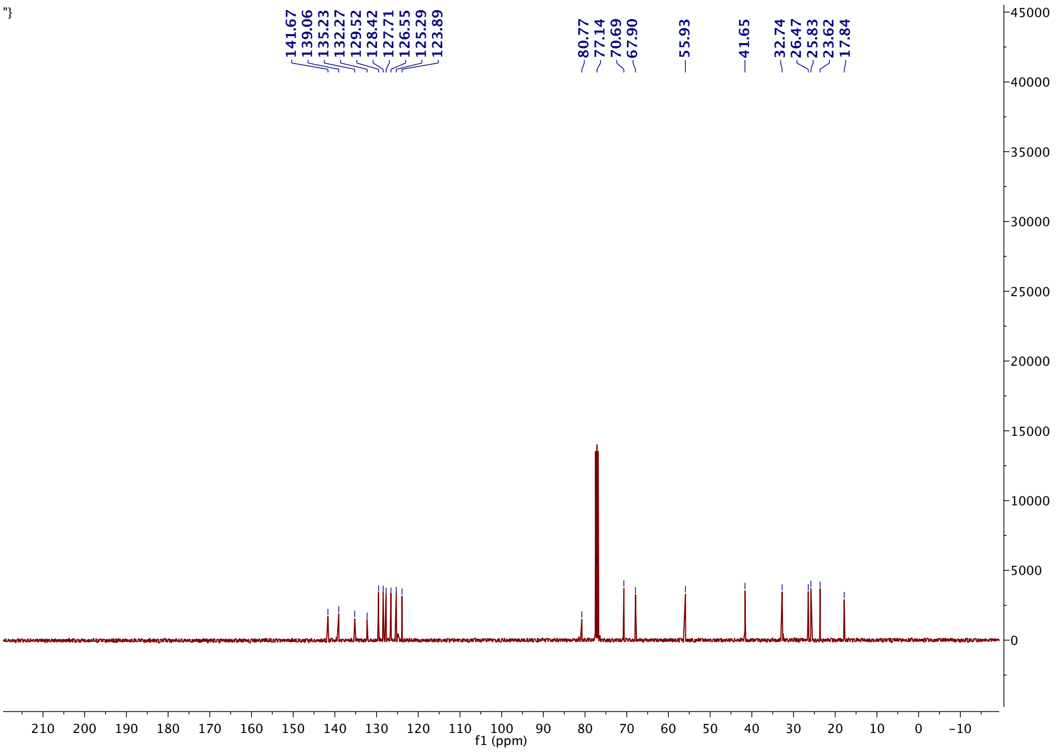

^{1}H-NMR and ^{13}C-NMR of **9b isomer B**

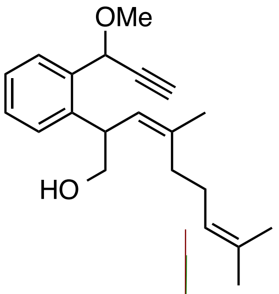

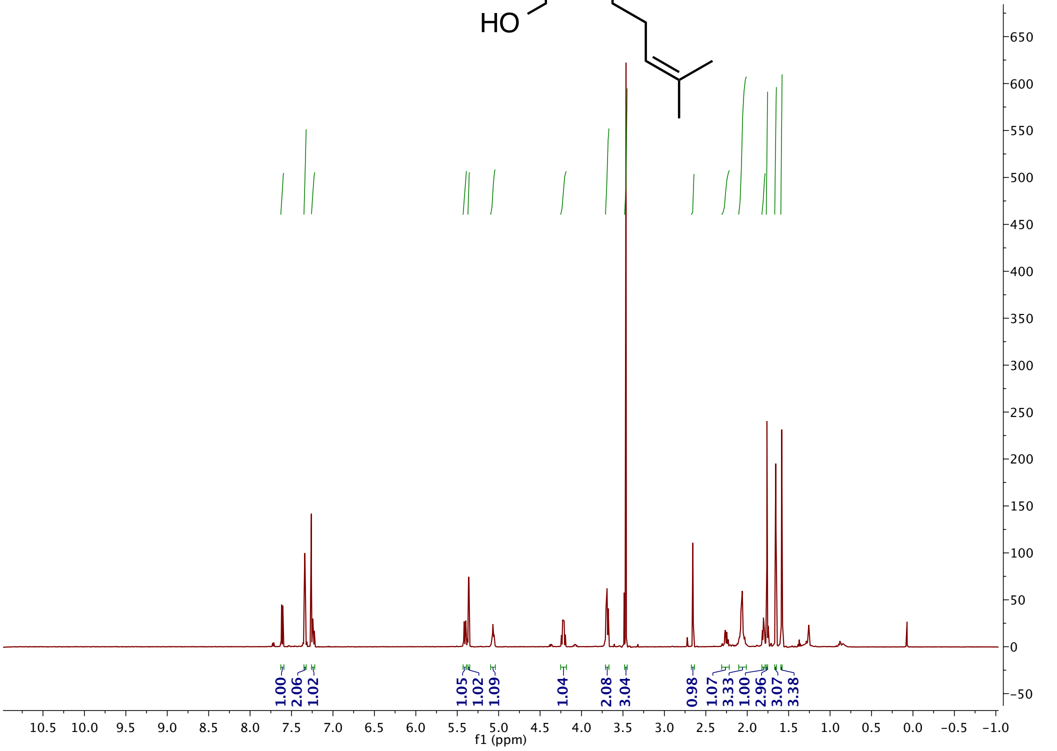

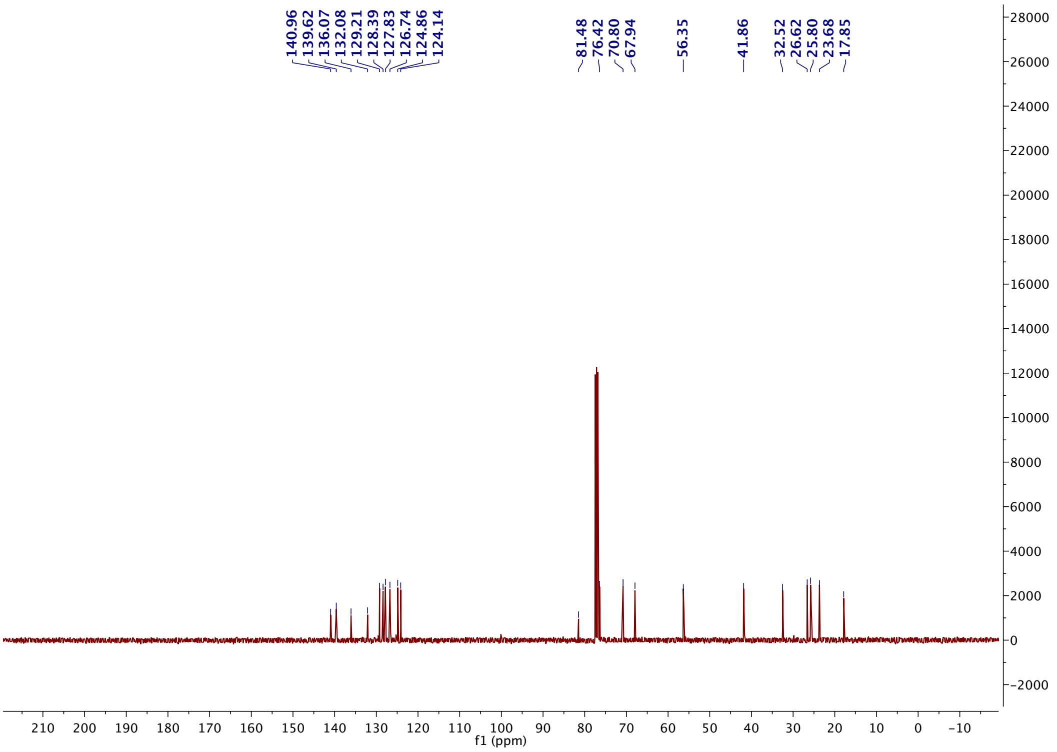

^{1}H-NMR and ^{13}C-NMR of **10a**

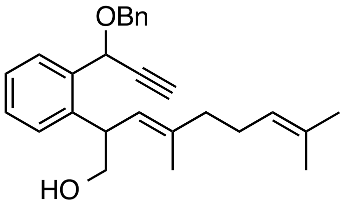

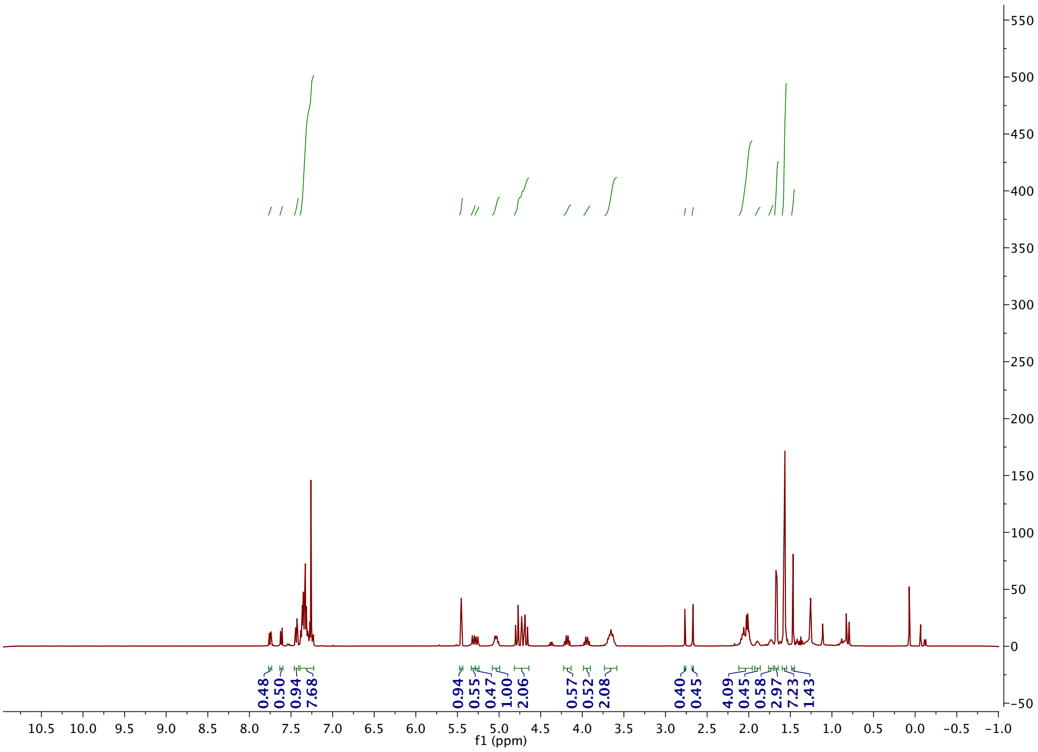

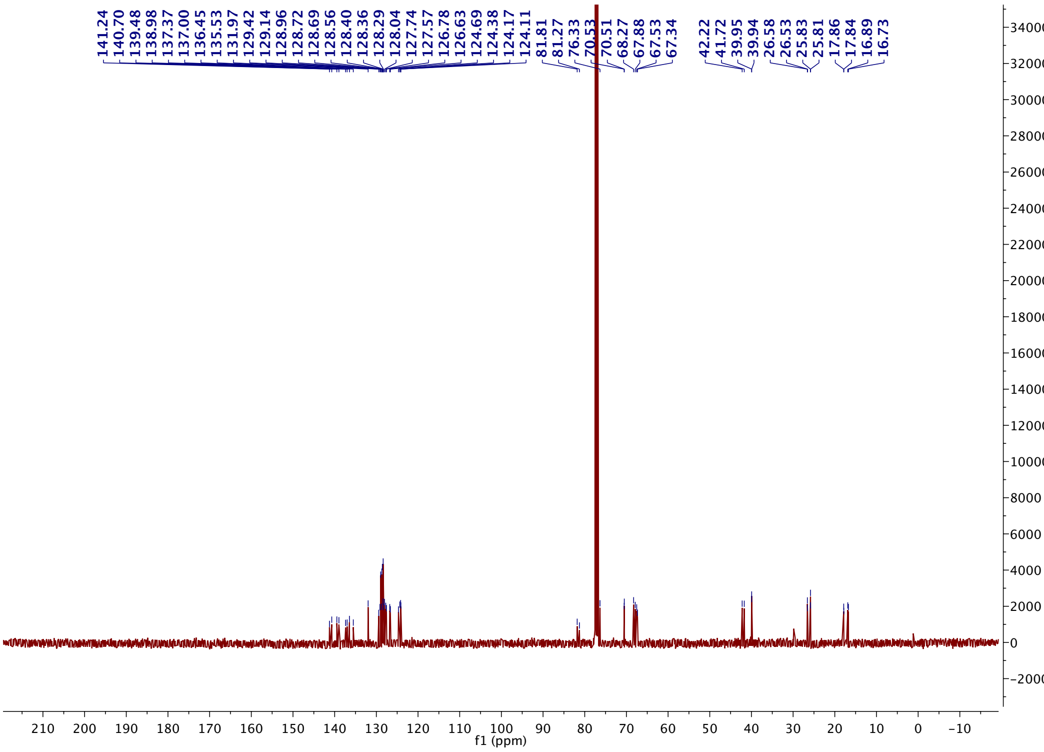

^{1}H-NMR and ^{13}C-NMR of **10b (mixture of diastereoisomers)**

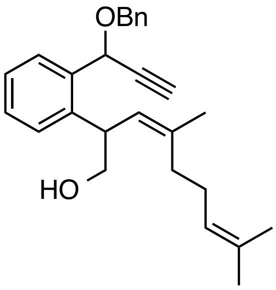

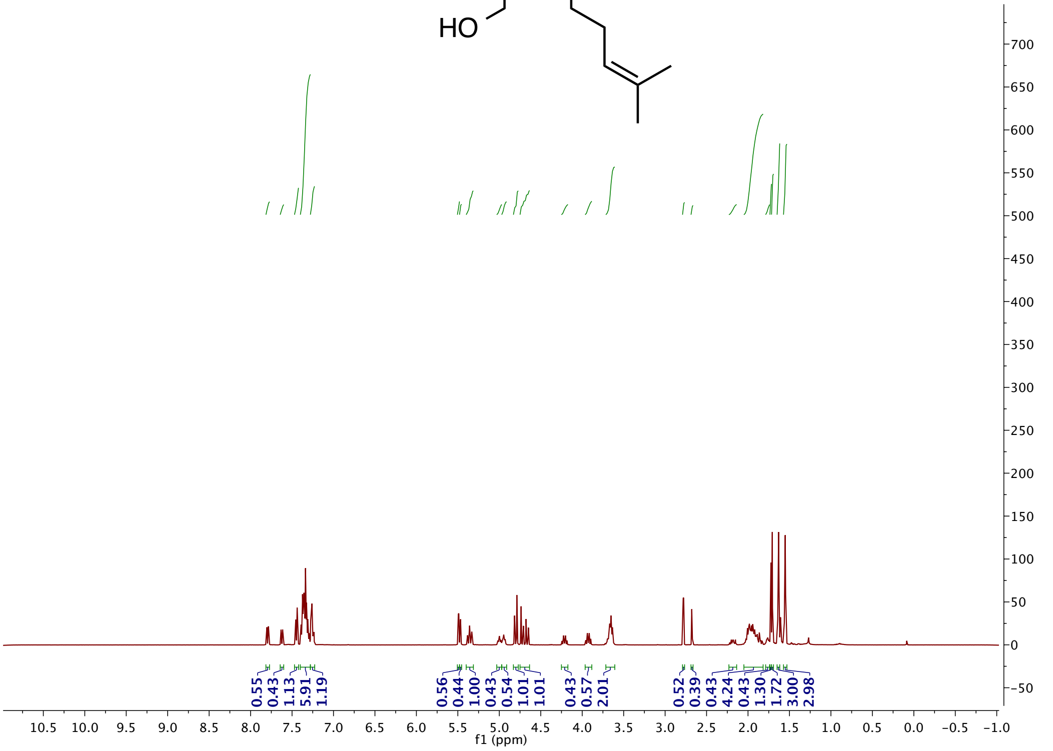

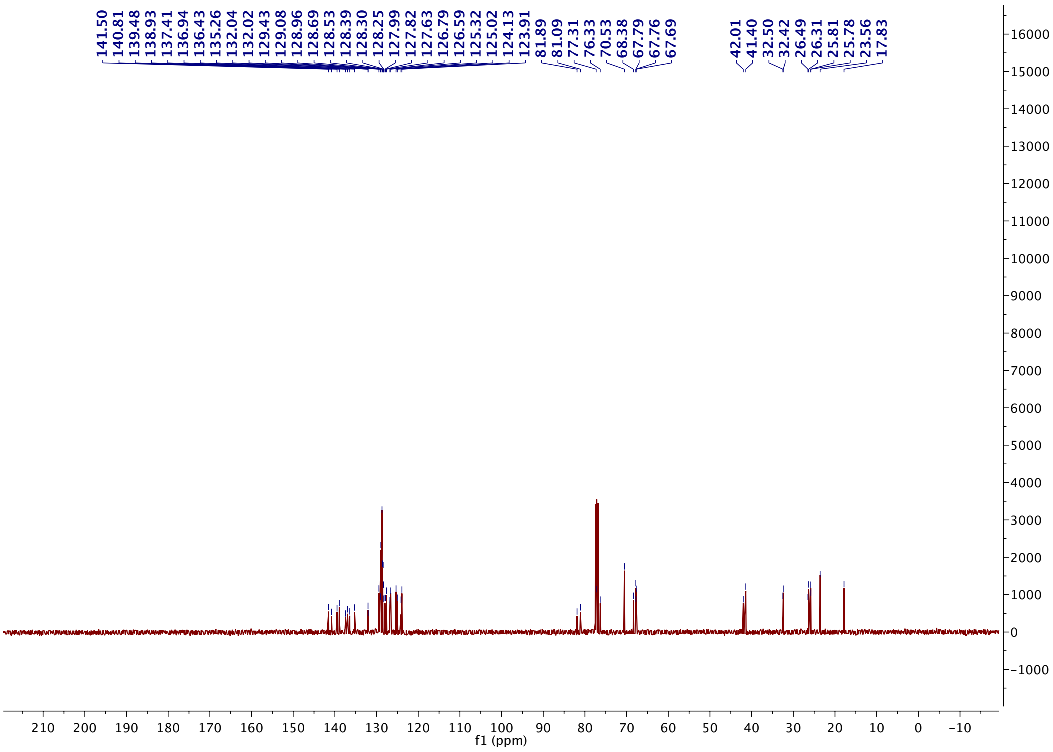

^{1}H-NMR and ^{13}C-NMR of **10b isomer 1**

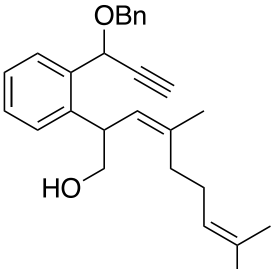

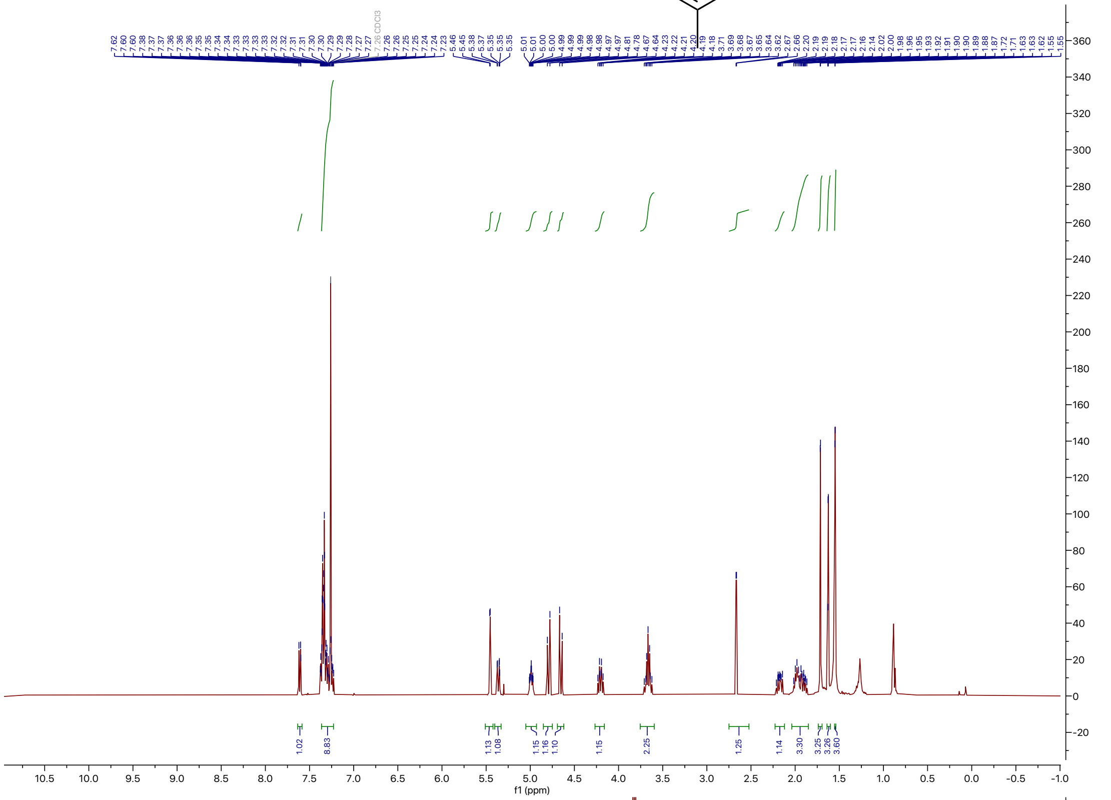

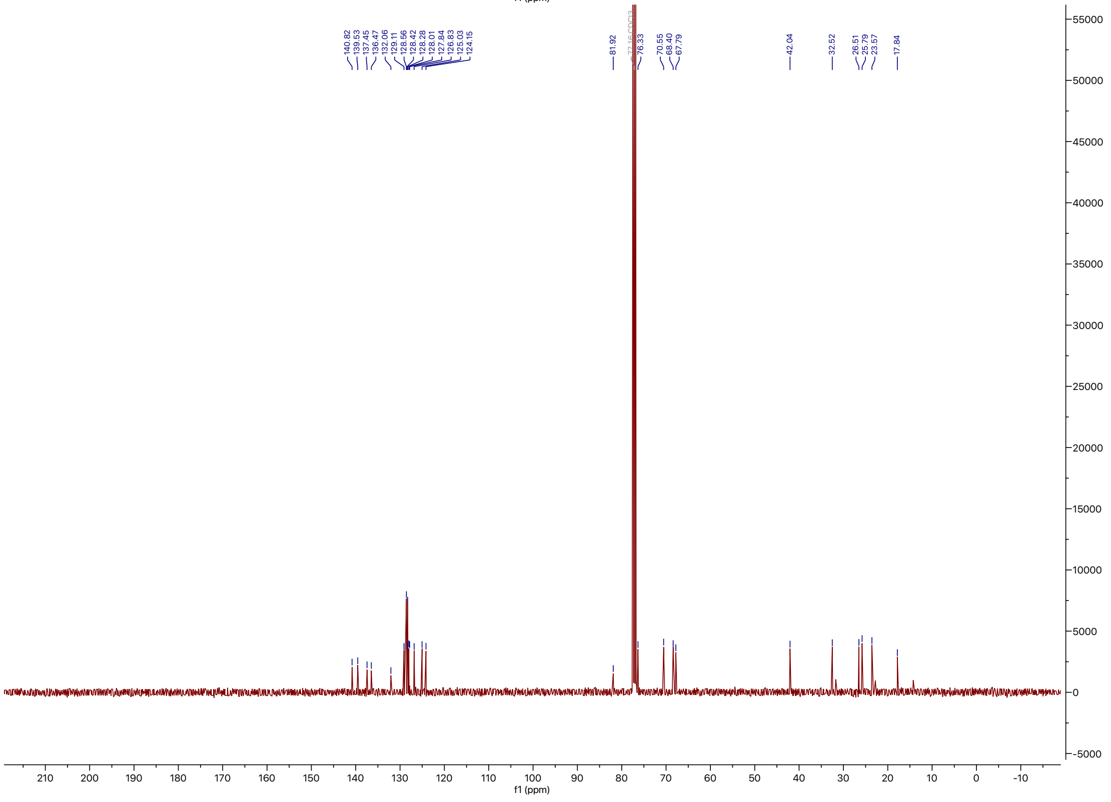

^{1}H-NMR and ^{13}C-NMR of **10b isomer 2**

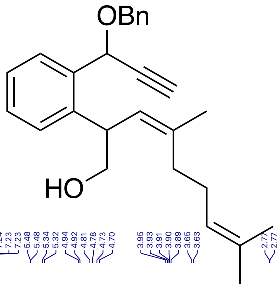

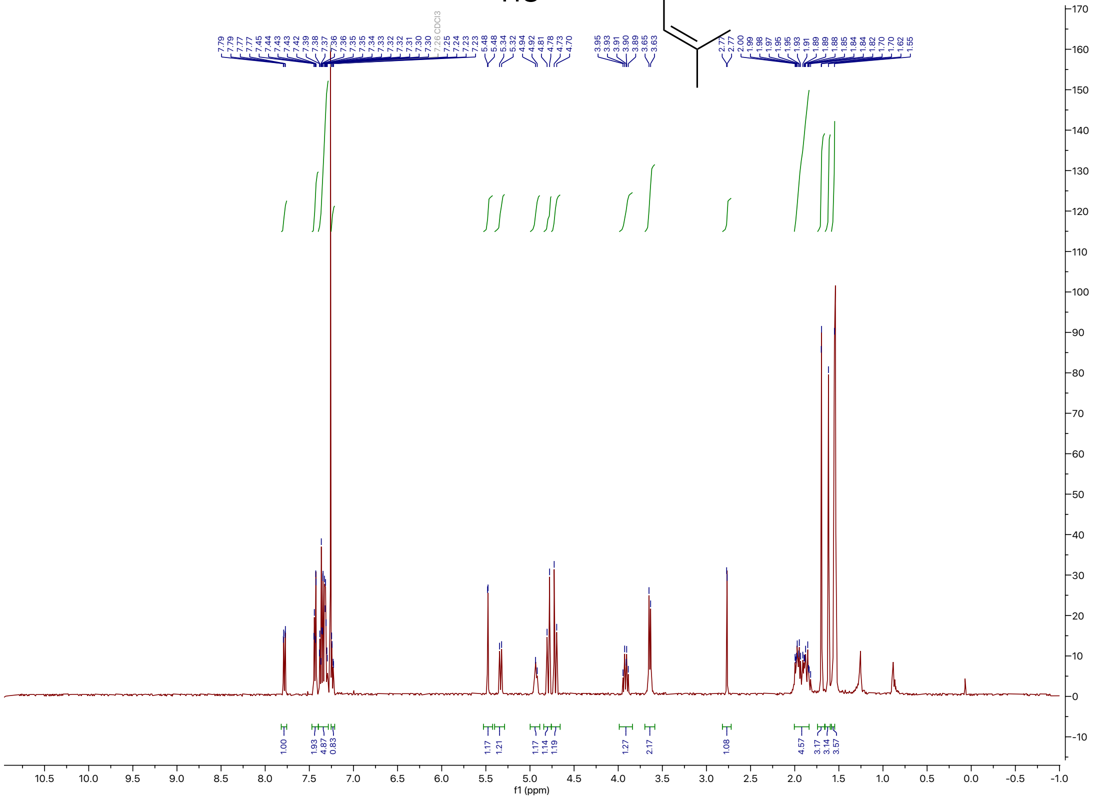

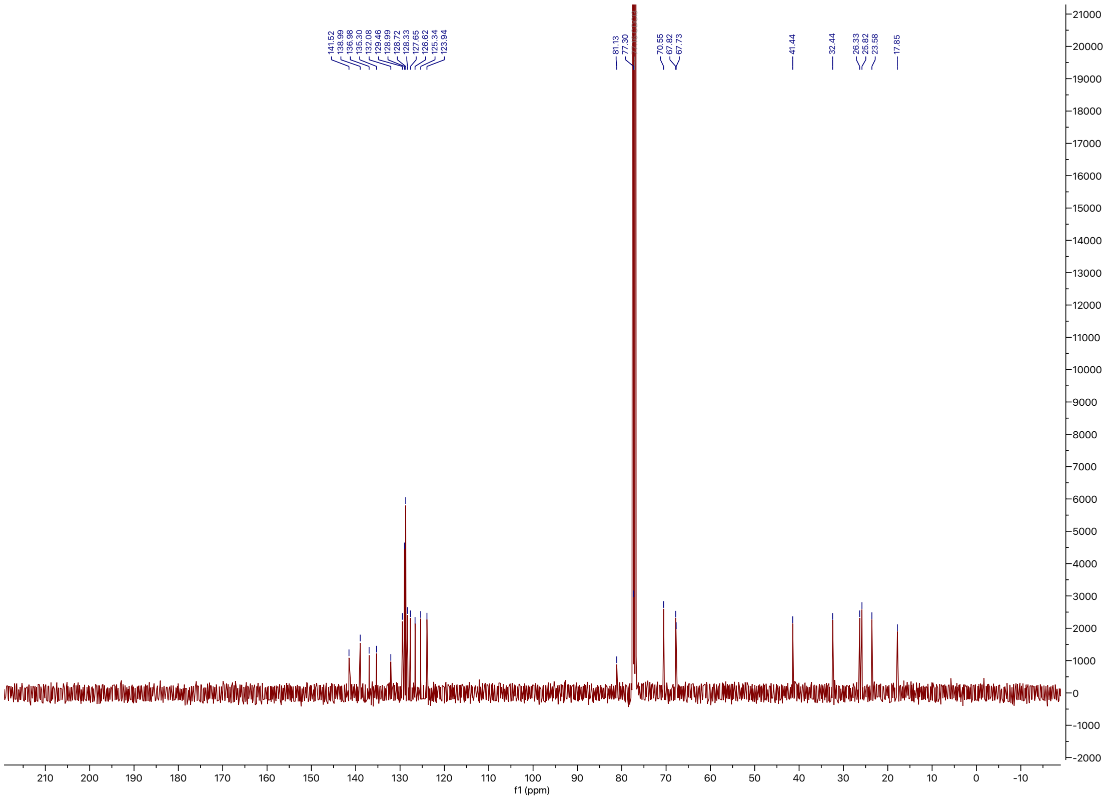

^{1}H-NMR and ^{13}C-NMR of **4a**

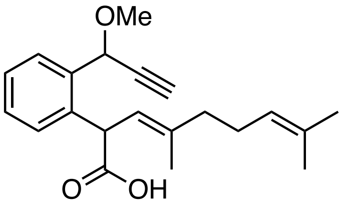

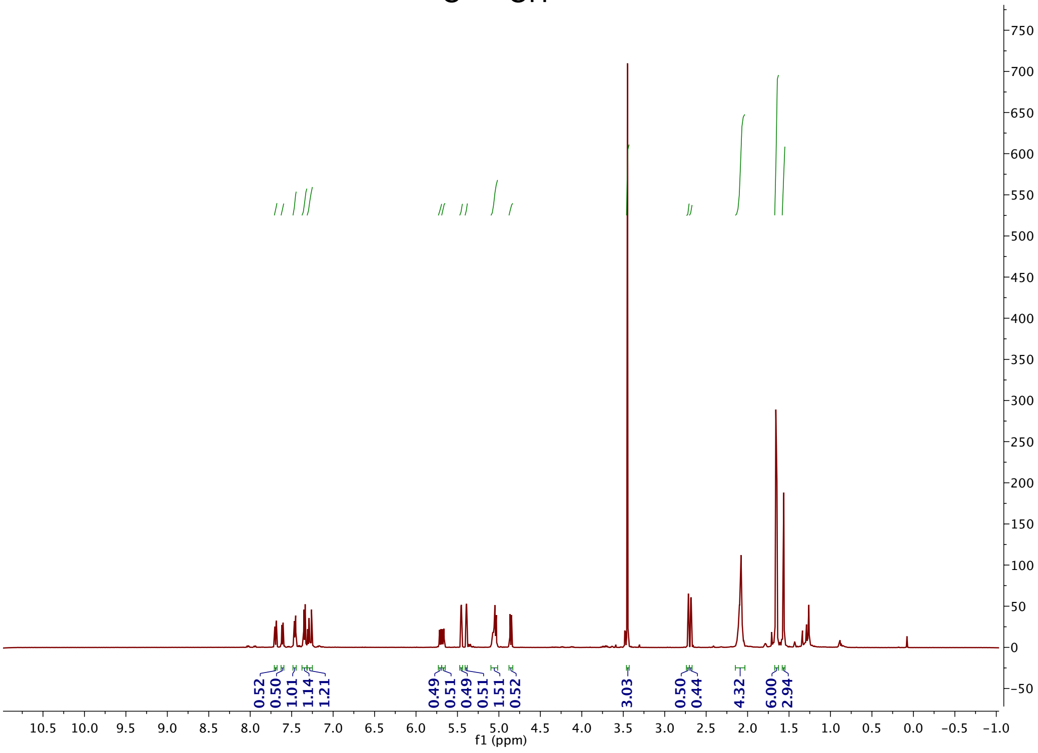

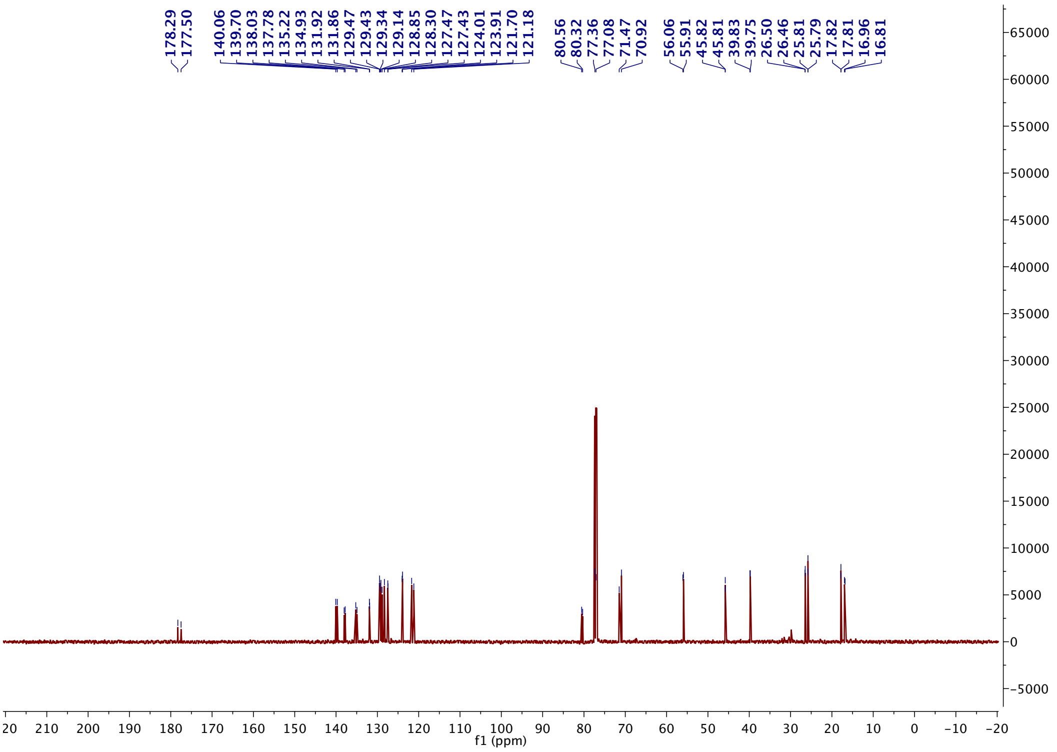

^{1}H-NMR and ^{13}C-NMR of **S10a**

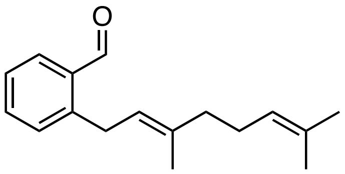

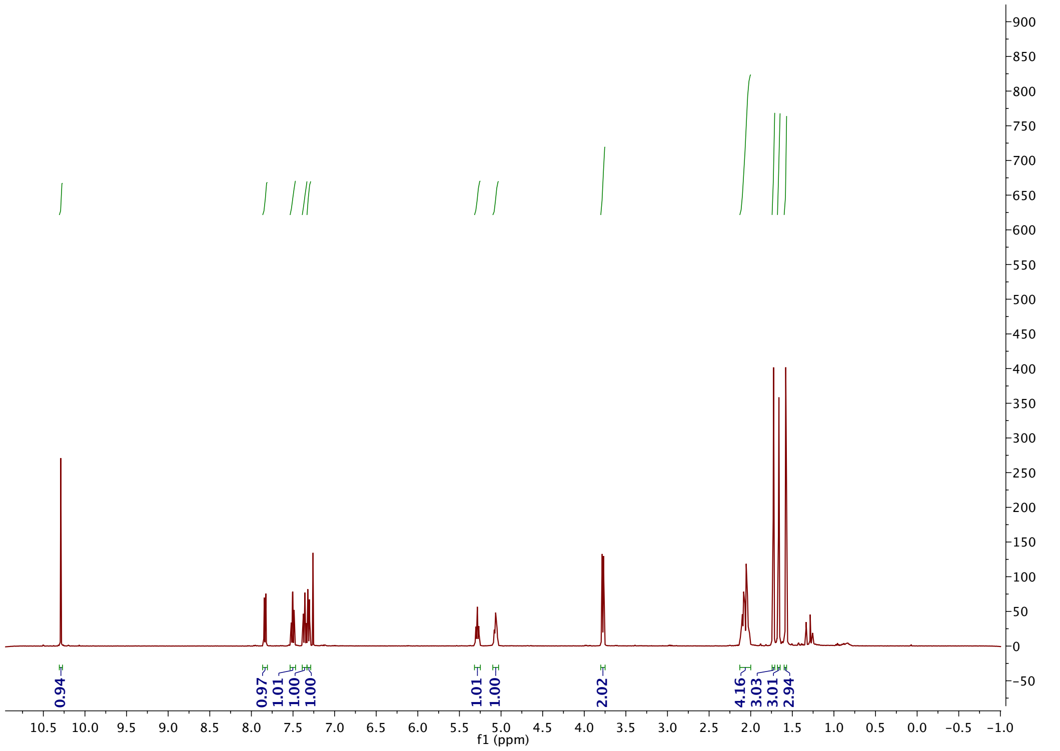

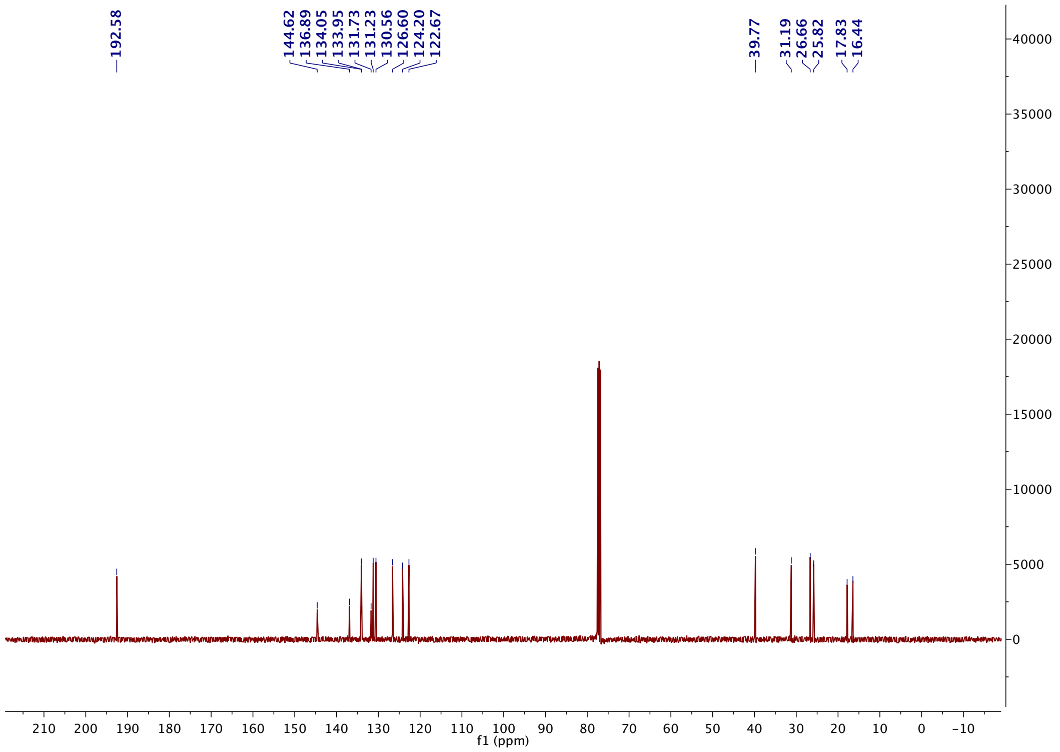

^{1}H-NMR and ^{13}C-NMR of **S10b**

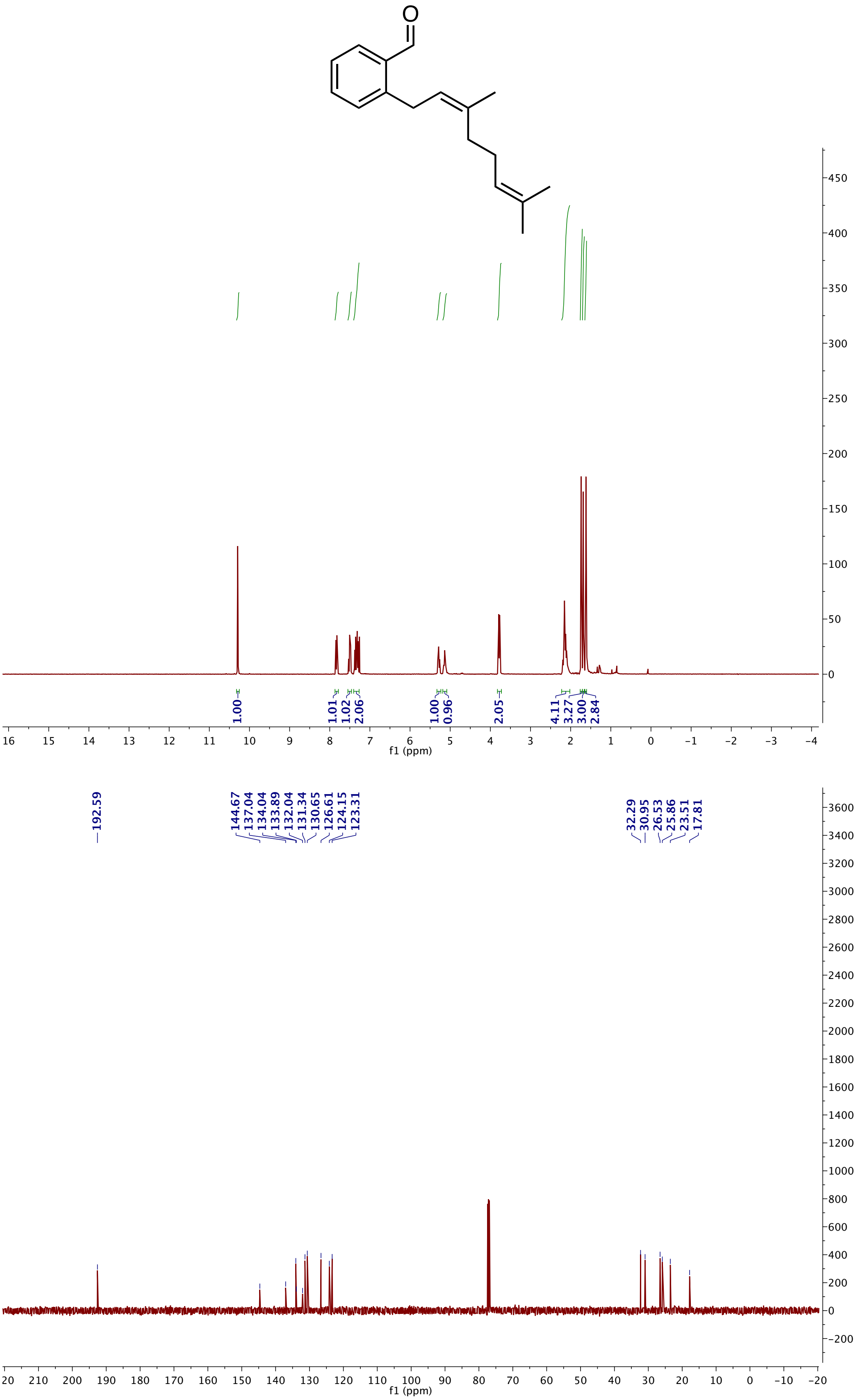

^{1}H-NMR and ^{13}C-NMR of **S11a**

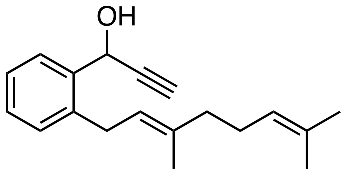

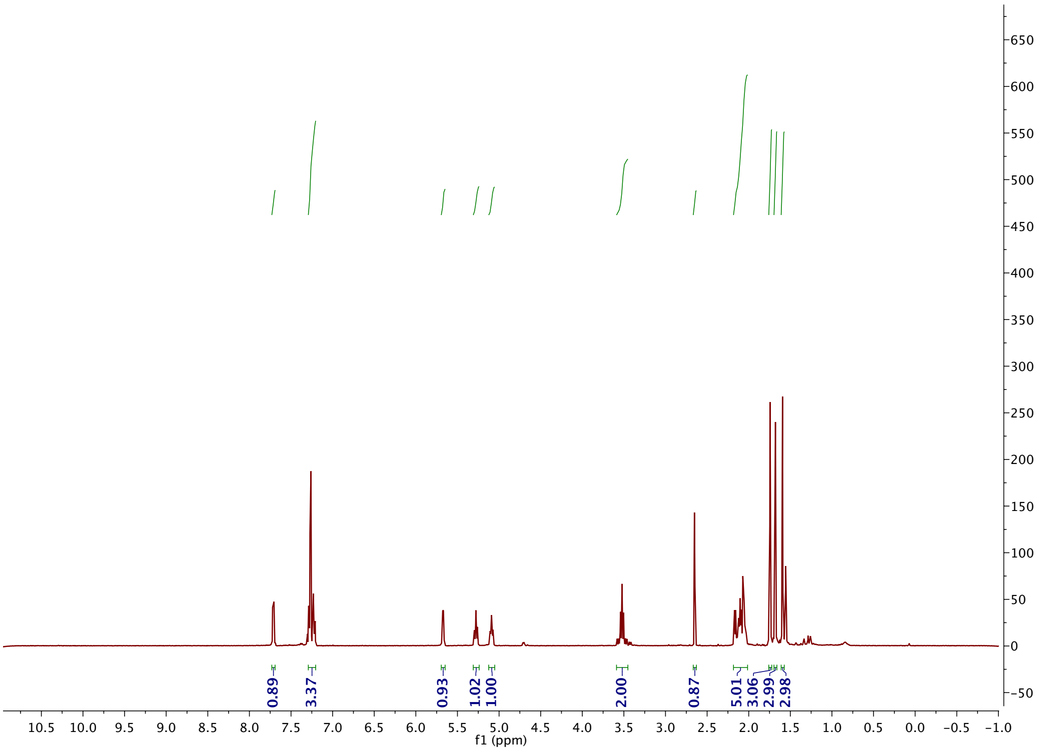

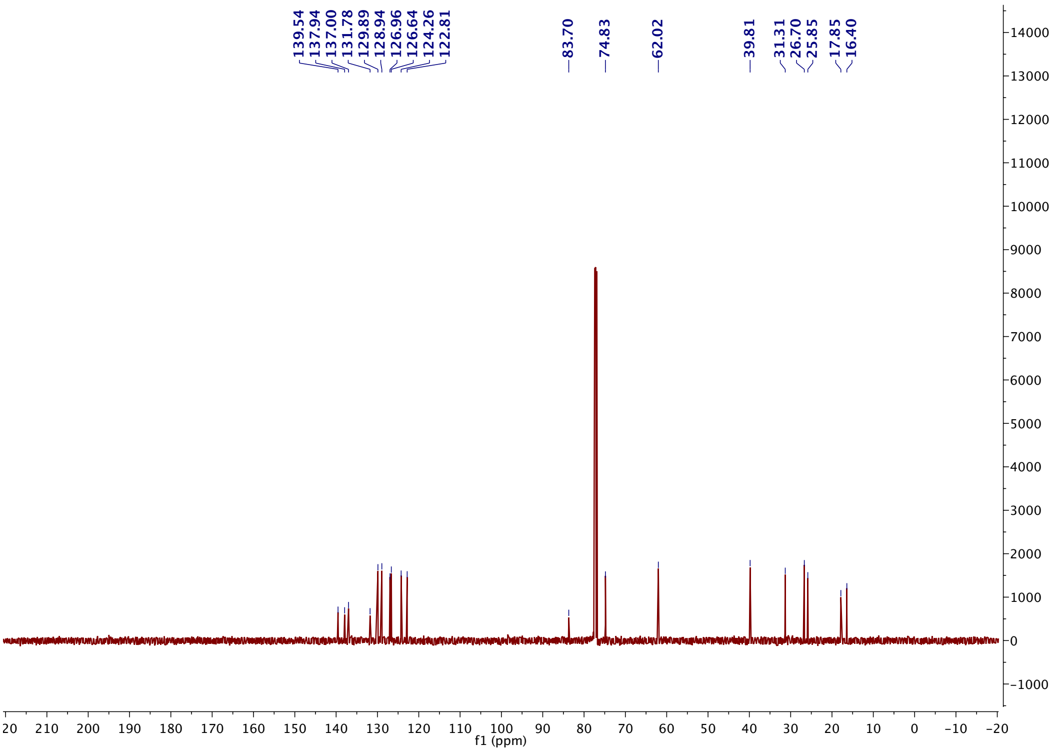

^{1}H-NMR and ^{13}C-NMR of **S11b**

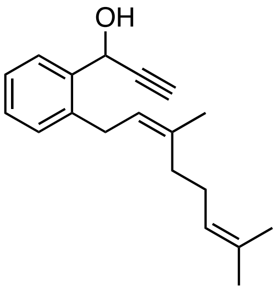

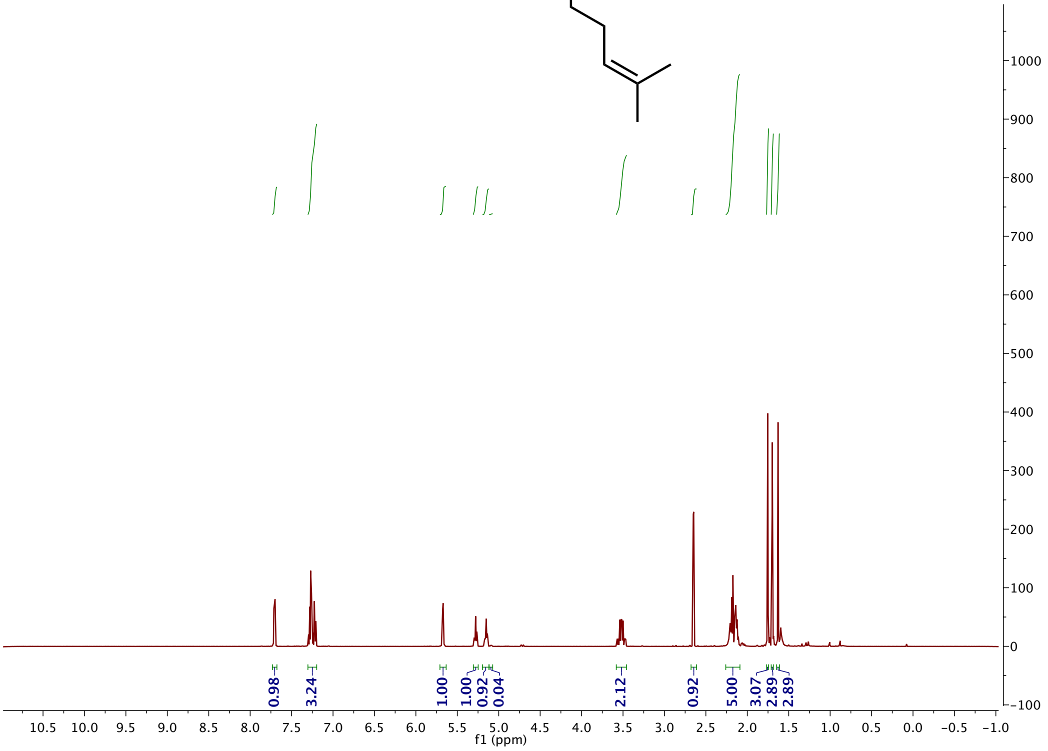

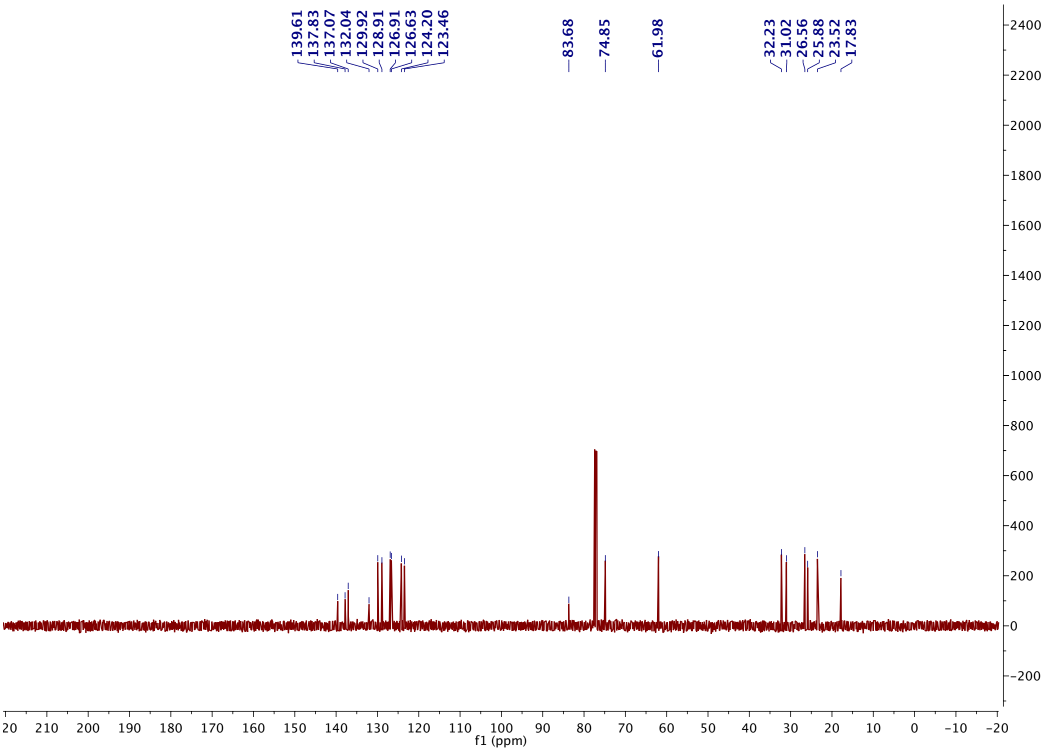

^{1}H-NMR and ^{13}C-NMR of **15a**

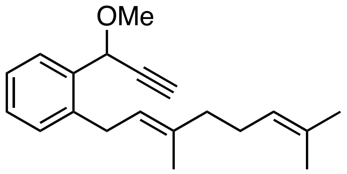

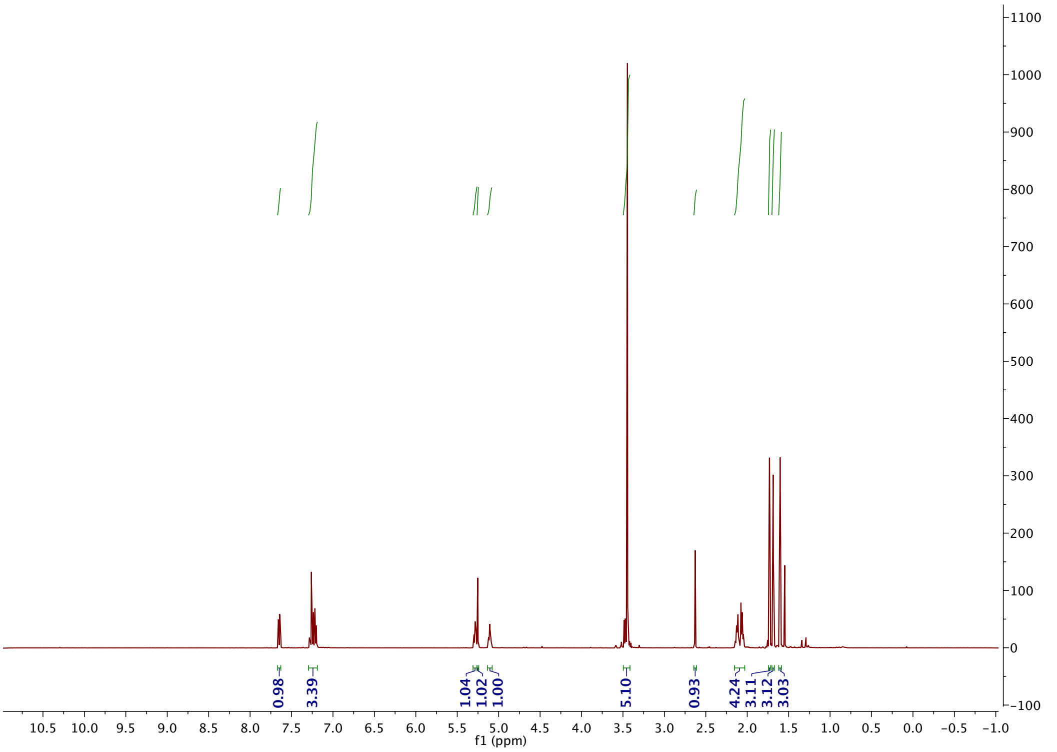

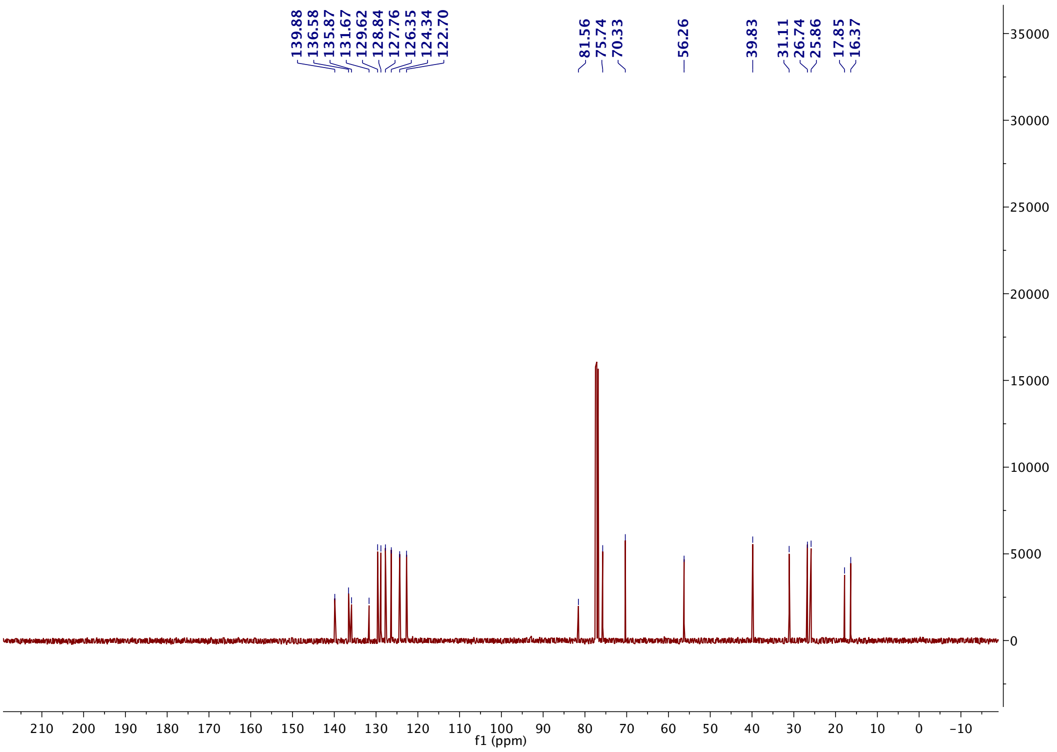

^{1}H-NMR and ^{13}C-NMR of **15b**

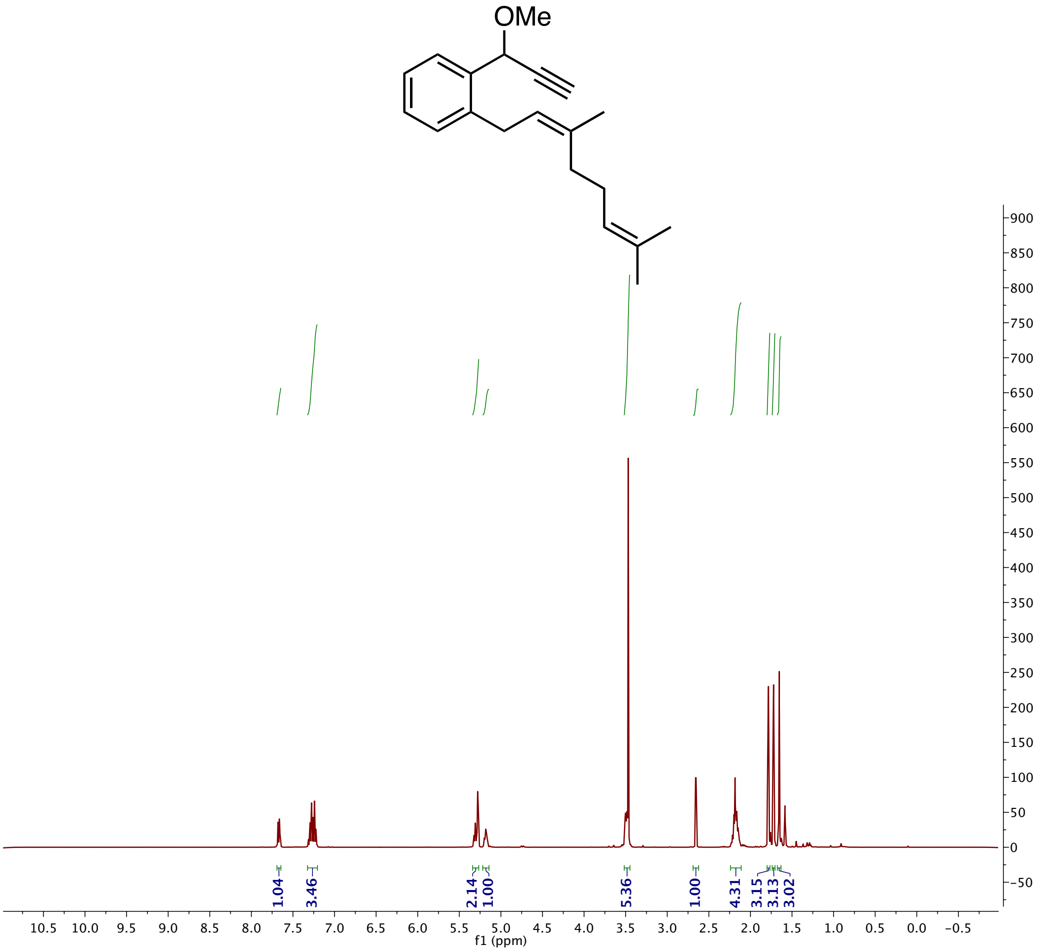

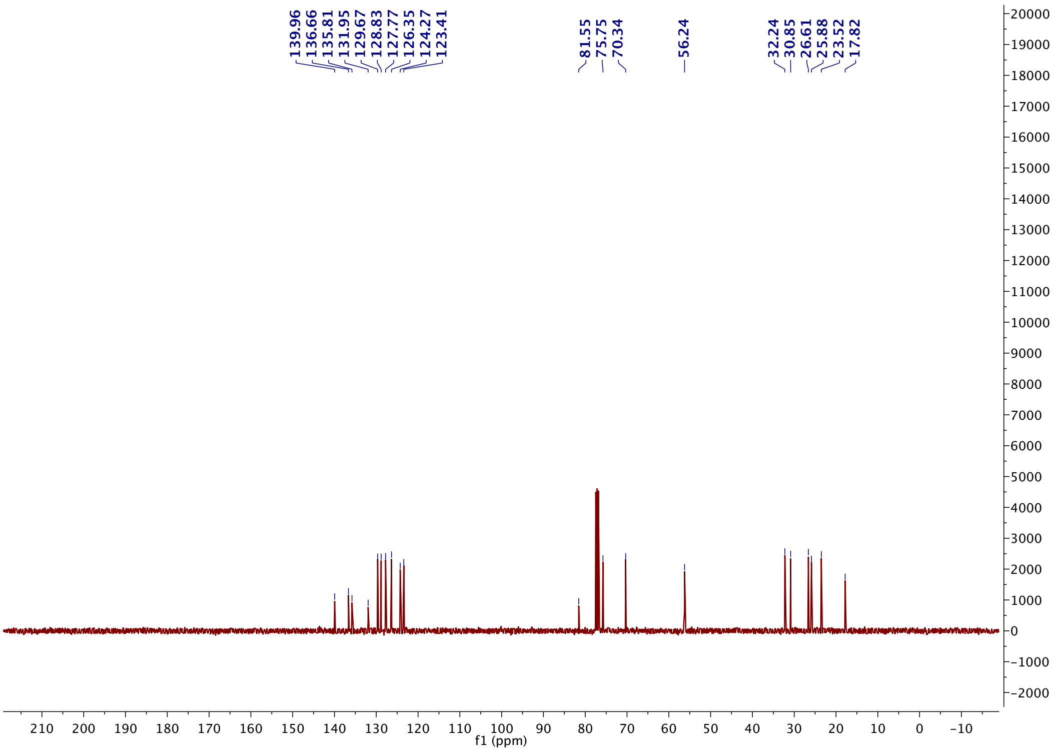

^{1}H-NMR and ^{13}C-NMR of **S12**

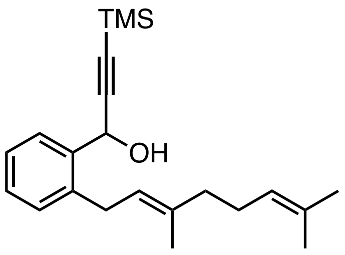

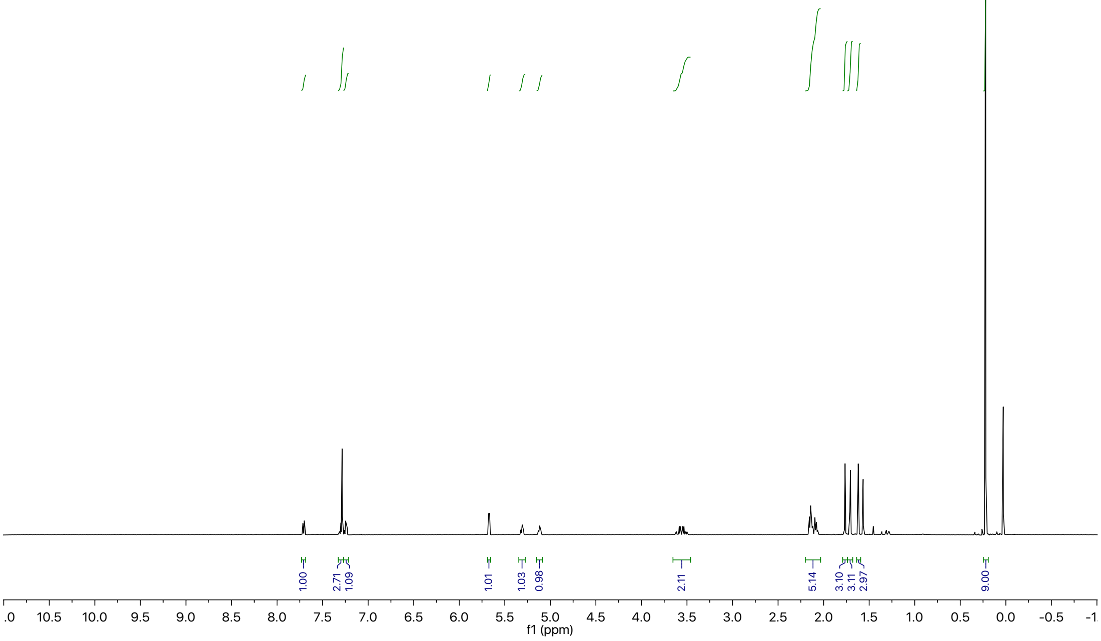

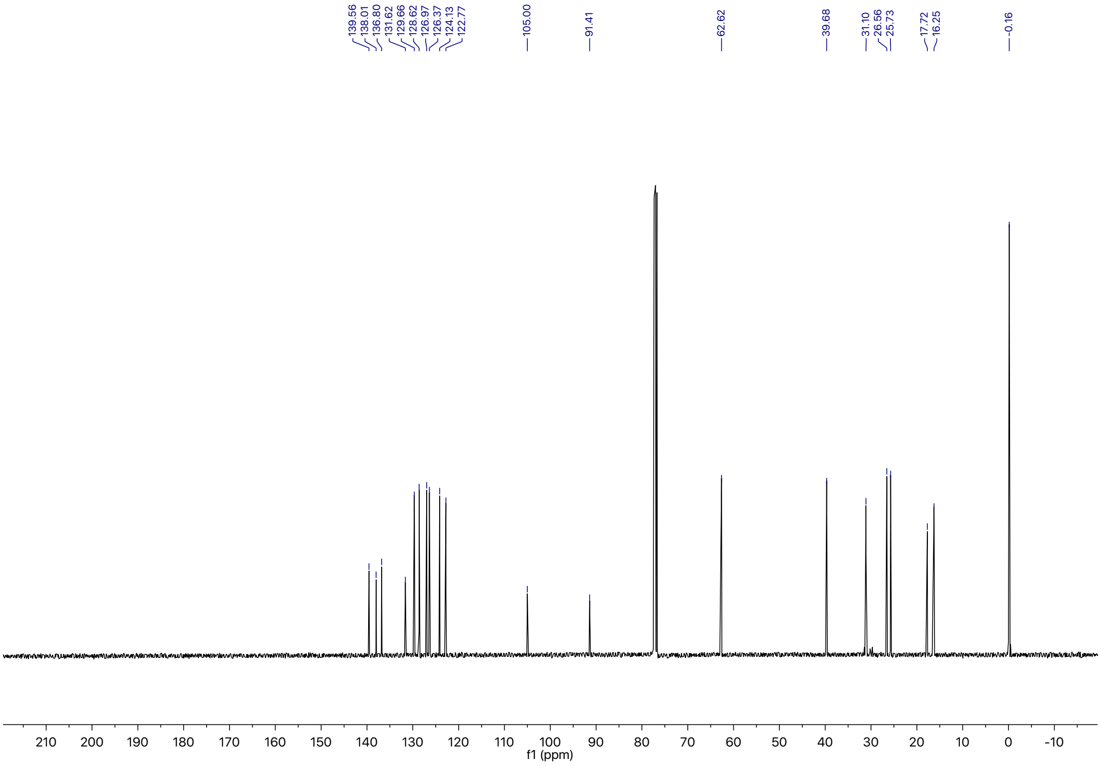

^{1}H-NMR and ^{13}C-NMR of **S13**

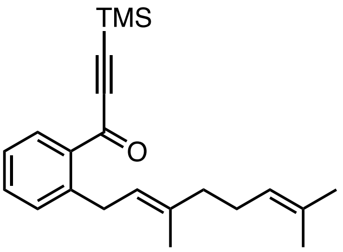

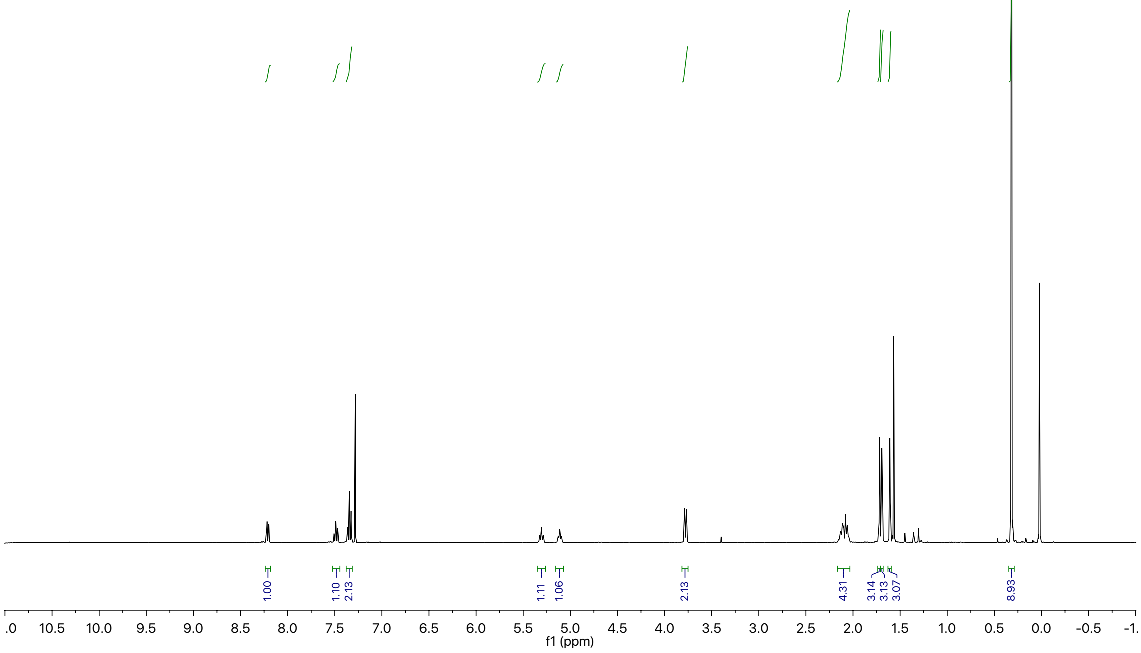

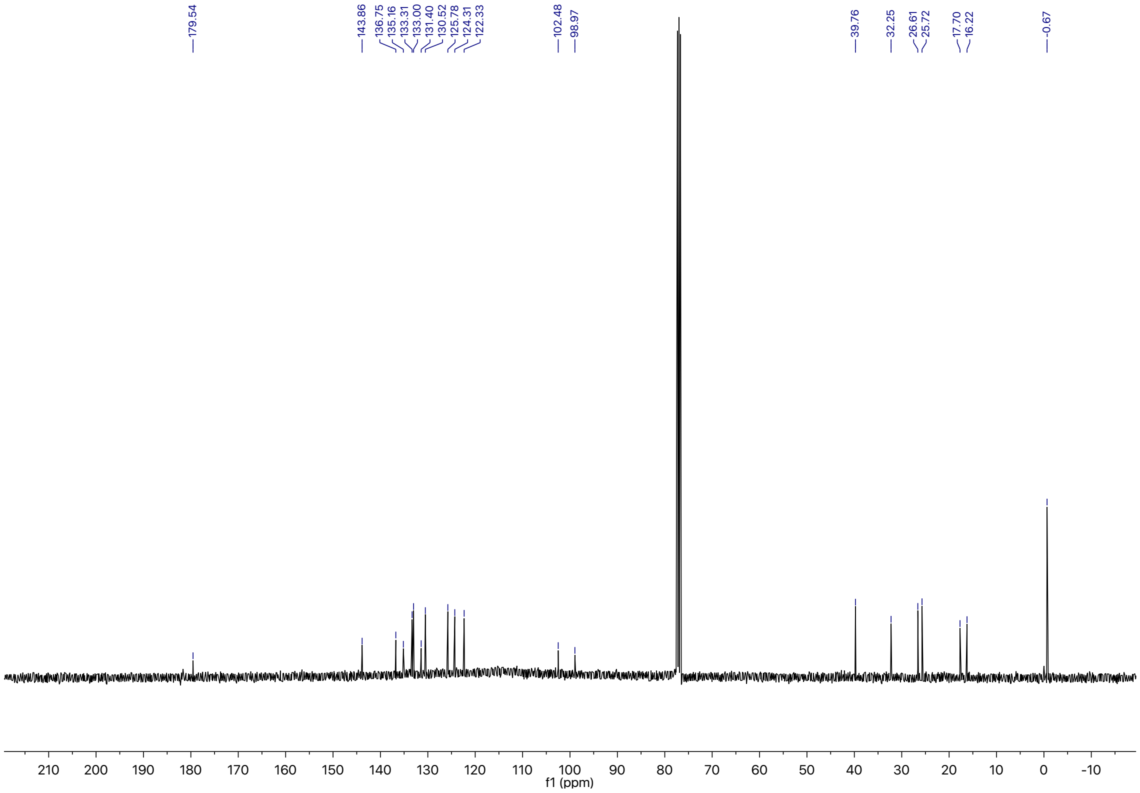

^{1}H-NMR and ^{13}C-NMR of **S14**

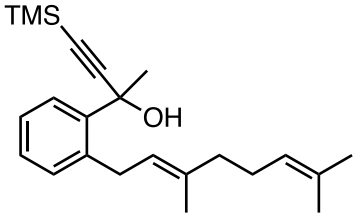

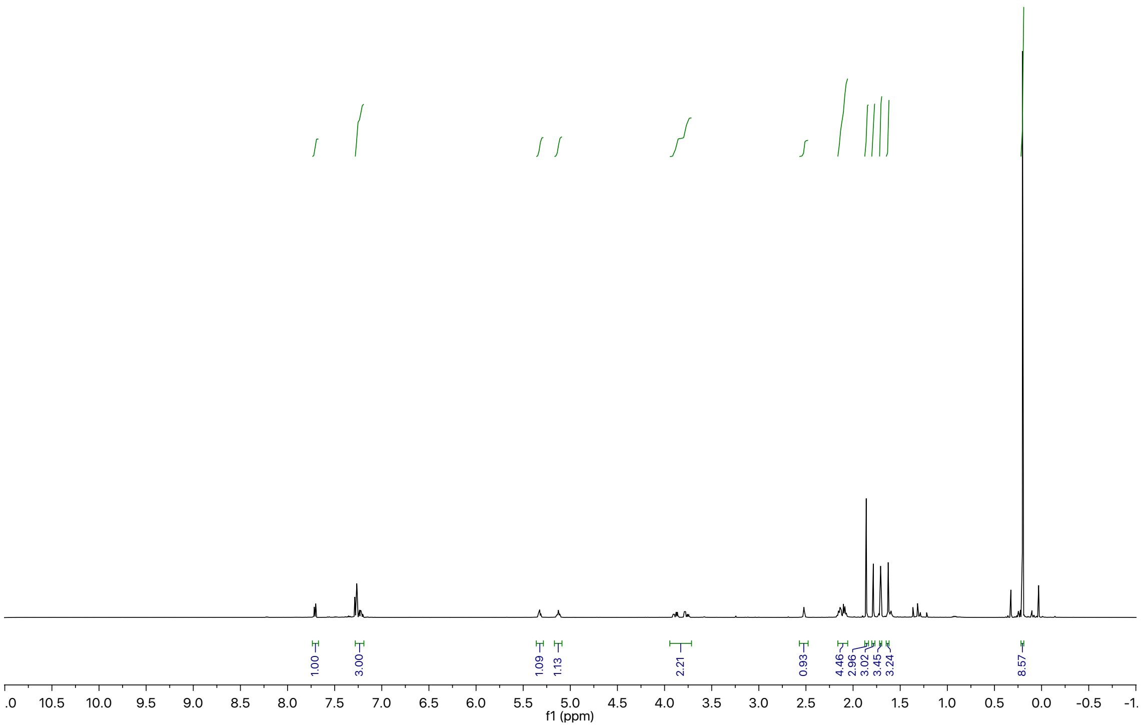

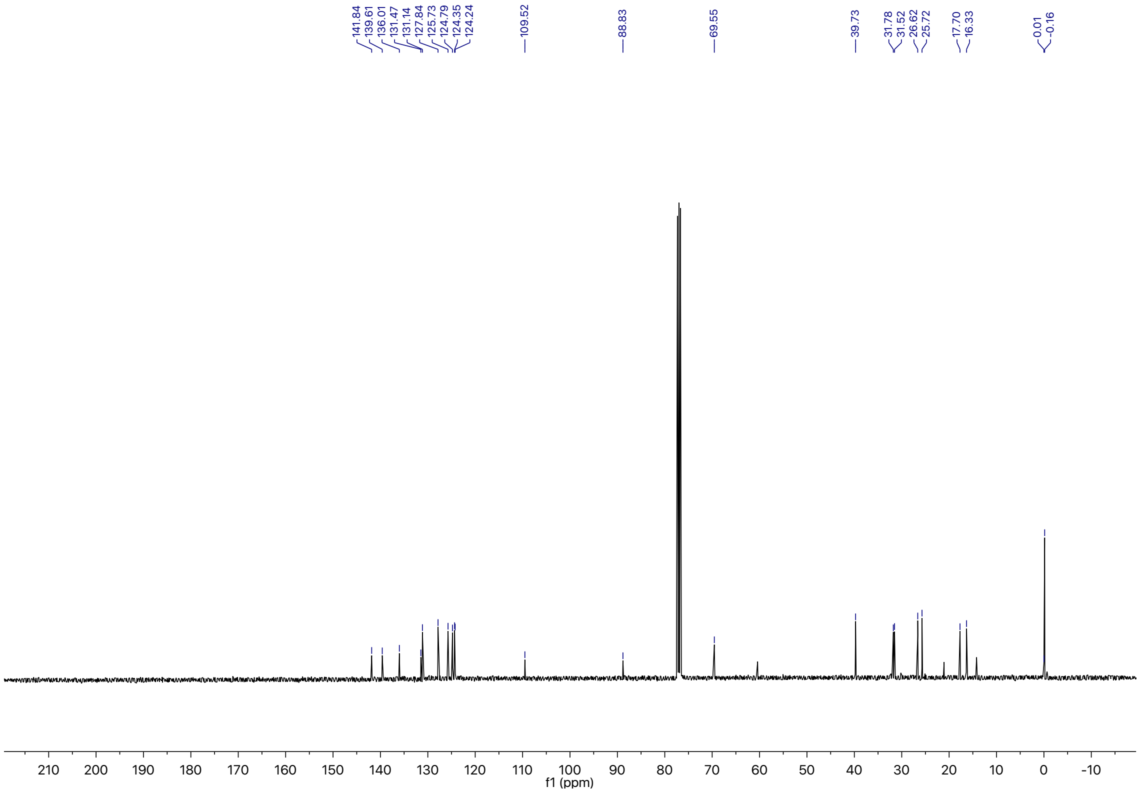

^{1}H-NMR and ^{13}C-NMR of **S15**

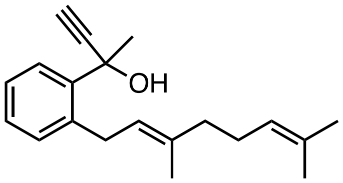

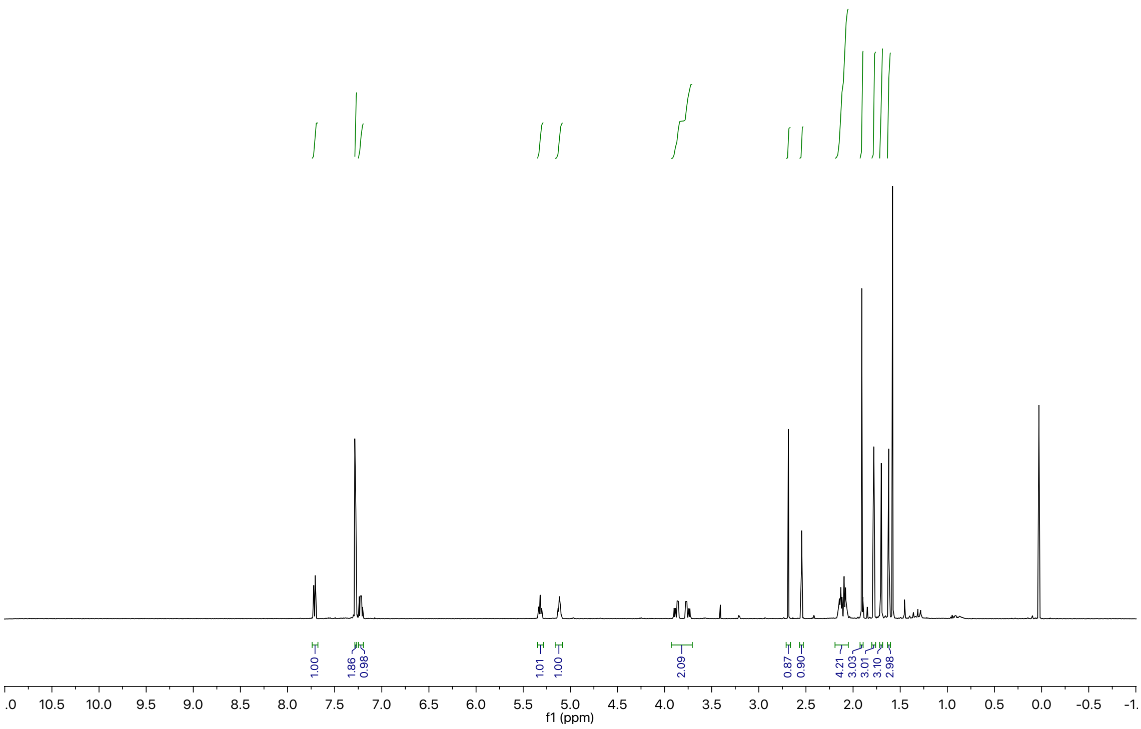

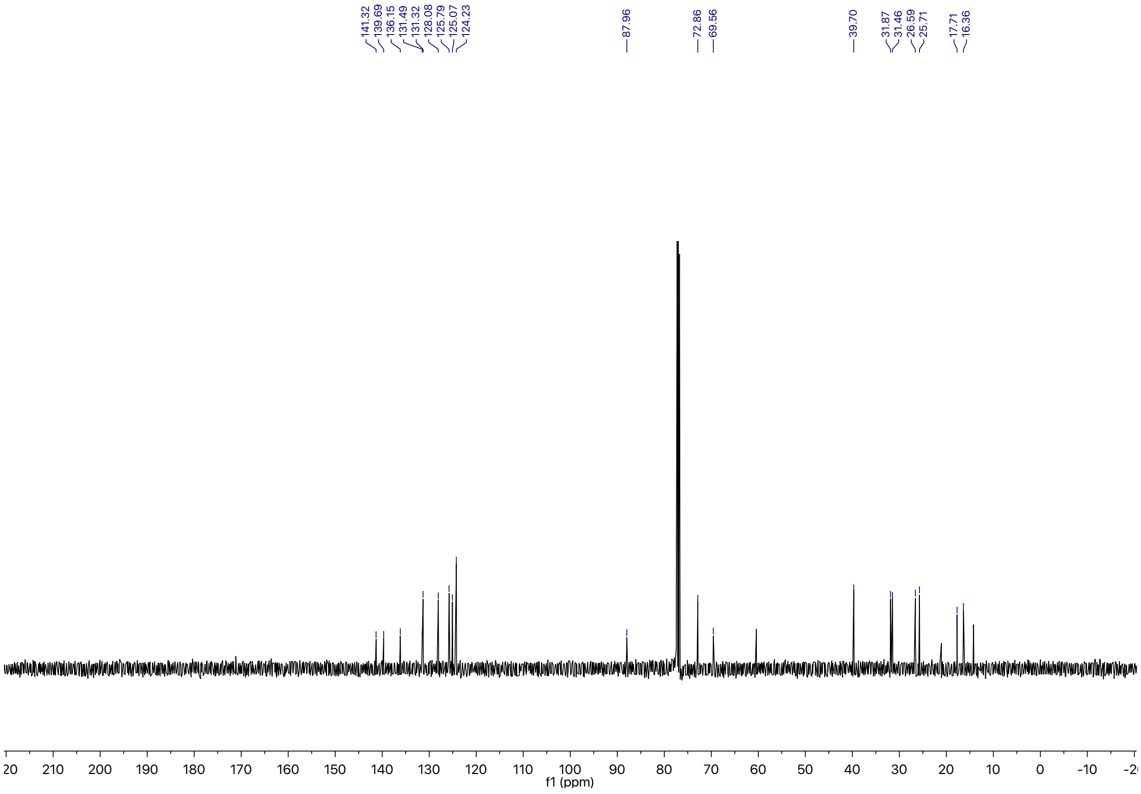

^{1}H-NMR and ^{13}C-NMR of **16**

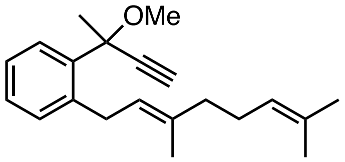

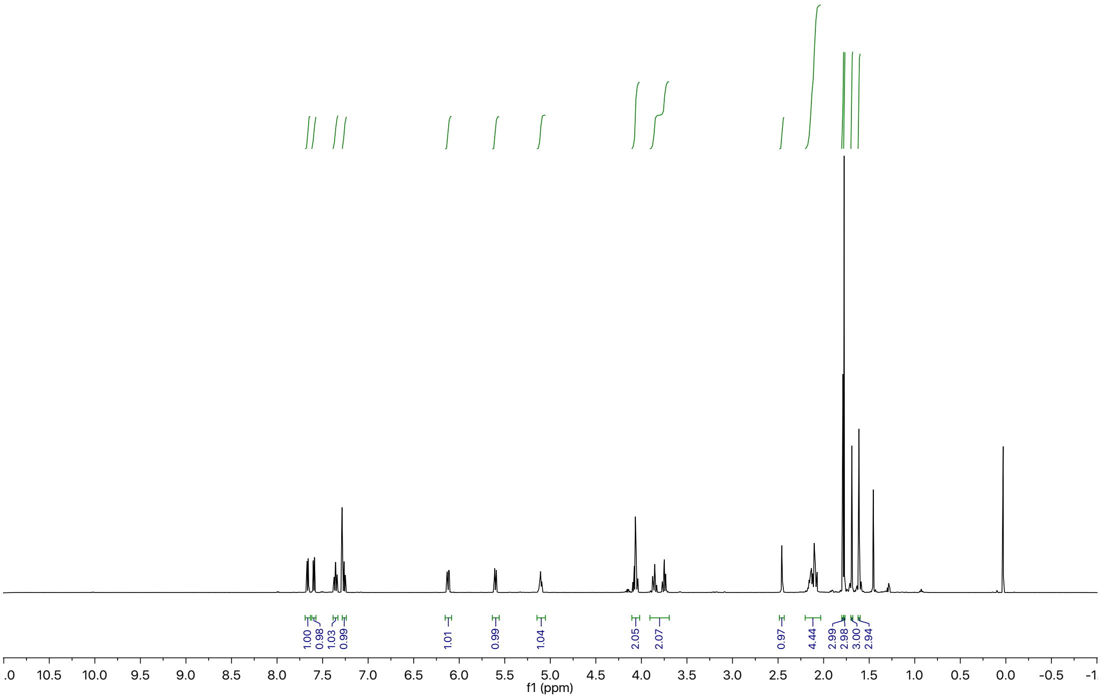

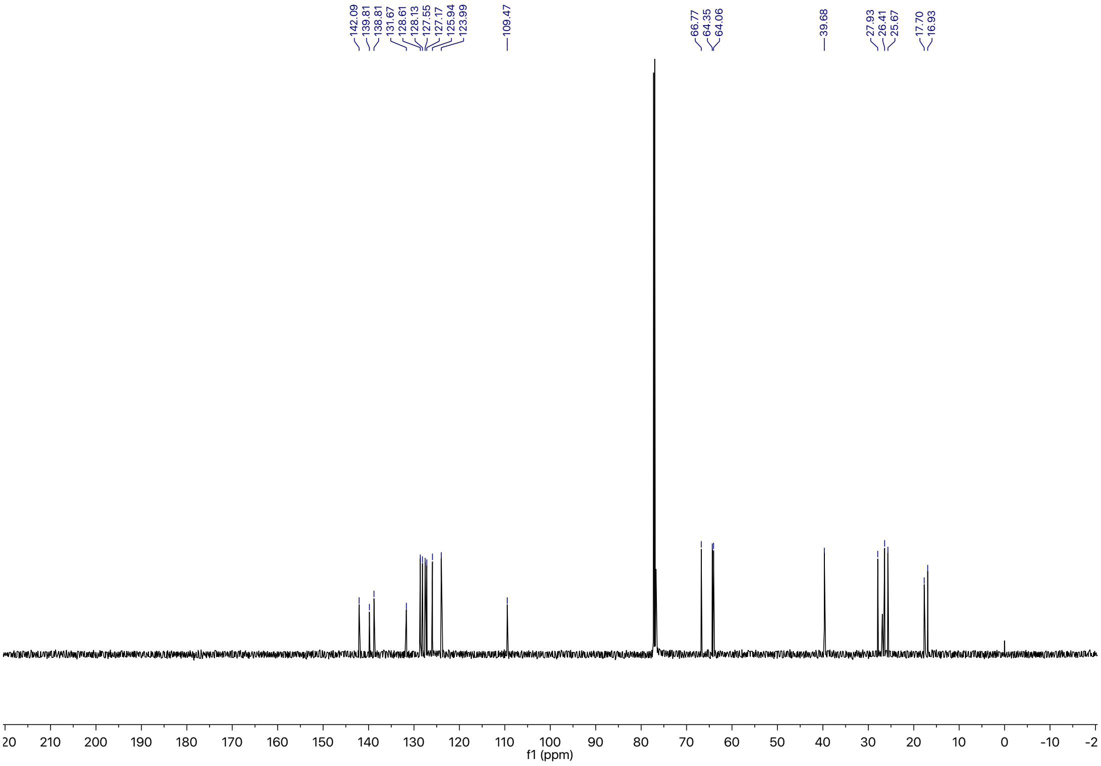

^{1}H-NMR and ^{13}C-NMR of **11**

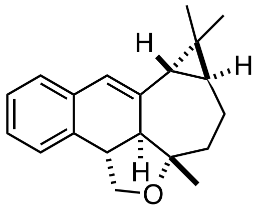

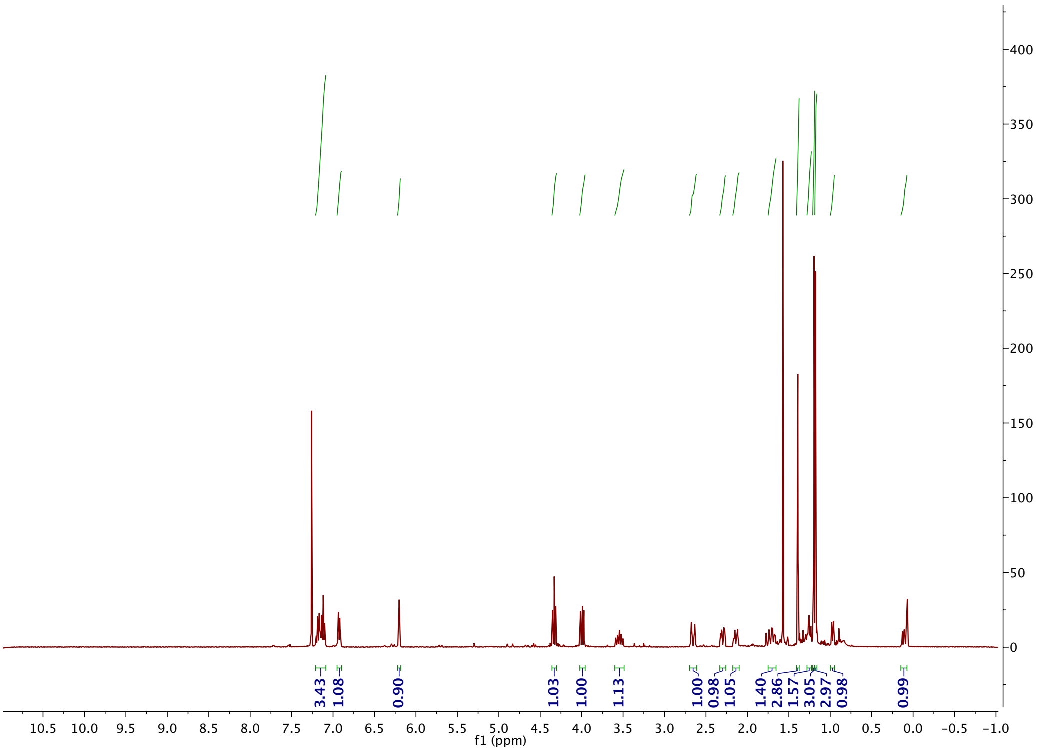

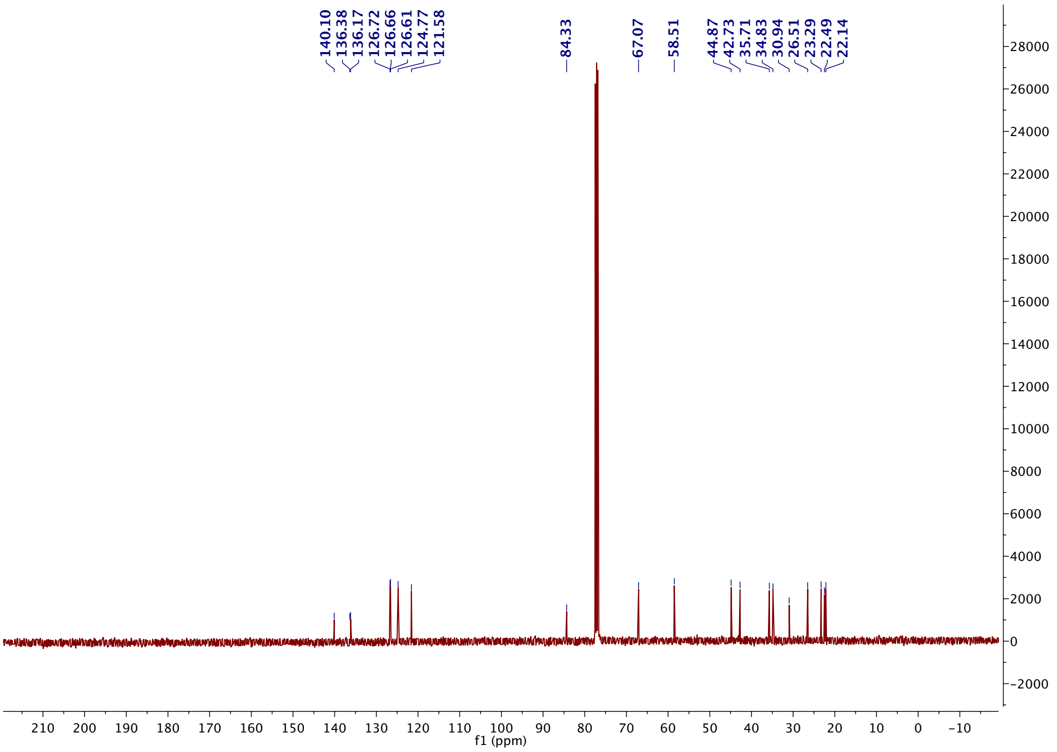

^{1}H-NMR and ^{13}C-NMR of **5a**

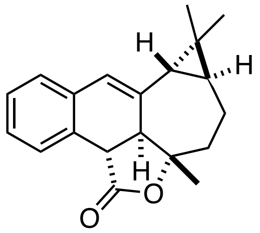

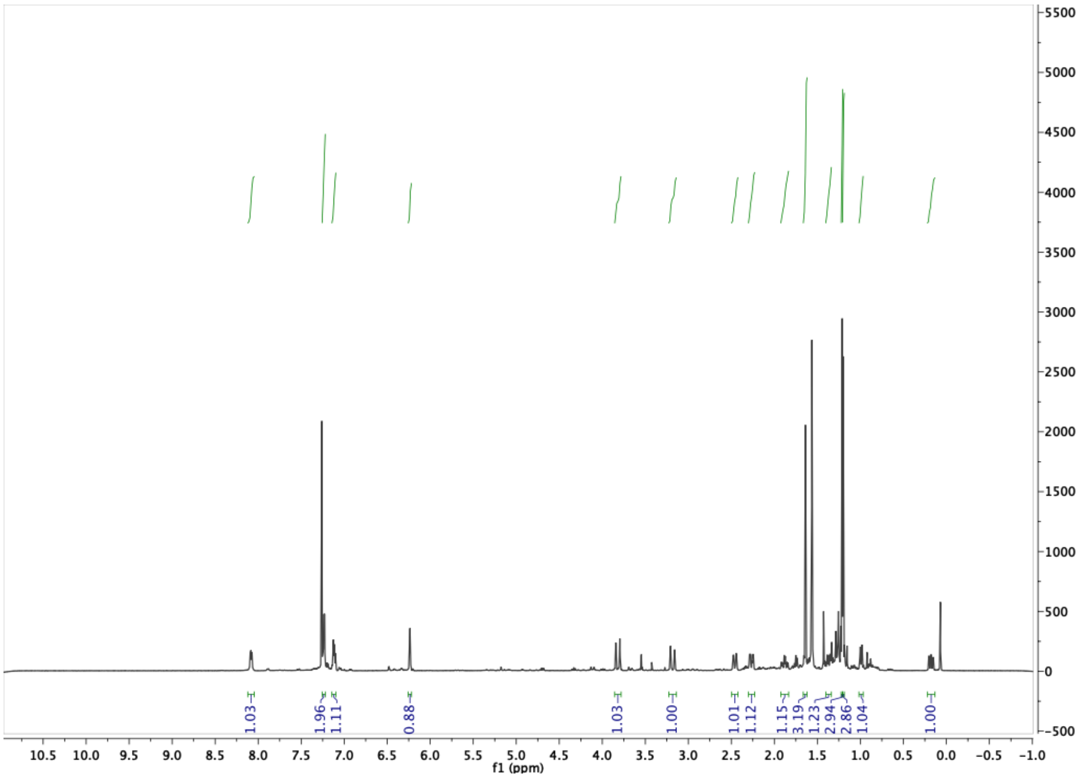

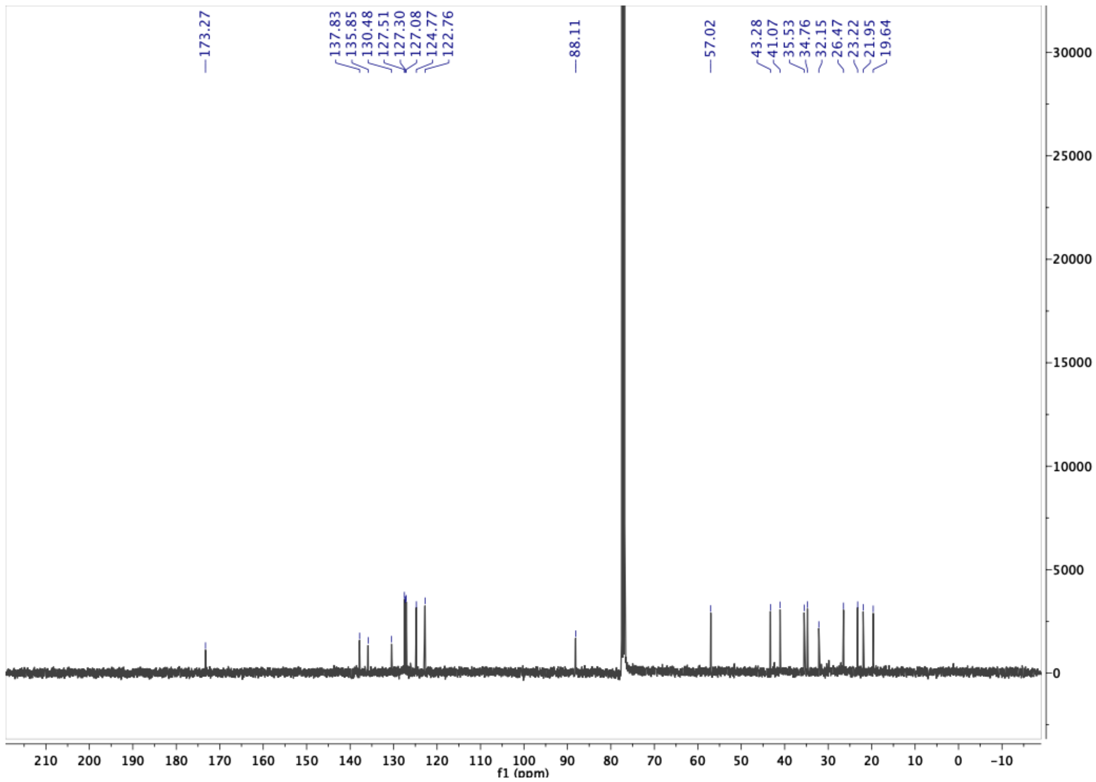

COSY ^1H-^1H of **5a**

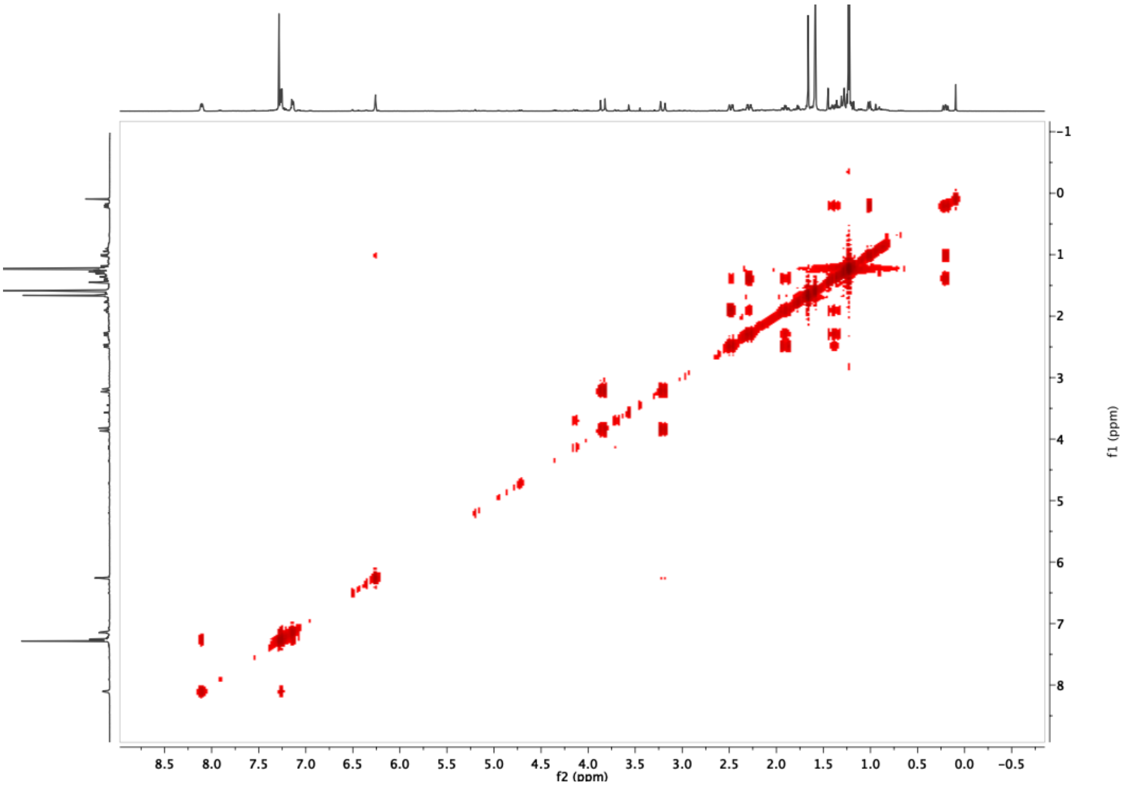

HSQC ^1H-^{13}C of **5a**

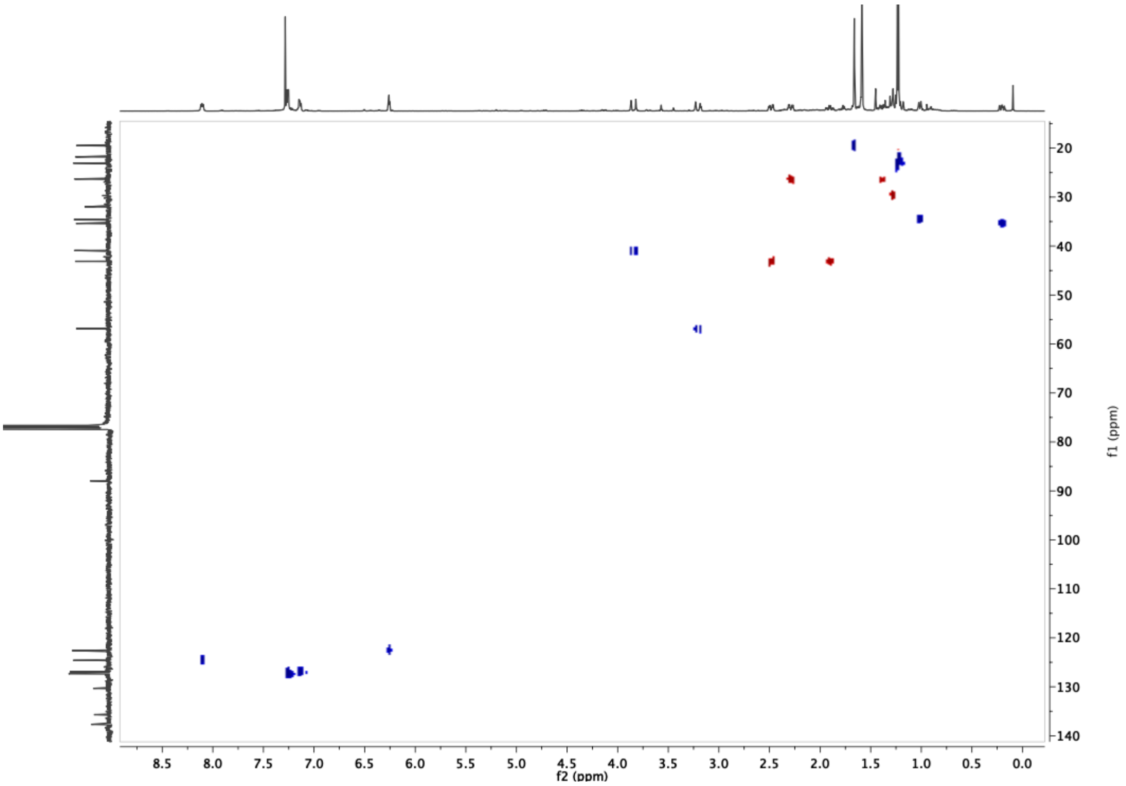

^{1}H-NOE NMR experiments of **5a**

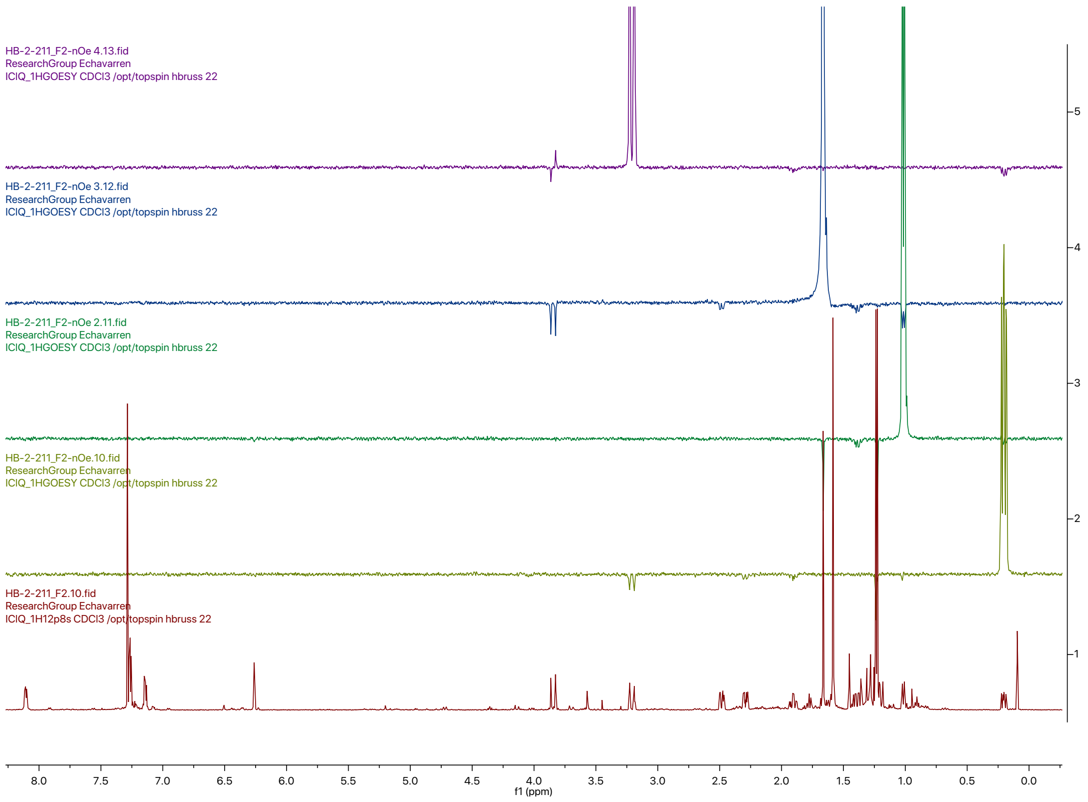

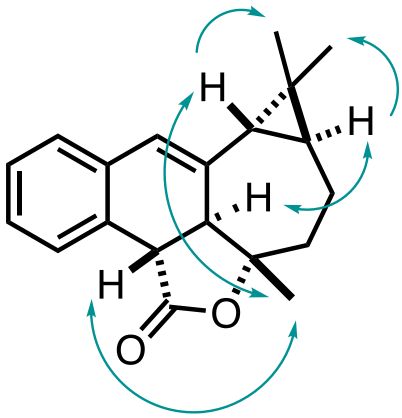

^{1}H-NMR and ^{13}C-NMR of **13** + **14**

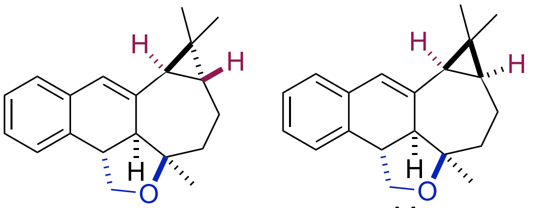

13 **14**

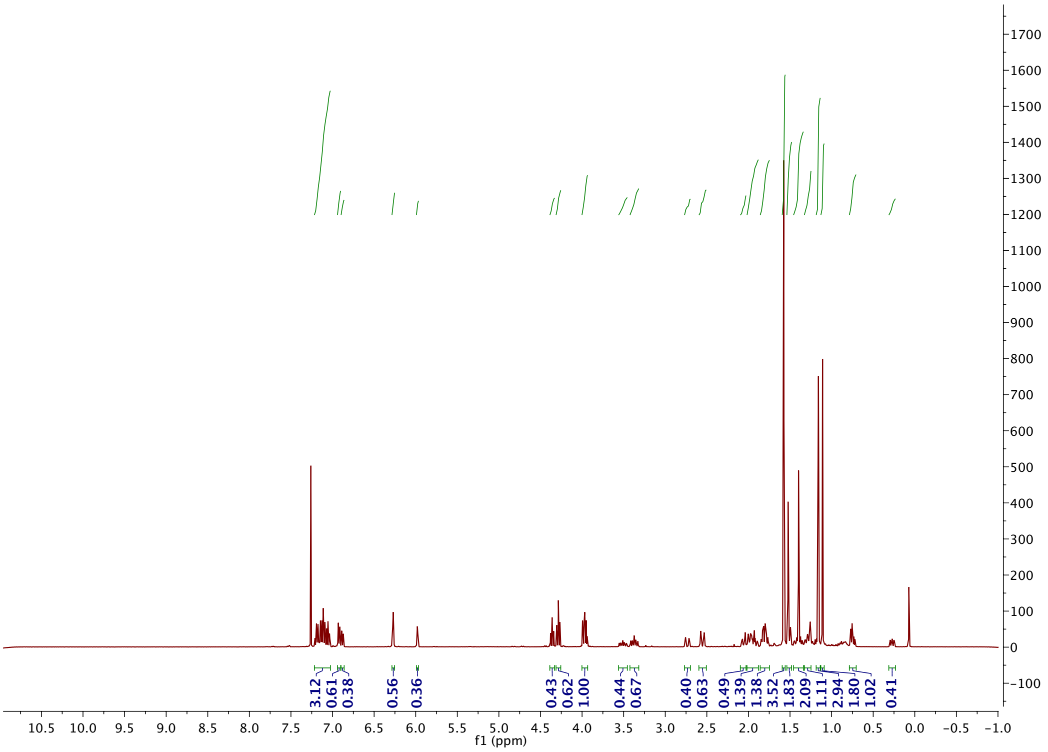

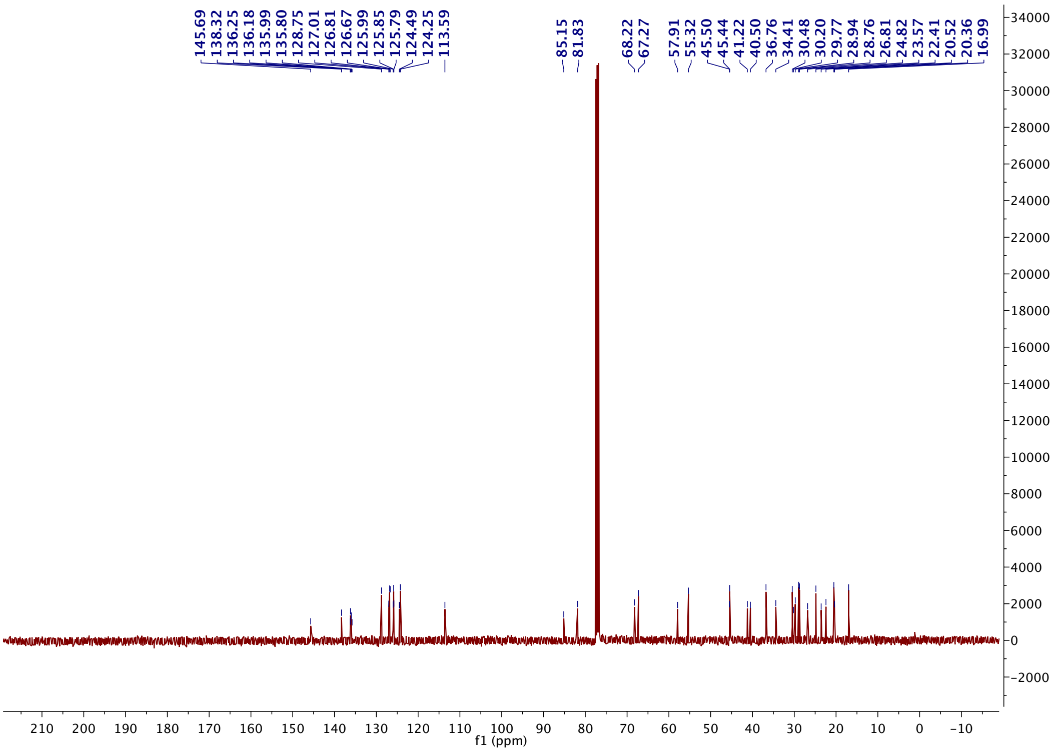

HSQC ^{1}H-^{13}C of **13**+**14**

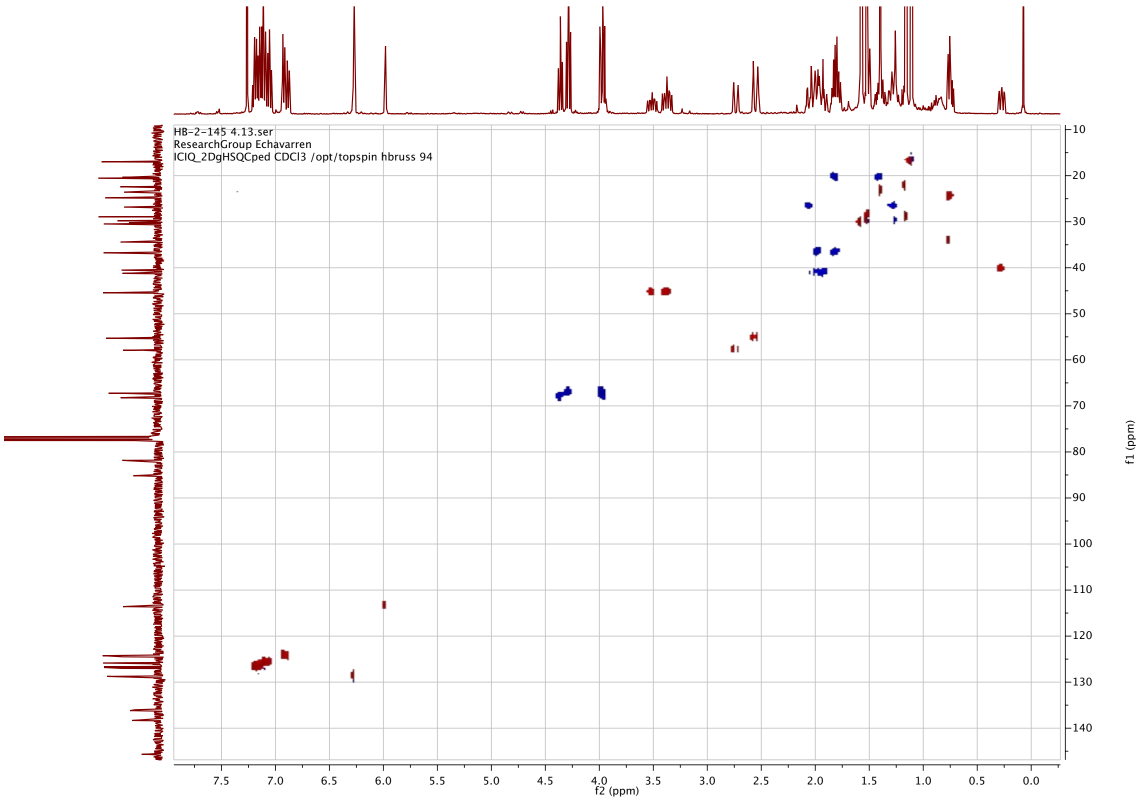

HMBC ^{1}H-^{13}C of **13**+**14**

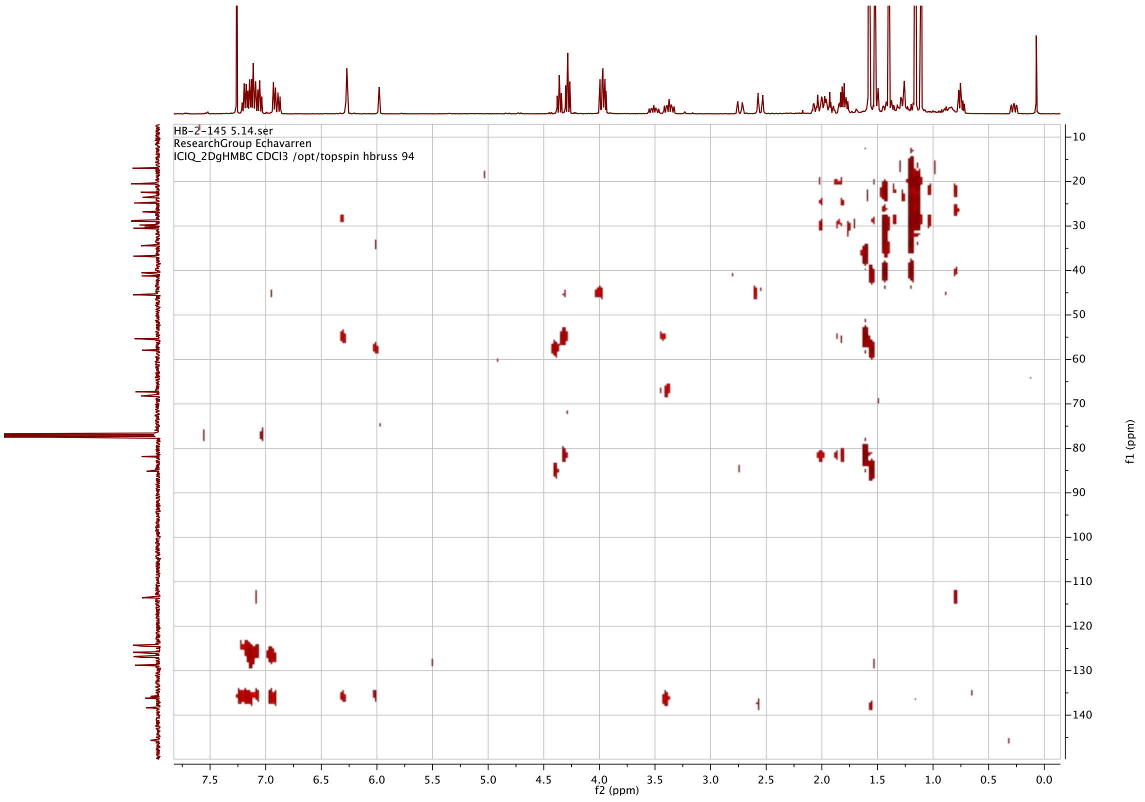

The irradiated proton is painted on **blue**. In the spectra, the **black circles** indicate the NOE signal, that is represented by a **blue arrow** on the structures.

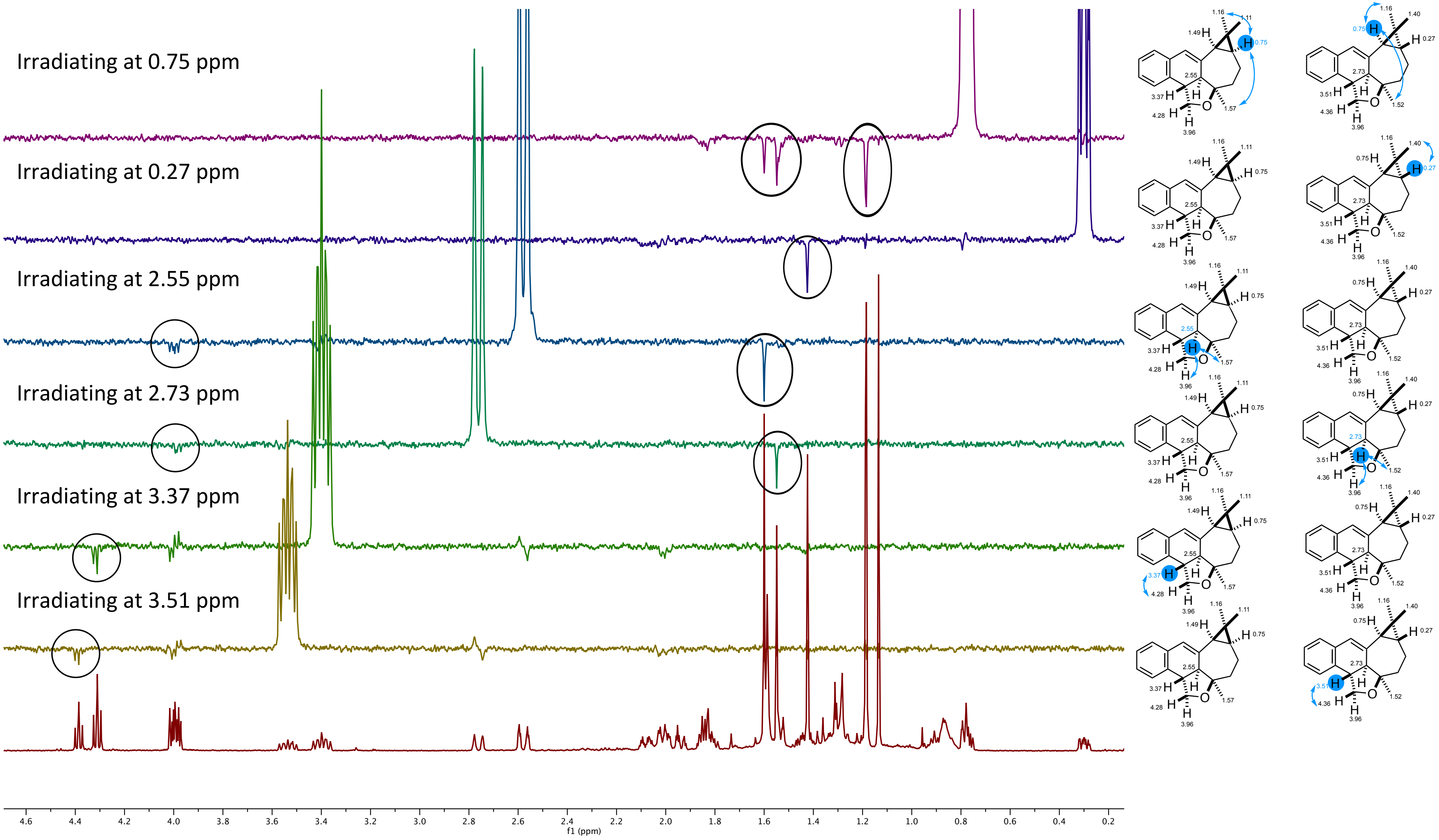

^{1}H-NMR and ^{13}C-NMR of **12**

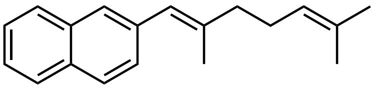

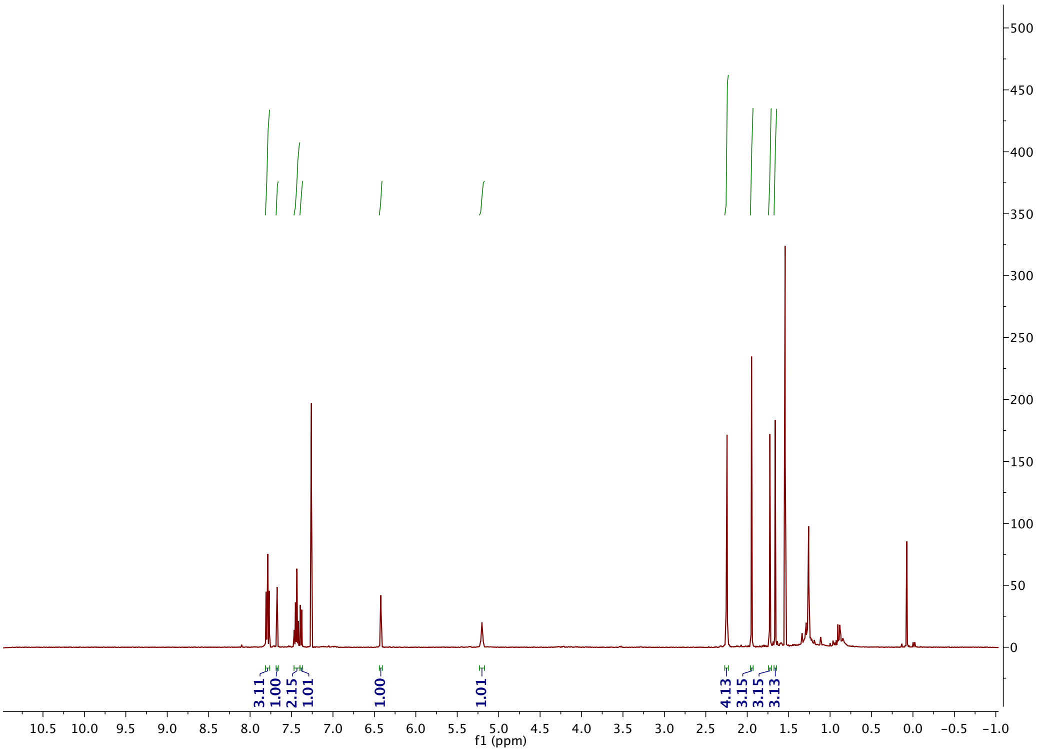

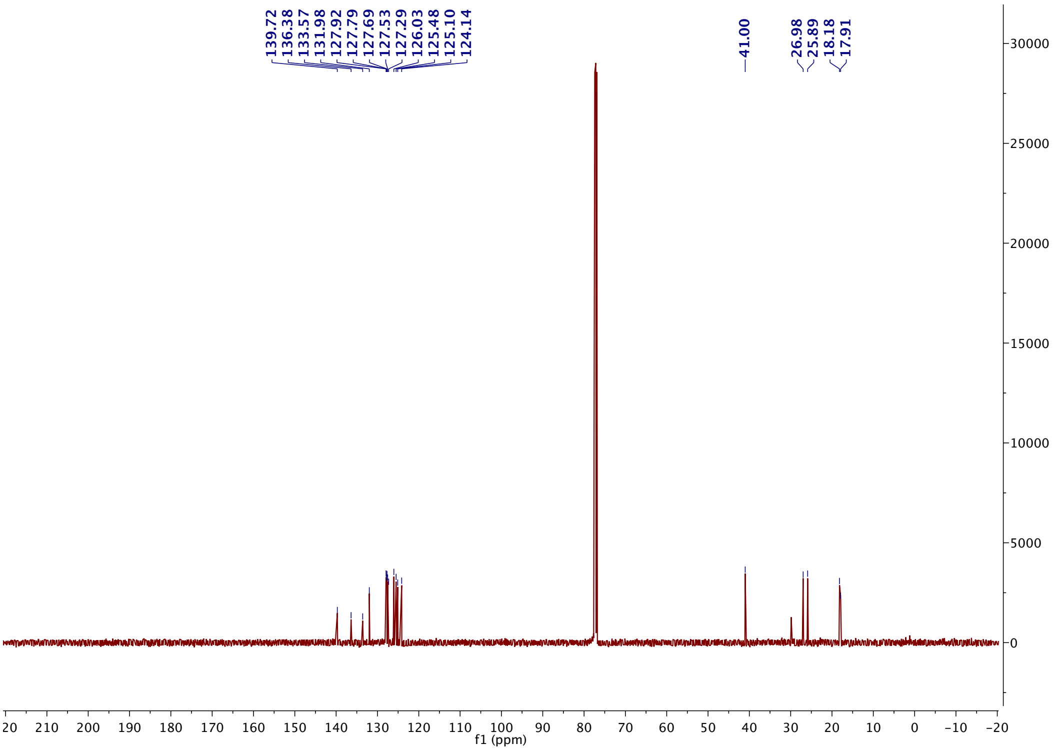

^{1}H-NMR and ^{13}C-NMR of **12’**

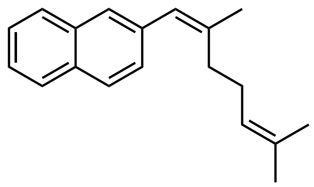

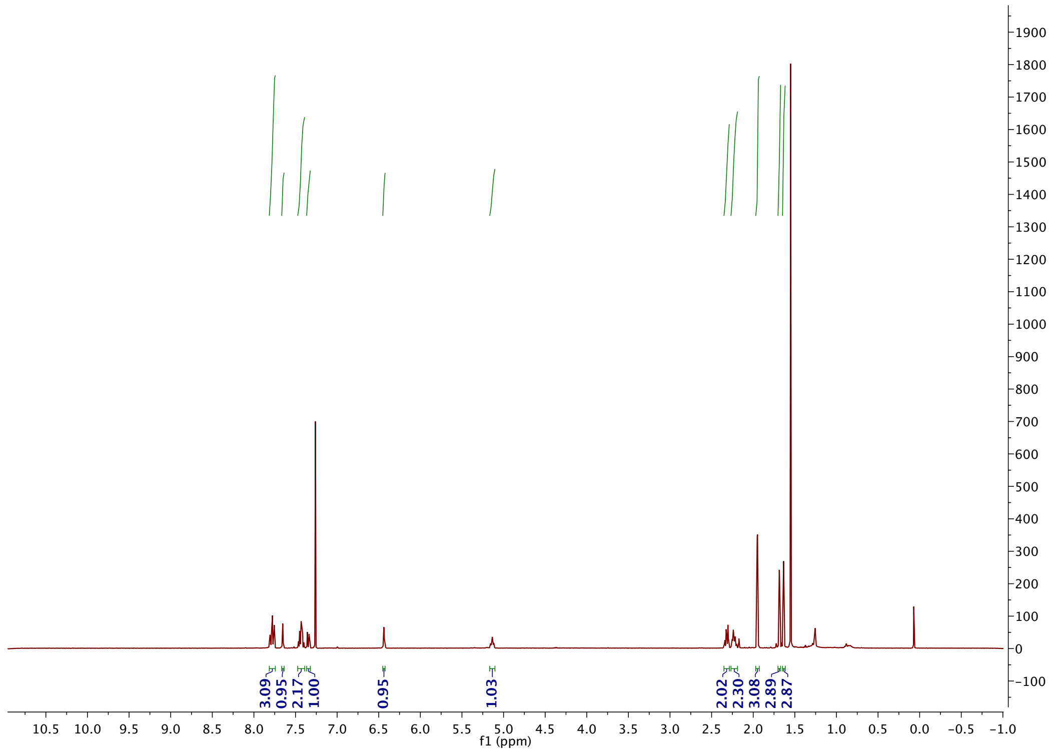

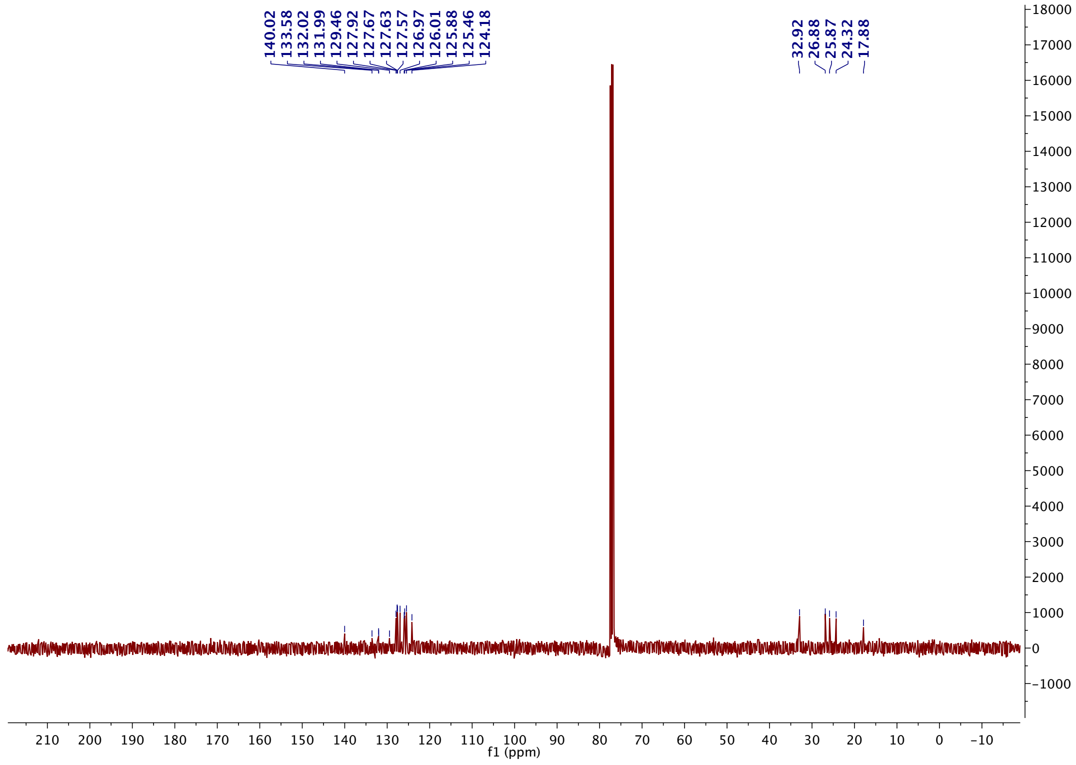

^{1}H-NMR and ^{13}C-NMR of **17a**

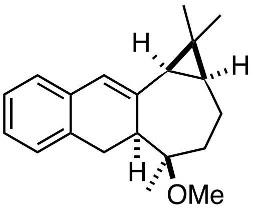

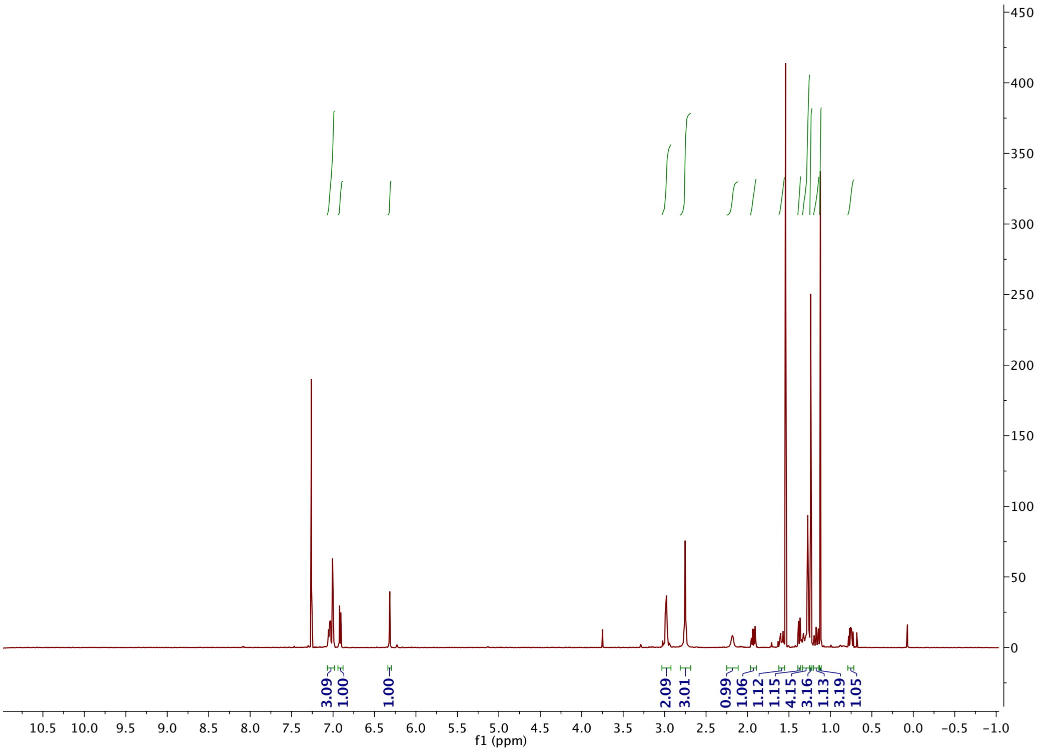

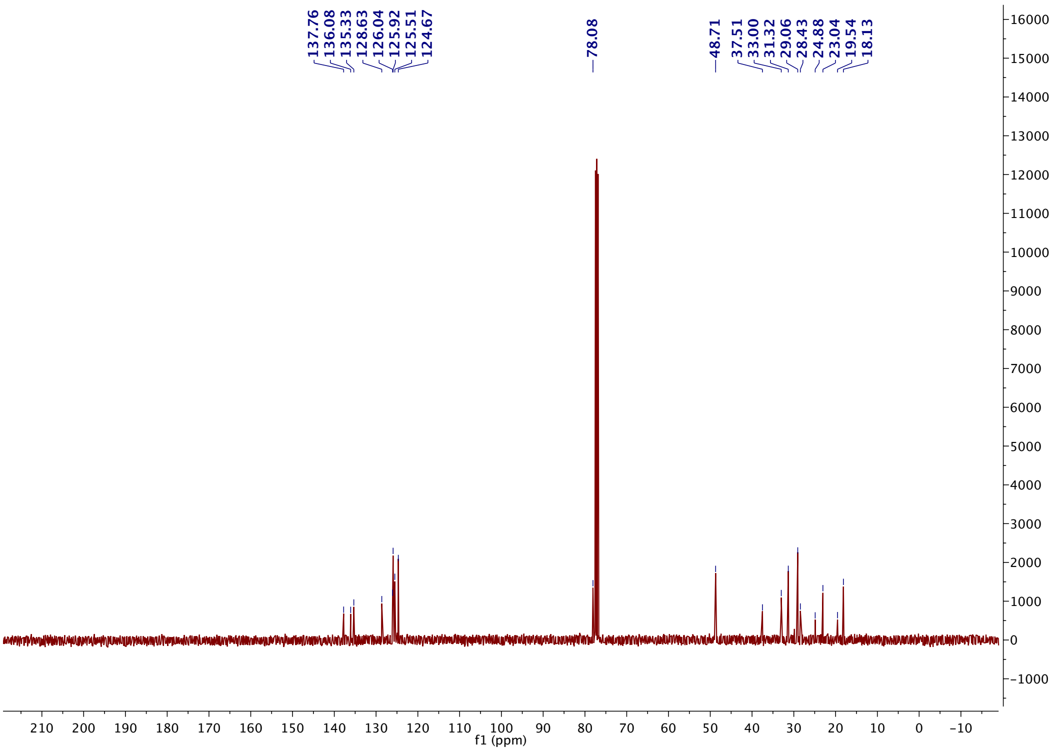

^{1}H-NMR and ^{13}C-NMR of **18**

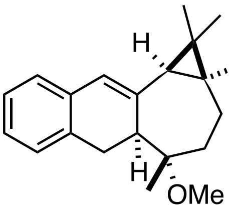

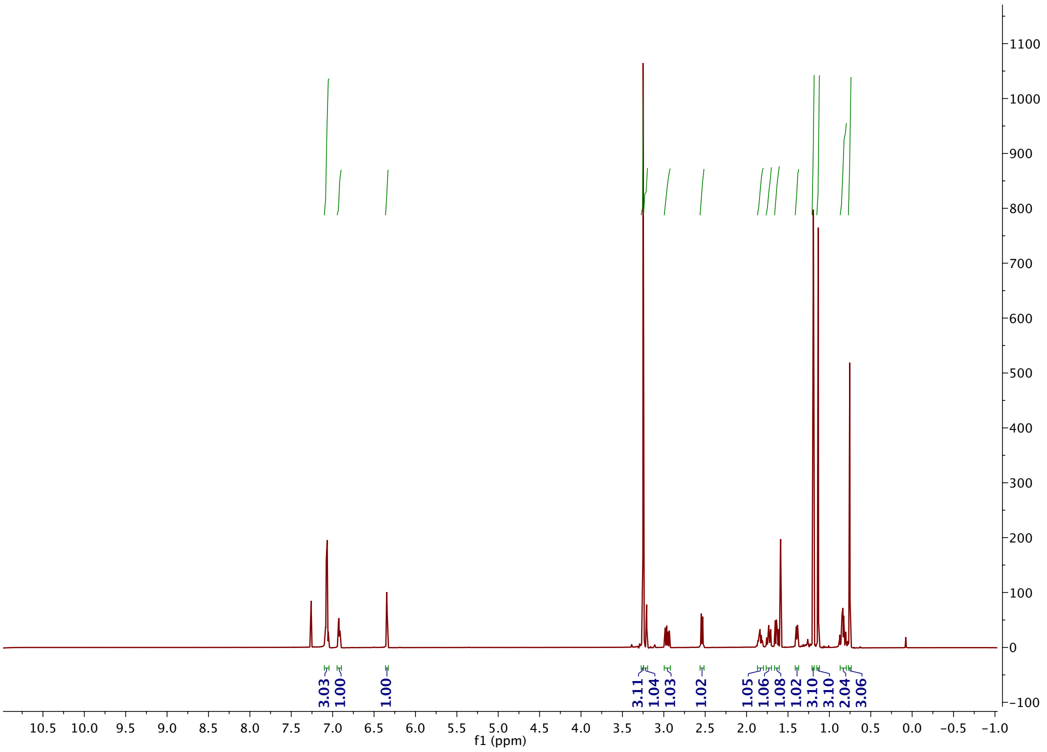

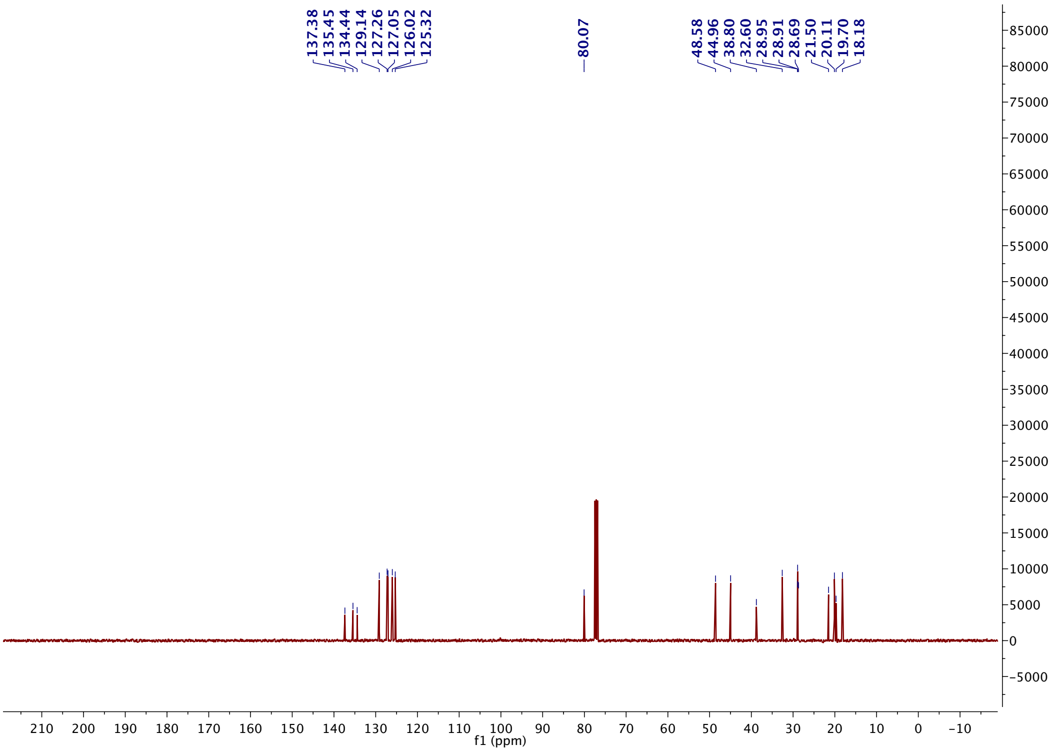

^{1}H-NMR and ^{13}C-NMR of **17b**

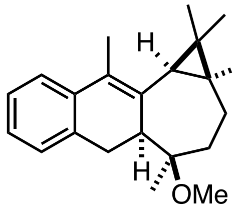

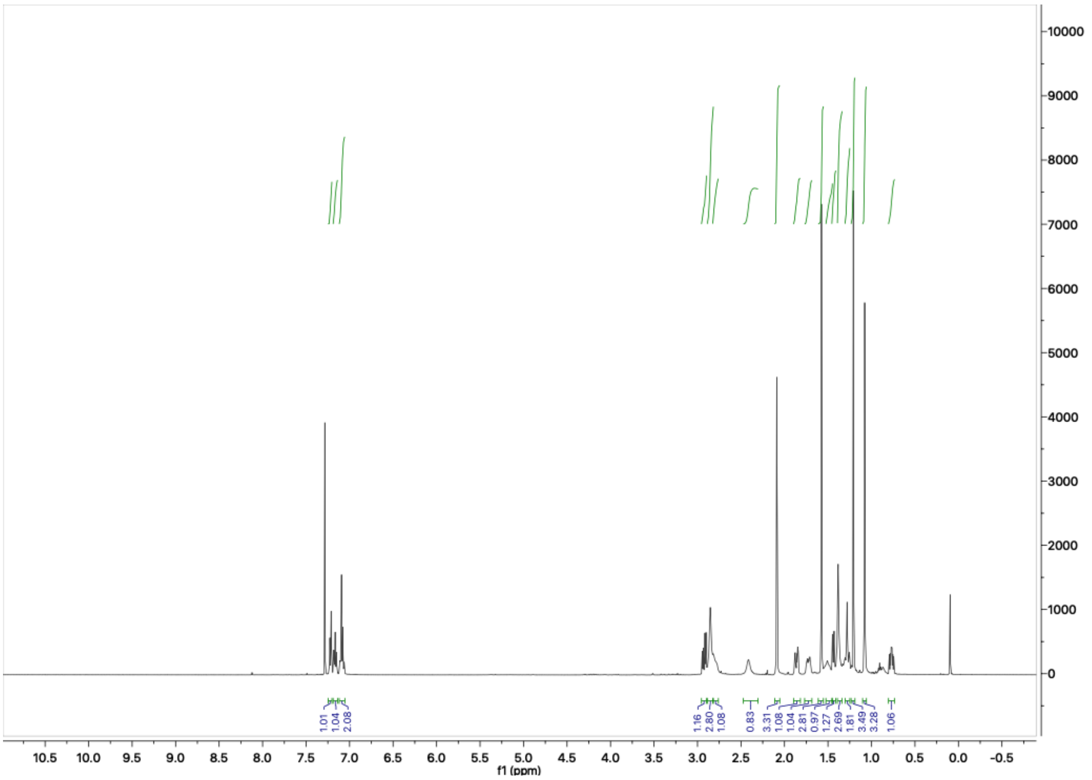

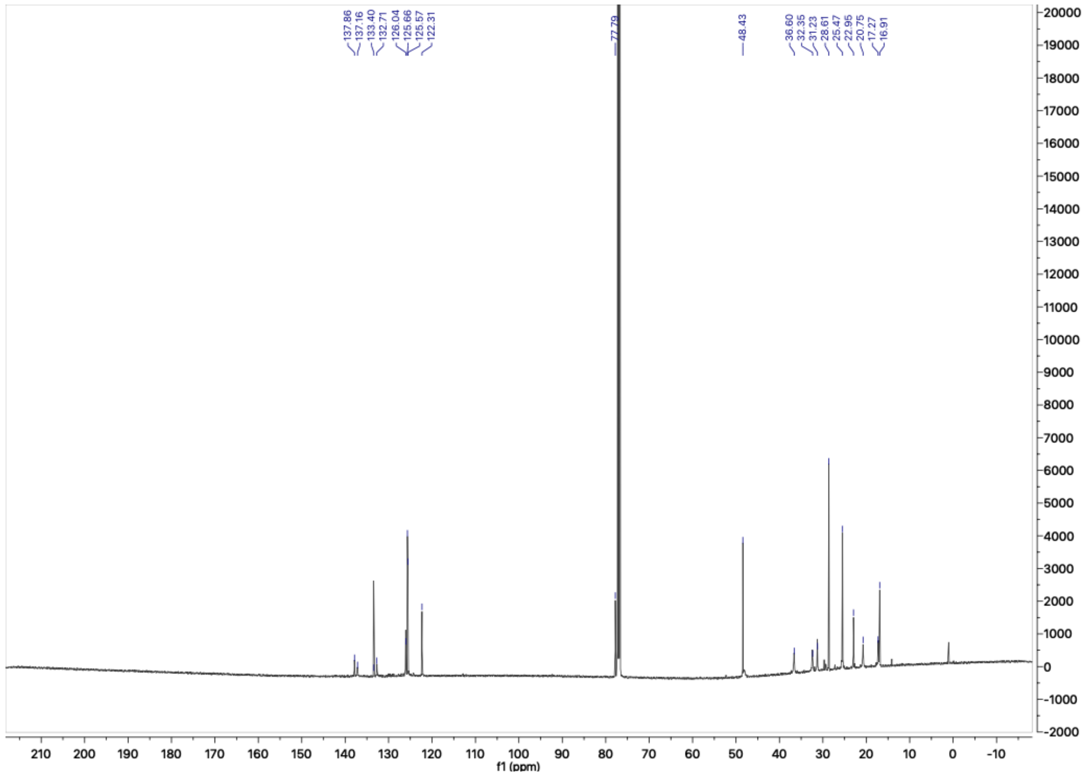

^{1}H-NMR and ^{13}C-NMR of **20**

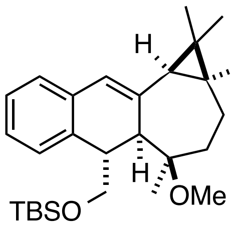

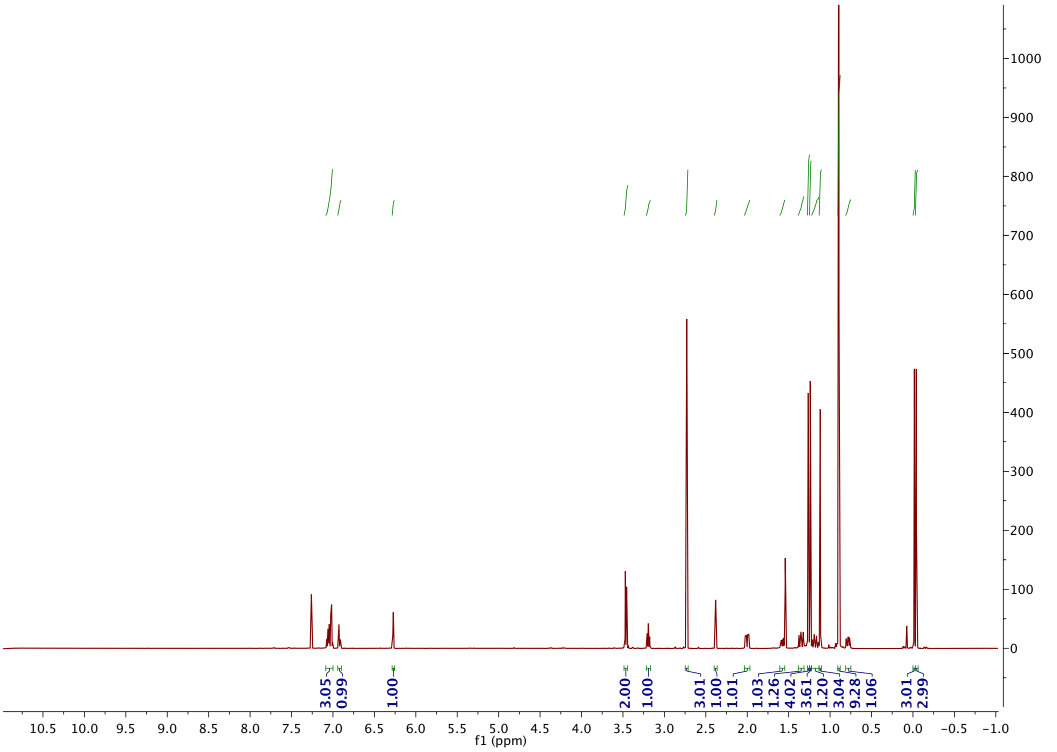

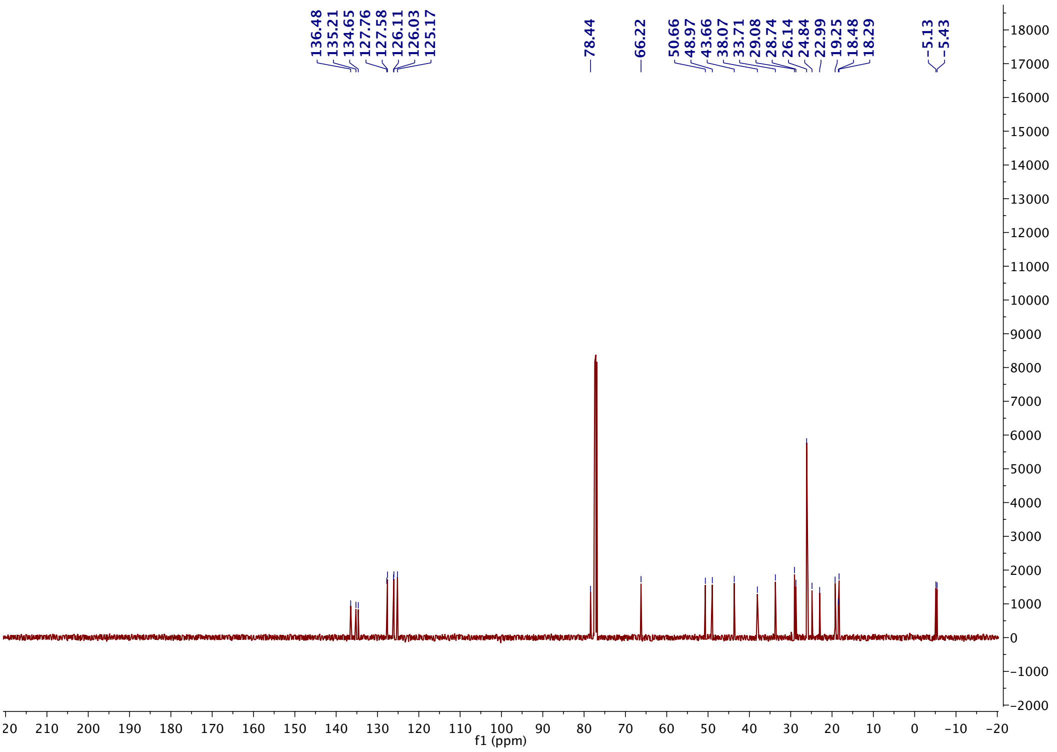

^{1}H-NMR and ^{13}C-NMR of **21**

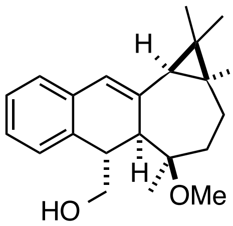

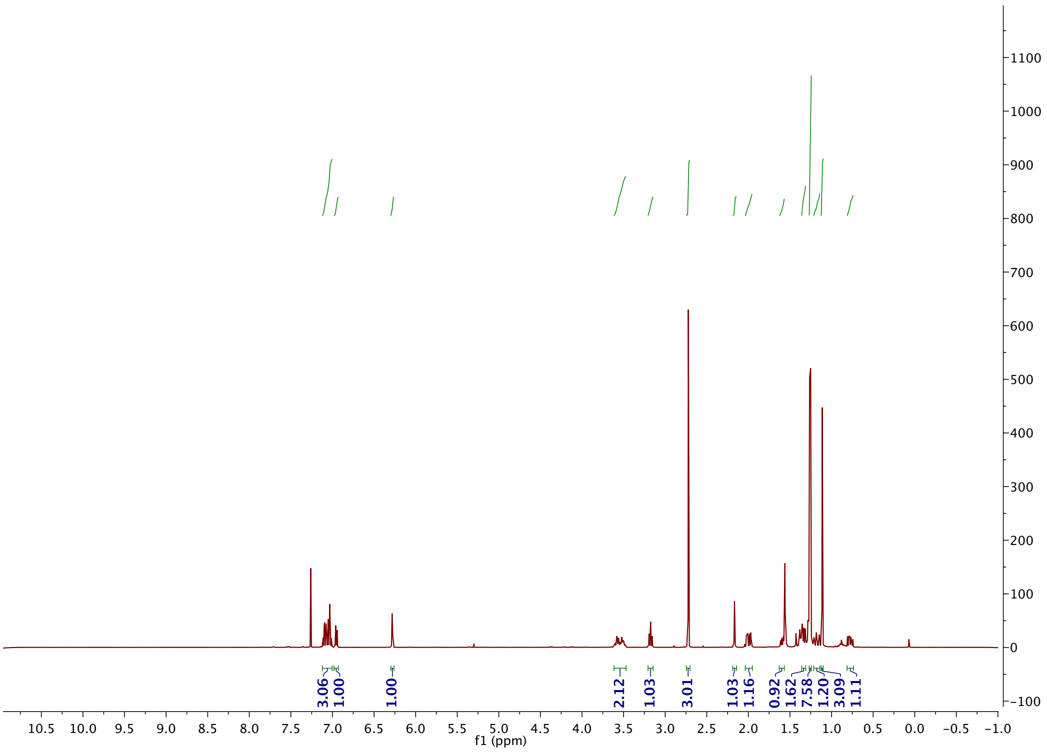

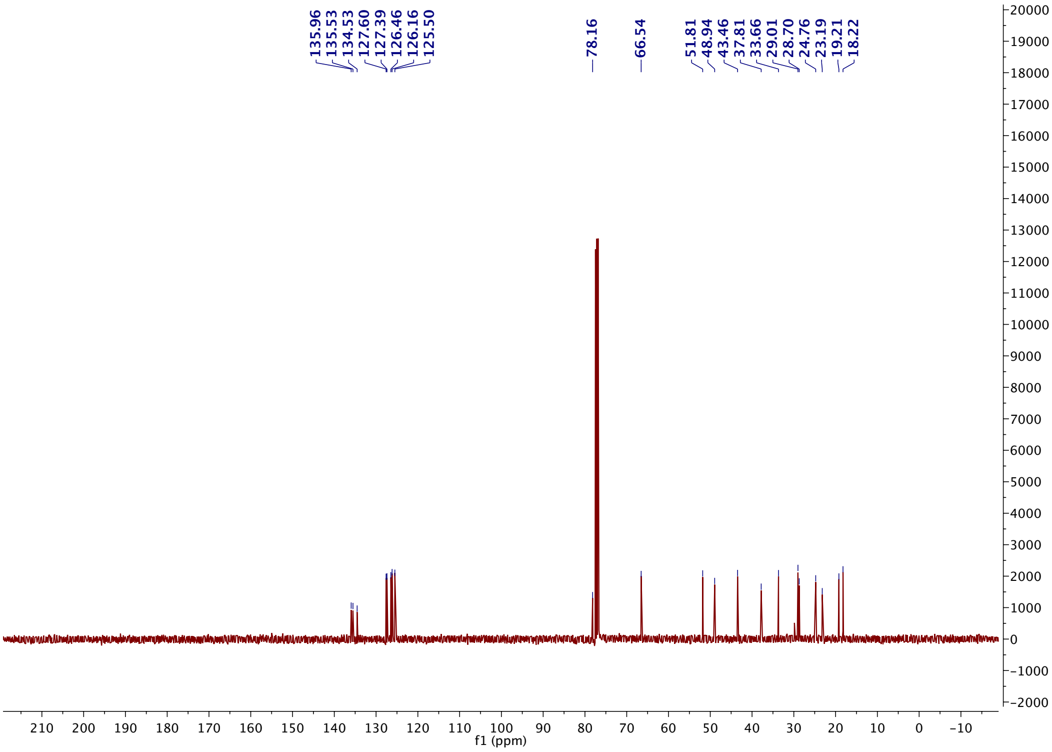

^{1}H-NMR and ^{13}C-NMR of **22**

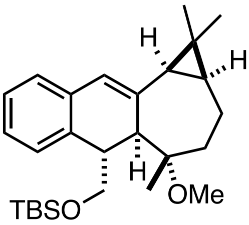

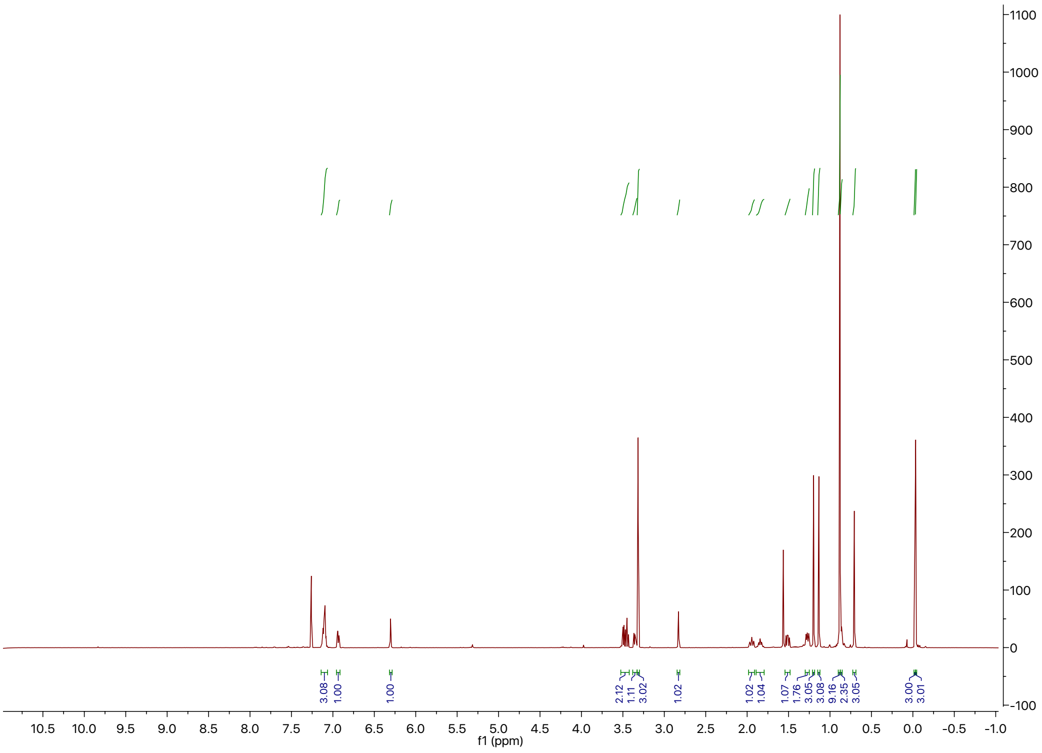

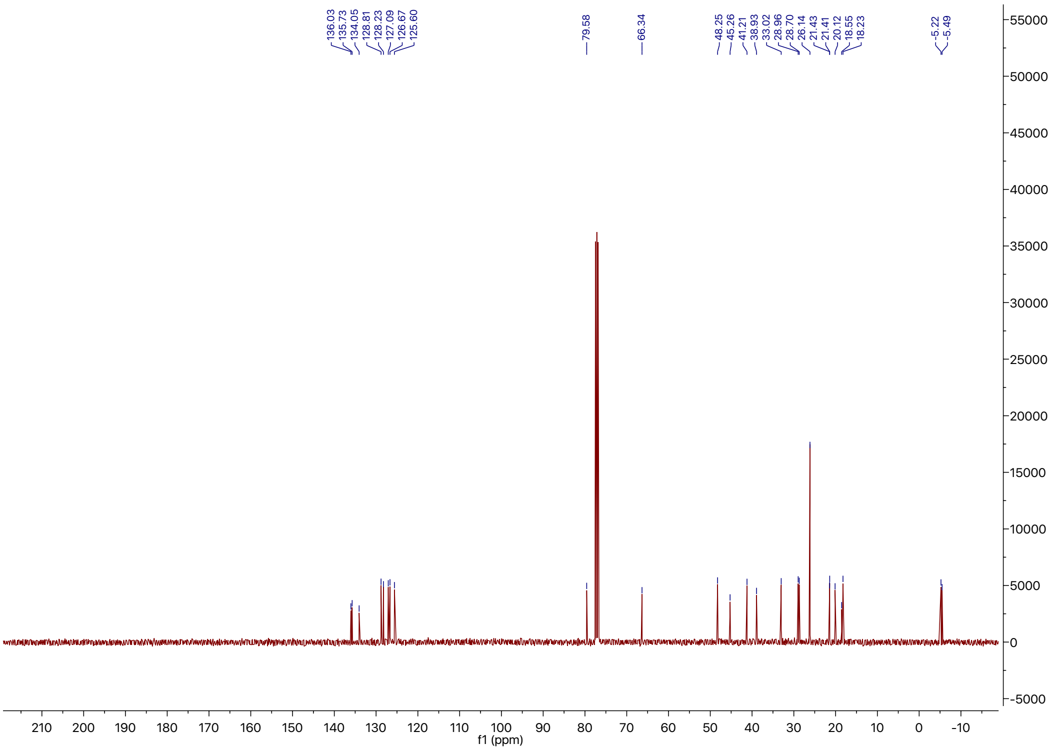

^{1}H-NMR and ^{13}C-NMR of **23**

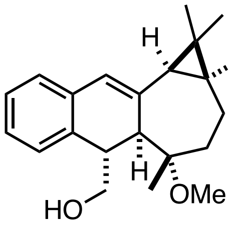

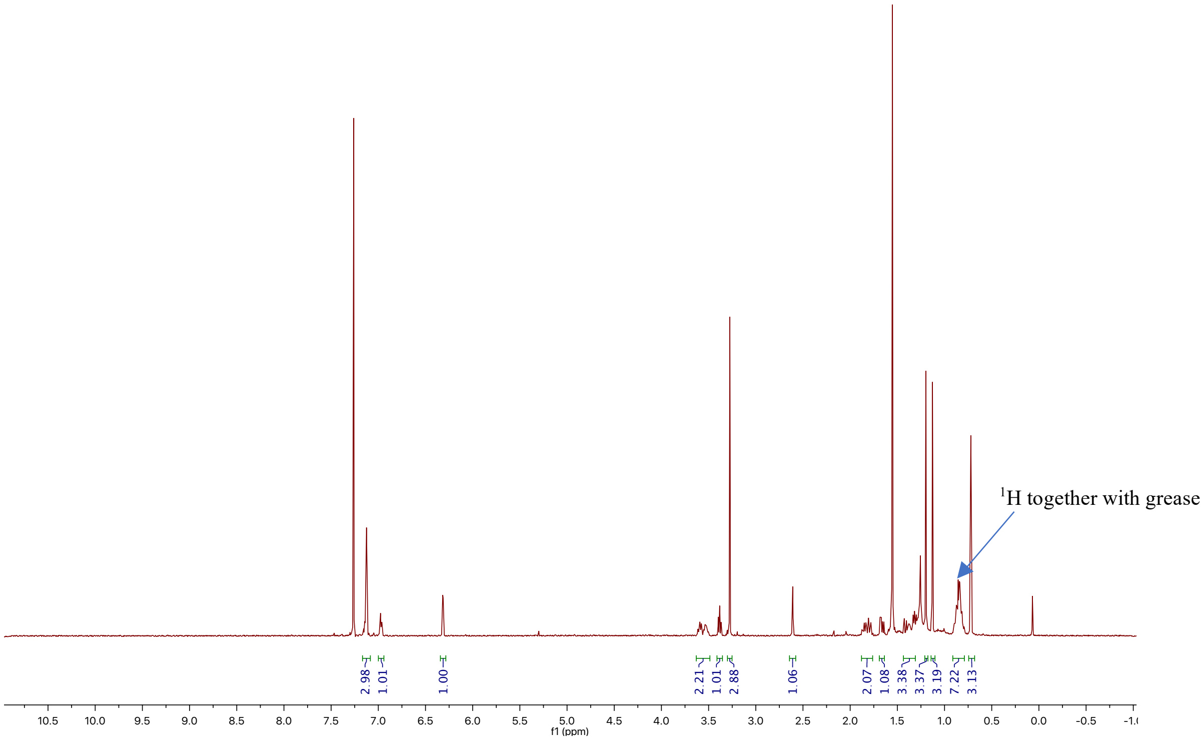

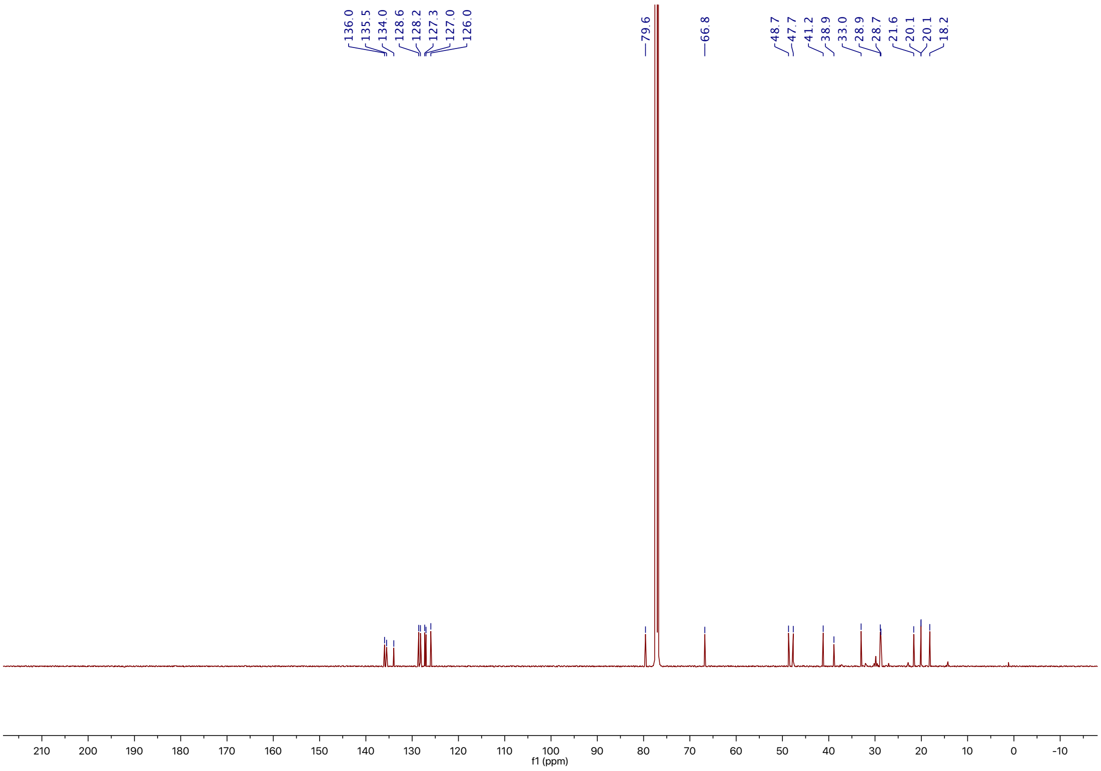

^{1}H-NMR and ^{13}C-NMR of **S19**

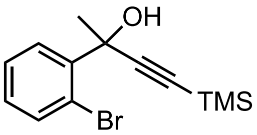

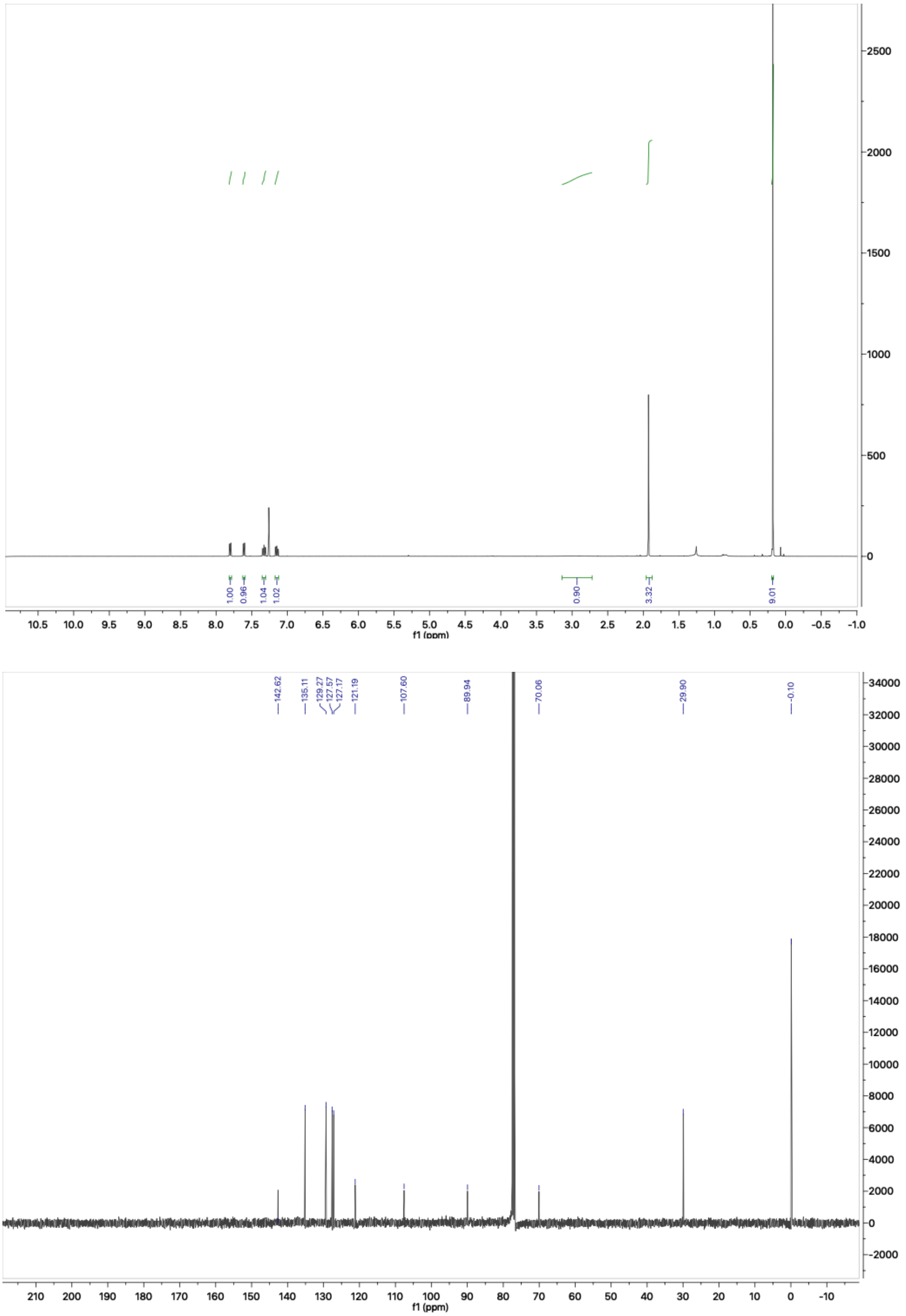

^{1}H-NMR and ^{13}C-NMR of **S20**

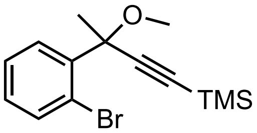

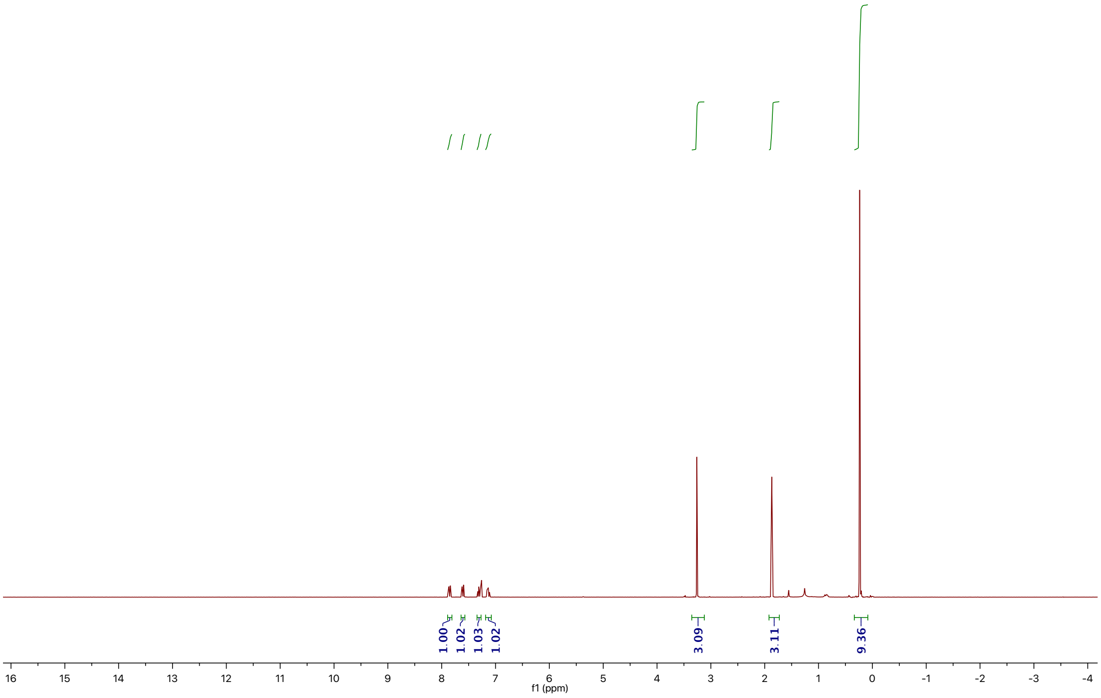

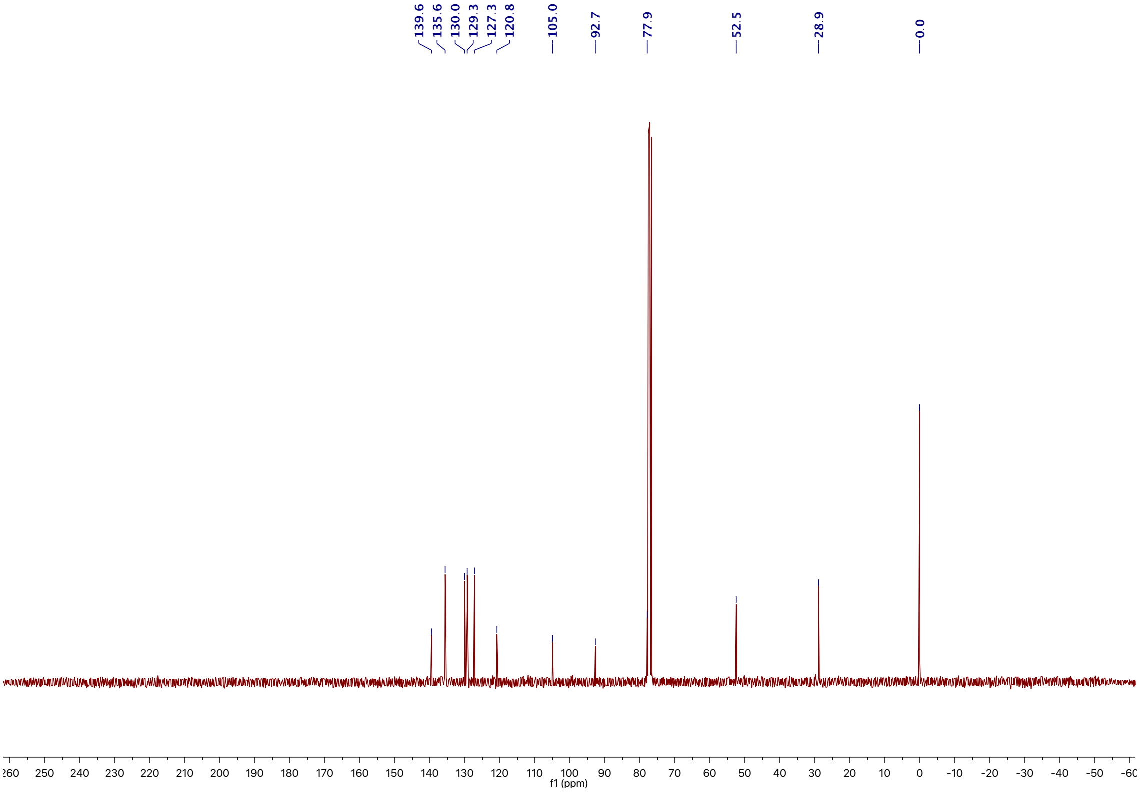

^{1}H-NMR and ^{13}C-NMR of **S21**

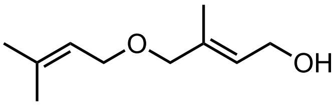

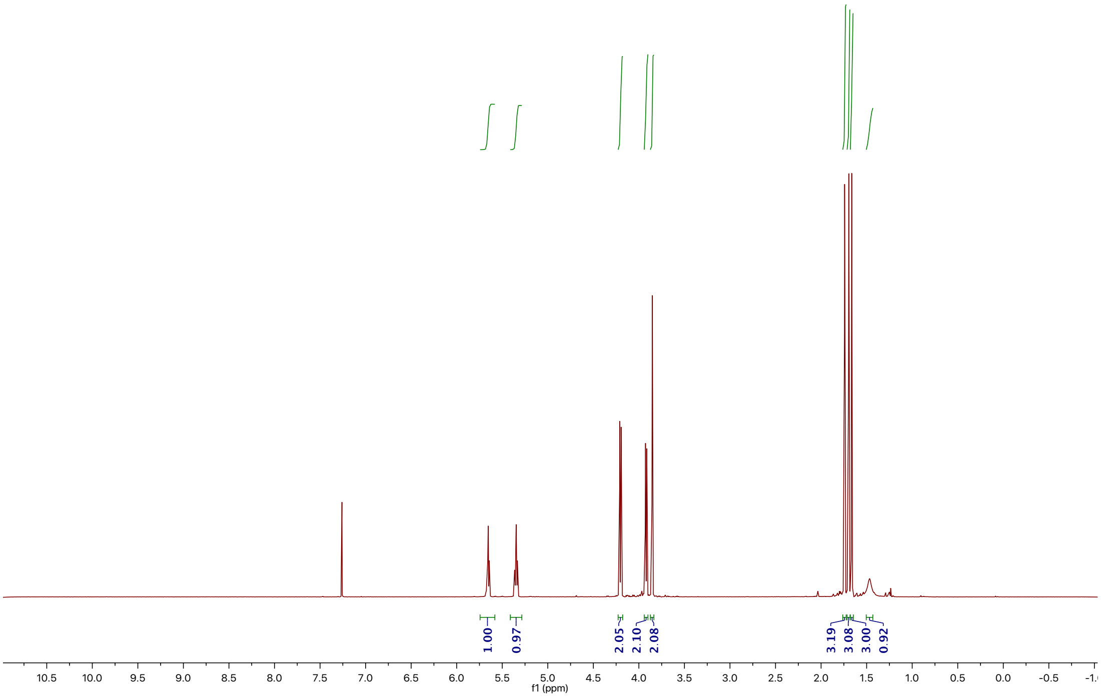

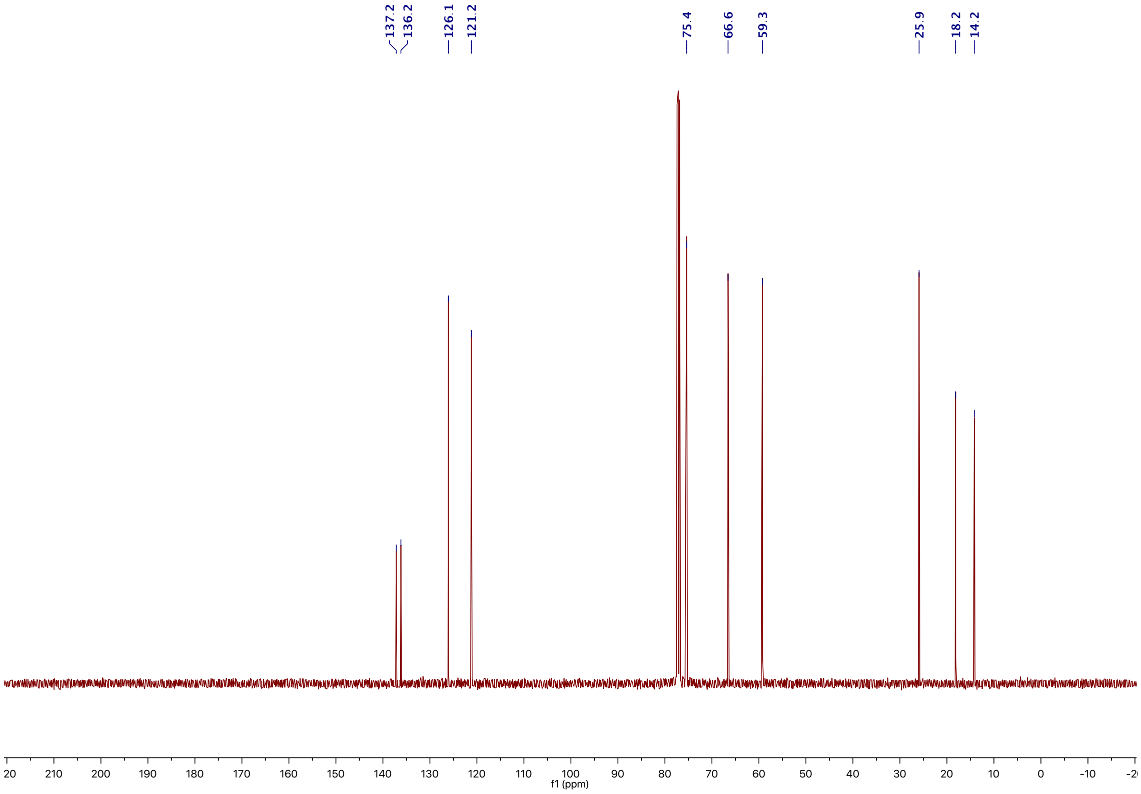

^{1}H-NMR and ^{13}C-NMR of **S22**

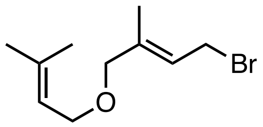

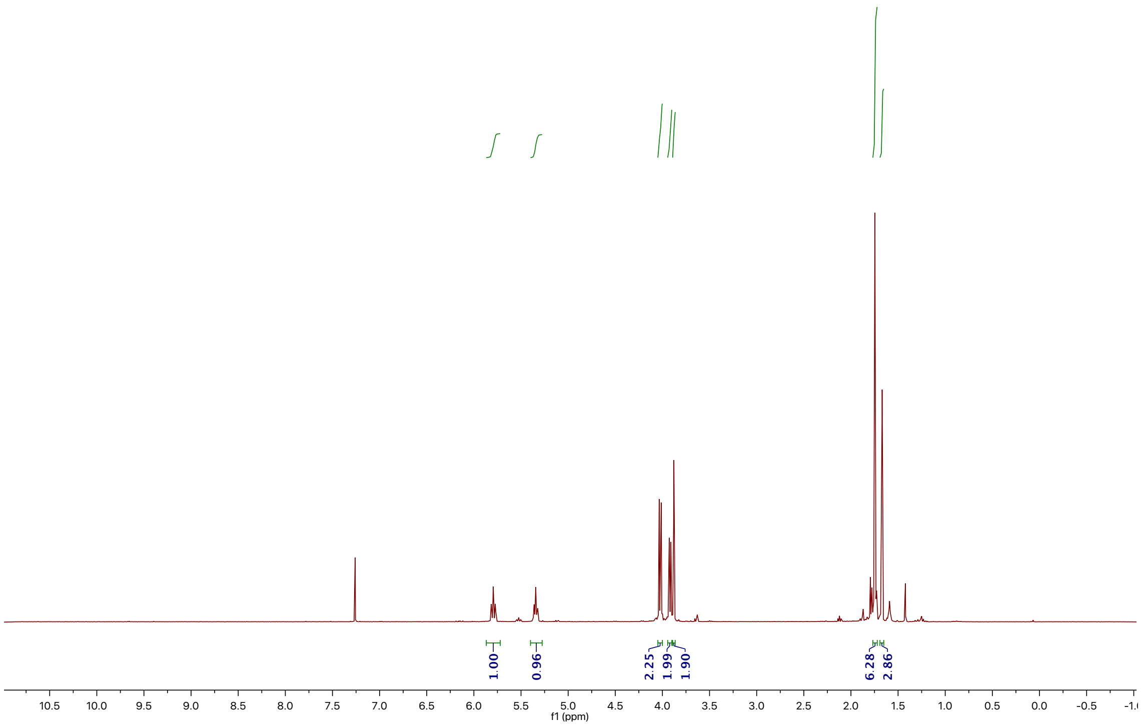

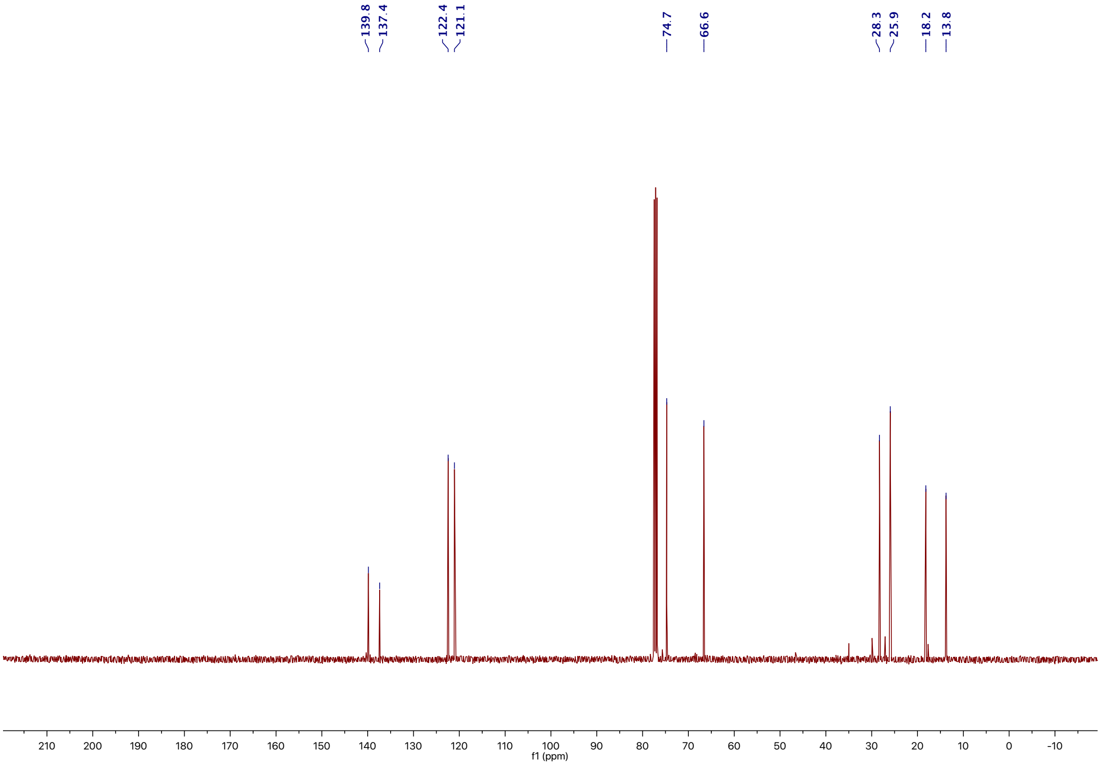

^{1}H-NMR and ^{13}C-NMR of **S16**

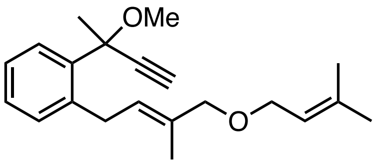

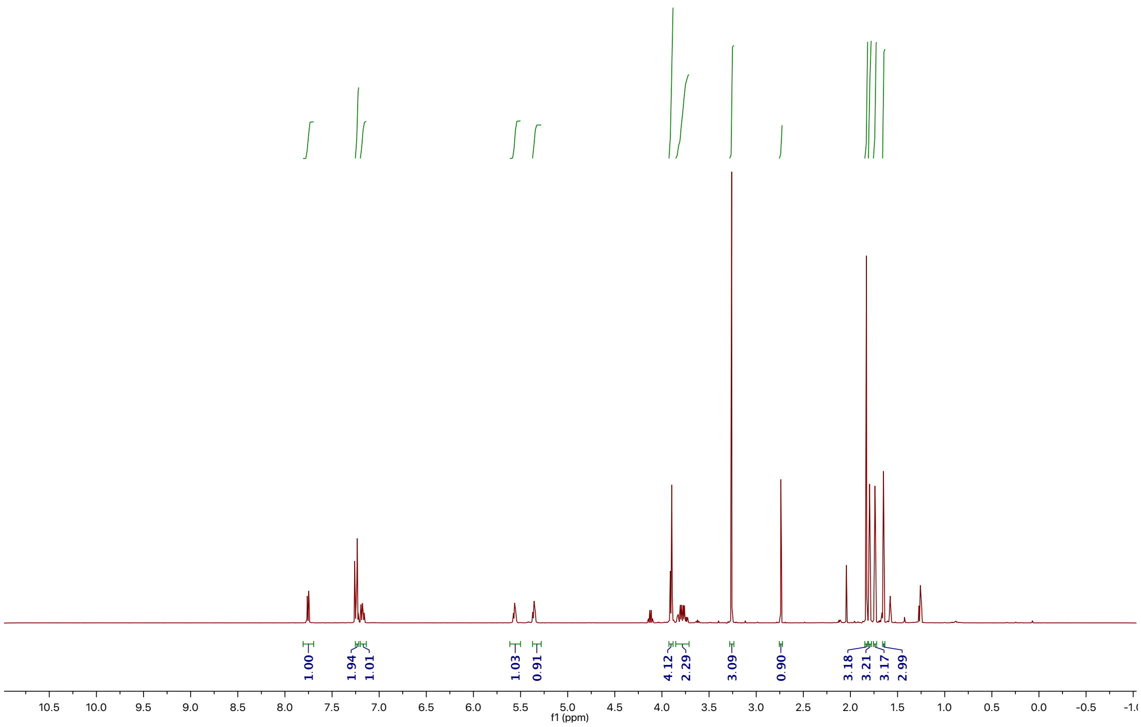

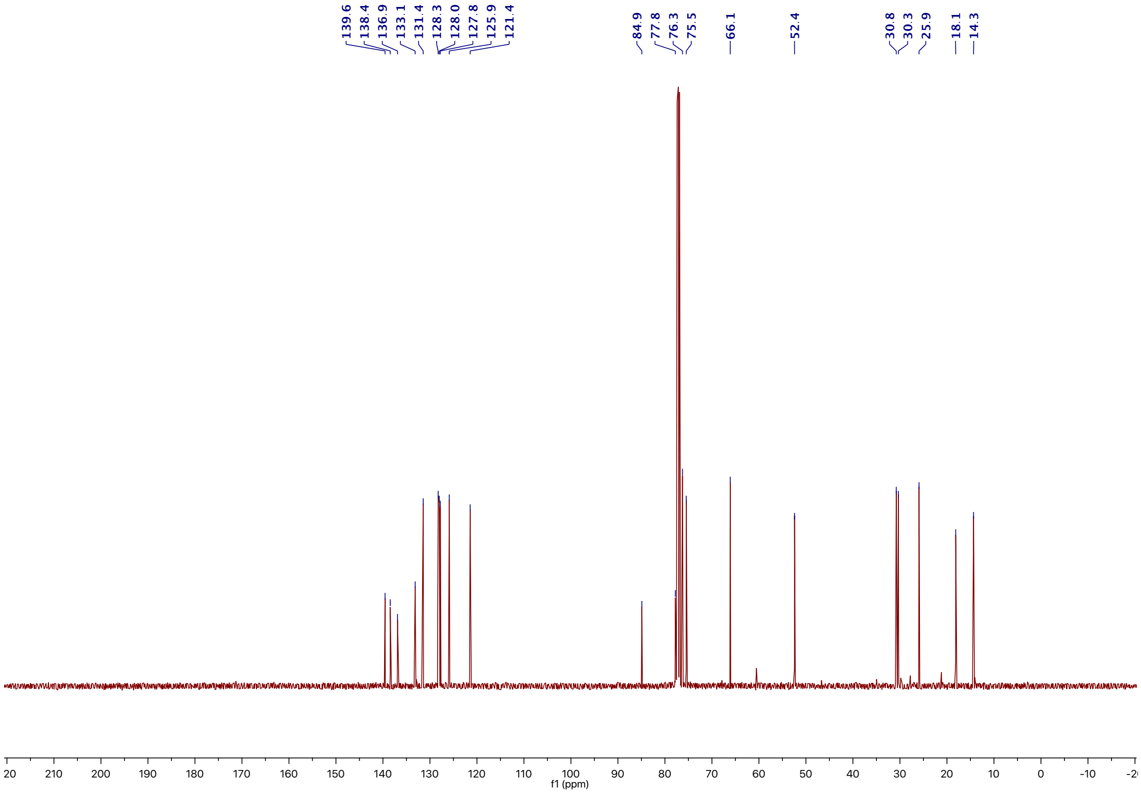

^{1}H-NMR and ^{13}C-NMR of **S17**

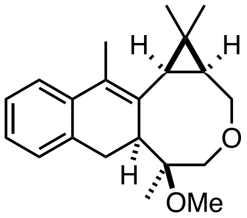

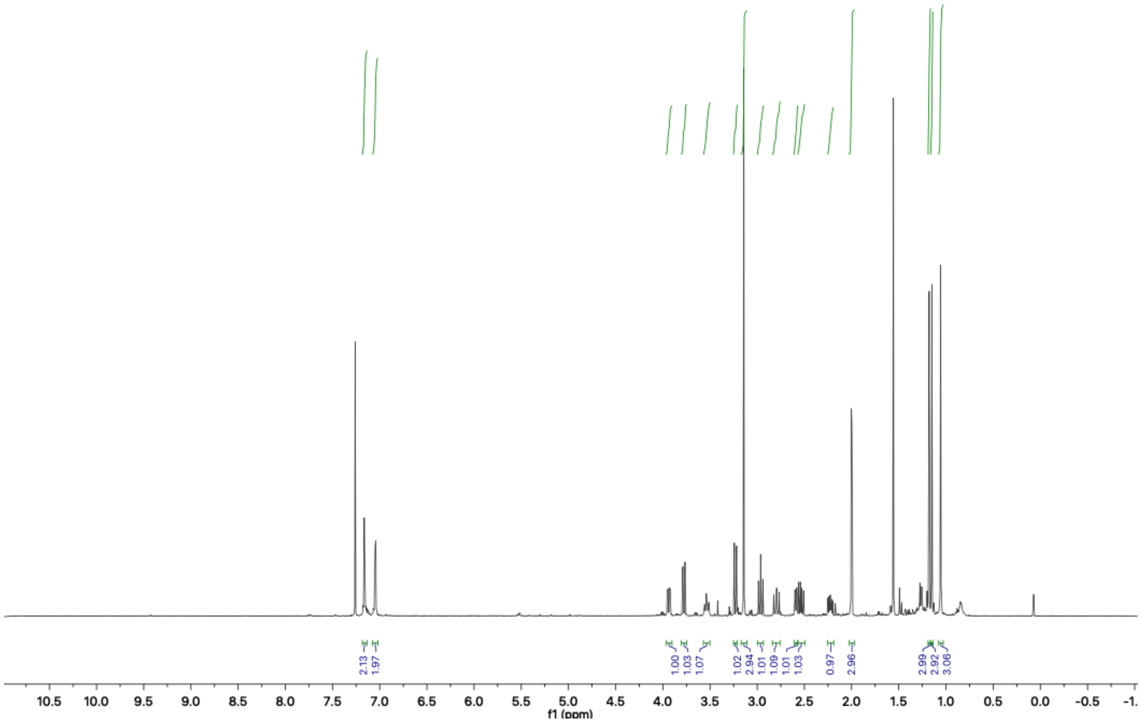

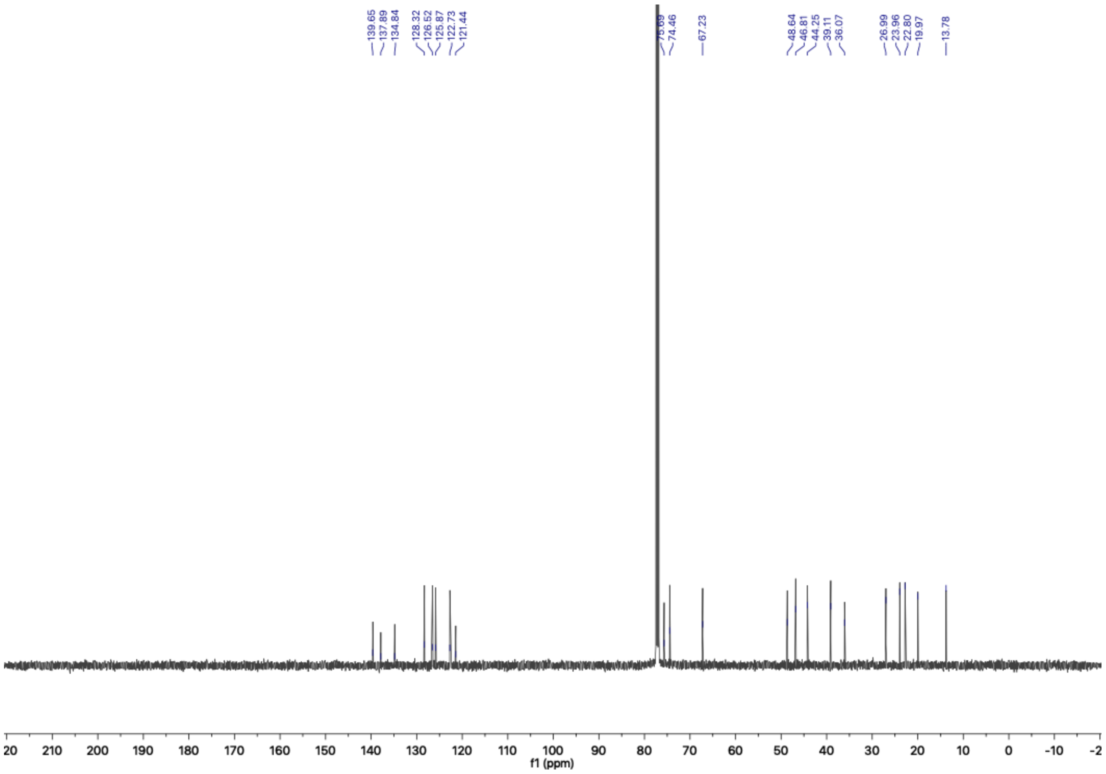

HSQC ^{1}H-^{13}C of **S17**

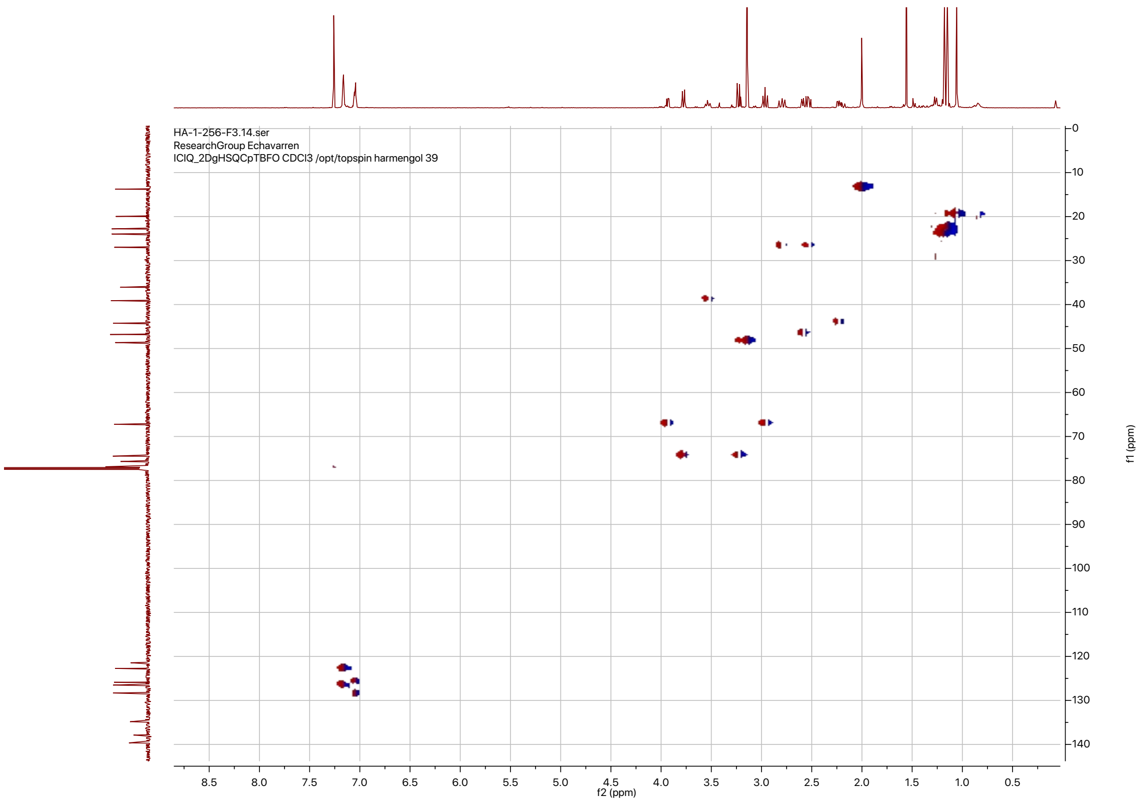

HMBC ^{1}H-^{13}C of **S17**

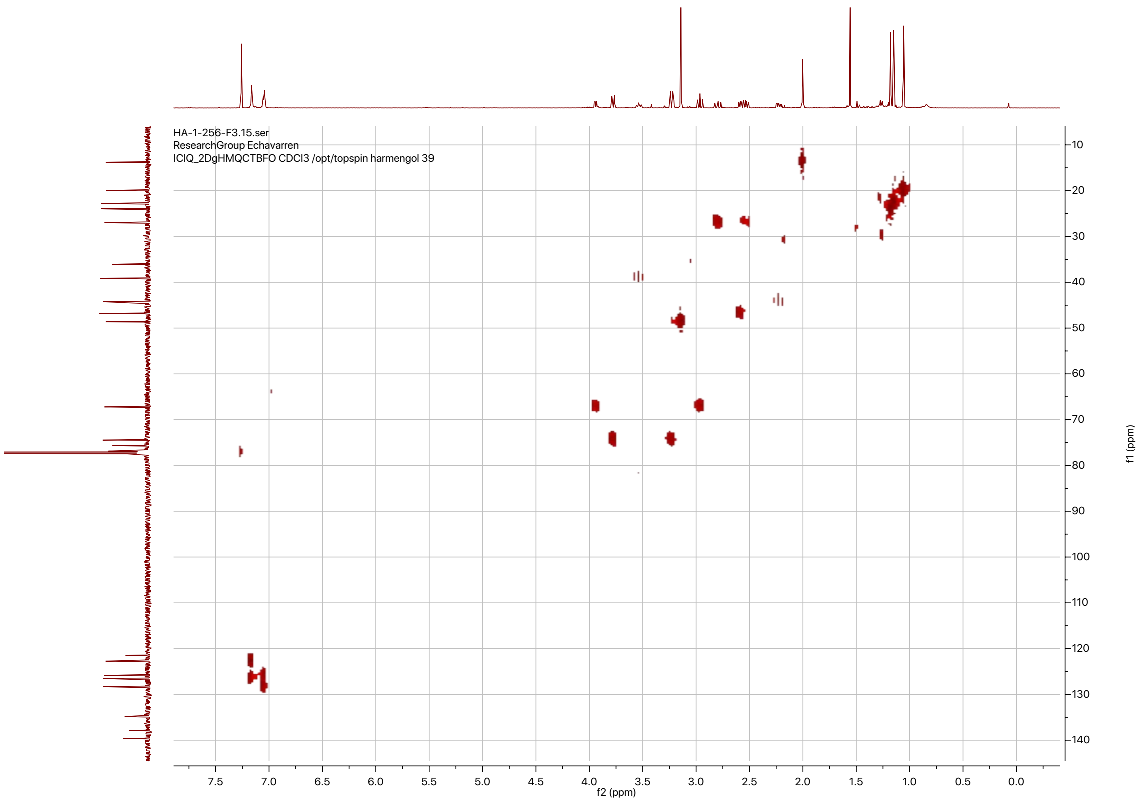

NOESY ^{1}H-^{1}H of **S17**

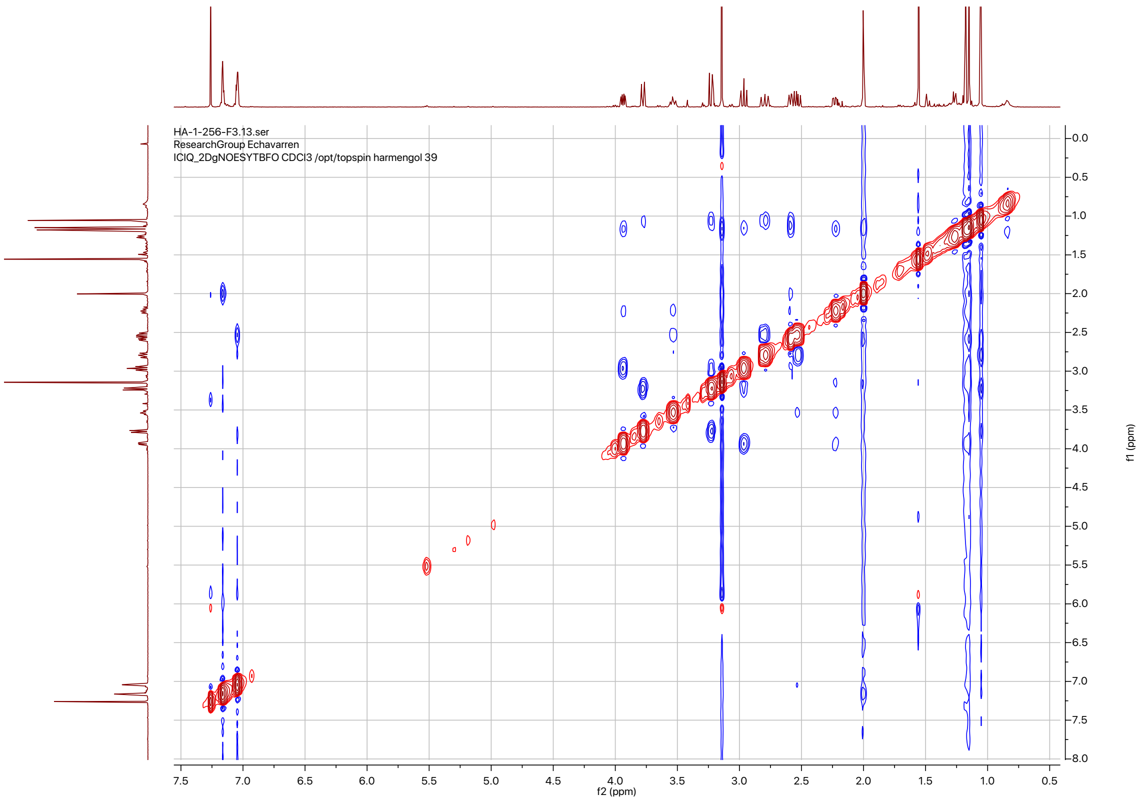

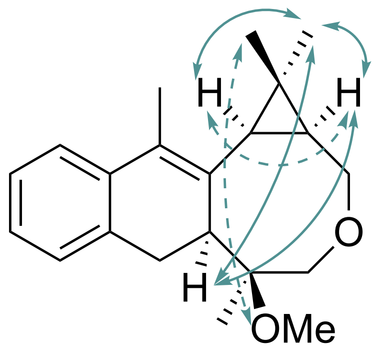

^{1}H-NMR and ^{13}C-NMR of **S18**

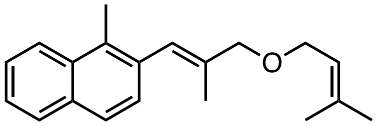

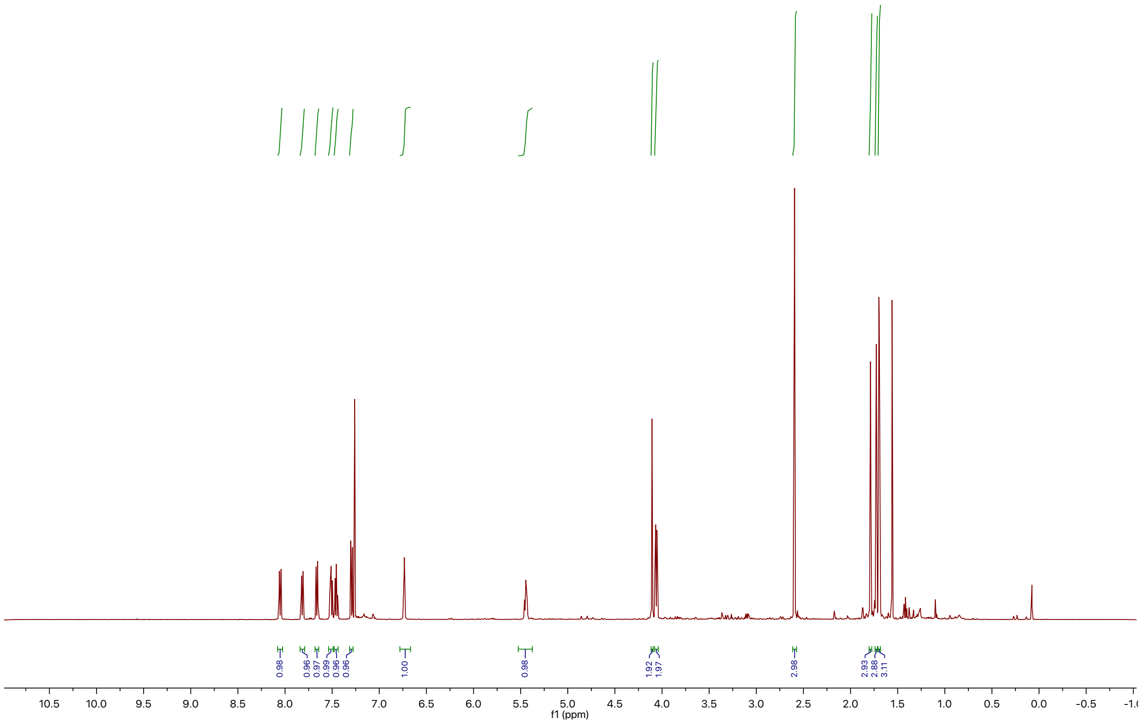

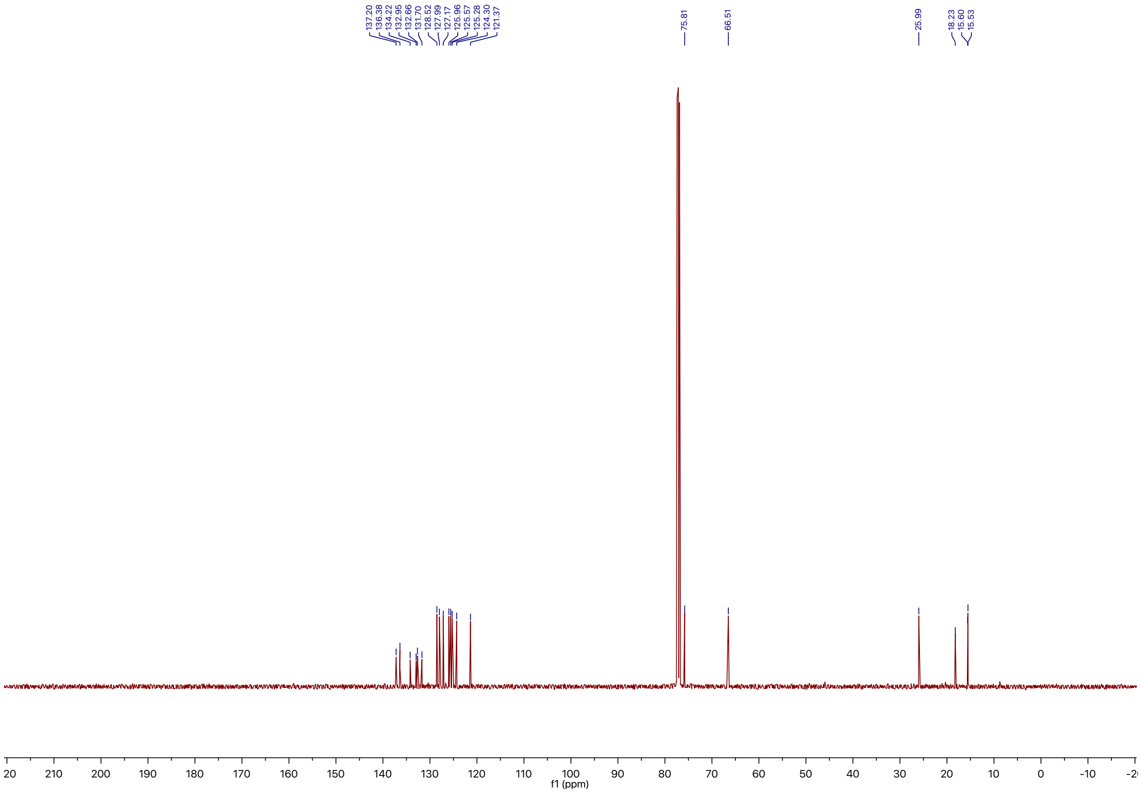

5. **X-ray data**

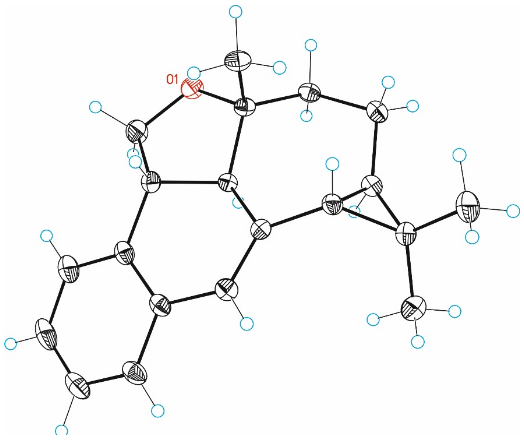

ORTEP representation of **11** with 50% probability of the thermal ellipsoids

Table 1. Crystal data and structure refinement for **11** (CCDC 1906609).

Identification code	mo_HB2154_0m	
Empirical formula	C20 H24 O	
Formula weight	280.39	
Temperature	100(2) K	
Wavelength	0.71073 Å	
Crystal system	Orthorhombic	
Space group	P2(1)2(1)2(1)	
Unit cell dimensions	a = 9.3468(3)Å	α= 90°.
	b = 11.3221(5)Å	β = 90°.
	c = 15.0175(6)Å	γ = 90°.
Volume	1589.23(11) $Å^3$	
Z	4	
Density (calculated)	1.172 Mg/m^3	
Absorption coefficient	0.070 mm^{-1}	
F(000)	608	
Crystal size	0.40 x 0.20 x 0.20 mm^3	
Theta range for data collection	2.253 to 30.568°.	
Index ranges	-7<=h<=13,-16<=k<=16,-21<=l<=20	
Reflections collected	14484	
Independent reflections	4801[R(int) = 0.0350]	
Completeness to theta =30.568°	98.799995%	
Absorption correction	Multi-scan	
Max. and min. transmission	0.986 and 0.927	
Refinement method	Full-matrix least-squares on F^2	
Data / restraints / parameters	4801/ 0/ 193	
Goodness-of-fit on F^2	1.074	
Final R indices [I>2sigma(I)]	R1 = 0.0424, wR2 = 0.1080	
R indices (all data)	R1 = 0.0488, wR2 = 0.1125	
Flack parameter	x =0.3(5)	
Largest diff. peak and hole	0.338 and -0.231 e.$Å^{-3}$	

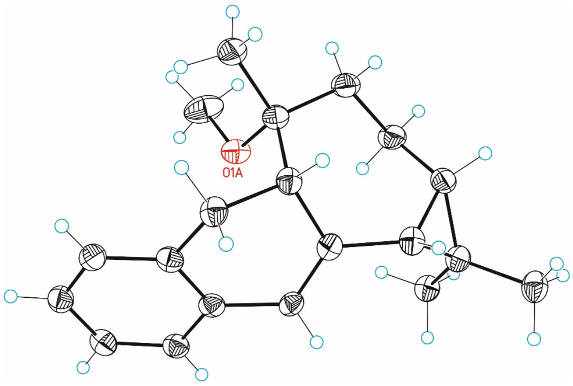

ORTEP representation of **17a** with 50% probability of the thermal ellipsoids

Table 2. Crystal data and structure refinement for **17a** (CCDC 1906610).

Identification code	hfb2-178f	
Empirical formula	C20 H26 O	
Formula weight	282.41	
Temperature	100(2) K	
Wavelength	0.71073 Å	
Crystal system	Triclinic	
Space group	P-1	
Unit cell dimensions	a = 8.3331(2)Å	α= 89.9747(17)°.
	b = 12.4807(2)Å	β = 81.948(2)°.
	c = 15.4876(4)Å	γ = 89.9708(17)°.
Volume	1594.87(6) Å^3	
Z	4	
Density (calculated)	1.176 Mg/m^3	
Absorption coefficient	0.070 mm^{-1}	
F(000)	616	
Crystal size	? x ? x ? mm^3	
Theta range for data collection	2.468 to 26.369°.	
Index ranges	-10<=h<=10,-15<=k<=15,-19<=l<=19	
Reflections collected	24117	
Independent reflections	6500[R(int) = 0.0460]	
Completeness to theta =26.369°	99.7%	
Absorption correction	Multi-scan	
Max. and min. transmission	0.998 and 0.768	
Refinement method	Full-matrix least-squares on F^2	
Data / restraints / parameters	6500/ 0/ 388	
Goodness-of-fit on F^2	1.229	
Final R indices [I>2sigma(I)]	R1 = 0.0642, wR2 = 0.1684	
R indices (all data)	R1 = 0.0664, wR2 = 0.1697	
Largest diff. peak and hole	0.682 and -0.281 e.Å^{-3}	

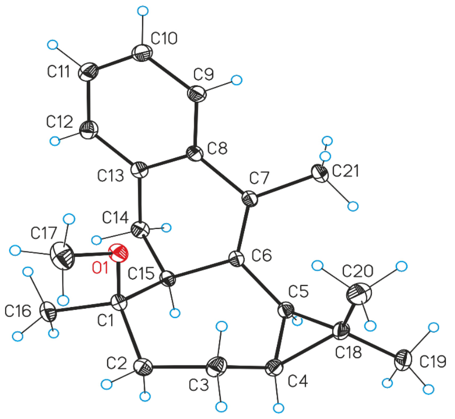

ORTEP representation of **17b** with 50% probability of the thermal ellipsoids

Table 3. Crystal data and structure refinement of 17b (CCDC 1906613).

Identification code	HA011	
Empirical formula	C21 H28 O	
Formula weight	296.43	
Temperature	100(2) K	
Wavelength	0.71073 Å	
Crystal system	Monoclinic	
Space group	P2(1)/c	
Unit cell dimensions	a = 8.99910(10) Å	α = 90°.
	b = 9.52500(10) Å	β = 91.7530(10)°.
	c = 9.7532(2) Å	γ = 90°.
Volume	1692.38(3) $Å^3$	
Z	4	
Density (calculated)	1.163 Mg/m^3	
Absorption coefficient	0.069 mm^{-1}	
F(000)	648	
Crystal size	0.25 x 0.25 x 0.10 mm^3	
Theta range for data collection	2.264 to 39.136°.	
Index ranges	-15<=h<=15,-16<=k<=16,-34<=l<=33	
Reflections collected	59329	
Independent reflections	9658[R(int) = 0.0342]	
Completeness to theta =39.136°	97.399994%	
Absorption correction	Empirical	
Max. and min. transmission	0.995 and 0.765	
Refinement method	Full-matrix least-squares on F^2	
Data / restraints / parameters	9658/ 0/ 204	
Goodness-of-fit on F^2	1.042	
Final R indices [I>2sigma(I)]	R1 = 0.0360, wR2 = 0.1073	
R indices (all data)	R1 = 0.0427, wR2 = 0.1115	
Largest diff. peak and hole	0.629 and -0.192 e.$Å^{-3}$	

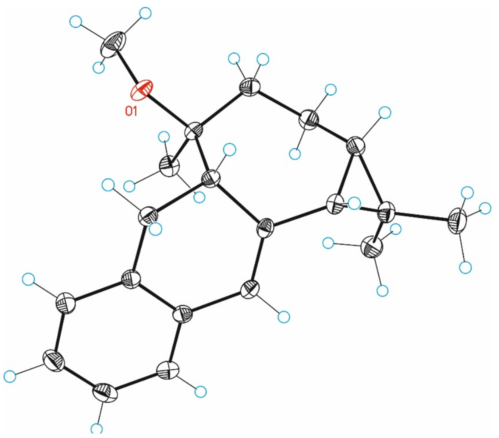

ORTEP representation of **18** with 50% probability of the thermal ellipsoids

Table 4. Crystal data and structure refinement for **18** (CCDC 1906611).

Identification code	mo_HB2_217_0m	
Empirical formula	C20 H26 O	
Formula weight	282.41	
Temperature	100(2) K	
Wavelength	0.71073 Å	
Crystal system	Monoclinic	
Space group	P2(1)/c	
Unit cell dimensions	a = 7.638(3)Å	α= 90°.
	b = 18.786(8)Å	β = 91.986(19)°.
	c = 11.247(5)Å	γ = 90°.
Volume	1612.8(12) $Å^3$	
Z	4	
Density (calculated)	1.163 Mg/m^3	
Absorption coefficient	0.069 mm^{-1}	
F(000)	616	
Crystal size	0.25 x 0.16 x 0.06 mm^3	
Theta range for data collection	2.111 to 32.513°.	
Index ranges	-8<=h<=11,-27<=k<=22,-16<=l<=11	
Reflections collected	13604	
Independent reflections	4943[R(int) = 0.0361]	
Completeness to theta =32.513°	84.6%	
Absorption correction	Multi-scan	
Max. and min. transmission	_exptl_absorpt_correction_T_max 0.7464 and	
_exptl_absorpt_correction_T_min 0.6884		
Refinement method	Full-matrix least-squares on F^2	
Data / restraints / parameters	4943/ 0/ 194	
Goodness-of-fit on F^2	1.053	
Final R indices [I>2sigma(I)]	R1 = 0.0480, wR2 = 0.1225	
R indices (all data)	R1 = 0.0673, wR2 = 0.1332	
Largest diff. peak and hole	0.380 and -0.245 e.$Å^{-3}$	

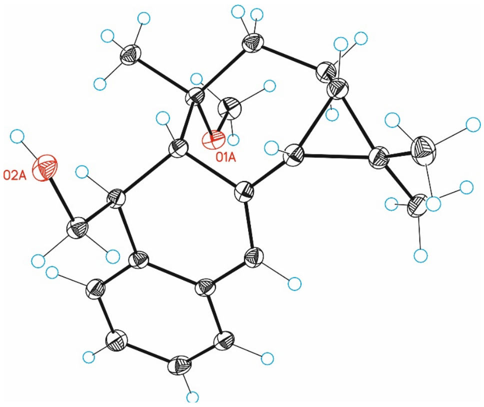

ORTEP representation of **21** with 50% probability of the thermal ellipsoids

Table 5. Crystal data and structure refinement for **21** (CCDC 1906612).

Identification code	HFB2-179	
Empirical formula	C21 H28 O2	
Formula weight	312.43	
Temperature	100(2) K	
Wavelength	0.71073 Å	
Crystal system	Triclinic	
Space group	P-1	
Unit cell dimensions	a = 10.3260(7)Å	α= 113.721(5)°.
	b = 12.6866(6)Å	β = 99.266(5)°.
	c = 14.8598(7)Å	γ = 100.653(5)°.
Volume	1690.48(17) $Å^3$	
Z	4	
Density (calculated)	1.228 Mg/m^3	
Absorption coefficient	0.077 mm^{-1}	
F(000)	680	
Crystal size	0.15 x 0.15 x 0.10 mm^3	
Theta range for data collection	2.245 to 30.782°.	
Index ranges	-14<=h<=14,-17<=k<=18,-21<=l<=19	
Reflections collected	29694	
Independent reflections	9385[R(int) = 0.1034]	
Completeness to theta =30.782°	88.8%	
Absorption correction	Multi-scan	
Max. and min. transmission	0.992 and 0.763	
Refinement method	Full-matrix least-squares on F^2	
Data / restraints / parameters	9385/ 6/ 433	
Goodness-of-fit on F^2	0.986	
Final R indices [I>2sigma(I)]	R1 = 0.0664, wR2 = 0.1724	
R indices (all data)	R1 = 0.0778, wR2 = 0.1841	
Largest diff. peak and hole	0.762 and -0.430 e.$Å^{-3}$	

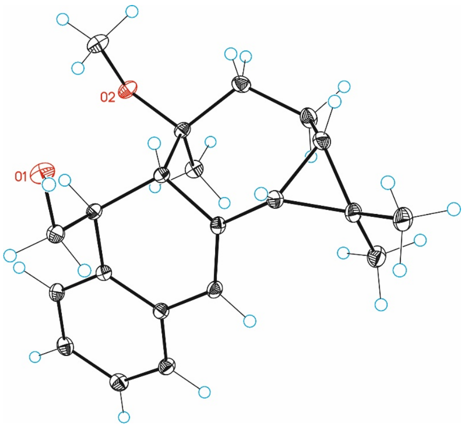

ORTEP representation of **23** with 50% probability of the thermal ellipsoids

Table 6. Crystal data and structure refinement for **23** (CCDC 1906608).

Identification code	mo_HB2291_0m	
Empirical formula	C21 H28 O2	
Formula weight	312.43	
Temperature	100(2) K	
Wavelength	0.71073 Å	
Crystal system	Monoclinic	
Space group	P2(1)/n	
Unit cell dimensions	a = 8.0240(3)Å	α= 90°.
	b = 21.9741(8)Å	β = 93.6585(11)°.
	c = 9.8486(3)Å	γ = 90°.
Volume	1732.97(10) Å^3	
Z	4	
Density (calculated)	1.197 Mg/m^3	
Absorption coefficient	0.075 mm^{-1}	
F(000)	680	
Crystal size	0.20 x 0.10 x 0.10 mm^3	
Theta range for data collection	1.853 to 30.606°.	
Index ranges	-10<=h<=11,-31<=k<=25,-14<=l<=12	
Reflections collected	18128	
Independent reflections	5138[R(int) = 0.0213]	
Completeness to theta =30.606°	96.100006%	
Absorption correction	Multi-scan	
Max. and min. transmission	0.993 and 0.961	
Refinement method	Full-matrix least-squares on F^2	
Data / restraints / parameters	5138/ 0/ 213	
Goodness-of-fit on F^2	1.041	
Final R indices [I>2sigma(I)]	R1 = 0.0393, wR2 = 0.1056	
R indices (all data)	R1 = 0.0449, wR2 = 0.1096	
Largest diff. peak and hole	0.413 and -0.193 e.Å^{-3}	
